# Comparative Analysis of Gene Expression Patterns for Oral Epithelial Cell Functions in Periodontitis

**DOI:** 10.3389/froh.2022.863231

**Published:** 2022-05-23

**Authors:** Octavio A. Gonzalez, Sreenatha Kirakodu, Linh M. Nguyen, Luis Orraca, Michael J. Novak, Janis Gonzalez-Martinez, Jeffrey L. Ebersole

**Affiliations:** ^1^Center for Oral Health Research, College of Dentistry, University of Kentucky, Lexington, KY, United States; ^2^Division of Periodontology, College of Dentistry, University of Kentucky, Lexington, KY, United States; ^3^Department of Biomedical Sciences, School of Dental Medicine, University of Nevada Las Vegas, Las Vegas, NV, United States; ^4^School of Dentistry, University of Puerto Rico, San Juan, Puerto Rico

**Keywords:** non-human primate, aging, epithelium, periodontitis, gingival transcriptome

## Abstract

The structure and function of epithelial cells are critical for the construction and maintenance of intact epithelial surfaces throughout the body. Beyond the mechanical barrier functions, epithelial cells have been identified as active participants in providing warning signals to the host immune and inflammatory cells and in communicating various detailed information on the noxious challenge to help drive specificity in the characteristics of the host response related to health or pathologic inflammation. Rhesus monkeys were used in these studies to evaluate the gingival transcriptome for naturally occurring disease samples (GeneChip® Rhesus Macaque Genome Array) or for ligature-induced disease (GeneChip® Rhesus Gene 1.0 ST Array) to explore up to 452 annotated genes related to epithelial cell structure and functions. Animals were distributed by age into four groups: ≤ 3 years (young), 3–7 years (adolescent), 12–16 years (adult), and 18–23 years (aged). For naturally occurring disease, adult and aged periodontitis animals were used, which comprised 34 animals (14 females and 20 males). Groups of nine animals in similar age groups were included in a ligature-induced periodontitis experiment. A buccal gingival sample from either healthy or periodontitis-affected tissues were collected, and microarray analysis performed. The overall results of this investigation suggested a substantial alteration in epithelial cell functions that occurs rapidly with disease initiation. Many of these changes were prolonged throughout disease progression and generally reflect a disruption of normal cellular functions that would presage the resulting tissue destruction and clinical disease measures. Finally, clinical resolution may not signify biological resolution and represent a continued risk for disease that may require considerations for additional biologically specific interventions to best manage further disease.

## Introduction

The structure and function of epithelial cells are critical for the integrity of epithelial surfaces throughout the body. These structures limit the access of pathogens that interact with mucosal surfaces to invade and initiate disease [1]. Beyond the mechanical barrier functions, epithelial cells have been identified as active participants in providing warning signals to the host immune and inflammatory cells and in communicating various detailed information on the noxious challenge to help drive specificity in the characteristics of the host response related to the nature of the challenge [2–6].

The signals that elicit epithelial responses include microbial-associated molecular patterns (MAMPs) that trigger inflammatory and antimicrobial peptide responses and the release of danger-associated molecular patterns (DAMPs) [7–9] to communicate the challenge to the inflammatory/immune cell populations at mucosal surfaces. Thus, knowledge of this array of functions of epithelial cells continues to evolve as critical determinants of maintaining host integrity from infection in the local mucosal environment [10–12].

Accumulation of microbial biofilms supra- and sub-gingivally trigger gingival tissue reactions, leading to biologic and clinical responses described as gingivitis [13–15]. Persistence of this microbial stimulus leads to the localized immunoinflammatory lesion of periodontitis with dynamic tissue/cellular effects, including ulceration of the epithelium, vasculitis, infiltration of an array of inflammatory cells, loss of connective tissue integrity, and resorption of alveolar bone the hallmark of the disease [3, 13, 16, 17]. Moreover, epidemiological evidence clearly demonstrates that periodontitis expression and severity increase significantly with age [18–20].

We have previously described the use of the non-human primate model of naturally occurring and ligature-induced periodontitis to identify gingival tissue transcriptome patterns and to examine an array of biologic pathways that are altered by disease and affected by aging [21–30]. Recently, we identified unique features of the transcriptome related to epithelial cell biology that is focused on changes that occur with aging in clinically healthy gingival tissues [21, 31]. The results indicated that in younger animals, the expression of epithelial genes appeared more developmentally flexible. In the older animals, the transcriptome associated with epithelial cell functions appeared to reflect a less regulated response to the surrounding environment and may be less able to effectively resolve the noxious challenge from the bacteria, leading to an increased potential risk for destruction of periodontal tissues.

As epithelial cell functions and an intact epithelium are critical to periodontal health, we extended this non-human primate model to pattern the transcriptome of gingival tissues with naturally occurring and ligature-induced periodontitis with aging as a co-factor for gene expression profiles.

## Methods

### Non-human Primate Model and Oral Clinical Evaluation

Rhesus monkeys (*Macaca mulatta*) housed at the Caribbean Primate Research Center (CPRC) at Sabana Seca, Puerto Rico were used in these studies. As reported previously, the non-human primates are fed a 20% protein, 5% fat, and 10% fiber commercial monkey diet (diet 8773, Teklad NIB primate diet modified: Harlan Teklad). The diet is supplemented with fruits and vegetables, and water is provided *ad libitum* in an enclosed corral setting. A protocol was approved by the Institutional Animal Care and Use Committee (IACUC) of the University of Puerto Rico, allowing clinical measures of the periodontium, including probing pocket depth (PPD), bleeding on probing (BOP; 0–5 scale), as we have described previously, and the collection of the gingival tissue biopsies [32].

In the cross-sectional study, healthy animals (5–7/group) were distributed by age into four groups: ≤ 3 years (young), 3–7 years (adolescent), 12–16 years (adult), and 18–23 years (aged). Adult and aged periodontitis animals (11 additional animals) were used, since younger animals do not develop naturally occurring disease and comprise 34 animals (14 females and 20 males). A Maryland/Moffit probe was used (1–10 mm markings; color coded) on the facial aspect of the teeth and 2 proximal sites per tooth (mesio- and disto-buccal), excluding the canines and 3rd molars. Our previous experiences found that interproximal disease in the macaques is generally detected on buccal sites, and that isolated lesions on lingual sites without buccal involvement were very infrequent. Periodontal health was defined by mean PPD ≤ 3.0 mm and mean BOP ≤ 1 (0–5 scale) in a full mouth examination excluding 3rd molars and canines [30]. Assignment of periodontal disease at the animal level required a mean PPD of >3 mm and required multiple sites on separate teeth exhibiting PPD >4 mm. Inclusion in the healthy group required no sites with >3 mm PPD. The sampled sites in the periodontitis groups were diseased teeth documented by assessment of the presence of BOP >1 and probing pocket depth >4 mm, as we have previously described [33].

The ligature-induced disease model has been previously described and used a total of 36 animals (17 males; 19 females), with 9 animals per same age groups [32]. In this model, baseline clinical and gingival tissue samples were obtained, followed by the placement of a ligature around selected premolar and molar teeth. Clinical and biological samples were obtained at 2 weeks for disease initiation, and 1 and 3 months for disease progression. After the 3-month sampling, the ligatures are removed, which leads to no further progression of destructive disease and a general resolution of inflammation by sampling at 5 months. The clinical characteristics of the animals in the naturally occurring and ligature-induced disease are presented in Table 1.

**Table 1:** Clinical features of naturally occurring (full-mouth measures) and ligature-induced disease (only ligated-teeth) in the nonhuman primates.

**Group**	**Clinical Category**	**BOP (Δ vs. Adult Health)**	**PPD (Δ vs. Adult Health)**
**Naturally-Occurring**			
Young	Health	0.277	0.529
Adolescent	Health	0.807	0.892
Adult	Health	1	1
Aged	Health	0.866	0.946
Adult	Disease	1.20	1.22
Aged	Disease	1.24	1.26
**Ligature-Induced**			
Young	Health	0	0.4
	Disease	3.08	1.32
	Resolution	1.20	1.16
Adolescent	Health	0.15	0.72
	Disease	3.28	1.47
	Resolution	1.69	1.16
Adult	Health	1	1
	Disease	2.64	2.09
	Resolution	ND	ND
Aged	Health	0.69	1.16
	Disease	2.41	2.14
	Resolution	1.78	1.32

*ND denotes that clinical data for the adult resolution samples was not available*.

### Tissue Sampling and Gene Expression Microarray Analysis

Gingival tissue samples of healthy or disease sites were surgically collected, as we have previously described [28, 29, 34]. Briefly, a buccal gingival sample from either healthy or periodontitis-affected tissue from the premolar/molar maxillary region of each animal was taken using a standard gingivectomy technique that included the removal of an interproximal dental papilla with the affected pocket. Samples were maintained frozen at−80°C in RNAlater solution until RNA preparation for microarray and real time RT-PCR analysis. Total RNA was isolated from tissues using TRizol reagent (Invitrogen, Carlsbad, CA, USA). After cleaning with Qiagen RNeasy mini kit (Qiagen, Valencia, CA, USA), all microarray RNA expression analyses were done at the University of Kentucky Microarray facility. Individual animal tissue RNA samples were submitted to the UK Microarray Core Facility, and RNA quality was assessed with an Agilent 2100 Bioanalyzer (Agilent Technologies, Santa Clara, CA, USA). Reverse transcription of equal amounts of RNA from each sample was performed, followed by hybridization to either GeneChip® Rhesus Macaque Genome Array (Affymetrix, Santa Clara, CA, USA) for naturally-occurring disease samples or the GeneChip® Rhesus Gene 1.0 ST Array (Affymetrix, Santa Clara, CA, USA) for ligature-induced disease samples similar to methods we have previously described [29, 33, 35].

### Data Analysis

Genes representing epithelial structure and functions (*n* = 452) targeted in the naturally occurring disease and ligature-induced disease analyses are identified in Table 2. Beyond the specific Affymetrix probe annotation provided by the company, we annotated within the GeneChip® Rhesus Gene 1.0 ST Array additional probes for the host genes in this microarray. These included unannotated probes, whereby the nucleotide base sequence (https://www.affymetrix.com/analysis/index.affx#1_2) for each probe ID was subjected to a Blast (https://blast.ncbi.nlm.nih.gov/Blast.cgi) query that identified the *M. mulatta* gene ID with the greatest percent identity for the specific sequence. We selected the most targeted gene ID that always showed >90% identity and routine >95% identity for annotating the gene list for the analysis.

**Table 2:** Targeted gene for functions of epithelium.

**Gene ID**	**Product, Fxn group**	**Gene ID**	**Product, Fxn group**	**Gene ID**	**Product, Fxn group**
COL17A1	Collagen, ECM structure	ITGA9	Integrin, Cell adhesion	NOTCH1	Transmembrane EGF, Kinases
COL1A1	Collagen, ECM structure	ITGAL	Integrin, Cell adhesion	NOTCH2	Transmembrane EGF, Kinases
COL1A2	Collagen, ECM structure	ITGAM	Integrin, Cell adhesion	NOTCH3	Transmembrane EGF, Kinases
COL3A1	Collagen, ECM structure	ITGAV	Integrin, Cell adhesion	NOTCH4	Transmembrane EGF, Kinases
COL5A1	Collagen, ECM structure	ITGAX	Integrin, Cell adhesion	PIK3C3	Lipid, Kinases
COL7A1	Collagen, ECM structure	ITGB1	Integrin, Cell adhesion	PIK3R4	Lipid, Kinases
FBLN5	Fibulin, ECM structure	ITGB2	Integrin, Cell adhesion	PRKAA1	AMP activated, Kinases
FBN1	Fibrillin, ECM structure	ITGB3	Integrin, Cell adhesion	PRKAA2	AMP activated, Kinases
FN1	Fibronectin, ECM structure	ITGB4	Integrin, Cell adhesion	PRKCG	PKC, Kinases
HSPG2	Heparin sulfate proteoglycan, ECM structure	ITGB5	Integrin, Cell adhesion	PRKCZ	PKC, Kinases
KRT1	Keratin, ECM structure	ITGB6	Integrin, Cell adhesion	PRKD1	PKD, Kinases
KRT10	Keratin, ECM structure	LGALS3	Galectin, Cell adhesion	RIPK1	Ser/thr, Kinases
KRT12	Keratin, ECM structure	MSN	Moesin, Cell adhesion	VPS13A	Vacuolar protein, Kinases
KRT13	Keratin, ECM structure	PVRL1	Poliovirus receptor-related, Cell, Adhesion	GSK3B	Glycogen synthase, Kinases
KRT14	Keratin, ECM structure	PVRL2	Poliovirus receptor-related, Cell adhesion	AGER	Glycation end products, Receptors
KRT15	Keratin, ECM structure	PVRL3	Poliovirus receptor-related, Cell adhesion	CD36	Scavenger, Receptors
KRT16	Keratin, ECM structure	PVRL4	Poliovirus receptor-related, Cell adhesion	CD44	Hyaluronic acide, Receptors
KRT17	Keratin, ECM structure	SELL	Selectin, Cell adhesion	CD59	C′ mediated lysis, Receptors
KRT18	Keratin, ECM structure	SELP	Selectin, Cell adhesion	EGFR	Epidermal growth factor, Receptors
KRT19	Keratin, ECM structure	SPP1	Secreted phosphoprotein, Cell adhesion	ESR1	Estrogen, Receptors
KRT2	Keratin, ECM structure	VTN	Vitronectin, Cell adhesion	F2R	Thrombin, Receptors
KRT20	Keratin, ECM structure	VWF	Von Willibrand factor, Cell adhesion	IL9R	Interleukin, Receptors
KRT23	Keratin, ECM structure	ACTN1	Actin, Cytoskeleton regulators	PECAM1	Platelet/endothelial, Receptors
KRT24	Keratin, ECM structure	ACTN2	Actin, Cytoskeleton regulators	PROCR	Protein C, Receptors
KRT25	Keratin, ECM structure	ACTN3	Actin, Cytoskeleton regulators	THBD	Thrombomodulin, Receptors
KRT27	Keratin, ECM structure	ACTN4	Actin, Cytoskeleton regulators	TNFRSF1A	TNF family, Receptors
KRT28	Keratin, ECM structure	ATP2C1	ATPase secretory pathway, Cytoskeleton regulators	TNFRSF6B	TNF family, Receptors
KRT3	Keratin, ECM structure	ATP2C2	ATPase secretory pathway, Cytoskeleton regulators	TRAF1	TNF associated, Receptors
KRT35	Keratin, ECM structure	CCDC19	Cilia/flagella associated protein, Cytoskeleton regulators	TRAF2	TNF associated, Receptors
KRT37	Keratin, ECM structure	DNM1	Dynamin, Cytoskeleton regulators	CDSN	Corneodesmosin, Junction proteins
KRT38	Keratin, ECM structure	ENTPD1	EctoATPase, Cytoskeleton regulators	DSC1	Desmocolin, Junction proteins
KRT4	Keratin, ECM structure	FLNA	Fliamin, Cytoskeleton regulators	DSC2	Desmocolin, Junction proteins
KRT5	Keratin, ECM structure	FLNB	Filamin, Cytoskeleton regulators	DSC3	Desmocolin, Junction proteins
KRT6A	Keratin, ECM structure	MAP1B	Microtubule, Cytoskeleton regulator	DSG1	Desmoglein, Junction proteins
KRT6B	Keratin, ECM structure	MAP2	Microtubule, Cytoskeleton regulator	DSG2	Desmoglein, Junction proteins
KRT6C	Keratin, ECM structure	PDGFRB	Platelet derived growth factor receptor, Cytoskeleton regulators	DSG3	Desmoglein, Junction proteins
KRT7	Keratin, ECM structure	RAC1	Ras family GTPase, Cytoskeleton regulators	DSP	Desmoplakin, Junction proteins
KRT71	Keratin, ECM structure	SMURF1	Ubiquitin ligase, Cytoskeleton regulators	EVPL	Envoplakin, Junction proteins
KRT72	Keratin, ECM structure	STX5	Syntaxin, Cytoskeleton regulators	F11R	F11 receptor, Junction proteins
KRT73	Keratin, ECM structure	TAGLN	Transgelin, Cytoskeleton regulators	GJA1	Gap junction, Junction proteins
KRT74	Keratin, ECM structure	TIAM1	T cell lymphoma invasion/metastases, Cytoskeleton regulator	GJA3	Gap junction, Junction proteins
KRT75	Keratin, ECM structure	TLN1	Talin, Cytoskeleton regulators	GJA4	Gap junction, Junction proteins
KRT76	Keratin, ECM structure	TLN2	Talin, Cytoskeleton regulators	GJA5	Gap junction, Junction proteins
KRT77	Keratin, ECM structure	VCL	Vinculin, Cytoskeleton regulators	GJA8	Gap junction, Junction proteins
KRT78	Keratin, ECM structure	WAS	Wiscott-aldrich syndrome, Cytoskeleton regulators	GJB1	Gap junction, Junction proteins
KRT79	Keratin, ECM structure	WASF1	Wiscott-aldrich syndrome Cytoskeleton regulators	GJB2	Gap junction, Junction proteins
KRT8	Keratin, ECM structure	WASL	Wiscott-aldrich syndrome Cytoskeleton regulators	GJB3	Gap junction, Junction proteins
KRT80	Keratin, ECM structure	ZYX	Zyxin, Cytoskeleton regulators	GJB4	Gap junction, Junction proteins
KRT84	Keratin, ECM structure	ALOX5	Lipoxygenase, Inflammation	GJB5	Gap junction, Junction proteins
KRT85	Keratin, ECM structure	APOH	Apolipoprotein, Inflammation	GJC2	Gap junction, Junction proteins
KRT9	Keratin, ECM structure	CCL2	MCP-1, Inflammation	GJC3	Gap junction, Junction proteins
LAD1	Ladinin, ECM structure	CCL5	RANTES, Inflammation	GJD2	Gap junction, Junction proteins
LAMA3	Laminin, ECM structure	CCL7	MCP-3, Inflammation	JAM2	Junctional adhesion, Junction proteins
LAMA5	Laminin, ECM structure	CXCL10	IP-10, Inflammation	JAM3	Junctional adhesion, Junction proteins
LAMB3	Laminin, ECM structure	CXCL11	I-TAC, Inflammation	JUP	Plakoglobin, Junction proteins
LAMC2	Laminin, ECM structure	CXCL17	DC/monocyte chemokine, Inflammation	MAGI1	Guanylate kinase, Junction proteins
PRELP	Prolargin proteoglycan, ECM structure	CXCL2	MIP-2α, Inflammation	MAGI2	Guanylate kinase, Junction proteins
SPARC	Osteonectin, ECM structure	CXCL5	ENA-78, Inflammation	OCLN	Occludin, Junction proteins
VCAN	Versican, ECM structure	IKBKB	NFκB inhibitor, Inflammation	PKP1	Plakophilin, Junction proteins
VIM	Vimentin, ECM structure	IL1RN	IL-1 receptor antagonist, Inflammation	PKP2	Plakophilin, Junction proteins
CHI3L1	Chitinase, ECM remodeling	IL23A	Cytokine, Inflammation	PKP3	Plakophilin, Junction proteins
CTSG	Cathepsin, ECM remodeling	LIF	Leukemia inhibitory factor, Inflammation	PKP4	Plakophilin, Junction proteins
CTSK	Cathepsin, ECM remodeling	NFKB1	NFκB, Inflammation	PLEC1	Plectin, Junction proteins
ELA2	Elastase, ECM remodeling	NFKBIA	NFκB, Inflammation	PNN	Pinin, Junction proteins
F13A1	Coagulation factor XIII, ECM remodeling	OSM	Oncostatin M, Inflammation	PPL	Periplakin, Junction proteins
F3	Thromboplastin, ECM remodeling	PTGS2	Cox2, Inflammation	TJAP1	Tight junction associated, Junction proteins
LOX	Lysyl oxidase, ECM remodeling	TNF	Tumor necrosis factor, Inflammation	TJP1	Tight junction, Junction proteins
MMP1	Matrix metalloproteinase, ECM remodeling	ARHGEF2	Microtubule regulated, Growth factors	TJP2	Tight junction, Junction proteins
MMP2	Matrix metalloproteinase, ECM remodeling	BMP1	Bone morphogenetic protein, Growth factors	CAMP	Cathelicidin, AMPs
MMP7	Matrix metalloproteinase, ECM remodeling	BMP2	Bone morphogenetic protein, Growth factors	DEFA1	Defensins, AMPs
MMP9	Matrix metalloproteinase, ECM remodeling	BMP7	Bone morphogenetic protein, Growth factors	DEFA4	Defensins, AMPs
PLAT	Plasminogen activator, ECM remodeling	CTGF	Connective tissue, Growth factors	DEFA5	Defensins, AMPs
PLAU	Plasminogen activator, ECM remodeling	EGF	Epidermal, Growth factors	DEFA6	Defensins, AMPs
PLAUR	Plasminogen activator receptor, ECM remodeling	FGF10	Fibroblast, Growth factors	DEFB1	Defensins, AMPs
PLOD1	Lysyl hydroxylase, ECM remodeling	FGF7	Fibroblast, Growth factors	DEFB103A	Defensins, AMPs
PLOD2	Lysyl hydroxylase, ECM remodeling	GNG11	G-protein, Growth factors	DEFB104A	Defensins, AMPs
SERPINE1	PAI-1, ECM remodeling	PPBP/CXCL7	Connective tissue, Growth factors	DEFB105A	Defensins, AMPs
SERPINF1	Alpha-2 antiplasmin, ECM remodeling	PPP2CA	Microtubules, Growth factors	DEFB106A	Defensins, AMPs
SERPINF2	Alha-2 antiplasmin, ECM remodeling	PTEN	Tumor suppressor, Growth factors	DEFB108B	Defensins, AMPs
TIMP1	Metallopeptidase inhibitor, ECM remodeling	PTP4A1	Phosphatase, Growth factors	DEFB118	Defensins, AMPs
CAV1	Calveolin, Cell adhesion	RHOA	Ras homolog, Growth factors	DEFB119	Defensins, AMPs
CAV2	Calveolin, Cell adhesion	TGFB1	Transforming, Growth factors	DEFB121	Defensins, AMPs
CAV3	Calveolin, Cell adhesion	TGFB2	Transforming, Growth factors	DEFB122	Defensins, AMPs
CDH1	Cadherin, Cell adhesion	TGFB3	Transforming, Growth factors	DEFB123	Defensins, AMPs
CDH2	Cadherin, Cell adhesion	TMEFF1	EGF-like, Growth factors	DEFB125	Defensins, AMPs
CDH3	Cadherin, Cell adhesion	TSPAN13	Tetraspanin, Growth factors	DEFB126	Defensins, AMPs
CDH4	Cadherin, Cell adhesion	VEGFA	Vascular, Growth factors	DEFB127	Defensins, AMPs
CDH5	Cadherin, Cell adhesion	WISP1	Connective, Growth factors	DEFB129	Defensins, AMPs
CTNNA1	Catenin, Cell adhesion	WNT5B	Adipogenesis, Growth factors	DEFB132	Defensins, AMPs
CTNNA2	Catenin, Cell adhesion	AKT1	Ser/thr, Kinases	DEFB4	Defensins, AMPs
CTNNA3	Catenin, Cell adhesion	CHUK	IKK-α, Kinases	GZMA	Granzyme, AMPs
CTNNAL1	Catenin, Cell adhesion	CSNK2A1	Casein, Kinases	PLA2G2A	Phospholipase, AMPs
CTNNB1	Catenin, Cell adhesion	CSNK2A2	Casein, Kinases	PLUNC	Palate/lung/nasal, AMPs
CTNNBIP1	Catenin, Cell adhesion	DBF4	Zinc finger, Kinases	CEBPA	Leu zipper, Transcription factors
CTNNBL1	Catenin, Cell adhesion	JAG1	Notch signaling, Kinases	EHF	ETS homologous, Transcription factors
CTNND1	Catenin, Cell adhesion	MAP2K1	Mitogen activated, Kinases	ETS1	Proto-oncogene, Transcription factors
CTNND2	Catenin, Cell adhesion	MAP2K3	Mitogen activated, Kinases	JAK3	Janus kinase, Transcription factors
DES	Desmin, Cell adhesion	MAP2K4	Mitogen activated, Kinases	JUNB	Proto-oncogene, Transcription factors
ICAM1	Intracellular adhesion, Cell adhesion	MAP2K6	Mitogen activated, Kinases	KRAS	Proto-oncogene, Transcription factors
ICAM2	Intracellular adhesion, Cell adhesion	MAP2K7	Mitogen activated, Kinases	MITF	Melaninogensis, Transcription factors
ITGA1	Integrin, Cell adhesion	MAP3K1	Mitogen activated, Kinases	NFE2L2/NRF2	Nuclear Factor, Erythroid 2 Like 2
ITGA2	Integrin, Cell adhesion	MAP3K14	Mitogen activated, Kinases	RAF1	Proto-oncogene, Transcription factors
ITGA3	Integrin, Cell adhesion	MAP3K5	Mitogen activated, Kinases	TCF3	Ig, Transcription factors
ITGA4	Integrin, Cell adhesion	MAPK1	Mitogen activated, Kinases	TWIST1	bHLH, Transcription factors
ITGA5	Integrin, Cell adhesion	MAPK13	Mitogen activated, Kinases	ZEB1	Zinc finger homeobox, Transcription factors
ITGA6	Integrin, Cell adhesion	MAPK14	Mitogen activated, Kinases	ZEB2	Zinc finger homeobox, Transcription factors
ITGA7	Integrin, Cell adhesion	MAPK3	Mitogen activated, Kinases		
ITGA8	Integrin, Cell adhesion	MAPK8	Mitogen activated, Kinases		

The expression intensities across the samples were estimated using the Robust Multi-array Average (RMA) algorithm with probe-level quintile normalization, as implemented in the Partek Genomics Suite software version 6.6 (Partek, St. Louis, MO). The differential expression was initially compared using one way ANOVA across time points within an age group. For genes that had significant mean differences, two sample *t*-tests were used to investigate differences comparing baseline healthy to disease and resolution samples. Statistical significance was considered by a *p* < 0.05 which was adjusted for the number of correlations. The naturally occurring data have been uploaded into ArrayExpress data base (www.ebi.ac.uk) under accession number E-MTAB-1977, and the ligature-induced disease data have been uploaded into GEO accession GSE180588 (https://www.ncbi.nlm.nih.gov/gds). Correlation analyses with clinical parameters were determined using a Pearson Correlation Coefficient with a *p* < 0.005. Z-scores were calculated for each gene across all groups [Z=(x-μ)/σ)] with x gene expression value, μ means across the samples, and σ standard deviation across all samples.

The means of transcript data across all monkeys in the same age and disease category were calculated to yield 20 monkey age-disease groups. Gene expression data of 429 genes and 20 monkey age-disease groups was normalized on a scale of 0.130–0.297, with the lowest value set at 0.130 and the highest value at 0.297 to generate the heatmap and dendrograms for the cluster analyses (BioVinci software, BioTuring Inc., San Diego, CA). The unrooted dendrogram was constructed. The fan dendrogram was constructed by calculating the distance matrix with the Euclidean method, followed by Ward 2 linkage clustering method using the Ape package in R version 5.5, depicting the transcript data of 164 genes that were determined from univariate analysis to be significant at *p* < 0.01 and/or a change of >1.5/<−1.5 fold compared to baseline values.

## Results

### Epithelial Gene Profile Changes in Naturally Occurring Periodontitis

**Figure 1:**
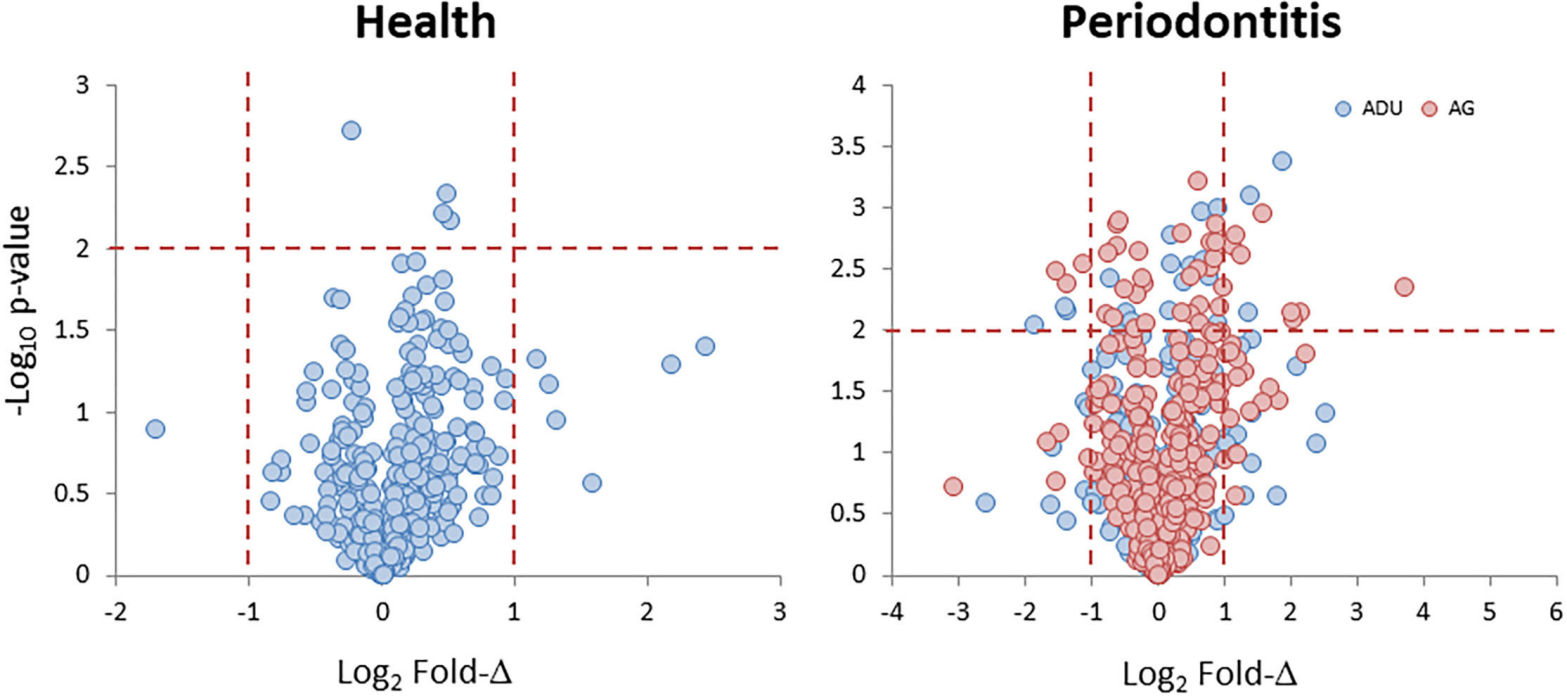
Volcano plots of gene expression levels in aged animals compared to the healthy adult tissue levels (Health) and in samples from periodontitis in adult and aged animals compared to healthy adult levels (Periodontitis). Each point denotes one of the 425 annotated genes related to fold difference and statistical difference from adult levels. The red dashed lines signify a *p* < 0.01 and a 2-fold increase or decrease in expression.

Data derived from the groups of healthy adult and aged animals and the same age groups with naturally occurring periodontitis are presented in Figure 1. The Health plot depicts the fold-difference and significance of genes expressed in aged compared to adult healthy tissues. The results showed only 11 of the epithelial cell-related genes that were significantly different at *p* ≤ 0.01 and/or increased/decreased by 2-fold or greater in the healthy aged tissues. The plot for the naturally occurring periodontitis tissues summarizes the gene expression in adult and aged diseased tissues compared to healthy adult samples. In these analyses, 39 and 19 genes were significantly different in the aged and adult disease samples, respectively. Furthermore, 31 (aged) and 26 (adult) genes were expressed at a 2-fold or greater difference in the disease samples.

Supplementary Figures 1A–G provides an overview of the normalized expression levels of the epithelial-related genes in functional categories of extracellular matrix (ECM) structural, remodeling, junction associated, cell adhesion, cytoskeleton regulation, growth factors, cell surface receptors, kinases, transcription factors, antimicrobial peptides, and inflammatory responses in the healthy and naturally occurring disease tissue samples from both age groups. These charts depict the epithelial-associated genes with their range of expression in the gingival tissues within each age group, and display genes with expression profiles that appear to differ between health and disease. Major expression of selected collagen, keratin, cornified epithelium, and laminin subunit genes is noted in the structural category. With ECM remodeling, cathepsins, kallikrein-related peptidases, plasminogen-related genes, TIMP1, and MMP1 and MMP9 are highly expressed in the gingival tissues. An important feature of the intact epithelial barrier is also the production of biomolecules that enables tight junctions between these cells. The data shows high expression of many of the genes for these molecules that are highly expressed in both health and disease with desmoplakin (DSP), desmocolin 2 (DSC2), and gap junction proteins (GJA1, GJB2) as prominent responses. There was a large number of cell adhesion genes that were highly expressed in the gingival tissues, with cadherins, LGALS3 (galectin 3), and SPRR2D (small proline rich protein 2D; keratinocyte protein) being prominent in this category. In the cytoskeleton regulation category, dominant responses in the gingival tissues are noted for Rac Family Small GTPase 1 (RAC1), TIAM Rac1 Associated GEF 1 (TIAM1), calmodulin-like 5 (CALML5), and vinculin (VCL). Ras Homolog Family Member A (RHOA), proteasome subunits, Phosphatase and Tensin Homolog (PTEN), Protein Tyrosine Phosphatase 4A1 (PTP4A1), and Transforming Growth Factor Beta Induced (TGFBI) were prominent growth factor responses in tissues from all groups. Epithelial cells also present a number of cell surface receptors that are crucial for cell-cell interactions in the gingival tissues. Dominant expression in this category was observed with BCAP31, CD164, CD44, CD9, and CD99 being highly expressed across the age groups. As an array of kinases are important in various cellular functions, including epithelial cell biology, many of these molecules are highly expressed in the gingival tissues. An array of transcription factor/regulator genes were quantified. Major genes of this category, expressed in the gingival tissues, included CEPBA and CEBPB, EHF, multiple proline rich proteins (e.g., PRR13), Twist Family BHLH Transcription Factor 1 (TWIST1), and Zinc Finger E-Box Binding Homeobox 2 (ZEB2). An important component of the epithelium at mucosal surfaces is the production of an array of antimicrobial peptides. These biomolecules have been shown to impact bacterial, viral, and fungal pathogens. Within the gingival tissues across all age groups were high levels of expression of beta-defensins (DEFB1, DEFB4, DEFB103A, DEFB121) and serine peptidase inhibitors Kazal types (SPINK5, SPINK7). Finally, as the epithelial cells are now recognized as important components of local inflammatory responses, we included a group of genes related to these responses. While it must be noted that many of these genes are not epithelial cell exclusive, dominant levels in this category included C1QBP, CXCL17, IL1A, IL1RN, MIF, and NFKBIZ and provide some insights into the overall biology of the gingival tissues.

**Figure 2:**
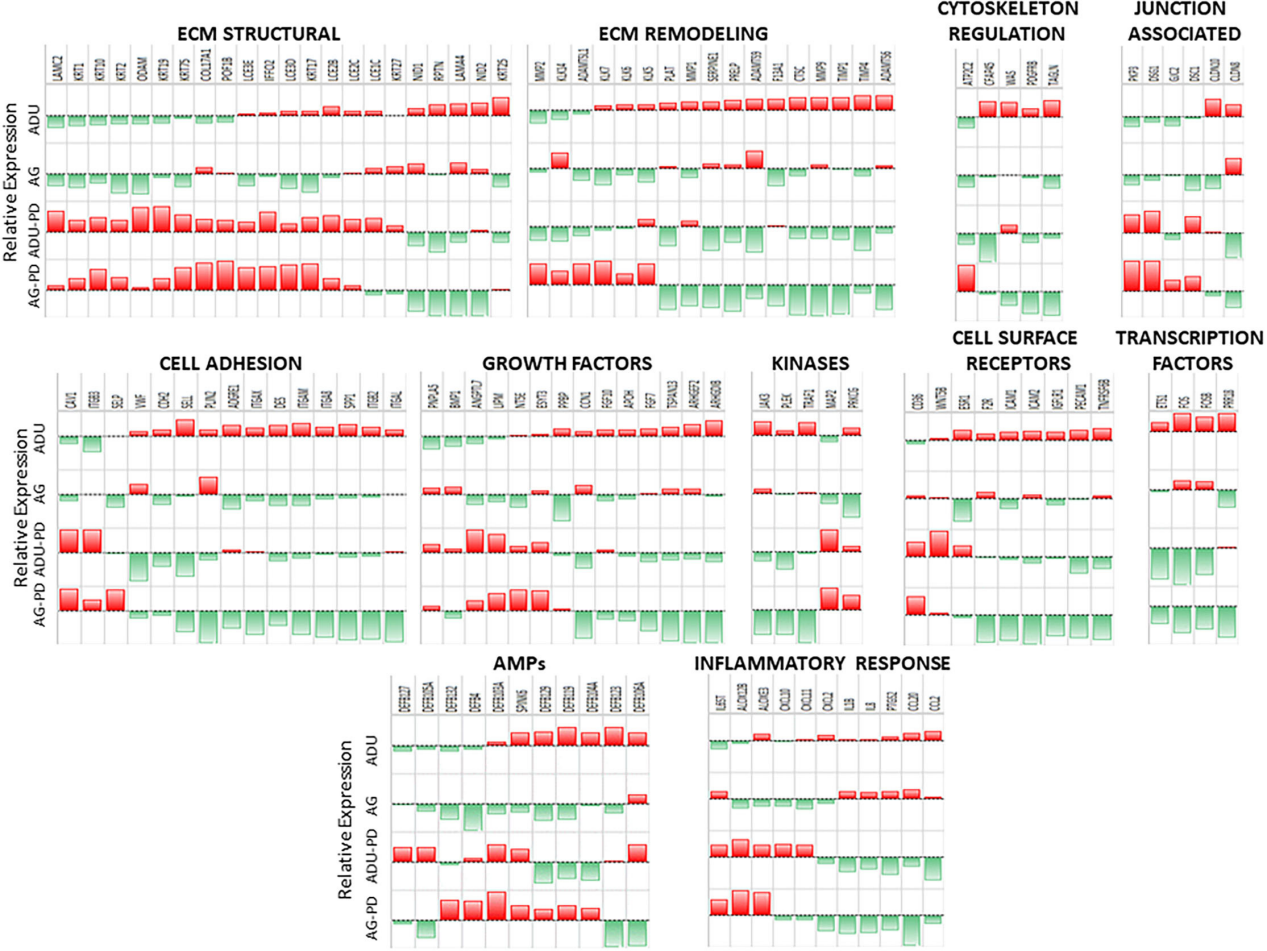
Heatmap of z-scores of fold differences in gene expression in adult and aged healthy and adult and aged periodontitis samples. The z-scores were determined across groups and within each gene. Genes are grouped into the 11 major categories. Red color and bar height denotes a low z-score, while green color and bar height signify a high z-score for that group and gene.

However, within this large assortment of epithelial cell-related genes, there was a more limited group of the genes that demonstrated differential expression in naturally occurring periodontitis. With ECM structural genes, the majority of genes were decreased with disease (Figure 2), while ECM remodeling genes were increased in the lesion tissues from adult and aged animals. Cell adhesion and cytoskeleton regulation genes were increased, with SPP1 demonstrating the most dramatic change in disease. While we explored numerous junction-associated genes, only a small subset showed decreased levels with disease. Of interest was that CLDN8 (claudin), a molecule regulating epithelial cell permeability, was substantially increased in disease samples (Figure 2). The growth factors were generally increased with disease, albeit ANGPTL7 (Angiopoietin Like 7) and PNPLA5 (Patatin Like Phospholipase Domain Containing 5) were decreased in both adults and aged samples (Figure 2). Cellular receptor genes were also generally increased, with only CD36 (thrombospondin receptor) demonstrating decreased levels in disease tissues. Few of the kinases were substantially affected with disease, generally showing the greatest increase in disease in aged animals. In addition, a few transcription factors were only upregulated in diseased tissues (Figure 2). Differential expression of the antimicrobial peptide genes was limited both with respect to the number of genes affected and the age of the animals. Finally, multiple inflammatory genes were affected by disease, demonstrating both elevations in various cytokines and a mixed differential expression of chemokines and enzymes in the inflammatory lipid pathway (Figure 2). Supplementary Figure 2 provides a heatmap summary of the z-scores for the array of epithelial genes for the individual animals in the healthy or periodontitis groups. The results showed some individual animal variation in each of the groups. However, the overall trends indicated decreases in ECM structural and cytoskeletal regulation and junction-associated genes in the adult and aged periodontitis specimens across all animals.

**Figure 3:**
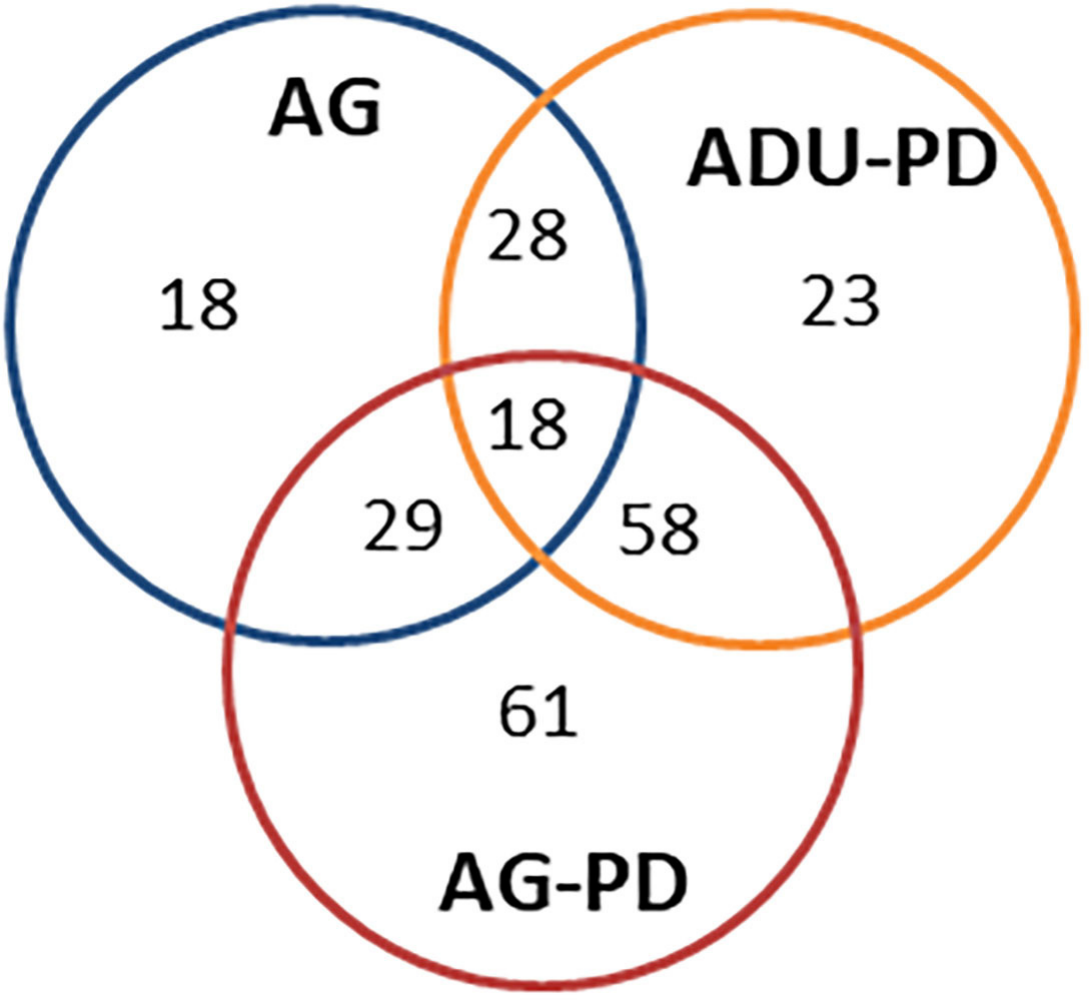
Venn diagram summarizing altered gene expression patterns in aging and naturally occurring periodontitis. The numbers denote unique or overlapping genes in each group that were significantly different and/or altered in expression by >1.5-fold compared to levels in healthy adult specimens.

Figure 3 provides a summary of the unique and overlapping AMF genes that were significantly different in the various groups and/or were altered by >1.5-fold in expression compared to levels in healthy adult samples. The Venn diagram demonstrates that the majority of altered genes were noted with the naturally-occurring periodontitis samples, with substantial overlap in the adult and aged group, along with a clearly increased number of uniquely elevated AMF genes in the aged periodontitis samples.

### Effects of Periodontitis on Gene Expression Related to Epithelial Cell Functions

**Figure 4:**
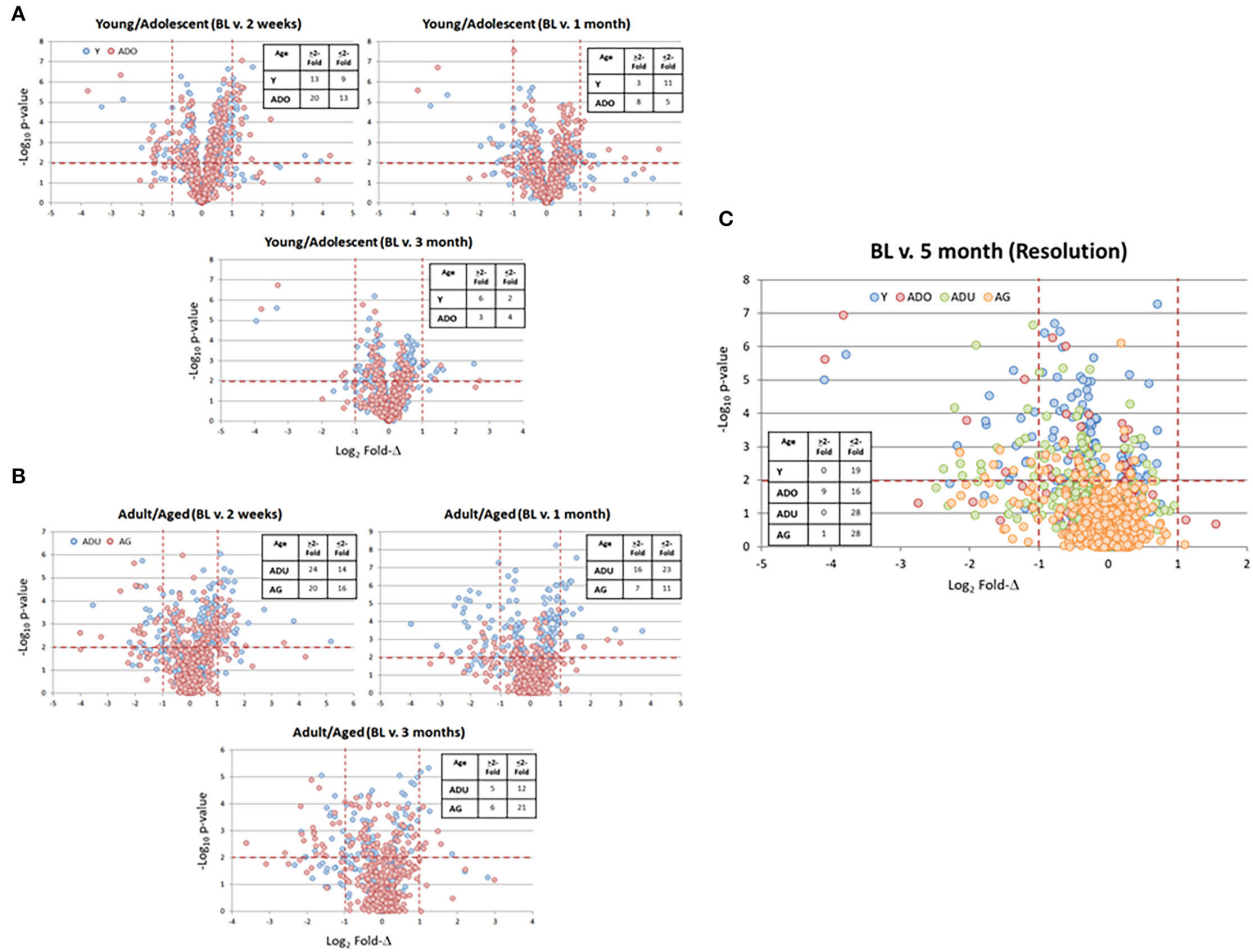
Volcano plots of gene expression differences in young and adolescent **(A)** and adult and aged **(B)** animals comparing the 3 time points during disease to the baseline healthy tissues. **(C)** The gene expression profiles in resolution samples for each of the age groups. Red lines denote *p* < 0.01 and fold-difference of >2. Each point denotes the value for one of the 452 annotated genes. The inset tables summarize the number of genes in each age group at each time point that was altered by >2-fold compared to baseline.

A benefit of the use of the non-human primate to model the immunobiology of periodontitis is the capacity to integrate a ligature-induced disease process into the experimental design. In this model, it allows a specific identification of the initiation, progression, and resolution of the disease lesions. Figure 4 provides volcano plots of differential gene expression profiles during disease in the young and adolescent animals (Figure 4A), and in the adult and aged animals (Figure 4B). Of note in the Y and ADO animals, a large number of genes were significantly (*p* < 0.01) differentially expressed at initiation (2 weeks), with somewhat fewer expressed during early (1 month) and late (3 month) disease progression. This was also emphasized with a greater number of genes showing a fold-difference compared to the samples from progressing disease. A similar pattern was noted in the adult and aged animals in relation to changes occurring with disease initiation. However, there was a predilection for an increased frequency of changes in the adult animals at 1 month, while this magnitude of significant differences was primarily noted in the aged animals at the 3 month time point. Figure 4C summarizes the characteristics of gene expression changes in the resolution samples from each of the age groups. The dominant pattern was a decreased level of these epithelial-related genes in all age groups with 50–75% more genes with these lower levels in resolved samples from the adult and aged groups.

Supplementary Figures 3A–3R summarizes the gene expression levels for the epithelial related genes at baseline (health), during disease, and with lesion resolution. Within the ECM structural category (Supplementary Figures 3A,B), multiple collagen, a subset of keratins, late cornified epithelium, laminin subunits, Repetin (RPTN), Fibrillin 1 (FBN1), and Nidogen $\frac{1}{2}$ (NID1/NID2) genes were all highly expressed in all age groups. ECM remodeling genes (Supplementary Figure 3C) were elevated for cathepsins, Kallikrein Related Peptidase 6 (KLK6), MMP2, Plasminogen Activator Tissue Type (PLAT), Serpin Family F Member 1 (SERPINF1), and TIMP Metallopeptidase Inhibitor 1 (TIMP1) in all age groups. Cytoskeleton regulation genes (Supplementary Figure 3D) included actinin, AHNAK Nucleoprotein (AHNAK), Ectonucleoside Triphosphate Diphosphohydrolase 1 (ENTPD1), Filamin A (FLNA), RAC1, TIAM1, VCL, and WASP Like Actin Nucleation Promoting Factor (WASL) genes across the age groups. Ten of the junction-associated genes (Supplementary Figure 3E) showed dominant responses, including Desmocollin 2 (DSC2), Desmoglein 1/3 (DSG1/3), F11 Receptor (F11R), gap junction proteins (GJA1/B2), Junction Plakoglobin (JUP), Plakophilin 1 (PKP1), and Periplakin (PPL) in all tissues. The dominant expression of cell adhesion genes (Supplementary Figures 3F,G) in all age groups were with Catenin Beta 1 (CTNNB1), Catenin Beta Interacting Protein 1 (CTNNBIP1), Fibronectin 1 (FN1), ITGA6/B1/B4 (integrin subunits), Moesin (MSN), and SPRR2D genes. A broad array of growth factors (Supplementary Figures 3H,I) were evaluated with Rho GDP Dissociation Inhibitor Beta (ARHGDIB), Protein Phosphatase 2 Catalytic Subunit Alpha (PPP2CA), PTEN, RHOA, and SLC2A1, showing the greatest expression in all age groups. The cell surface receptor signals (Supplementary Figures 3J,K) were dominated by Amyloid Beta Precursor Protein (APP), CD44, CD81, CD59, CD9, CD99, CD164, Corneodesmosin (CDSN), Epidermal Growth Factor Receptor (EGFR), and Platelet and Endothelial Cell Adhesion Molecule 1 (PECAM1) in all age groups. Dominant kinase (Supplementary Figures 3L,M) expression included Casein Kinase 2 Alpha $\frac{1}{2}$ (CSNK2A1/2), Erb-B2 Receptor Tyrosine Kinase 3 (ERBB3), Glycogen Synthase Kinase 3 Beta (GSK3B), Insulin Like Growth Factor Binding Protein 4 (IGFBP4), Jagged Canonical Notch Ligand 1 (JAG1), a number of map kinases, and Notch Receptor 2/3 (NOTCH2/3) in all age groups. Supplementary Figures 3N,O displays levels of transcription factors in the gingival tissues. ETS Homologous Factor (EHF), JunB Proto-Oncogene, AP-1 Transcription Factor Subunit (JUNB), Nuclear Factor, Erythroid 2 Like 2; NRF2 (NFE2L2), Snail Family Transcriptional Repressor 2 (SNAI2), and Signal Transducer And Activator Of Transcription 3 (STAT3). A limited subset of antimicrobial peptides (Supplementary Figure 3P) showed elevated levels in the gingival tissue in all age groups and included DEFB1, DEFB103A, DEFB4, and SPINK5/7. Finally, within the subset of inflammatory mediators (Supplementary Figures 3Q,R) that were targeted, CXCL17, IL1A, IL1RN, and LITAF predominated in all age groups.

**Figure 5:**
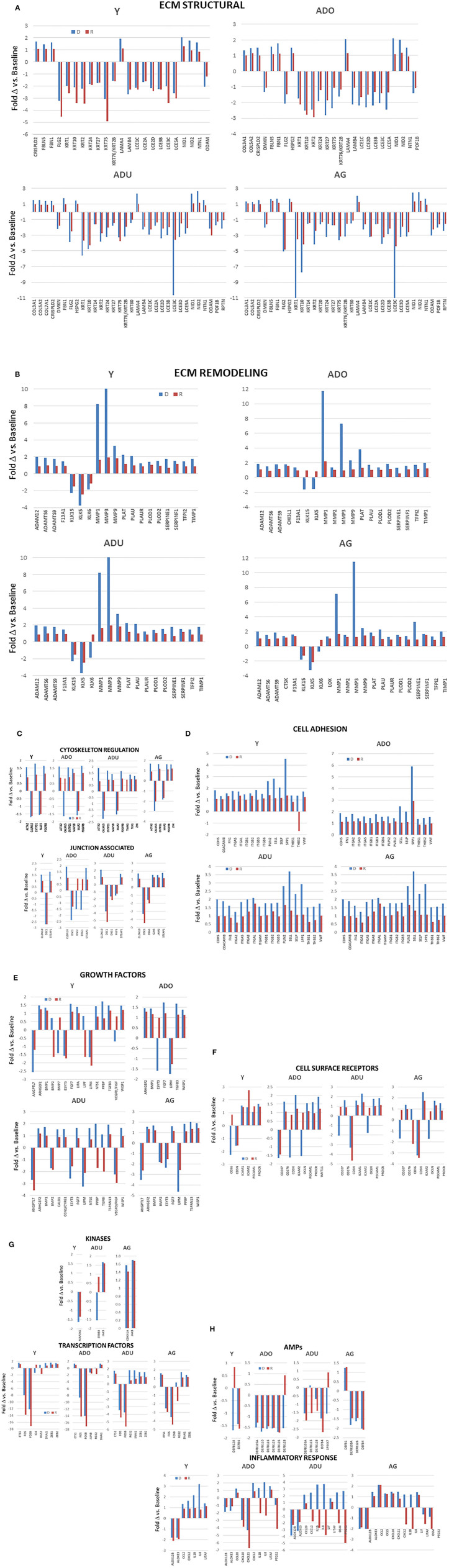
**(A–H)** Bars denote fold-difference of genes in each of the functional categories that were expressed at >1.5-fold in disease (D) or resolution (R) tissues compared to healthy baseline levels for each age group.

Within the larger array of various functional categories, beyond just the magnitude of expression, it was useful to highlight those genes related to epithelial cell functions that demonstrated a larger fold-change with disease and resolution comparted to heathy tissues (Figures 5A–H). The general outcomes of the focus in this subset of genes, as shown by the majority of ECM structural genes, were decreased in disease and resolution samples across all age groups. In contrast the ECM, remodeling genes were increased in all age groups, with MMPs demonstrating the greatest fold difference in expression with disease. Cytoskeleton regulation genes were generally increased except Calmodulin Like 5 (CALML5) that was decreased in all age samples. While we included a large array of junction associated genes, relatively few were increased or decreased at 1.5-fol or more. However, those that were affected showed a similar directional impact across all age groups. The altered cell adhesion genes overlapped across age groups and were virtually all increased in both disease and resolution samples. Examination of the growth factor responses also demonstrated variation in expression changes within the genes that were examined. However, the direction of change for a particular growth factor gene was generally conserved across the age groups. Also of note was that the changes were similar in the diseased and resolution samples. Generally, the cell surface receptors that were altered showed considerable overlap and were skewed toward increased levels in disease and resolution across the age groups. Few kinases were affected with a substantial fold difference from health, while a number of transcription factors related to epithelial cell functions were increased in the various age groups. In addition, a dramatic impact was noted with substantially decreased levels of FOS and FOSB in both disease and resolution samples in this disease model. The antimicrobial peptides altered in the various age group somewhat varied. However, the trend was toward decreased expression with disease that was maintained in resolution samples. Finally, the inflammatory response changes showed a large variation in differences in expression levels related to disease vs. resolution and within the various age groups. Of note was the consistent decrease in ALOX12B and ALOXE3 across age groups in disease and resolution, with each of these involved in lipid aspects of epithelial biology. In the older groups, the cytokines/chemokines were increased in disease and decreased in resolutions samples particularly in the adult group.

**Figure 6:**
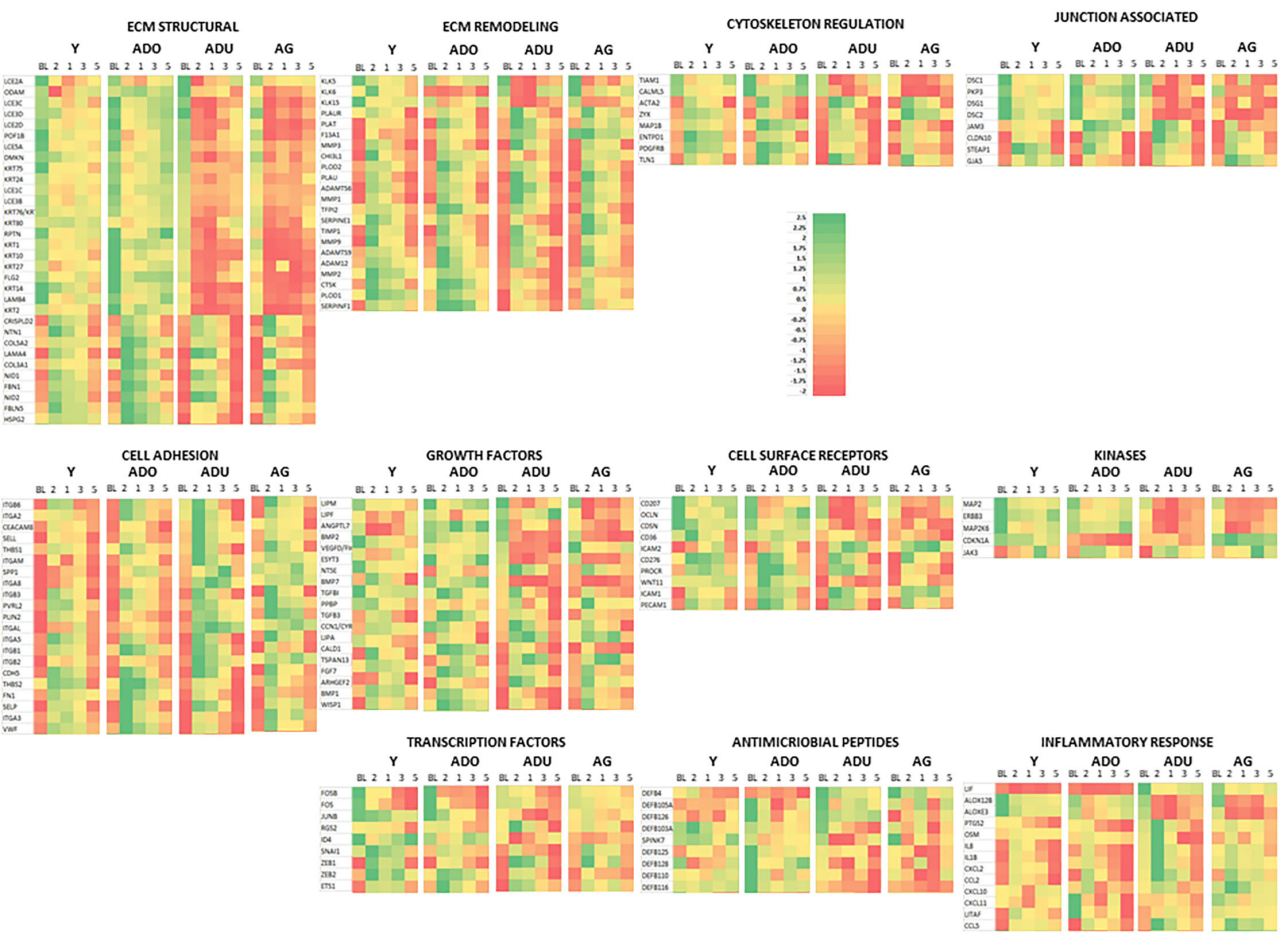
Heatmap of genes in each functional category that were significantly different from baseline expression at disease and/or resolution samples. A z-score was determined across time points and within each gene.

Figure 6 provides a heatmap representation of the genes within the various functional categories that significantly altered from baseline (health) compared to any of the disease time points and/or in resolution samples. Specific patterns are notable with the majority of ECM structure, growth factors, kinases, cell surface receptors, antimicrobial peptides, and transcription factor genes decreased with disease. This change was exacerbated in the adult and aged groups. In contrast, ECM remodeling, cell adhesion, inflammatory response, and cytoskeleton regulation genes were at lower levels in health and increased with disease. Also, this display demonstrates that the patterns of these gene levels in resolution samples most often moved toward the levels in the baseline samples. However, they generally did not return to the levels observed in healthy specimens.

### Epithelial-Related Gene Expression and Clinical Outcomes

**Figure 7:**
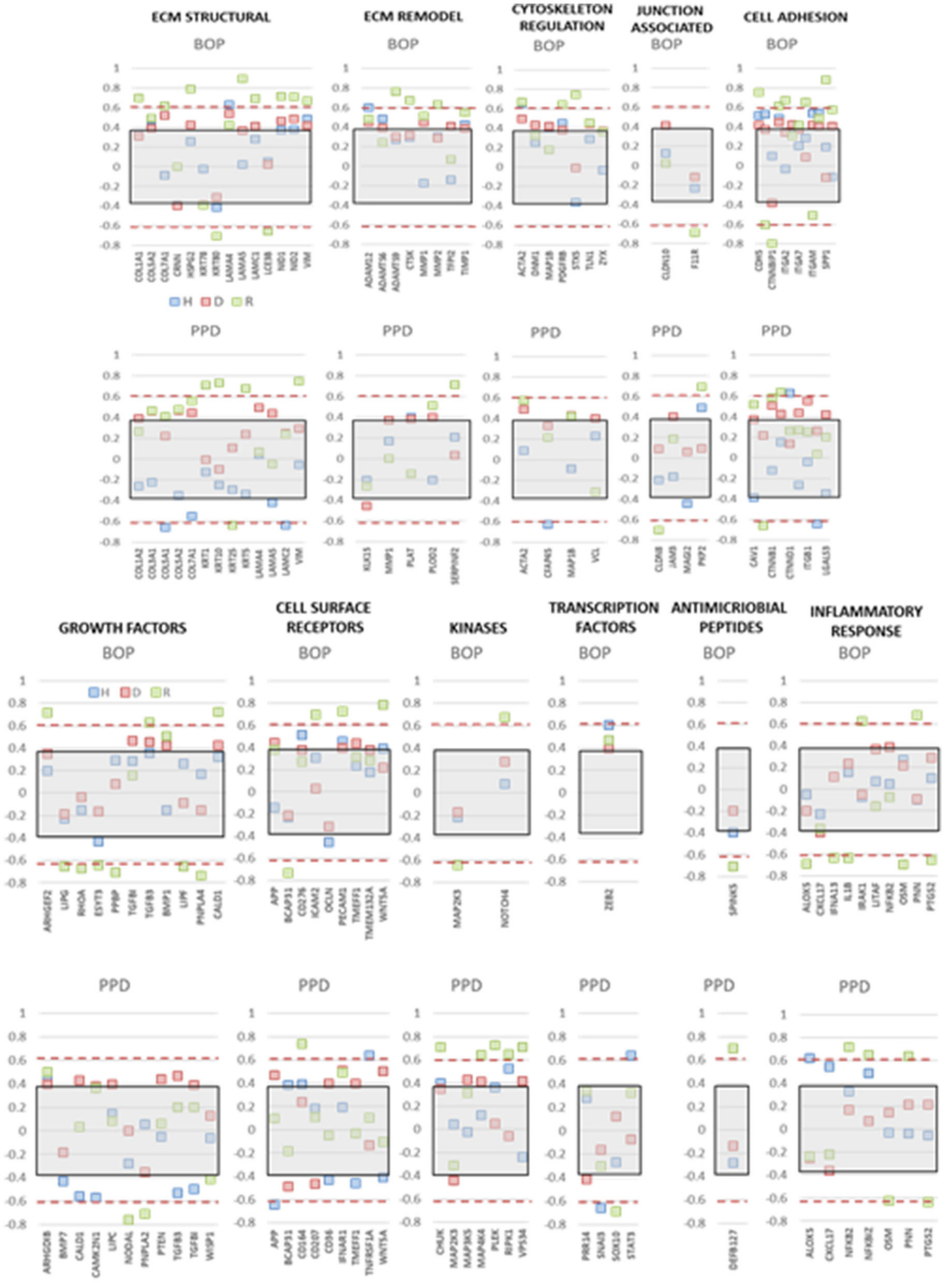
Correlation analysis of gene expression levels in adult and aged animals with clinical parameters of mean bleeding on probing (BOP) and mean probing pocket depth (PPD). The red dashed lines denote significance level of the correlation coefficient at *p* < 0.01 for the health (baseline 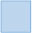) and resolution (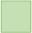) samples. The gray shaded rectangle denotes the significance level of the correlation coefficient at *p* < 0.01 for the pooled disease (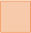) sample data.

The integrity of the epithelium and broader functions of the epithelial cells are critical for maintaining or returning to homeostasis. Thus, we examined the expression of this array of genes related to the clinical parameters of disease, e.g., bleeding on probing and probing pocket depth, in the adult and aged animals, reflecting the patterns that might be expected in chronic periodontitis in humans. Figure 7 summarizes correlation data presenting those genes in each functional category that showed a significant correlation with either of the clinical parameters in health, disease, or resolution samples. The data indicated that few genes in any functional category were significantly correlated with the clinical parameters in healthy samples. In contrast, multiple functional categories demonstrated frequent genes in resolution samples that were significantly correlated with BOP and/or PPD measures. This was particularly notable with positive correlations with ECM structure and remodeling, cell adhesion, cytoskeleton regulation, and cell surface receptors. The same categories were correlated in the disease samples, with the addition of growth factor and kinase genes.

**Figure 8:**
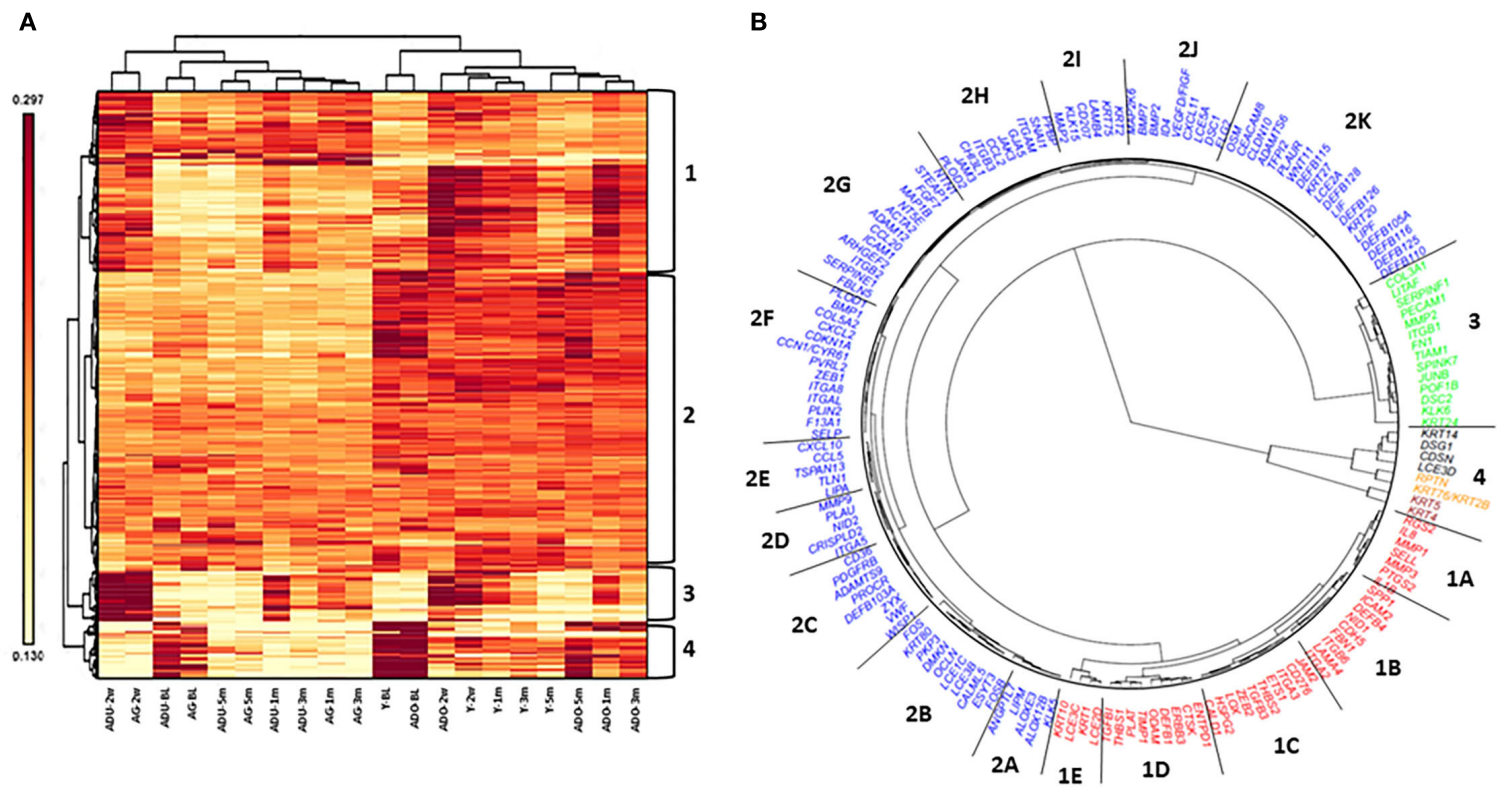
Heatmap and cluster analysis **(A)** of epithelial-related genes organized by age (Y, ADO, ADU, AG) and health (BL)/disease (2 w, 1 m, 3 m)/resolution (5 m) specimens. Numeric identifiers (1–4) provide major clustering of genes that discriminate the samples based upon age and/or disease characteristics of the samples. **(B)** Fan dendogram of the prominent gene IDs identifying the clusters (*p* < 0.01 and/or fold-difference >1.5/ < -1.5).

Figure 8A depicts the results of a cluster analysis of the 452 epithelial gene expression levels across all of the various functional groups. The results showed clear distinction of the expression of this set of genes between the younger (young and adolescent) and older (adult and aged) samples. Additionally, within these age categories, the baseline/healthy tissues and resolution samples generally demonstrated substantial similarities in expression, albeit the healthy samples were clustered somewhat distinct from the resolution samples. Within the disease time points, there existed age clustering, although there was rather minimal stratification comparing disease initiation vs. progression samples. Distinctive features of the gene expression groups show the array of genes in Cluster 1 discriminate the younger from older age groups across all time points. Moreover, within the age groups, the disease samples are unique compared to the healthy and resolution specimens. Cluster 2 was a large array of epithelial-associated genes that showed some differences in health and disease, but primarily discriminated based upon the age of the samples. Gene cluster 3 identified disease initiation samples (2 weeks) from the other samples. Cluster 4 genes differentiated younger and older baseline (healthy) samples from the other time points.

These relationships are also demarcated in a fan graphic analysis (Figure 8B). This analysis includes 164 of the genes that were identified to be significant at *p* < 0.01 and/or differed from baseline health by >1.5-fold at any of the time points. The results refined the cluster analytics and identified 18 clusters that discriminated the samples based on disease and age. Table 3 summarizes the functional categories of the genes in the various clusters. There were certain samples of these clusters that were skewed with certain functional categories. For example, 1B (cell adhesion), 1E, 2B, 2I, and 4 (ECM structural), and 2K (AMPs). However, seemingly more prevalent was a mixture of epithelial biologic functions in each of the clusters that contributed to the discrimination of the samples based upon age and disease status. This finding suggests a rather dynamic role for broad functions of the epithelial cells within the tissue microenvironment that contributes to homeostasis or in response to a noxious process-resulting disease.

**Table 3:** Characteristics of gene clusters. Functional categories (Fnx) are defined as follows: AMP, antimicrobial peptide; CA, cell adhesion; CSR, cell surface receptor; CR, cytoskeleton regulation; ER, ECM remodeling; ES, ECM structural; GF, growth factor; IR, inflammatory response; JA, junction associated; K, Kinase; TF, transcription factors.

**Cluster/Gene ID**	**Fxn**	**Cluster/Gene ID**	**Fxn**	**Cluster/Gene ID**	**Fxn**	**Cluster/Gene ID**	**Fxn**
**1A**		**2A**		**CCN1/CYR61**	**GF**	**2K**	
SELL	CA	ALOX12B	IR	CDKN1A	K	ADAMTS6	ER
IL1B	IR	ALOXE3	IR	ZEB1	TF	PLAUR	ER
IL8	IR	ANGPTL7	GF	F13A1	ER	TFPI2	ER
PTGS2	IR	LIPM	GF	PLOD1	ER	CEACAM8	CA
RGS2	TF	KLK5	ER	COL5A2	ES	LIF	IR
MMP1	ER	**2B**		**2G**		OSM	IR
MMP3	ER	CALML5	CR	ITGB2	CA	LIPF	GF
**1B**		ESYT3	GF	ACTA2	CR	WNT11	CSR
CDH5	CA	OCLN	CSR	MAP1B	CR	CLDN10	JA
ITGA2	CA	PKP3	JA	CCL20	IR	DEFB105A	AMP
ITGB6	CA	FOS	TF	ARHGEF2	GF	DEFB110	AMP
SPP1	CA	FOSB	TF	FGF7	GF	DEFB115	AMP
ICAM2	CSR	DMKN	ES	NT5E	GF	DEFB116	AMP
DEFB4	AMP	KRT80	ES	ICAM1	CSR	DEFB125	AMP
FBN1	ES	LCE1C	ES	STEAP1	JA	DEFB126	AMP
LAMA4	ES	LCE3B	ES	ADAM12	ER	DEFB128	AMP
NID1	ES	**2C**		SERPINE1	ER	KRT20	ES
**1C**		VWF	CA	FBLN5	ES	KRT27	ES
ITGA3	CA	PDGFRB	CR	NTN1	ES	LCE2A	ES
THBS2	CA	ZYX	CR	**2H**		**3**	
CALD1	GF	WISP1	GF	ITGAM	CA	KLK6	ER
TGFB3	GF	CD36	CSR	ITGB3	CA	MMP2	ER
CD276	CSR	PROCR	CSR	CCL2	IR	SERPINF1	ER
JAM2	JA	DEFB103A	AMP	PPBP	GF	FN1	CA
ETS1	TF	ADAMTS9	ER	JAK3	K	ITGB1	CA
ZEB2	TF	**2D**		GJA5	JA	TIAM1	CR
LOX	ER	ITGA5	CA	JAM3	JA	LITAF	IR
HSPG2	ES	MMP9	ER	SNAI1	TF	PECAM1	CSR
**1D**		PLAU	ER	CHI3L1	ER	DSC2	JA
THBS1	CA	CRISPLD2	ES	PLOD2	ER	SPINK7	AMP
ENTPD1	CR	NID2	ES	**2I**		JUNB	TF
TGFBI	GF	**2E**		MAP2	K	COL3A1	ES
ERBB3	K	TLN1	CR	CD207	CSR	KRT24	ES
DEFB1	AMP	CCL5	IR	KLK15	ER	POF1B	ES
CTSK	ER	CXCL10	IR	KRT2	ES	**4**	
PLAT	ER	LIPA	GF	KRT75	ES	CDSN	CSR
TIMP1	ER	TSPAN13	GF	LAMB4	ES	DSG1	JA
ODAM	ES	**2F**		**2J**		KRT14	ES
**1E**		ITGA8	CA	CXCL11	IR	KRT4	ES
KRT1	ES	ITGAL	CA	BMP2	GF	KRT5	ES
KRT10	ES	PLIN2	CA	BMP7	GF	KRT76/KRT2B	ES
LCE2D	ES	PVRL2	CA	VEGFD/FIGF	GF	LCE3D	ES
LCE3C	ES	SELP	CA	MAP2K6	K	RPTN	ES
		CXCL2	IR	DSC1	JA		
		BMP1	GF	ID4	TF		
				FLG2	ES		
				LCE5A	ES		

*Grey headers denotes gene clusters identified in Figure 8*.

The data were then subjected to a pathway analysis to highlight those pathways primarily represented by the altered expression of this gene array related to epithelial cell biology. Supplementary Table 1 identifies pathways representing the functional categories that are specifically related to epithelial cell signaling through multiple pathways of growth factors, proliferation, and response to a changing microenvironment.

## Discussion

An intact epithelial barrier is a critical component of the oral mucosa and clearly interfaces with the complex oral microbiome to maintain homeostasis. Additionally, beyond this mechanical barrier, considerable evidence has been generated regarding critical innate immune functions of the epithelial cells in production of protective factors and communication activities with resident and emigrating inflammatory and immune cells in health and disease. However, how these cells or tissues function at the molecular level across their portfolio of biological activities during the transition from health to inflammation and the formation of a periodontal lesion is not clear. Studies in human disease generally compare the features of clinically healthy gingival tissues with samples from sites with measurable clinical features of bleeding, pocketing, and attachment loss. However, a critical assessment of the characteristics and kinetics of the changes that occur with disease initiation and progression is limited in the human model. This occurs since the clinical features that can be measured are not necessarily coincident with the biological changes that fundamentally underpin dysbiotic microbiome changes and dysregulated host responses [21]. Cell biology studies with an array of cells that comprise the gingival tissues, including oral epithelial cells, have demonstrated changes in gene expression and/or product formation in response to oral bacteria [36–40]. However, while these approaches provide some insights into various cell capabilities, they can neither reflect the complexity of the tissue nor recapitulate the dynamics of the cellular infiltrate and maturation during the disease process. Furthermore, various approaches have utilized murine models of oral inflammation and alveolar bone loss combined with genetic manipulation to confirm a likely role for some biologic factors in the disease process [41–45]. Some more recent studies have also documented that the disease occurrence also reflects a dysbiosis of the murine oral microbiome, albeit there is little similarity in the microbiome components between humans and mice [43, 46]. Thus, we have implemented studies using a non-human primate model of periodontitis that demonstrates clinical, microbiological, and immunological features of human disease [47–50]. Moreover, traits of naturally occurring disease in the non-human primates reflect findings in humans that are related to age and sex effects [51, 52].

This study examined the biology of epithelial cell related gene expression in gingival tissues from non-human primates with naturally occurring and experimental ligature-induced disease. We also detailed the impact of age on the features of these responses and related those to the clinical disease features in the animals. Specifically, we quantified the expression of 452 genes that are related to epithelial cell biological functions. These included those reflecting extracellular matrix structure, ECM remodeling, cellular adhesion, cytoskeleton regulation, junction associated, growth factors, cell surface receptors, kinases, and response markers of inflammation and antimicrobial peptides.

Across the functional categories of the epithelial related genes were substantial similarities in the expression of these individual genes irrespective of the age of the animals. Generally, ECM structural genes and junction-associated genes were decreased in disease, and these changes were noted with initiation of the disease lesion. In contrast, genes representing cytoskeleton regulation, cell adhesion, cell surface receptors, and ECM remodeling were all increased with the disease process. Growth factor genes appeared altered in expression related to age of the animals, even in samples from healthy sites. Related to intracellular biological activities, the array of kinases was generally decreased in disease, while transcription factors appeared to be predominantly increased in the disease tissues. The summation of these gene expression differences provides a snapshot of the biology of gingival tissues that are particularly related to altered functions of the epithelium and epithelial cells, which clearly occur in the disease lesions, but are seemingly less affected by age.

Various reports have attempted to examine transcriptomes in complex tissue environments and in cell cultures to identify “hub genes” for epithelial cells functions. The hub genes are described as genes with high connectivity in tissues and diseases. Reports using Weighted Gene Co-expression Network Analysis (WGCNA) [53, 54] detected co-expression of highly correlated genes in pathway modules associated with clinical traits [55]. Some of these included BMP4, FOS, FN1, EGFR, and SPP1 related to corticosteroid resistance in epithelial cells [56]. A study by Li et al. [57] identified 12 hub genes comprised of CTSS, NOTCH4, IL8, CREB1, TCF3, SERPINA1, PTGER3, RGS4, OPRM1, MPP6, FGFR1, and NSUN3. An additional report described the top 10 hub genes FOS, CREB1, JAK2, ATF3, ATM, FYN, CREM, VEGFA, RAF1, and NCOA3 that included transcription factors and response elements for various cellular functions in oral epithelial cells infected with *Fusobacterium nucleatum* [58]. Of interest, virtually all of these were represented in our dataset in the gingival tissues, and many were identified as altered with age and particularly related to changes occurring with the disease process.

Another important question in periodontal biology is related to providing a clearer understanding of the temporal nature of changes in responses reflecting a breakdown in the integrity of the tissues that would be a harbinger of clinical disease outcomes. We have reported on the kinetics of the biology of the lesion using this non-human primate model [21]. Specifically, we identified patterns within the overall transcriptome that hallmarked health, initiation, progression, and resolution of the periodontal lesion. In this reported portfolio, some of these epithelial related genes were clearly indicative of the dynamics of the disease process. Within the context of this more detailed evaluation of epithelial functions, it was clear that many of these changes were occurring as early as 2 weeks from lesion initiation. Additionally, while the changes in multiple functional categories continued throughout disease progression, a number of the gene expression differences were primarily detected only at initiation, while other patterns demonstrated a disruption of epithelial functions that predominated later in disease progression. There now exists a potential to evaluate these differences in the non-human primates with similar transcriptomic results from cross-sectional human specimens [59–61]. It would be expected that with great similarities in the microbiome characteristics between the nonhuman primate and humans, and associated tropisms for these bacteria to cell surface receptors that are generally conserved between these mammalian species, that the comparisons would shed light on molecular disease mechanisms in humans.

Finally, this disease model enables us to explore the patterns of responses in gingival tissues where the periodontal lesion has been clinically resolved. Thus, we addressed the question of whether the epithelial cell biology of the resolved sites reflected the transcriptome patterns of healthy non-diseased gingival tissues. Across all age groups, there were numerous differences in the epithelial genes in the resolution samples, almost exclusively with decreased expression compared to baseline healthy samples. In this regard, the expression of these genes most often paralleled the expression in diseased tissues and was most prevalent in ECM structural, ECM remodeling, and cell adhesion genes. These results are consistent with clinical findings that suggest that the greatest risk for a periodontal lesion is in tooth sites with a previous episode of destructive disease [13, 15]. This biological reflection suggests that the characteristic of the epithelium/epithelial cells may not return to healthy patterns even with apparent clinical resolution, and thus poses an enhanced risk for further disease exacerbation.

The overall results of this investigation suggest a substantial alteration in epithelial cell functions that occurs rapidly with disease initiation. Many of these changes are prolonged throughout disease progression and generally reflect a disruption of normal cellular functions that would presage the resulting tissue destruction and clinical disease measures. Additionally, the overall profile of gene expression of epithelium-related genes differed in younger and older animal samples. There is an elevated prevalence and severity of periodontitis with aging suggesting a potential role for epithelial cell biology with increased age. Finally, clinical resolution may not signify biological resolution and continued risk for disease that may require considerations for additional biologic specific interventions. Future transcriptomic studies evaluating specifically dissected gingival epithelial tissue would be needed to confirm some of the main findings described in this report.

## Data Availability Statement

The datasets presented in this study can be found in online repositories. The names of the repository/repositories and accession number(s) can be found at: https://www.ebi.ac.uk/metagenomics/, E-MTAB-1977; https://www.ncbi.nlm.nih.gov/geo/, GSE180588.

## Ethics Statement

The animal study was reviewed and approved by a protocol that was approved by the Institutional Animal Care and Use Committee (IACUC) of the University of Puerto Rico.

## Author Contributions

OG and JE were responsible for the design, conduct of the experiment, clinical data and sample collection, interpretation of the data, and preparation of the manuscript. SK was responsible for the preparation of the tissue specimens for analysis and the microbiome samples. LN provided advanced statistical analysis and contribution to the methods. LO, MN, and JG-M all provided support for the implementation of the non-human primate disease model, data collection, and review of the manuscript. All authors contributed to the article and approved the submitted version.

## Funding

This work was supported by National Institute of Health grant P20GM103538. We express our gratitude to the Caribbean Primate Research Center (CPRC) supported by grant P40RR03640, specifically by Dr. Armando Burgos and the Center for Oral Health Research in the College of Dentistry at the University of Kentucky.

## Conflict of Interest

The authors declare that the research was conducted in the absence of any commercial or financial relationships that could be construed as a potential conflict of interest.

## Publisher's Note

All claims expressed in this article are solely those of the authors and do not necessarily represent those of their affiliated organizations, or those of the publisher, the editors and the reviewers. Any product that may be evaluated in this article, or claim that may be made by its manufacturer, is not guaranteed or endorsed by the publisher.

## Supplementary Material

Supplementary Figure 1 (A–G) Normalized gene expression levels in gingival tissues reflecting epithelium/epithelial cell functions. The points represent the mean normalized signal level for each age group of healthy [Adult (ADU), Aged (AG)] or periodontitis [Adult-periodontitis (ADU-PD), Aged-periodontitis (AG-PD)] animals. The genes are stratified into general functional categories (ECM Structural; ECM remodeling; Cytoskeleton Regulation; Junction Associated; Cell Adhesion; Growth Factors; Cell Surface Receptors; Kinases; Transcription Factors; Antimicrobial Peptides; Inflammatory Responses) and grouped in the graphs in alphabetical order.

Supplementary Figure 2 Z-score normalization and heatmap of the array of epithelial gene expression patterns in individual animals in healthy or periodontitis tissues.

Supplementary Figure 3 (A–R) Normalized gene expression levels in gingival tissues reflecting epithelium/epithelial cell functions. The points represent the mean normalized signal level for each age group of healthy (ADU, AG) or periodontitis (ADU-PD, AG-PD) animals. The genes are stratified into general functional categories and grouped as in Supplementary Figure 1.

Supplementary Table 1 Panther pathway analysis of altered gene expression patterns that were over-represented within the epithelial cell biology using *M. mulatta* as the reference genome.
