# Supplementary material for: Comparative Analysis of Gene Expression Patterns for Oral Epithelial Cell Functions in Periodontitis
Source: Front Oral Health. 2022 May 23;3:863231. doi: 10.3389/froh.2022.863231 (PMC9169451; doi:10.3389/froh.2022.863231)
Supplement: Supplementary Figure 1 — (A–G) Normalized gene expression levels in gingival tissues reflecting epithelium/epithelial cell functions. The points represent the mean normalized signal level for each age group of healthy [Adult (ADU), Aged (AG)] or periodontitis [Adult-periodontitis (ADU-PD), Aged-periodontitis (AG-PD)] animals. The genes are stratified into general functional categories (ECM Structural; ECM remodeling; Cytoskeleton Regulation; Junction Associated; Cell Adhesion; Growth Factors; Cell Surface Receptors; Kinases; Transcription Factors; Antimicrobial Peptides; Inflammatory Responses) and grouped in the graphs in alphabetical order. [file Presentation_1.pptx]

## Slide 1
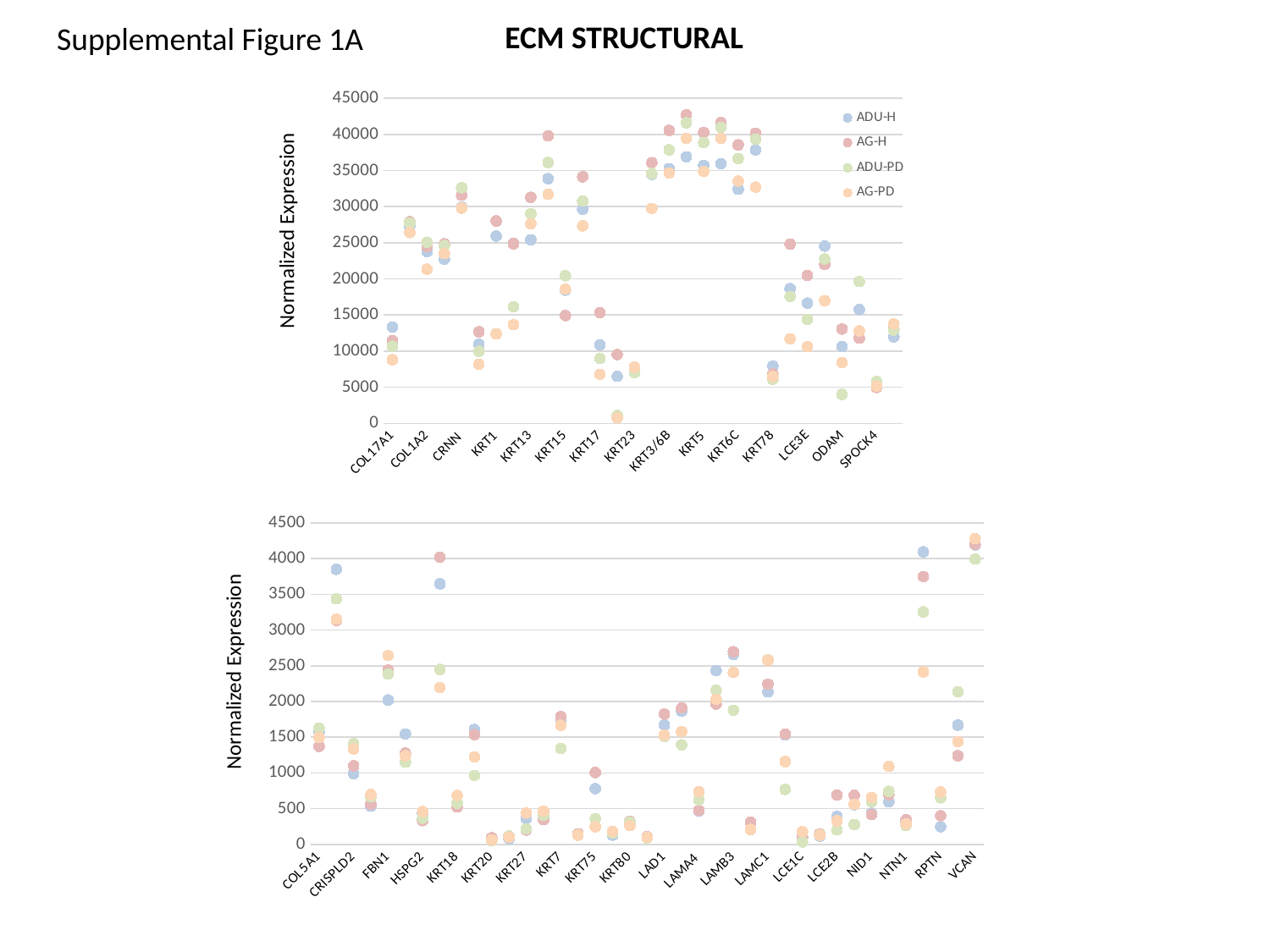

ECM STRUCTURAL
Supplemental Figure 1A
### Chart
| Category | ADU-H | AG-H | ADU-PD | AG-PD |
|---|---|---|---|---|
| COL17A1 | 13322.874285714284 | 11468.487166666666 | 10662.9984 | 8807.726666666667 |
| COL1A1 | 27300.504285714287 | 27929.454999999998 | 27760.122000000003 | 26415.826666666664 |
| COL1A2 | 23785.90142857143 | 24435.245 | 25075.019999999997 | 21345.665 |
| COL3A1 | 22741.382857142857 | 24849.32666666667 | 24610.088 | 23497.285000000003 |
| CRNN | 29907.79285714286 | 31576.671666666665 | 32617.348000000005 | 29804.076666666664 |
| DMKN | 10928.399 | 12682.131 | 9987.967 | 8177.222833333333 |
| KRT1 | 25923.50285714286 | 28019.707500000004 | 12399.26742 | 12381.718316666667 |
| KRT10 | 24945.397142857142 | 24863.920000000002 | 16121.124000000002 | 13654.587666666668 |
| KRT13 | 25422.532857142858 | 31290.721666666665 | 29019.584000000003 | 27648.925 |
| KRT14 | 33879.23714285715 | 39787.565 | 36112.978 | 31706.043333333335 |
| KRT15 | 18444.302857142855 | 14915.8655 | 20445.742 | 18586.451666666668 |
| KRT16 | 29639.965714285714 | 34128.325000000004 | 30770.026 | 27344.833333333332 |
| KRT17 | 10861.319428571427 | 15316.844666666666 | 8970.7438 | 6790.637166666666 |
| KRT2 | 6517.868157142858 | 9516.242683333334 | 1072.7648080000001 | 770.9696983333333 |
| KRT23 | 7098.292 | 7485.953833333334 | 7033.5152 | 7758.334166666667 |
| KRT24 | 34488.94857142857 | 36085.041666666664 | 34610.116 | 29754.153333333332 |
| KRT3/6B | 35261.28 | 40565.17333333333 | 37863.886 | 34679.06833333333 |
| KRT4 | 36897.86428571429 | 42720.125 | 41604.992 | 39460.14666666666 |
| KRT5 | 35697.92571428572 | 40278.871666666666 | 38876.302 | 34882.551666666666 |
| KRT6A | 35924.357142857145 | 41623.22333333333 | 40990.654 | 39463.095 |
| KRT6C | 32414.318571428576 | 38541.71166666666 | 36654.19 | 33528.29 |
| KRT76 | 37850.08285714285 | 40184.89666666666 | 39351.476 | 32701.368333333332 |
| KRT78 | 7926.178571428571 | 6843.601833333333 | 6112.989799999999 | 6490.542833333333 |
| LCE3D | 18644.777142857143 | 24816.161666666667 | 17558.566 | 11695.324999999999 |
| LCE3E | 16637.95 | 20479.163333333334 | 14383.073199999999 | 10603.507166666666 |
| LOR | 24539.671428571426 | 22015.346 | 22756.938199999997 | 16987.222833333333 |
| ODAM | 10654.509285714286 | 13062.054666666669 | 4016.093 | 8403.553933333333 |
| RPTN | 15751.379428571428 | 11784.517 | 19640.47 | 12787.691166666666 |
| SPOCK4 | 5197.726428571428 | 4965.491000000001 | 5782.393399999999 | 5164.436000000001 |
| VIM | 11979.862714285715 | 13103.733333333332 | 12899.998 | 13762.231666666667 |Normalized Expression
### Chart
| Category | ADU-H | AG-H | ADU-PD | AG-PD |
|---|---|---|---|---|
| COL5A1 | 1575.234 | 1370.7623833333334 | 1626.7132000000001 | 1494.8845000000001 |
| COL7A1 | 3851.384142857143 | 3137.2016666666664 | 3437.2582 | 3153.1756666666665 |
| CRISPLD2 | 988.8095 | 1098.1256333333333 | 1410.1943800000001 | 1336.7049166666666 |
| FBLN5 | 532.8113285714286 | 560.44275 | 665.45574 | 697.5672500000001 |
| FBN1 | 2021.029142857143 | 2440.7478333333333 | 2383.7948 | 2643.921966666667 |
| FLG2 | 1545.3908714285712 | 1278.6085 | 1149.7835 | 1240.51151 |
| HSPG2 | 429.77439999999996 | 331.1290666666667 | 364.94698 | 457.13121666666666 |
| IFFO2 | 3648.437714285715 | 4019.8965000000003 | 2449.7081 | 2197.6419499999997 |
| KRT18 | 521.8272285714286 | 522.15495 | 576.3055 | 683.6693 |
| KRT19 | 1607.7063 | 1536.3653166666666 | 965.00714 | 1225.1411333333333 |
| KRT20 | 65.69268142857143 | 91.45517166666666 | 51.064918 | 61.187624833333324 |
| KRT25 | 78.12673714285714 | 116.86384 | 113.06330399999999 | 99.47420666666669 |
| KRT27 | 359.18471 | 200.15448333333327 | 217.86339600000002 | 436.5427383333333 |
| KRT38 | 364.2494428571428 | 344.2836 | 406.013818 | 463.10562333333337 |
| KRT7 | 1720.522742857143 | 1788.4143833333335 | 1339.77968 | 1666.9285166666666 |
| KRT74 | 142.77248571428572 | 147.49695 | 126.84566 | 128.18981666666664 |
| KRT75 | 778.7699142857144 | 1005.4778833333335 | 358.675 | 245.0210833333333 |
| KRT8 | 127.7509857142857 | 162.5944 | 154.590534 | 181.50535333333335 |
| KRT80 | 270.6772714285715 | 321.6143666666666 | 307.33158000000003 | 261.17111666666665 |
| KRT9 | 105.37188285714285 | 107.86588333333334 | 88.229746 | 97.64844333333333 |
| LAD1 | 1669.9257142857143 | 1823.679833333333 | 1510.1066 | 1530.889 |
| LAMA3 | 1865.7430000000002 | 1908.5529999999999 | 1392.4076 | 1579.1226833333333 |
| LAMA4 | 464.3592 | 473.5934833333333 | 624.0401599999999 | 737.90515 |
| LAMA5 | 2433.9121428571425 | 1963.8688333333332 | 2157.4676 | 2030.7103333333337 |
| LAMB3 | 2657.188857142857 | 2696.222 | 1877.1072 | 2407.7676666666666 |
| LAMB4 | 302.6492285714285 | 309.3421083333334 | 203.99492 | 207.66955 |
| LAMC1 | 2134.9525714285714 | 2241.768333333333 | 2587.3906 | 2574.5068333333334 |
| LAMC2 | 1528.9046285714287 | 1541.8778666666667 | 769.4665600000001 | 1158.3972166666667 |
| LCE1C | 102.08731 | 96.14724999999999 | 33.616352 | 175.612073 |
| LCE2A | 113.11045 | 147.10815 | 133.53896 | 127.04583833333334 |
| LCE2B | 389.0929714285714 | 689.2800033333333 | 201.221792 | 329.1479516666667 |
| LCE2C | 554.1701571428572 | 685.4348283333334 | 275.922344 | 559.4221133333333 |
| NID1 | 433.6572714285714 | 415.78388333333334 | 594.42084 | 655.98955 |
| NID2 | 595.1407428571428 | 697.3317499999999 | 740.76918 | 1090.1726666666666 |
| NTN1 | 320.6988571428572 | 343.3093166666667 | 262.04158 | 289.3947333333333 |
| POF1B | 4096.119142857143 | 3750.6445 | 3253.688 | 2414.4443333333334 |
| RPTN | 246.14042999999995 | 399.51955 | 651.0204 | 733.27405 |
| SLURP1 | 1668.2637571428572 | 1240.8567166666667 | 2137.54004 | 1439.2410666666665 |
| VCAN | 4193.280857142857 | 4200.094166666667 | 3994.4854 | 4281.123833333333 |Normalized Expression

## Slide 2
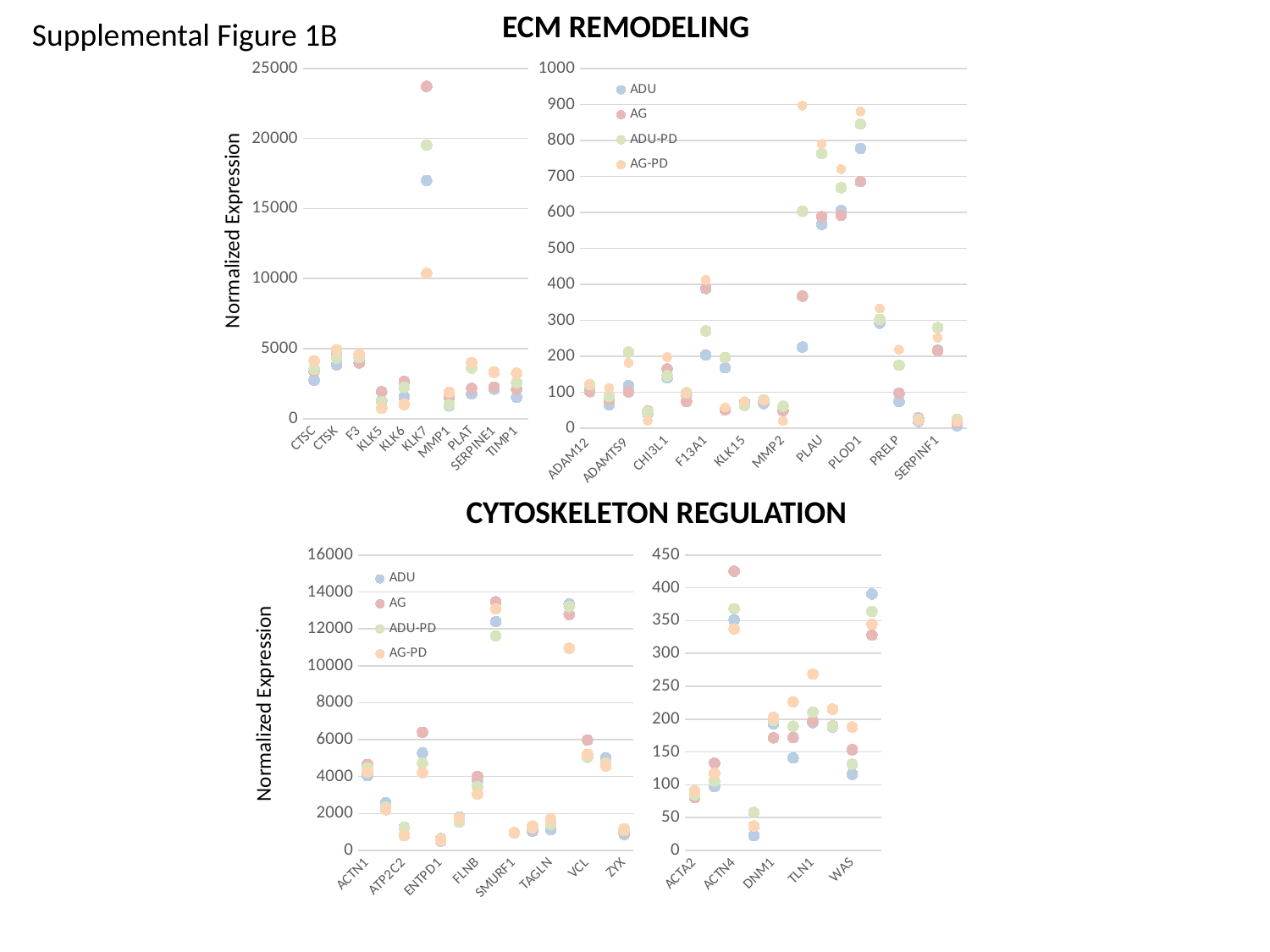

ECM REMODELING
Supplemental Figure 1B
### Chart
| Category | ADU | AG | ADU-PD | AG-PD |
|---|---|---|---|---|
| CTSC | 2741.602428571429 | 3387.3423333333335 | 3521.6166 | 4124.994333333333 |
| CTSK | 3849.248857142857 | 4643.075000000001 | 4328.6394 | 4914.340499999999 |
| F3 | 4348.309714285714 | 3973.6438333333335 | 4363.748600000001 | 4594.026000000001 |
| KLK5 | 1273.3198 | 1922.519716666667 | 1203.6037199999998 | 741.7195833333334 |
| KLK6 | 1556.529385714286 | 2661.2070833333332 | 2230.92486 | 1012.9286666666667 |
| KLK7 | 16999.155714285713 | 23719.574333333334 | 19528.620000000003 | 10374.2835 |
| MMP1 | 904.8009428571429 | 1504.5916 | 996.2034800000001 | 1897.8267166666665 |
| PLAT | 1771.6151142857143 | 2159.3049833333334 | 3604.0366000000004 | 4005.1358333333333 |
| SERPINE1 | 2104.7652857142857 | 2262.5056666666665 | 3336.1943999999994 | 3310.3016666666667 |
| TIMP1 | 1524.9085714285716 | 2098.229333333333 | 2575.1910000000003 | 3242.8933333333334 |
### Chart
| Category | ADU | AG | ADU-PD | AG-PD |
|---|---|---|---|---|
| ADAM12 | 110.13978714285713 | 102.06045333333334 | 121.38376600000001 | 123.58912500000001 |
| ADAMTS6 | 65.31369428571428 | 79.274735 | 89.730492 | 111.94406166666668 |
| ADAMTS9 | 118.77534285714285 | 101.37414833333332 | 211.9816 | 181.68695 |
| ADAMTSL1 | 41.645652857142856 | 47.988634999999995 | 45.96071400000001 | 21.59406316666666 |
| CHI3L1 | 140.51007142857142 | 165.25160000000002 | 146.134934 | 197.99435000000003 |
| ELA2 | 93.16200285714287 | 74.57524666666667 | 98.89837399999999 | 95.16144166666668 |
| F13A1 | 203.63887142857146 | 388.64948333333336 | 270.57394000000005 | 412.4757666666667 |
| KLK14 | 168.08958857142855 | 51.51596833333334 | 196.33966800000002 | 57.87328500000001 |
| KLK15 | 72.1420242857143 | 68.38193333333332 | 63.892323999999995 | 75.37423666666666 |
| LOX | 68.93055857142856 | 76.74068 | 79.16953 | 76.42836166666667 |
| MMP2 | 60.07254714285714 | 49.11372883333334 | 61.34972799999999 | 20.739089999999997 |
| MMP9 | 226.01285714285717 | 367.3998166666667 | 603.4347799999999 | 897.1409 |
| PLAU | 567.3609285714285 | 588.4340166666667 | 763.61462 | 790.9877333333334 |
| PLAUR | 605.1075999999999 | 592.2601166666667 | 669.02268 | 720.7360166666666 |
| PLOD1 | 778.3446714285712 | 686.0724333333334 | 845.98184 | 880.8616499999999 |
| PLOD2 | 292.2353428571429 | 302.85735 | 302.98246 | 333.09133333333335 |
| PRELP | 74.35988571428571 | 97.697785 | 175.584488 | 218.70988166666666 |
| PTK2 | 19.40673185714286 | 28.796226666666666 | 24.824715 | 20.792342666666666 |
| SERPINF1 | 217.17201428571428 | 215.48946666666666 | 280.00058 | 251.95095 |
| TIMP4 | 6.813383857142858 | 16.9768535 | 24.997947999999997 | 17.77195416666667 |Normalized Expression
CYTOSKELETON REGULATION
### Chart
| Category | ADU | AG | ADU-PD | AG-PD |
|---|---|---|---|---|
| ACTN1 | 4050.5817142857145 | 4654.836833333333 | 4471.066 | 4246.1123333333335 |
| ATP2C1 | 2587.6127142857144 | 2208.611666666666 | 2350.7828 | 2212.968833333333 |
| ATP2C2 | 1237.1297285714286 | 1247.0088500000002 | 1231.1483199999998 | 796.9898 |
| CALML5 | 5285.311428571427 | 6400.865666666666 | 4735.2916 | 4208.388 |
| ENTPD1 | 502.8822571428571 | 630.6796666666665 | 607.3771399999999 | 549.0858333333334 |
| FLNA | 1810.6802857142852 | 1703.4824999999998 | 1528.9828 | 1760.0088333333333 |
| FLNB | 3772.0864285714283 | 4004.5733333333337 | 3446.3086000000003 | 3047.6510000000003 |
| RAC1 | 12385.358571428571 | 13466.49 | 11615.243799999998 | 13074.25 |
| SMURF1 | 948.3725857142857 | 945.0410333333334 | 942.9039399999999 | 968.8483166666668 |
| STX5 | 1031.6903 | 1132.9698999999998 | 1231.0888 | 1319.2636666666667 |
| TAGLN | 1119.693657142857 | 1532.4151666666667 | 1412.5902 | 1694.4886666666669 |
| TIAM1 | 13346.66857142857 | 12776.245 | 13205.653999999999 | 10950.78016666667 |
| VCL | 5082.131571428572 | 5977.286166666668 | 5043.631800000001 | 5208.6195 |
| WASL | 5018.078285714286 | 4779.033166666666 | 4741.6604 | 4579.5015 |
| ZYX | 865.7302857142856 | 1002.0292833333333 | 1080.0569799999998 | 1170.5183166666668 |
### Chart
| Category | ADU | AG | ADU-PD | AG-PD |
|---|---|---|---|---|
| ACTA2 | 82.79780857142858 | 80.28139166666666 | 84.7546 | 90.86029333333335 |
| ACTN3 | 97.28863714285716 | 132.92636 | 105.481106 | 117.11701833333335 |
| ACTN4 | 351.09052857142854 | 425.22436666666664 | 368.18272 | 337.37115 |
| CCDC19 | 22.401819999999997 | 36.40717466666666 | 57.790178000000004 | 37.23772483333334 |
| DNM1 | 192.65962857142858 | 171.52542666666668 | 197.88228 | 202.5961666666667 |
| PDGFRB | 140.88897142857144 | 171.95136333333335 | 189.145536 | 226.3565833333333 |
| TLN1 | 194.76207142857146 | 196.91715 | 210.45326 | 268.74685 |
| TLN2 | 187.88331428571428 | 189.50083333333336 | 189.25276000000002 | 215.21288333333334 |
| WAS | 115.85606142857145 | 153.27328666666668 | 131.227304 | 187.96142666666665 |
| WASF1 | 390.5694 | 327.70390000000003 | 363.93812 | 344.4717166666667 |Normalized Expression

## Slide 3
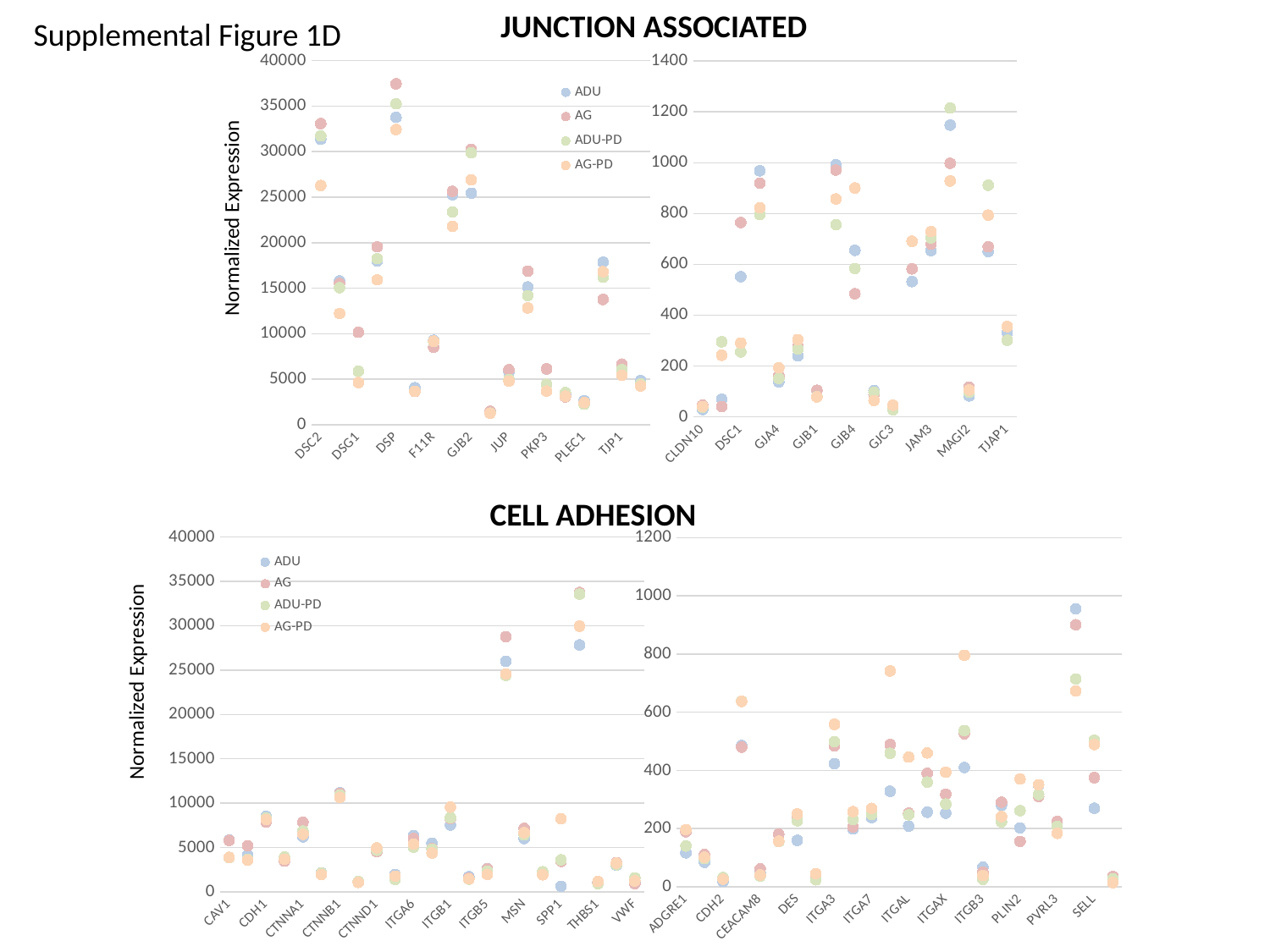

JUNCTION ASSOCIATED
Supplemental Figure 1D
### Chart
| Category | ADU | AG | ADU-PD | AG-PD |
|---|---|---|---|---|
| DSC2 | 31359.477142857137 | 33069.35333333333 | 31718.056 | 26280.02 |
| DSC3 | 15785.732857142855 | 15512.13 | 15045.876 | 12219.059333333333 |
| DSG1 | 10154.252857142857 | 10150.732333333333 | 5867.338400000001 | 4640.743666666666 |
| DSG3 | 17971.832857142857 | 19534.631666666668 | 18234.96 | 15910.271666666666 |
| DSP | 33762.39714285714 | 37435.19666666666 | 35242.39200000001 | 32416.050000000003 |
| EVPL | 4064.5330000000004 | 3644.9166666666665 | 3678.7194000000004 | 3667.787 |
| F11R | 9273.319428571429 | 8505.205666666667 | 9135.5196 | 9177.928833333333 |
| GJA1 | 25265.83714285714 | 25631.71666666667 | 23361.557999999997 | 21783.32666666667 |
| GJB2 | 25430.94857142857 | 30245.213333333333 | 29858.833999999995 | 26888.671666666665 |
| GJB5 | 1354.5357142857142 | 1470.787 | 1277.1172 | 1258.2300333333333 |
| JUP | 5805.885285714286 | 6037.163833333333 | 4961.6136 | 4787.739166666666 |
| PKP1 | 15126.564285714285 | 16870.786666666663 | 14180.506 | 12820.03 |
| PKP3 | 6126.564 | 6136.7446666666665 | 4435.7991999999995 | 3690.185166666666 |
| PKP4 | 3375.9438571428577 | 3056.8233333333333 | 3531.5114000000003 | 3140.907833333333 |
| PLEC1 | 2635.203142857143 | 2399.5136666666667 | 2259.6178 | 2434.9768333333336 |
| PPL | 17853.57 | 13753.465166666667 | 16196.338 | 16810.773333333334 |
| TJP1 | 6263.258714285715 | 6661.567666666669 | 6052.8758 | 5434.264166666667 |
| TJP2 | 4831.027 | 4444.151666666666 | 4488.4166000000005 | 4260.787666666667 |
### Chart
| Category | ADU | AG | ADU-PD | AG-PD |
|---|---|---|---|---|
| CLDN10 | 28.585440428571427 | 45.935333333333325 | 37.805886 | 41.02615333333333 |
| CLDN8 | 69.30623571428572 | 40.825469999999996 | 295.38144 | 242.45589500000003 |
| DSC1 | 551.3567142857142 | 764.5555666666668 | 255.38888000000003 | 290.51003333333335 |
| DSG2 | 968.1600999999999 | 919.4168333333333 | 795.85528 | 822.5044666666668 |
| GJA4 | 137.0823142857143 | 162.27606666666665 | 151.12624 | 192.8606166666667 |
| GJA5 | 240.00012857142858 | 276.8265833333333 | 266.70732 | 304.12128333333334 |
| GJB1 | 80.76727285714286 | 104.59148833333334 | 79.50236199999999 | 78.47040666666668 |
| GJB3 | 991.7920571428573 | 971.18395 | 756.4315 | 857.4626666666667 |
| GJB4 | 655.2815857142857 | 484.31659999999994 | 583.3562800000001 | 900.4249833333333 |
| GJC2 | 103.46949000000002 | 87.06973666666669 | 97.14014 | 64.30813500000001 |
| GJC3 | 40.673595 | 38.21794833333333 | 27.736971999999998 | 45.433054999999996 |
| JAM2 | 532.0604 | 582.0518166666667 | 690.70378 | 691.1639666666666 |
| JAM3 | 654.2104714285714 | 679.8642333333333 | 704.6732599999999 | 729.1037500000001 |
| MAGI1 | 1147.9230142857143 | 997.5899499999999 | 1215.1221999999998 | 927.8779000000001 |
| MAGI2 | 82.43231285714286 | 117.10106999999998 | 96.91241800000002 | 105.81938166666667 |
| PKP2 | 650.6682285714286 | 669.3148166666667 | 911.44838 | 793.7698999999999 |
| TJAP1 | 331.49359999999996 | 301.70498333333336 | 301.55690000000004 | 355.0783666666666 |Normalized Expression
CELL ADHESION
### Chart
| Category | ADU | AG | ADU-PD | AG-PD |
|---|---|---|---|---|
| CAV1 | 5835.934428571428 | 5797.265666666666 | 3845.5208 | 3891.540333333334 |
| CAV2 | 4148.081142857142 | 5197.0498333333335 | 3662.3388 | 3575.012 |
| CDH1 | 8512.122000000001 | 7867.033833333334 | 8333.117999999999 | 8158.707666666666 |
| CDH3 | 3464.6147142857144 | 3472.581166666667 | 3934.1416 | 3675.6095 |
| CTNNA1 | 6195.950142857143 | 7836.315 | 6849.299000000001 | 6479.917166666666 |
| CTNNAL1 | 2162.182142857143 | 2076.0933333333332 | 2100.5572 | 1953.9223333333332 |
| CTNNB1 | 11168.345714285713 | 11102.091666666667 | 10903.036 | 10655.968333333332 |
| CTNNBIP1 | 1067.1465571428573 | 1073.3553666666667 | 1176.3141 | 1060.1456333333333 |
| CTNND1 | 4715.096 | 4527.3805 | 4685.488800000001 | 4950.060333333334 |
| ITGA2 | 1958.8155714285713 | 1763.548566666667 | 1391.6694 | 1779.9496666666666 |
| ITGA6 | 6344.056857142858 | 6029.309333333334 | 5040.7996 | 5384.623166666667 |
| ITGAV | 5484.544857142857 | 4892.3715 | 4816.3514 | 4381.143166666666 |
| ITGB1 | 7538.778714285714 | 8256.1885 | 8358.9252 | 9558.034833333333 |
| ITGB4 | 1709.0534285714286 | 1522.2434999999998 | 1421.9666 | 1452.9646666666667 |
| ITGB5 | 2618.806 | 2586.2425 | 2321.4737999999998 | 1971.9291666666668 |
| LGALS3 | 25983.477142857148 | 28765.786666666667 | 24420.622000000003 | 24564.961666666666 |
| MSN | 6005.759571428572 | 7154.378333333333 | 6420.6485999999995 | 6674.2015 |
| PVRL1 | 2243.0767142857144 | 2138.9026666666664 | 2261.7828 | 1924.2381666666668 |
| SPP1 | 634.1098857142857 | 3438.5511 | 3603.9184 | 8244.392849999998 |
| SPRR2D | 27831.3 | 33753.01833333333 | 33563.780000000006 | 29946.493333333332 |
| THBS1 | 1068.1058142857144 | 1018.4153999999999 | 927.47968 | 1167.2535500000001 |
| THBS2 | 2999.4032857142856 | 3293.872 | 3063.8329999999996 | 3243.3995 |
| VWF | 1020.9364714285714 | 925.744 | 1578.0467999999998 | 1227.3793833333332 |
### Chart
| Category | BL | 2 | 1 | 3 |
|---|---|---|---|---|
| ADGRE1 | 116.84160285714286 | 189.12204999999997 | 140.38150000000002 | 196.51528333333331 |
| CAV3 | 84.20186571428573 | 110.91185 | 96.921258 | 101.97933666666665 |
| CDH2 | 18.36057142857143 | 29.757761666666667 | 32.2451634 | 26.304513833333335 |
| CDH5 | 485.8210571428571 | 480.1578666666667 | 638.1674 | 637.1713666666667 |
| CEACAM8 | 48.62894428571428 | 61.60855166666666 | 36.863932 | 40.95011833333333 |
| CTNNBL1 | 158.38000000000002 | 181.36926666666668 | 155.33204 | 156.82342833333334 |
| DES | 159.80528142857142 | 237.99578333333332 | 227.14584 | 250.31213499999998 |
| FN1 | 30.842437142857143 | 37.543540666666665 | 24.0254624 | 45.630925 |
| ITGA3 | 423.34275714285707 | 484.15796666666665 | 498.58534 | 558.25085 |
| ITGA5 | 198.54494285714284 | 206.46571666666668 | 231.06869999999998 | 258.3186166666666 |
| ITGA7 | 237.81392857142856 | 252.98403333333331 | 248.90861999999998 | 269.7410666666667 |
| ITGA8 | 328.30237142857146 | 488.58545000000004 | 458.6741 | 741.7891833333333 |
| ITGAL | 208.80089999999998 | 253.11463333333333 | 247.64272 | 445.85201666666666 |
| ITGAM | 256.44992857142853 | 389.9359166666666 | 359.88824 | 459.9510333333333 |
| ITGAX | 253.11318571428572 | 318.1645833333333 | 284.36874000000006 | 393.98181666666665 |
| ITGB2 | 410.0848571428572 | 525.9677833333333 | 536.91914 | 795.9593166666667 |
| ITGB3 | 67.34867285714286 | 50.98402816666666 | 25.900870000000005 | 39.06772833333333 |
| ITGB6 | 279.7878 | 290.72738333333336 | 223.50302000000002 | 241.40928333333338 |
| PLIN2 | 202.50202857142858 | 156.14445833333332 | 261.7679 | 370.53358333333335 |
| PVRL2 | 349.6901142857144 | 310.44651666666664 | 317.35478 | 351.0656000000001 |
| PVRL3 | 213.49987142857142 | 224.6224 | 207.60944 | 183.94831666666667 |
| PVRL4 | 955.0676000000001 | 900.6470333333332 | 714.5474999999999 | 672.9462 |
| SELL | 269.95284285714285 | 375.3283333333334 | 503.49676 | 489.19104999999996 |
| SELP | 27.60644285714286 | 35.41771666666667 | 27.984524 | 14.788580666666668 |Normalized Expression

## Slide 4
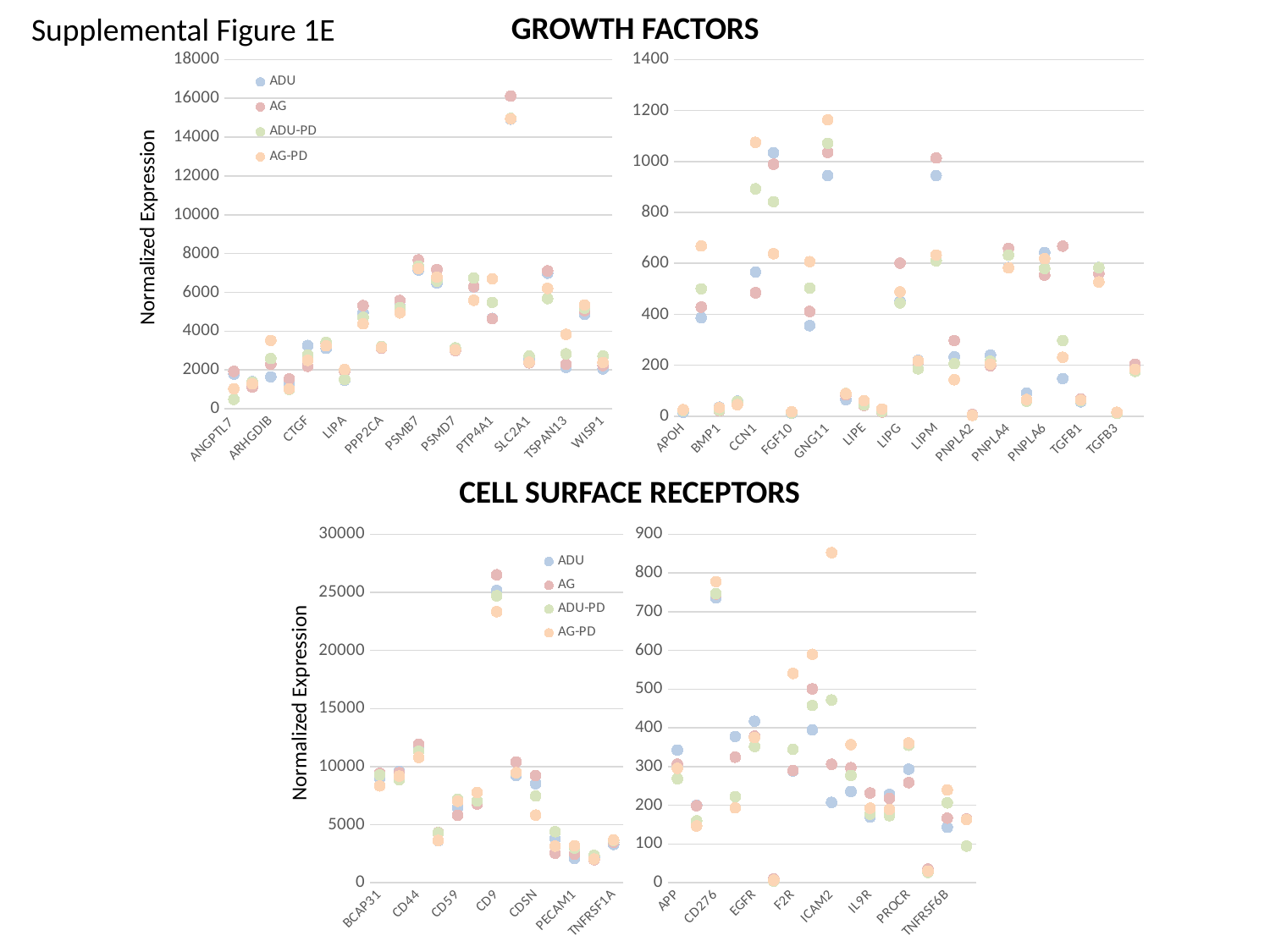

GROWTH FACTORS
Supplemental Figure 1E
### Chart
| Category | ADU | AG | ADU-PD | AG-PD |
|---|---|---|---|---|
| ANGPTL7 | 1788.0690571428572 | 1923.2592666666667 | 486.18565 | 1025.9071166666665 |
| ARG2 | 1396.5658857142857 | 1117.3675833333334 | 1377.234 | 1285.583 |
| ARHGDIB | 1645.229142857143 | 2304.428 | 2586.6166000000003 | 3519.1161666666667 |
| BMP2 | 1248.2317714285714 | 1539.0268000000003 | 1004.98814 | 1026.4207833333332 |
| CTGF | 3260.1488571428567 | 2190.021166666667 | 2765.95 | 2494.2411666666667 |
| GPI | 3112.424714285714 | 3364.5691666666667 | 3421.9026 | 3240.636 |
| LIPA | 1474.4468428571429 | 1945.8940000000002 | 1511.4717999999998 | 2020.2205000000001 |
| PNPLA8 | 4945.924857142856 | 5315.034166666666 | 4710.9612 | 4372.9525 |
| PPP2CA | 3170.9542857142856 | 3119.058333333333 | 3201.0178 | 3153.1939999999995 |
| PSMB5 | 5258.627714285714 | 5579.018333333333 | 5218.633 | 4940.551166666667 |
| PSMB7 | 7150.510428571429 | 7671.328166666666 | 7347.9928 | 7223.822499999999 |
| PSMC2 | 6484.256 | 7174.873833333334 | 6581.4524 | 6785.384166666667 |
| PSMD7 | 2997.3095714285714 | 2993.8668333333335 | 3131.4629999999997 | 3037.9893333333334 |
| PTEN | 6282.917142857143 | 6287.968000000001 | 6733.1219999999985 | 5588.808 |
| PTP4A1 | 4655.879 | 4637.142666666667 | 5483.8412 | 6693.404500000001 |
| RHOA | 14927.894285714285 | 16115.981666666667 | 14961.722 | 14946.908333333333 |
| SLC2A1 | 2563.412857142857 | 2367.215166666667 | 2710.8831999999998 | 2400.1611666666668 |
| TGFBI | 6986.206571428572 | 7099.607500000001 | 5677.64 | 6200.998833333334 |
| TSPAN13 | 2140.0244285714284 | 2288.2865 | 2829.2836 | 3835.003 |
| VEGFA | 4866.402428571428 | 5050.934499999999 | 5184.038 | 5351.965166666667 |
| WISP1 | 2055.910714285714 | 2225.8865 | 2719.0934 | 2388.2876666666666 |
### Chart
| Category | ADU | AG | ADU-PD | AG-PD |
|---|---|---|---|---|
| APOH | 15.800806285714284 | 22.755511666666663 | 22.2185508 | 25.74417866666667 |
| ARHGEF2 | 386.4212142857142 | 428.2699833333333 | 499.9678 | 668.2717666666666 |
| BMP1 | 35.43776999999999 | 20.851274999999998 | 23.6797854 | 33.717474833333334 |
| BMP7 | 59.158705714285716 | 47.60619833333333 | 58.238816 | 45.40928333333334 |
| CCN1 | 566.0780714285714 | 484.50078333333335 | 892.39418 | 1074.8092333333334 |
| ESYT3 | 1033.8175714285715 | 989.3173666666667 | 842.2049800000001 | 637.8977333333333 |
| FGF10 | 11.754908999999998 | 16.866299666666666 | 12.942324600000001 | 17.637645000000003 |
| FGF7 | 355.5180714285715 | 411.2417 | 503.22065999999995 | 606.7410383333333 |
| GNG11 | 944.5903428571428 | 1035.4340499999998 | 1071.2292400000001 | 1163.3023333333333 |
| LIPC | 65.2530957142857 | 83.81911000000001 | 89.27510199999999 | 88.68259333333333 |
| LIPE | 43.06576142857143 | 42.26170833333334 | 44.362442 | 61.10749333333333 |
| LIPF | 22.662402428571426 | 16.449454166666666 | 18.921446600000003 | 28.331998333333335 |
| LIPG | 451.3626 | 601.0463166666667 | 444.69118 | 488.0449333333333 |
| LIPH | 219.7834371428571 | 211.99656666666667 | 186.124164 | 217.77973333333333 |
| LIPM | 944.5445428571429 | 1013.4048499999999 | 609.92326 | 632.7973333333333 |
| NT5E | 232.79285714285714 | 296.99865 | 206.7819 | 143.32198 |
| PNPLA2 | 4.707738142857143 | 6.6495365 | 3.377332 | 3.7967251666666666 |
| PNPLA3 | 240.1459142857143 | 198.1249 | 216.14679999999998 | 204.51633333333336 |
| PNPLA4 | 583.1766285714286 | 658.6111500000001 | 632.57574 | 582.2725333333334 |
| PNPLA5 | 91.54378571428572 | 62.91937333333333 | 59.550670000000004 | 65.51775583333334 |
| PNPLA6 | 642.2309857142856 | 553.7398999999999 | 580.2479599999999 | 618.3732333333334 |
| PPBP | 147.81761857142857 | 667.729915 | 296.89553799999993 | 231.30429333333328 |
| TGFB1 | 56.94946142857143 | 68.04404999999998 | 61.02297599999999 | 63.68053333333334 |
| TGFB2 | 583.5024000000001 | 559.7265166666666 | 583.24914 | 526.8744333333334 |
| TGFB3 | 12.371058857142858 | 11.3679635 | 10.9109912 | 15.5831715 |
| VEGFD | 191.16885714285715 | 204.47646666666665 | 176.219362 | 184.60023333333334 |Normalized Expression
CELL SURFACE RECEPTORS
### Chart
| Category | ADU | AG | ADU-PD | AG-PD |
|---|---|---|---|---|
| BCAP31 | 8978.059714285713 | 9419.141 | 9270.069 | 8334.447000000002 |
| CD164 | 9605.237428571429 | 9475.916833333335 | 8862.7514 | 9179.131166666666 |
| CD44 | 11712.198142857143 | 11917.83 | 11322.8186 | 10794.872500000001 |
| CD46 | 3601.0817142857145 | 4332.009333333334 | 4294.1024 | 3643.384666666667 |
| CD59 | 6459.608714285714 | 5810.666166666667 | 7180.3212 | 7017.5053333333335 |
| CD81 | 6872.612714285714 | 6777.428 | 7053.6906 | 7781.755499999999 |
| CD9 | 25164.99142857143 | 26516.524999999994 | 24714.756 | 23340.486666666668 |
| CD99 | 9229.77757142857 | 10396.224 | 9460.987799999999 | 9472.956 |
| CDSN | 8527.263857142858 | 9228.437833333335 | 7461.583600000001 | 5820.104750000001 |
| FKBP5 | 3783.4331428571422 | 2540.1836666666672 | 4396.4604 | 3153.5151666666666 |
| PECAM1 | 2088.8707142857143 | 2459.975 | 2984.1542 | 3187.9245000000005 |
| THBD | 2216.8397142857143 | 1979.4693333333335 | 2348.2228 | 2039.9011666666665 |
| TNFRSF1A | 3298.883571428572 | 3491.523333333333 | 3645.9863999999993 | 3688.3921666666665 |
### Chart
| Category | ADU | AG | ADU-PD | AG-PD |
|---|---|---|---|---|
| APP | 342.55569999999994 | 306.1786166666667 | 268.64738 | 295.4198999999999 |
| CD207 | 200.0106142857143 | 198.90905 | 159.65658 | 146.52376333333333 |
| CD276 | 735.6273142857144 | 745.9318499999999 | 746.838 | 778.0062166666667 |
| CD36 | 377.7559428571429 | 324.33025000000004 | 222.42942000000002 | 193.29368333333332 |
| EGFR | 417.4490142857143 | 378.6266166666667 | 351.97334 | 374.9764333333333 |
| ESR1 | 4.342751428571429 | 9.755353833333333 | 4.181548 | 6.4118848333333345 |
| F2R | 288.6194142857143 | 289.8648333333333 | 344.66949999999997 | 540.6407833333333 |
| ICAM1 | 394.62910000000005 | 500.75756666666666 | 457.89745999999997 | 590.0939333333334 |
| ICAM2 | 207.41628571428572 | 305.8896333333333 | 471.98378 | 852.6037833333334 |
| IGFLR1 | 235.67464285714283 | 297.21745 | 277.11276 | 356.4955 |
| IL9R | 169.8966428571429 | 231.76018333333332 | 177.3038 | 192.4120583333333 |
| OCLN | 227.90444285714284 | 217.79218333333336 | 173.31289999999998 | 189.23969166666666 |
| PROCR | 293.2668428571428 | 258.69638333333336 | 354.97862 | 360.81005 |
| TMEFF1 | 32.10252857142857 | 35.15982833333334 | 26.894137999999998 | 30.241275 |
| TNFRSF6B | 143.02291285714287 | 166.88363333333334 | 206.23540000000003 | 239.62839999999997 |
| WNT5B | 163.53258000000002 | 164.41347333333334 | 94.64516400000001 | 163.13883833333335 |Normalized Expression

## Slide 5
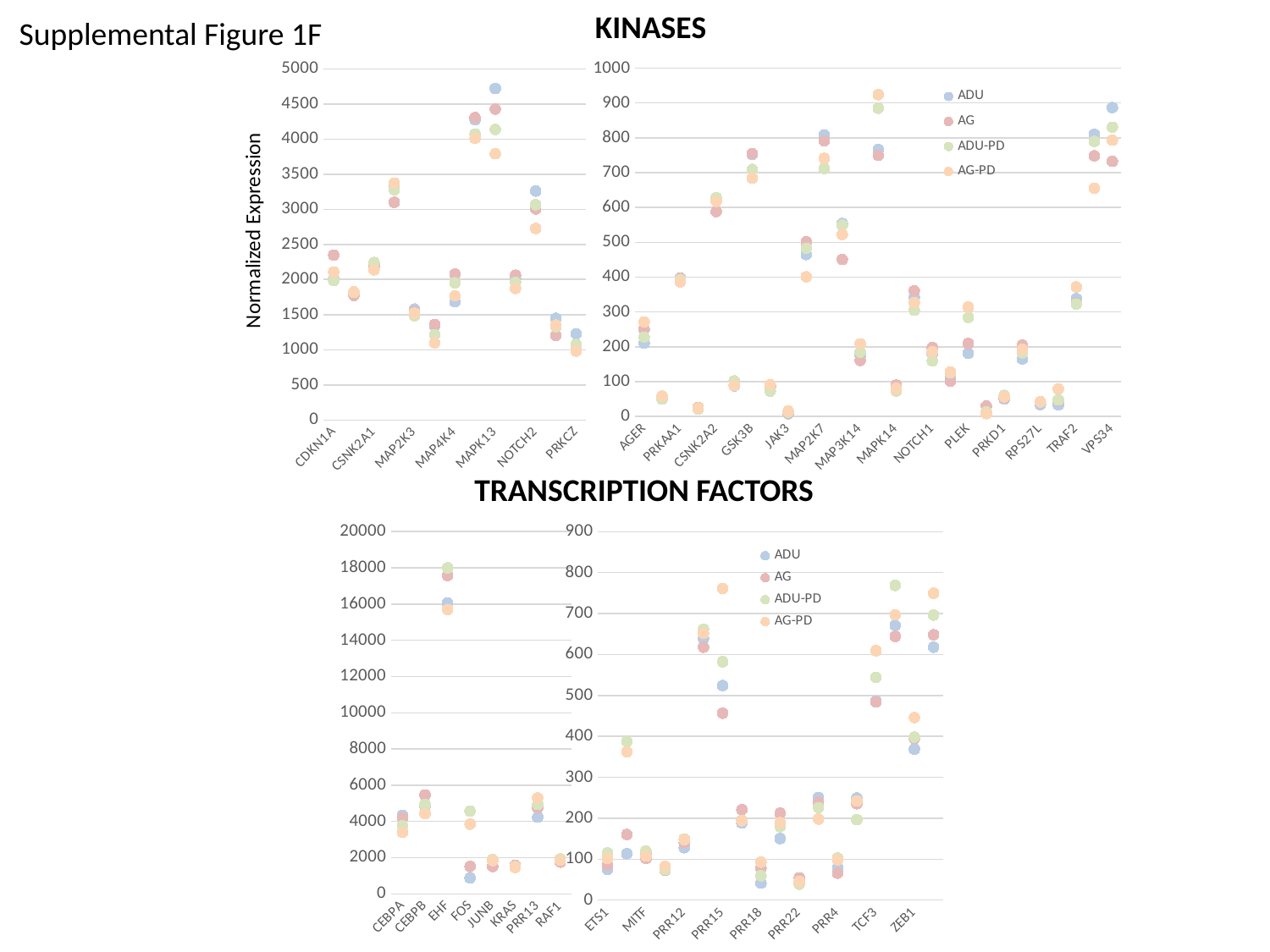

KINASES
Supplemental Figure 1F
### Chart
| Category | ADU | AG | ADU-PD | AG-PD |
|---|---|---|---|---|
| AGER | 210.39024285714282 | 250.14848333333336 | 227.71510000000004 | 271.46021666666667 |
| AKT1 | 55.96149857142858 | 57.60345166666665 | 49.493352 | 58.50583333333333 |
| PRKAA1 | 397.42900000000003 | 396.50865 | 393.18036 | 386.42356666666666 |
| PRKAA2 | 23.330126142857146 | 26.280607500000002 | 20.948128000000004 | 24.177832999999996 |
| CSNK2A2 | 627.9939142857144 | 588.0787833333334 | 628.2589399999999 | 617.44035 |
| DBF4 | 87.70115142857144 | 101.24248 | 101.974938 | 88.46140999999999 |
| GSK3B | 751.8269142857142 | 754.5474833333334 | 709.0898 | 683.7275333333333 |
| JAG1 | 82.54662857142857 | 85.83216166666666 | 72.77662000000001 | 90.84714666666667 |
| JAK3 | 8.013828571428572 | 9.879078 | 12.5553092 | 15.7167575 |
| MAP2K6 | 464.97151428571425 | 501.5993166666667 | 482.9837799999999 | 400.77628333333337 |
| MAP2K7 | 807.8602571428572 | 790.8763833333333 | 711.8443599999999 | 741.1716166666666 |
| MAP3K1 | 553.9419142857142 | 450.55665000000005 | 549.22752 | 522.5120499999999 |
| MAP3K14 | 174.85965714285717 | 160.52258333333333 | 184.5526 | 208.35198333333332 |
| MAP3K5 | 766.4524714285715 | 749.3711833333333 | 884.9316600000002 | 923.8875166666667 |
| MAPK14 | 86.35670857142857 | 90.00546833333334 | 72.99547 | 80.42724333333335 |
| MAPK8 | 342.36497142857144 | 360.65349999999995 | 304.83147999999994 | 326.80421666666666 |
| NOTCH1 | 180.1597285714286 | 197.74179999999998 | 159.570006 | 185.56858333333332 |
| NOTCH4 | 114.82108714285714 | 101.45157999999999 | 124.94732400000001 | 127.94365166666667 |
| PLEK | 181.2551014285714 | 209.27827 | 284.28643999999997 | 313.81871666666666 |
| PRKCG | 12.49196157142857 | 29.79382166666667 | 13.448451 | 8.231633166666667 |
| PRKD1 | 50.08191028571429 | 52.940975 | 60.40242599999999 | 56.32949166666666 |
| RIPK1 | 165.36738571428572 | 204.45658333333333 | 182.84218 | 192.47128333333333 |
| RPS27L | 34.03548142857143 | 42.32530333333334 | 41.893367999999995 | 42.159056666666665 |
| TRAF1 | 33.456784285714285 | 45.13173316666667 | 47.541380000000004 | 79.0461 |
| TRAF2 | 337.96212857142854 | 325.1025833333333 | 322.68222000000003 | 371.7632833333334 |
| VPS13A | 809.922057142857 | 748.0797166666667 | 789.31642 | 655.1626499999999 |
| VPS34 | 886.6840857142857 | 732.3339166666666 | 830.4621 | 792.9527333333332 |
### Chart
| Category | ADU | AG | ADU-PD | AG-PD |
|---|---|---|---|---|
| CDKN1A | 2006.0064285714284 | 2347.3573333333334 | 1985.55834 | 2107.1036666666664 |
| CHUK | 1793.286142857143 | 1772.1091666666669 | 1815.8557999999998 | 1824.3555 |
| CSNK2A1 | 2202.3245714285713 | 2178.2119999999995 | 2242.0298000000003 | 2137.4535 |
| MAP2K1 | 3327.5737142857142 | 3099.83 | 3278.4440000000004 | 3374.1109999999994 |
| MAP2K3 | 1578.4394285714284 | 1537.7086666666667 | 1485.2899999999997 | 1520.6544999999999 |
| MAP2K4 | 1340.3392857142856 | 1360.692 | 1214.61446 | 1098.9159166666668 |
| MAP4K4 | 1682.1024285714286 | 2076.227 | 1951.6586 | 1766.4781666666668 |
| MAPK1 | 4274.454857142858 | 4305.571833333334 | 4072.1767999999997 | 4010.5268333333333 |
| MAPK13 | 4720.257714285714 | 4425.694333333334 | 4136.0786 | 3790.163 |
| MAPK3 | 2005.6298571428572 | 2060.4996666666666 | 1958.2798000000003 | 1872.1883333333333 |
| NOTCH2 | 3259.4977142857138 | 3005.334833333334 | 3065.8808 | 2726.581166666667 |
| NOTCH3 | 1446.3304285714287 | 1202.2534166666667 | 1329.0456 | 1350.4366333333332 |
| PRKCZ | 1225.3539285714287 | 994.37785 | 1074.6461599999998 | 981.3474333333334 |Normalized Expression
TRANSCRIPTION FACTORS
### Chart
| Category | ADU | AG | ADU-PD | AG-PD |
|---|---|---|---|---|
| CEBPA | 4343.958285714286 | 4189.817333333333 | 3764.4044000000004 | 3397.552333333333 |
| CEBPB | 4819.596857142858 | 5464.305333333334 | 4945.410400000001 | 4428.8015000000005 |
| EHF | 16063.024285714286 | 17576.531666666666 | 17999.874 | 15697.808333333334 |
| FOS | 878.1079142857143 | 1512.7741166666667 | 4568.1939600000005 | 3849.2663666666667 |
| JUNB | 1806.291857142857 | 1515.1163333333334 | 1896.6009999999999 | 1841.5286000000003 |
| KRAS | 1562.7764285714281 | 1590.7243333333333 | 1507.2496800000001 | 1463.2453333333333 |
| PRR13 | 4234.863857142857 | 4747.597666666667 | 4934.365 | 5297.150666666666 |
| RAF1 | 1846.0878571428573 | 1755.3216666666667 | 1930.8120000000004 | 1857.0106666666668 |
### Chart
| Category | ADU | AG | ADU-PD | AG-PD |
|---|---|---|---|---|
| ETS1 | 74.81103428571429 | 85.43488 | 115.252086 | 101.75508 |
| FOSB | 113.23203714285715 | 160.27317166666663 | 387.50963800000005 | 362.35516666666666 |
| MITF | 111.96203 | 101.88245166666667 | 119.77439399999999 | 107.57875666666666 |
| PRR10 | 72.35932571428572 | 72.39665833333335 | 74.342184 | 82.48895833333333 |
| PRR12 | 128.03334285714286 | 140.47876666666667 | 149.49438 | 147.36391666666665 |
| PRR14 | 639.0496714285715 | 617.9022500000001 | 662.01334 | 651.7188833333333 |
| PRR15 | 523.9643857142858 | 456.6541666666667 | 582.3158000000001 | 760.8472 |
| PRR16 | 188.26731714285717 | 220.93203333333335 | 195.13364 | 193.86769999999999 |
| PRR18 | 41.41033971428571 | 78.46863333333333 | 59.449776 | 92.844665 |
| PRR19 | 150.42990000000003 | 212.18605000000002 | 179.47146000000004 | 189.31571666666665 |
| PRR22 | 44.52809714285714 | 54.28513649999999 | 38.71063 | 45.66394333333333 |
| PRR3 | 250.90057142857137 | 238.79876666666667 | 226.08092000000002 | 197.96033333333335 |
| PRR4 | 78.75012285714286 | 65.90615333333334 | 102.721214 | 98.36367333333334 |
| PRR7 | 249.35082857142856 | 235.43805999999998 | 196.64392 | 242.1339 |
| TCF3 | 486.3822857142858 | 483.87795 | 544.17966 | 609.3510499999999 |
| TWIST1 | 671.1254714285715 | 644.3884 | 768.84488 | 696.8334166666667 |
| ZEB1 | 368.64397142857143 | 394.84386666666666 | 398.17398000000003 | 445.8198666666667 |
| ZEB2 | 617.9617142857143 | 648.2477333333334 | 696.42534 | 749.7059333333333 |

## Slide 6
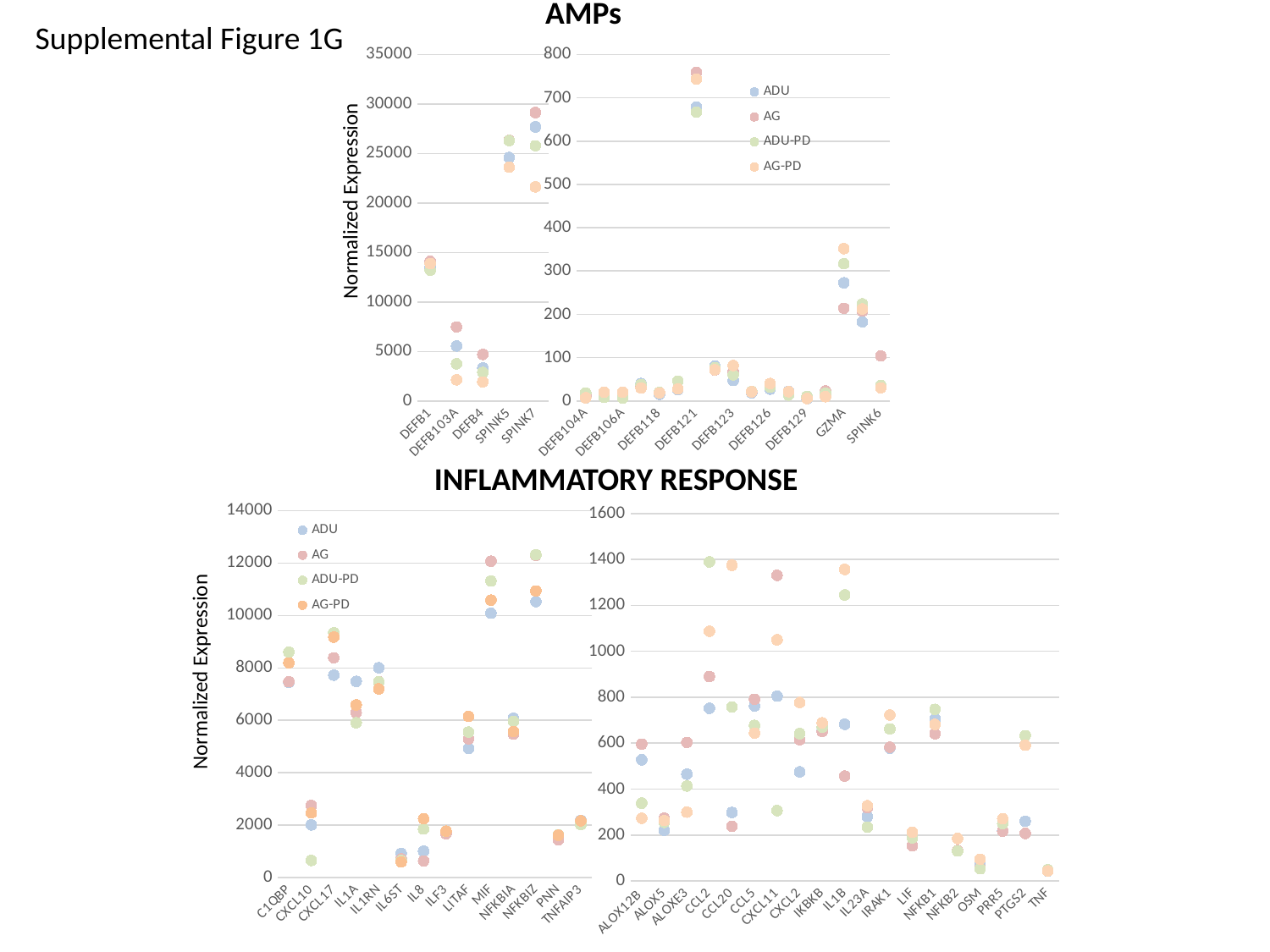

AMPs
Supplemental Figure 1G
### Chart
| Category | ADU | AG | ADU-PD | AG-PD |
|---|---|---|---|---|
| DEFB1 | 13504.33857142857 | 14097.518333333333 | 13214.784 | 13866.193166666666 |
| DEFB103A | 5550.879142857143 | 7472.891666666666 | 3744.84778 | 2133.2277333333336 |
| DEFB4 | 3325.176857142857 | 4694.238166666666 | 2870.72862 | 1915.4025000000001 |
| SPINK5 | 24589.018571428573 | 26323.408333333336 | 26297.72 | 23621.215 |
| SPINK7 | 27682.07285714286 | 29132.013333333332 | 25777.798 | 21622.08666666667 |
### Chart
| Category | ADU | AG | ADU-PD | AG-PD |
|---|---|---|---|---|
| DEFB104A | 6.9310727142857145 | 12.388351833333333 | 18.4629038 | 7.2472805 |
| DEFB105A | 15.114451714285712 | 16.567243166666668 | 8.094167 | 20.418120000000002 |
| DEFB106A | 8.263080857142857 | 9.539512833333335 | 6.7760386 | 20.1303345 |
| DEFB108B | 40.212936 | 30.98025816666667 | 38.0936512 | 29.8441565 |
| DEFB118 | 15.26531742857143 | 19.83182333333333 | 19.674371400000002 | 18.616204333333332 |
| DEFB119 | 25.907934285714287 | 45.70173166666667 | 45.925844000000005 | 27.787341666666666 |
| DEFB121 | 678.9720857142858 | 758.7927666666668 | 666.86488 | 743.3893666666667 |
| DEFB122 | 80.87102142857144 | 71.56586666666666 | 76.728764 | 71.95209833333334 |
| DEFB123 | 47.10169571428571 | 68.21442666666667 | 60.565322 | 81.90908666666667 |
| DEFB125 | 18.549664000000003 | 20.6141645 | 21.99271 | 21.107739833333337 |
| DEFB126 | 27.367950714285712 | 35.542162 | 31.617669799999998 | 40.23641833333333 |
| DEFB127 | 22.34643442857143 | 20.591689500000005 | 13.7560088 | 21.541033166666665 |
| DEFB129 | 5.3854617142857135 | 9.947149666666666 | 10.674295 | 5.808682999999999 |
| DEFB132 | 19.377605285714285 | 22.89773733333334 | 18.348206800000003 | 9.886611666666667 |
| GZMA | 272.6734 | 213.95351666666667 | 316.89928 | 351.64410000000004 |
| BPIFA1 | 182.8457142857143 | 207.39405 | 223.91962000000004 | 212.36434999999997 |
| SPINK6 | 34.66508142857143 | 103.93498666666666 | 35.932274 | 30.244731666666667 |Normalized Expression
INFLAMMATORY RESPONSE
### Chart
| Category | ADU | AG | ADU-PD | AG-PD |
|---|---|---|---|---|
| C1QBP | 7448.871857142857 | 7471.766333333333 | 8603.6122 | 8193.412 |
| CXCL10 | 2010.299685714286 | 2748.7585 | 655.2834599999999 | 2466.2698666666665 |
| CXCL17 | 7724.133 | 8386.426666666666 | 9338.556 | 9175.644666666665 |
| IL1A | 7486.234571428572 | 6290.418333333332 | 5904.3996 | 6589.2300000000005 |
| IL1RN | 8004.141857142857 | 7478.795666666668 | 7471.3604 | 7198.446333333333 |
| IL6ST | 920.2161571428571 | 714.5618666666668 | 653.01656 | 597.38855 |
| IL8 | 1007.1653142857142 | 636.3718833333334 | 1851.47346 | 2246.050516666666 |
| ILF3 | 1698.6618571428573 | 1673.0273333333334 | 1763.708 | 1771.926333333333 |
| LITAF | 4926.075428571428 | 5291.616000000001 | 5550.662 | 6145.517666666667 |
| MIF | 10087.55357142857 | 12070.203333333333 | 11314.7222 | 10583.065333333334 |
| NFKBIA | 6079.818714285714 | 5468.3994999999995 | 5957.8192 | 5569.033833333334 |
| NFKBIZ | 10530.017857142857 | 12293.930999999999 | 12318.34 | 10937.633999999998 |
| PNN | 1578.4954285714284 | 1445.9348666666667 | 1632.4128 | 1614.8016666666665 |
| TNFAIP3 | 2184.677 | 2031.1451666666665 | 2021.8985999999998 | 2165.237333333333 |
### Chart
| Category | ADU | AG | ADU-PD | AG-PD |
|---|---|---|---|---|
| ALOX12B | 527.5319714285714 | 596.0500333333333 | 338.5065 | 273.02794 |
| ALOX5 | 220.45941428571427 | 273.24068333333327 | 255.314 | 262.87393333333335 |
| ALOXE3 | 465.08127142857137 | 603.0360666666667 | 413.3221 | 299.57576666666665 |
| CCL2 | 751.9263857142857 | 890.0832333333333 | 1389.1270399999999 | 1087.3217333333334 |
| CCL20 | 298.1521771428571 | 238.05596666666668 | 757.0184999999999 | 1374.5997666666665 |
| CCL5 | 762.1592142857143 | 791.45735 | 677.5508400000001 | 643.8782500000001 |
| CXCL11 | 804.7071757142857 | 1330.8069333333335 | 306.292666 | 1050.1110833333335 |
| CXCL2 | 474.6190285714286 | 614.7021333333333 | 641.36348 | 776.9802166666667 |
| IKBKB | 654.4845571428572 | 650.8812166666665 | 669.8860000000001 | 688.72985 |
| IL1B | 682.1201571428572 | 456.55435000000006 | 1245.35746 | 1356.6020499999997 |
| IL23A | 280.3339142857143 | 318.68350000000004 | 234.45766000000003 | 327.4946166666667 |
| IRAK1 | 578.2032 | 582.5621666666667 | 662.22744 | 722.6345333333333 |
| LIF | 188.45565714285718 | 152.717885 | 188.02256000000003 | 211.64256333333333 |
| NFKB1 | 705.7001714285715 | 641.0405166666667 | 747.38986 | 681.6355666666667 |
| NFKB2 | 132.8355857142857 | 132.26511166666668 | 130.36520000000002 | 184.84069999999997 |
| OSM | 74.91035571428571 | 56.45618666666667 | 52.728818000000004 | 94.94907333333333 |
| PRR5 | 219.25554285714287 | 216.45020000000002 | 249.60153999999997 | 271.0874166666667 |
| PTGS2 | 259.7828457142857 | 207.049815 | 632.66828 | 591.0021999999999 |
| TNF | 42.198529857142866 | 47.85888666666667 | 48.283618000000004 | 43.628473333333325 |Normalized Expression

## Slide 7
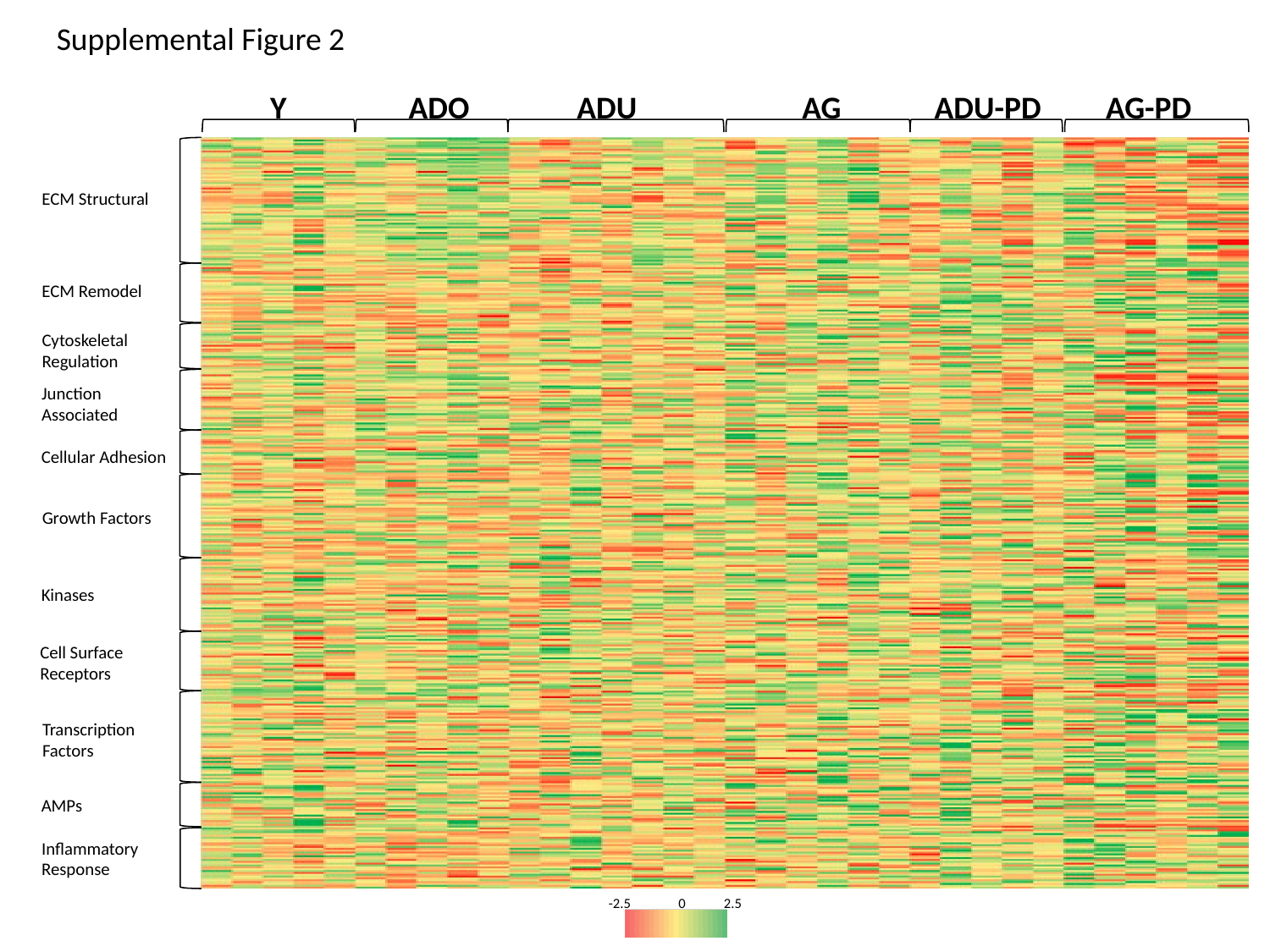

Supplemental Figure 2
Y ADO ADU AG ADU-PD AG-PD
ECM Structural
ECM Remodel
Cytoskeletal
Regulation
Junction
Associated
Cellular Adhesion
Growth Factors
Kinases
Cell Surface
Receptors
Transcription
Factors
AMPs
Inflammatory
Response
-2.5 0 2.5

## Slide 8
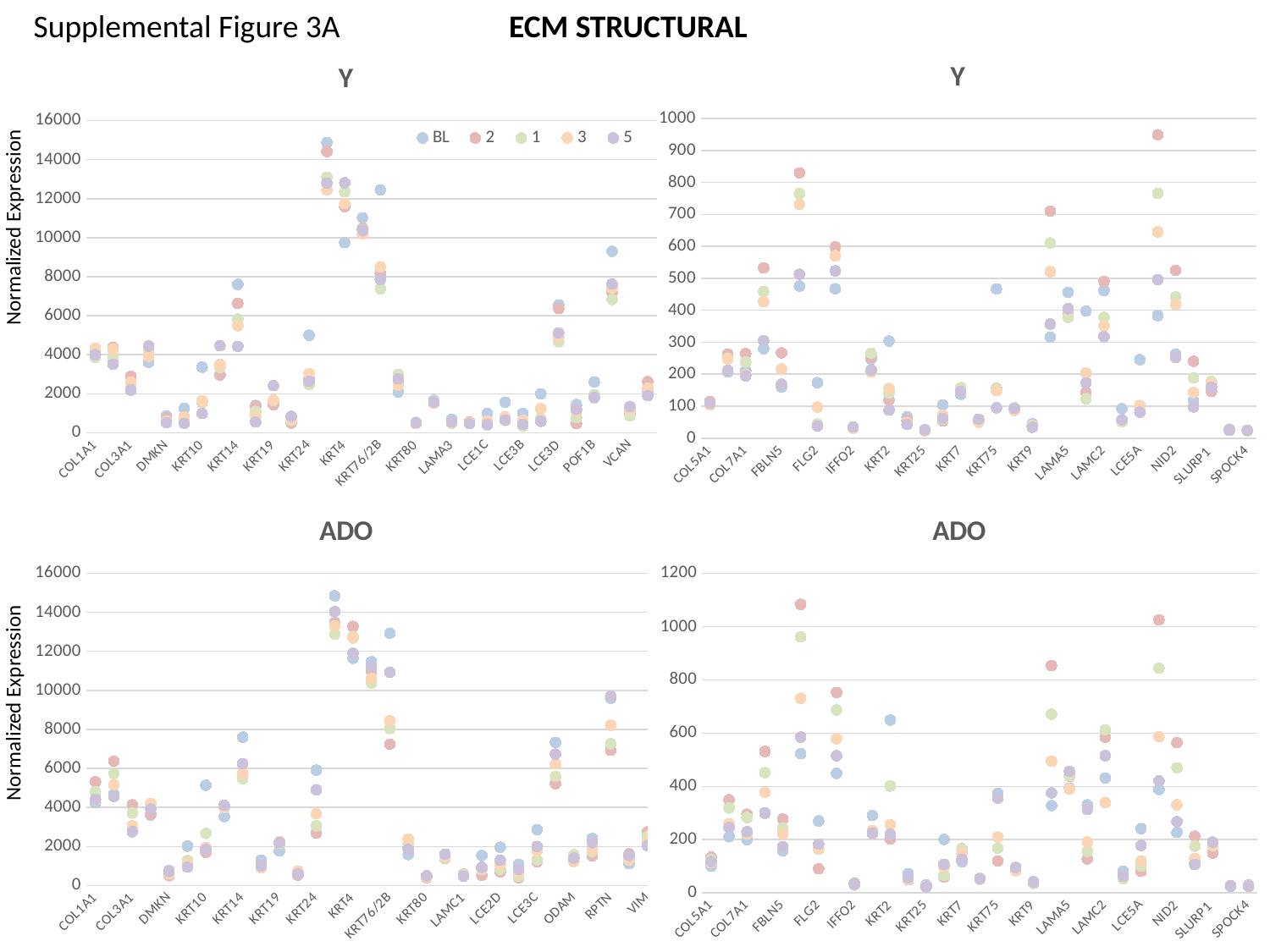

Supplemental Figure 3A
ECM STRUCTURAL
### Chart: Y
| Category | BL | 2 | 1 | 3 | 5 |
|---|---|---|---|---|---|
| COL5A1 | 103.83730700279264 | 115.02179221614948 | 108.2889148598513 | 103.49258279531341 | 109.98493194760464 |
| COL5A2 | 206.9037559655224 | 263.1209057728872 | 249.36122125544387 | 247.9934480108377 | 212.09565318984653 |
| COL7A1 | 211.0160981608995 | 264.82107946760334 | 238.19031461402042 | 198.99446165431488 | 194.84319254880705 |
| CRISPLD2 | 279.87446899947224 | 532.8545393281023 | 459.0820214095468 | 426.7746939218787 | 305.24238176570293 |
| FBLN5 | 159.83593343950758 | 267.19468470303724 | 216.36061172132963 | 215.60024516429655 | 168.85241935626405 |
| FBN1 | 475.7091808641447 | 830.0527662569584 | 765.496402120861 | 731.629243389512 | 512.8045833438233 |
| FLG2 | 173.59231129795768 | 43.589313496988964 | 44.393585856209604 | 97.12173919395953 | 38.28524723795115 |
| HSPG2 | 467.50852670009783 | 598.5697752210342 | 570.9152564498935 | 569.7495539087672 | 523.4860871214094 |
| IFFO2 | 31.271146801422738 | 31.891374768926752 | 31.290640837387134 | 31.880782069443185 | 35.42444794986639 |
| KRT18 | 247.94233464885363 | 252.93532214921484 | 265.82873988888775 | 206.8577763660112 | 213.41954282588415 |
| KRT2 | 303.75427916762015 | 118.92350249539825 | 141.44536569527557 | 155.144760029756 | 87.68543031679378 |
| KRT20 | 67.03210251629831 | 55.22931370725608 | 43.046894111188614 | 49.093389204404026 | 43.762964972244085 |
| KRT25 | 23.17864032998878 | 22.87006184563326 | 25.241703276144776 | 22.639594213100384 | 26.520605093840334 |
| KRT27 | 104.18289877120645 | 53.3085499477593 | 58.588150526576264 | 71.57405816257491 | 61.520289412825754 |
| KRT7 | 137.46395905176803 | 153.7939090319818 | 158.45829574625552 | 145.9481579199711 | 146.59589778806165 |
| KRT74 | 54.6222186998165 | 53.044392051841236 | 57.85848091165557 | 49.89141193431526 | 59.55892354844161 |
| KRT75 | 467.0302420125315 | 155.982051890118 | 153.40744294942354 | 148.78090420549572 | 95.20957909488136 |
| KRT8 | 91.73425796507262 | 88.39233775299031 | 95.0887883562969 | 87.24259195533062 | 93.28565648431419 |
| KRT9 | 45.086885163929225 | 38.387994377365985 | 40.41512050151584 | 36.49643637542284 | 34.1075430527421 |
| LAMA4 | 316.37174377164735 | 710.8874106621893 | 610.770926014838 | 521.008032475713 | 356.93133516779505 |
| LAMA5 | 456.4866762186913 | 391.4937600313299 | 377.89480542675324 | 402.8460215571452 | 405.10869446722353 |
| LAMB4 | 397.75620887417415 | 142.5743677664514 | 123.28209025083646 | 203.68700557427016 | 173.40946158036127 |
| LAMC2 | 462.0328481052143 | 490.7125554747024 | 378.20657915877206 | 351.7256510293816 | 317.83094738299616 |
| LCE2A | 91.99998374932528 | 57.86994672941458 | 51.53332117101641 | 55.552026568240954 | 58.04665018387306 |
| LCE5A | 245.58111980491185 | 87.66277202927341 | 95.43404880325815 | 102.1122366793957 | 81.48681681974176 |
| NID1 | 383.142870520649 | 949.3982733229054 | 765.7335342900354 | 645.6363291293567 | 496.1642609294035 |
| NID2 | 262.55356795458243 | 525.3448689948502 | 442.3948343497463 | 418.0247883734911 | 252.60019723005846 |
| NTN1 | 117.70751189908987 | 240.53530606744567 | 188.35927994250895 | 142.8985173125497 | 97.63363998072911 |
| SLURP1 | 174.63957549556304 | 145.94290819211966 | 177.7024696617455 | 169.98974242476172 | 159.90829050877343 |
| SPARC | 24.425365440360824 | 25.35484003854928 | 28.045975714185715 | 26.268278946797384 | 26.547426335170933 |
| SPOCK4 | 24.459574239805093 | 24.950297173362557 | 23.297309797263917 | 23.54904752650975 | 24.956703709372366 |
### Chart: Y
| Category | BL | 2 | 1 | 3 | 5 |
|---|---|---|---|---|---|
| COL1A1 | 3913.735670399062 | 4224.009926849664 | 3855.3623113081358 | 4335.062358222789 | 3998.611714648871 |
| COL1A2 | 3615.049536067149 | 4376.6725857306155 | 3938.4740253260534 | 4282.587086618695 | 3517.8680931193758 |
| COL3A1 | 2179.621003043143 | 2893.139870966155 | 2573.6486246589943 | 2590.9463001084478 | 2217.2328666527164 |
| CRNN | 3605.7855932129887 | 4011.6702931035306 | 4139.60415908513 | 3925.2652965356265 | 4441.106778539226 |
| DMKN | 861.7386023097773 | 751.3170754391317 | 598.3119134887826 | 561.2471341444165 | 515.3360635238744 |
| KRT1 | 1250.8706316524392 | 525.2243027333104 | 642.8794198718565 | 799.421124488757 | 479.7194842966362 |
| KRT10 | 3357.5716871028476 | 1601.3031425714123 | 1530.336476897344 | 1649.1295545575438 | 983.4814357147516 |
| KRT13 | 2982.0001230848675 | 2958.2071377007437 | 3338.6212309486496 | 3496.15804600549 | 4459.2210949796745 |
| KRT14 | 7600.109416662646 | 6631.074653766958 | 5820.7000101926205 | 5480.465483909803 | 4426.2635674958265 |
| KRT17 | 1393.4948932349957 | 1355.6073303335954 | 1089.4127708880214 | 857.5040354480602 | 551.7064545868803 |
| KRT19 | 1480.4333762591195 | 1438.0216233641456 | 1659.0556614717507 | 1675.7582937985003 | 2414.607596438353 |
| KRT23 | 571.8013844349827 | 499.81404879359695 | 632.9058366842902 | 695.2884063269698 | 842.8455364261638 |
| KRT24 | 4994.0902644798725 | 2781.422440645565 | 2466.52075715415 | 3027.786555414811 | 2630.787387247841 |
| KRT3/6B | 14870.518452224916 | 14413.60498605713 | 13103.260634688102 | 12448.89762590331 | 12797.81422737922 |
| KRT4 | 9745.880584883578 | 11583.573739955864 | 12348.468148397315 | 11744.182500623114 | 12817.465316381576 |
| KRT5 | 11012.618845819892 | 10478.483510026446 | 10397.732804461839 | 10185.982155225527 | 10380.116301155018 |
| KRT76/2B | 12446.617815586984 | 8178.765164042726 | 7375.022893534339 | 8496.647245112446 | 7856.823575453553 |
| KRT78 | 2089.062764249888 | 2622.4638244024545 | 2994.043494399071 | 2430.9868182199293 | 2761.10246142413 |
| KRT80 | 503.62436499569463 | 511.8973982645955 | 527.5846076687835 | 469.6519124115523 | 505.5159582884257 |
| LAD1 | 1666.946146614988 | 1572.8691875173504 | 1644.395372888168 | 1505.5706825599575 | 1580.706517838512 |
| LAMA3 | 687.3188354132404 | 505.3650065405143 | 485.8083286077938 | 525.6523732448585 | 555.223269534641 |
| LAMC1 | 510.264419526039 | 564.5241035337682 | 498.8467761609259 | 512.1270465864354 | 464.9551823360325 |
| LCE1C | 991.0801363229303 | 432.97944395316085 | 393.13843933168846 | 604.7101626948775 | 434.9452544797979 |
| LCE2D | 1567.9952759671144 | 736.5028092557283 | 629.7295281101971 | 816.8592726625513 | 653.0559626654999 |
| LCE3B | 989.2582488284175 | 425.90218703364127 | 338.0709855445076 | 635.6026641217019 | 429.5085448873367 |
| LCE3C | 1993.7917090583069 | 1231.1601995908795 | 717.1932755626588 | 1234.0123050768946 | 585.4596720829747 |
| LCE3D | 6552.467851152276 | 6362.813177963839 | 4667.22696613007 | 4911.567970113435 | 5115.609761815533 |
| ODAM | 1441.1034250863613 | 470.9850164097486 | 782.0855829237112 | 1118.3213786616143 | 1210.6225442758168 |
| POF1B | 2600.826651493402 | 1870.8541686906638 | 1954.0511248590717 | 1789.2738991936005 | 1810.4504566344676 |
| RPTN | 9296.001702174084 | 7177.478072383309 | 6828.3613614696105 | 7411.99333923084 | 7618.503157207819 |
| VCAN | 949.0313046850218 | 1060.8881959830753 | 866.6761737407612 | 1164.936833278346 | 1338.6277301375349 |
| VIM | 2121.9389678465814 | 2621.629955366528 | 2288.868503339958 | 2285.57987704313 | 1904.3898635122775 |Normalized Expression
### Chart: ADO
| Category | BL | 2 | 1 | 3 | 5 |
|---|---|---|---|---|---|
| COL1A1 | 4234.568765265225 | 5312.952796177897 | 4802.971451813474 | 4403.364391657054 | 4424.222236880014 |
| COL1A2 | 4661.138756648933 | 6367.836853216477 | 5739.448926864641 | 5169.687693633987 | 4558.707848018365 |
| COL3A1 | 2750.841083164633 | 4128.664177287889 | 3712.2453511561926 | 3057.2708820254725 | 2769.423458199215 |
| CRNN | 3607.6473640409904 | 3661.1250776826473 | 3922.6162235916727 | 4206.336046302046 | 3917.7731862809064 |
| DMKN | 762.0846058992291 | 503.32303891748234 | 621.4961724843157 | 592.7594113159709 | 711.2823862938799 |
| KRT1 | 2016.3733687178294 | 1055.143931231197 | 1285.772573700456 | 1135.8926299531286 | 939.0509099390388 |
| KRT10 | 5135.381367662765 | 1694.635147212744 | 2669.7389733688515 | 1951.9194552109677 | 1838.8352679077968 |
| KRT13 | 3533.106242883571 | 4013.2932475405573 | 3978.3919137541216 | 4029.040182099875 | 4102.598426582841 |
| KRT14 | 7601.2624738058885 | 5633.985716319549 | 5466.473691658038 | 5749.773372082374 | 6243.3660248552915 |
| KRT17 | 1299.4259913723242 | 1015.3930420634234 | 919.4995372779841 | 896.2565501661588 | 999.9798070362987 |
| KRT19 | 1770.511527286595 | 2143.806355923281 | 2163.485772971677 | 2246.227836956453 | 2200.4626029827277 |
| KRT23 | 573.793127816593 | 526.7610018363433 | 665.8589211387067 | 742.7529186260805 | 593.7496250985928 |
| KRT24 | 5910.189304951864 | 2677.3816294990042 | 3056.6526441180185 | 3672.527658574301 | 4904.801393784576 |
| KRT3/6B | 14846.581451386543 | 13464.080959718187 | 12883.505383720953 | 13304.463519061563 | 14033.058901141201 |
| KRT4 | 11639.368391626615 | 13276.43728580482 | 12756.110978502453 | 12678.430550351426 | 11895.502156002394 |
| KRT5 | 11464.772169565545 | 11001.721552230025 | 10379.128145500927 | 10615.638383913818 | 11219.04079506657 |
| KRT76/2B | 12930.183158149324 | 7245.6116909247685 | 8044.473510618783 | 8444.817421432954 | 10925.631462455654 |
| KRT78 | 1585.2134140687704 | 1948.8647374660452 | 2133.870056395694 | 2376.8074015600055 | 1835.9664951568957 |
| KRT80 | 441.677322575978 | 394.4705925780802 | 433.60108882002226 | 455.59193479290036 | 499.3345437707969 |
| LAD1 | 1521.0081720968437 | 1388.0564685431289 | 1421.4352791496083 | 1515.151619610559 | 1608.1473095519236 |
| LAMC1 | 489.7165088390943 | 593.3682487009343 | 569.3895078260814 | 487.3136248842958 | 478.06471607720334 |
| LCE1C | 1536.1925130662432 | 522.6481220297767 | 810.6183941570753 | 881.6129836473876 | 932.7870290112346 |
| LCE2D | 1960.1682690042778 | 695.0693586371667 | 838.4036335274939 | 1086.9164296919462 | 1295.9887304970625 |
| LCE3B | 1087.9212654626272 | 407.3850891886862 | 477.54888686952734 | 656.2581982856981 | 822.7507709827358 |
| LCE3C | 2862.9339802745158 | 1213.649450958198 | 1312.9424357357382 | 1786.7181080321616 | 2000.8877315338189 |
| LCE3D | 7332.35517231753 | 5213.48346041637 | 5589.189039730616 | 6232.246854210504 | 6730.355274345194 |
| ODAM | 1236.5908401853444 | 1440.9001314806783 | 1574.9898592705406 | 1228.187836285697 | 1401.9948760303073 |
| POF1B | 2408.6796580296404 | 1513.0827088763078 | 1744.1915895462325 | 1879.4963801054587 | 2193.616577561085 |
| RPTN | 9600.122274490664 | 6926.088595946067 | 7263.77985552174 | 8212.396686864593 | 9703.07548353368 |
| VCAN | 1119.7794157775393 | 1617.3468891359507 | 1354.0079258908586 | 1307.9096330267753 | 1532.1715339921868 |
| VIM | 2252.8926535353835 | 2741.281931258023 | 2490.0987239909045 | 2224.305801083922 | 2047.5360032484123 |
### Chart: ADO
| Category | BL | 2 | 1 | 3 | 5 |
|---|---|---|---|---|---|
| COL5A1 | 100.42388537201558 | 134.70734529941842 | 125.58402364248354 | 113.42627695268986 | 118.02006296091713 |
| COL5A2 | 211.06649379667036 | 350.1504502102152 | 319.1844224058083 | 259.7382604087103 | 244.9358251237249 |
| COL7A1 | 200.13746946162132 | 295.4278096736894 | 283.4548898810542 | 221.73287050285833 | 230.13344005243567 |
| CRISPLD2 | 301.62987904804856 | 531.2835630650452 | 451.5464764792351 | 377.8522745653093 | 299.10033352545014 |
| FBLN5 | 158.34080300274766 | 277.02111026331 | 244.07206600371748 | 221.15233801614212 | 173.73925888519165 |
| FBN1 | 522.5741073636522 | 1083.617633516279 | 961.3345611906973 | 730.7439973596161 | 585.1184591616327 |
| FLG2 | 270.4105673502233 | 90.91123551543737 | 164.2907235854447 | 166.51796559164288 | 183.63839006364418 |
| HSPG2 | 449.1254364031927 | 752.9085656115975 | 687.1388231314744 | 579.0781239894751 | 515.1101332402923 |
| IFFO2 | 37.18099650609507 | 35.27595000839795 | 29.699554427413577 | 34.05181781412607 | 33.17036096317188 |
| KRT18 | 290.6453279008907 | 221.75136770767918 | 223.60272794390272 | 234.73253119294966 | 225.4343885037196 |
| KRT2 | 649.4240840172032 | 202.54641773776407 | 401.297443822587 | 256.6873671358018 | 219.9642255608244 |
| KRT20 | 72.67917357100511 | 49.29544644247263 | 54.42849554477444 | 52.032951156890256 | 57.891407077848335 |
| KRT25 | 30.365787285823558 | 24.489320738071015 | 21.82999893643627 | 24.820625141261694 | 23.041424667626156 |
| KRT27 | 201.12813069338858 | 59.770709875837774 | 66.53397371458374 | 97.1234466551438 | 107.8242851599718 |
| KRT7 | 116.83129920513299 | 165.83053900433848 | 163.75113671206924 | 149.09431908431236 | 126.73393954096225 |
| KRT74 | 53.936888849156304 | 55.70694483721357 | 55.82849397559973 | 50.77354840802957 | 53.71110533717427 |
| KRT75 | 374.2058863934387 | 119.91152554137582 | 168.31911702046207 | 209.90625237437675 | 356.0011578027595 |
| KRT8 | 89.13395499476304 | 85.13825440171904 | 83.96972756306721 | 83.7744661705907 | 95.71513315333912 |
| KRT9 | 42.3975522503132 | 36.14725175198499 | 37.87884817375642 | 40.4138640526812 | 40.84287674833176 |
| LAMA4 | 328.1110198072897 | 853.5728266977717 | 671.1696202008183 | 494.4005565795758 | 375.48881051461507 |
| LAMA5 | 393.36017300609615 | 438.0471920983566 | 440.17507025217805 | 390.28661603376844 | 456.7732301999747 |
| LAMB4 | 330.7089884999251 | 128.14879015584472 | 156.35554262577077 | 190.8851976732962 | 313.8534233287011 |
| LAMC2 | 431.6586948412049 | 584.6907916387684 | 612.9261254829892 | 338.98525349095206 | 515.2868756894355 |
| LCE2A | 81.2924169674032 | 55.62603530094998 | 52.57877356965807 | 59.23847969007925 | 62.95703968149548 |
| LCE5A | 241.26739991824394 | 81.37620708960736 | 100.11310176887395 | 120.10156475674528 | 178.14198627366312 |
| NID1 | 388.6227763927101 | 1025.163708043511 | 843.7253290277889 | 586.2827705072964 | 420.59981752407504 |
| NID2 | 227.38387300356584 | 564.2856936675341 | 470.0714169811185 | 330.79898981577645 | 267.9228776403778 |
| NTN1 | 115.00278496626521 | 212.92238567180357 | 175.6232733931765 | 129.5526958836854 | 106.73947152022342 |
| SLURP1 | 189.25044090017158 | 149.7619606516862 | 183.55914343508266 | 177.318206505524 | 191.13359283112266 |
| SPARC | 24.716458035490167 | 27.59250926291882 | 26.609903547637042 | 27.070883725911457 | 26.632248921650127 |
| SPOCK4 | 30.10658914266926 | 26.894539885409404 | 23.573158757021492 | 26.60936958499361 | 25.548966689733618 |Normalized Expression

## Slide 9
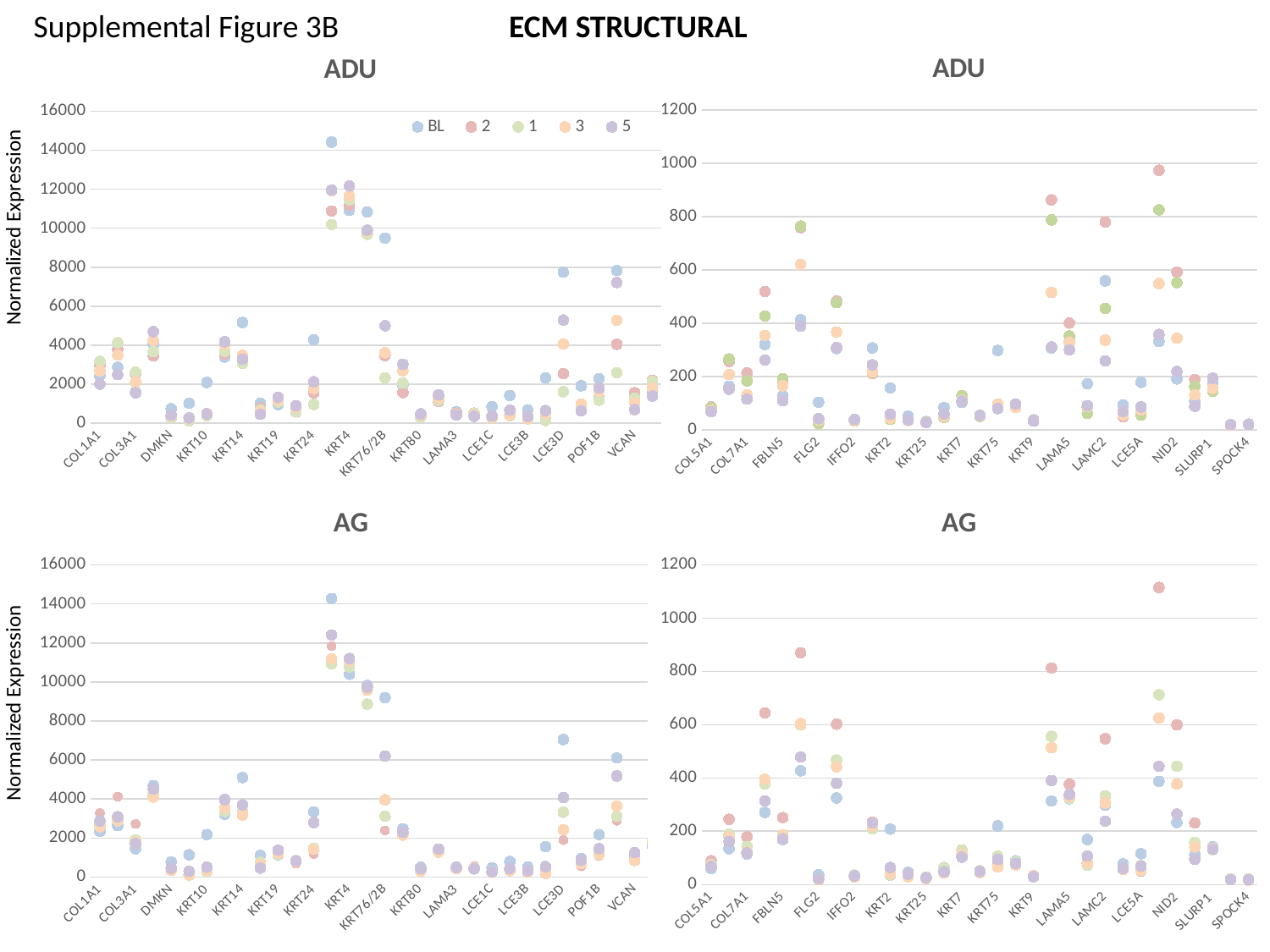

Supplemental Figure 3B
ECM STRUCTURAL
### Chart: ADU
| Category | BL | 2 | 1 | 3 | 5 |
|---|---|---|---|---|---|
| COL5A1 | 78.68808859570078 | 85.3461110845912 | 81.23487159135102 | 71.96694943673306 | 67.8152323808772 |
| COL5A2 | 163.05850863986421 | 256.03221129760095 | 265.0819303040918 | 206.44023092838532 | 151.90547854827736 |
| COL7A1 | 125.63392599235219 | 212.88776177549718 | 183.12688773554314 | 131.02523845137011 | 114.38379496167425 |
| CRISPLD2 | 320.06995051728387 | 518.4714574374055 | 426.57764578834525 | 353.8046945619371 | 260.94372608821806 |
| FBLN5 | 126.88623668035919 | 185.5848639055293 | 191.5718042895599 | 164.95766454247482 | 108.35595772539092 |
| FBN1 | 412.35504584908807 | 758.5215321461969 | 764.1116979193131 | 620.4333762930534 | 388.06429496666925 |
| FLG2 | 102.91932206789521 | 24.290794634496933 | 22.542010702693585 | 36.31887798375389 | 41.17139555532655 |
| HSPG2 | 304.46123228385994 | 482.78282346870765 | 476.6995898196196 | 366.5407978469145 | 308.1636077740775 |
| IFFO2 | 38.48978196176558 | 33.67485870030357 | 32.7464785802197 | 34.679191546677565 | 38.31596909896811 |
| KRT18 | 306.6288217441043 | 210.71546786852676 | 213.70301732747396 | 216.95057443797947 | 243.48809107062328 |
| KRT2 | 156.97218842155607 | 42.94239661523764 | 38.17029462462608 | 43.179587163204815 | 57.439714720734806 |
| KRT20 | 50.48295144271357 | 34.981816928297604 | 35.5947972896072 | 34.53900611021686 | 35.65782071296534 |
| KRT25 | 30.183326366893905 | 28.512612156888842 | 27.71518459004183 | 26.818887352917184 | 26.331368180100263 |
| KRT27 | 83.1121604332392 | 47.18311977195523 | 45.3542874636286 | 50.08484979573626 | 58.85000756245964 |
| KRT7 | 101.95506167317583 | 127.16769579621041 | 127.96856736529682 | 110.3382879637496 | 107.05178563966322 |
| KRT74 | 52.67777994259596 | 53.19669417807136 | 49.641184728043385 | 51.38352645985765 | 53.44782725377218 |
| KRT75 | 297.5341166646557 | 89.29766026884293 | 89.43486763392008 | 96.76110559903665 | 79.15038692868855 |
| KRT8 | 96.11310602766883 | 83.00987714888505 | 90.29618412820695 | 83.83666882843102 | 95.25862501841199 |
| KRT9 | 36.45843920977657 | 33.64736034161283 | 31.81137895466266 | 31.851589176152306 | 31.749885059214698 |
| LAMA4 | 306.6759981010483 | 862.5804543237102 | 787.5153228431193 | 515.0679391943418 | 310.54718057896423 |
| LAMA5 | 331.2319704849695 | 400.017543086014 | 351.787090299684 | 326.7928476412774 | 299.4234384483243 |
| LAMB4 | 172.01726398517505 | 85.23729512841429 | 61.140922187004875 | 85.76084296484363 | 90.18261519162964 |
| LAMC2 | 558.5190343971026 | 779.6582641931269 | 454.9559652051146 | 335.9615649502733 | 258.6656158443231 |
| LCE2A | 93.39874938340364 | 47.82266893100758 | 54.401846970703964 | 55.936809370899404 | 68.38557312724284 |
| LCE5A | 177.6011590595135 | 65.16776187014499 | 54.93647373138461 | 73.60350571635735 | 85.05983722733035 |
| NID1 | 331.07142846690584 | 973.948504484453 | 825.4237586312004 | 548.2804013664547 | 357.3392623889292 |
| NID2 | 190.46832957992572 | 591.8737880322125 | 552.1977891327809 | 343.3159202947127 | 218.61093161612857 |
| NTN1 | 105.39605244989086 | 187.8544820494332 | 162.35038150642572 | 130.0117698237395 | 87.69999056196447 |
| SLURP1 | 177.75025572864126 | 149.18731392734108 | 143.95196466304617 | 154.45934343095658 | 193.0519578542642 |
| SPARC | 18.701826287103973 | 16.017239930975112 | 17.217856827168127 | 16.79979245875521 | 19.07634310969221 |
| SPOCK4 | 19.74966371345726 | 20.78590901253726 | 18.98894120682568 | 20.06174661337061 | 19.946383908619218 |
### Chart: ADU
| Category | BL | 2 | 1 | 3 | 5 |
|---|---|---|---|---|---|
| COL1A1 | 2427.7903269010667 | 2979.9041320951637 | 3178.5975091043238 | 2676.5125954883124 | 2010.7217955475446 |
| COL1A2 | 2869.5310850893425 | 3791.6757946131393 | 4133.160950508653 | 3493.827202179723 | 2493.55496298308 |
| COL3A1 | 1593.2447451982382 | 2537.811429215226 | 2619.7738516758272 | 2098.6610143941493 | 1563.0465180131591 |
| CRNN | 4045.3897713014126 | 3455.9335166824058 | 3669.917366005937 | 4251.964119357565 | 4694.350572854057 |
| DMKN | 744.6263535147132 | 353.8988815237041 | 284.1963495057587 | 386.3287030761826 | 418.55943097926183 |
| KRT1 | 1023.1580478377824 | 262.63892105466323 | 117.46071089463791 | 248.68651794044504 | 276.87993721489744 |
| KRT10 | 2094.73769752689 | 453.99117150066013 | 387.0283904341461 | 501.14705299267916 | 486.1399666832549 |
| KRT13 | 3401.019533618788 | 3539.382360124429 | 3688.82409482373 | 4035.972475326638 | 4178.135658248655 |
| KRT14 | 5167.792269257271 | 3261.3762497819753 | 3069.9488747504706 | 3487.225964424519 | 3281.72707653044 |
| KRT17 | 1027.0203320789801 | 786.0527702968213 | 674.5222994832211 | 598.5296932976516 | 457.15883412172843 |
| KRT19 | 964.4996394940982 | 1077.936715651433 | 1092.1702791224366 | 1202.8028343158867 | 1330.6703467256157 |
| KRT23 | 677.8505912904402 | 583.6193794026874 | 614.7384540708445 | 828.7667281391492 | 901.6957640992729 |
| KRT24 | 4282.807386443663 | 1560.9237404228043 | 968.5420465634262 | 1783.9164369334658 | 2127.5407901310605 |
| KRT3/6B | 14409.618141375868 | 10884.616602653065 | 10188.394677535558 | 11924.296515560807 | 11950.409090600915 |
| KRT4 | 10913.834365203726 | 11179.115388509448 | 11460.654941415858 | 11666.823166766268 | 12166.33056745413 |
| KRT5 | 10830.930789337355 | 9744.566778561733 | 9686.083662039833 | 9833.948076398028 | 9898.217555181822 |
| KRT76/2B | 9495.590257034935 | 3472.0059513537926 | 2333.7485916554756 | 3617.1258201506544 | 4998.861222219864 |
| KRT78 | 1946.6361916149103 | 1574.4401653201676 | 2059.0701736003693 | 2692.6933099255434 | 3024.3500425353286 |
| KRT80 | 481.0179109561464 | 311.307031668644 | 308.16840694714915 | 414.12759074521205 | 479.6487203433919 |
| LAD1 | 1455.6041384081673 | 1123.216936941945 | 1181.2868907690097 | 1297.5191808432403 | 1460.9272898383456 |
| LAMA3 | 591.075974328446 | 508.79115607166864 | 431.4266794410384 | 453.0436756486569 | 429.18160654407285 |
| LAMC1 | 429.2832112879345 | 522.1042443375654 | 497.0734552974231 | 421.855348342742 | 356.1551165645256 |
| LCE1C | 856.9950809894505 | 304.79935527092545 | 268.440463800183 | 309.8753784919643 | 374.81839954035127 |
| LCE2D | 1426.5396552531734 | 420.75073374359795 | 389.7002378622245 | 448.9322579911565 | 675.4810083386674 |
| LCE3B | 687.9455359334327 | 232.3869534125593 | 224.12859570813507 | 244.4940256661587 | 353.3686016032571 |
| LCE3C | 2328.9985856057165 | 199.06155078050725 | 147.8133920409376 | 521.556353338055 | 648.9186292605633 |
| LCE3D | 7740.388750176311 | 2541.1792710370805 | 1623.0599004074973 | 4057.487043051738 | 5292.611507520386 |
| ODAM | 1921.253769391577 | 844.5060574837643 | 876.0069139230759 | 980.3607680554136 | 630.0901181043724 |
| POF1B | 2291.0300187673865 | 1287.1658519772038 | 1186.0221009703698 | 1668.4004665344428 | 1814.102796656508 |
| RPTN | 7827.676634426229 | 4048.1604938930122 | 2596.342659761175 | 5279.247293094851 | 7212.781404438817 |
| VCAN | 1166.0522811654696 | 1565.709690976513 | 1284.1440011595887 | 1042.3864961780434 | 698.3991265738753 |
| VIM | 1575.4325404273832 | 2207.4717813037346 | 2142.4535003844267 | 1812.6929733723464 | 1395.316814649054 |Normalized Expression
### Chart: AG
| Category | BL | 2 | 1 | 3 | 5 |
|---|---|---|---|---|---|
| COL1A1 | 2338.837982721637 | 3275.0039898460445 | 2646.2655954315765 | 2581.8576919436164 | 2869.3251729584144 |
| COL1A2 | 2636.432820649933 | 4122.435044845359 | 2934.8359717632748 | 2880.238549030332 | 3086.219173006107 |
| COL3A1 | 1445.0556556173287 | 2715.5873901788364 | 1903.0405446073805 | 1796.963778106594 | 1705.314081624032 |
| CRNN | 4681.800454738988 | 4212.064493855801 | 4272.144783047255 | 4101.558138519539 | 4512.371072776473 |
| DMKN | 770.8617295501577 | 330.43480905190137 | 443.54829485777384 | 338.94733731232736 | 457.01416325395815 |
| KRT1 | 1137.9284794423984 | 63.78483348563006 | 101.18279509978174 | 119.54079303320901 | 290.63386174545525 |
| KRT10 | 2174.3610603554903 | 220.7384370489336 | 269.8549571034137 | 347.9781085069862 | 503.7938901647948 |
| KRT13 | 3216.349971603641 | 3513.505644247459 | 3361.597663231054 | 3546.9033288323553 | 3964.1555294319533 |
| KRT14 | 5097.987262426656 | 3404.308938287378 | 3330.098559035472 | 3179.916892475733 | 3697.5187700259567 |
| KRT17 | 1104.8588109930101 | 764.7111595131811 | 749.665295851595 | 653.1430727733273 | 459.83844250055324 |
| KRT19 | 1139.5656203196304 | 1223.4249284038553 | 1161.7911056747282 | 1206.989337409269 | 1374.6048631073954 |
| KRT23 | 817.8250382265965 | 689.002218719649 | 844.4011095274147 | 792.8529505039845 | 822.9026470220147 |
| KRT24 | 3338.5356220108074 | 1182.1353264872605 | 1471.0105403923956 | 1418.4167189880009 | 2792.59723287913 |
| KRT3/6B | 14273.82294419628 | 11837.412777961059 | 10922.38188120099 | 11175.749899841308 | 12404.9831995099 |
| KRT4 | 10393.773698528508 | 11041.905265459789 | 10772.303903329122 | 11086.969913863335 | 11196.567392564428 |
| KRT5 | 9819.785489136462 | 9534.184811884901 | 8858.181328913866 | 9592.201224655619 | 9730.672984905164 |
| KRT76/2B | 9191.63798518651 | 2391.401883659258 | 3114.2413950272967 | 3956.3412333791257 | 6194.893124748416 |
| KRT78 | 2471.9614200381716 | 2153.0618783055324 | 2232.662468967408 | 2148.1549038299836 | 2298.779122858567 |
| KRT80 | 501.22924883166786 | 313.46163514790044 | 345.5110324270406 | 314.2820975742258 | 423.6427483590982 |
| LAD1 | 1413.8787212413588 | 1304.4735399698957 | 1277.3680314362953 | 1271.522338959418 | 1433.3350531741464 |
| LAMA3 | 506.59488411014274 | 500.3466623036728 | 434.0965403853171 | 440.54444884259897 | 489.7577687405224 |
| LAMC1 | 423.4280890356415 | 570.8758990040717 | 477.9105749548347 | 450.7714937434089 | 416.92917646655724 |
| LCE1C | 470.29637797782055 | 270.6580578444196 | 294.83533983645185 | 244.00409107153394 | 271.28528163953814 |
| LCE2D | 793.1670730195601 | 343.4003276819982 | 383.7497231793328 | 324.02808132447325 | 433.8614864586249 |
| LCE3B | 512.1957015061264 | 210.10178940841416 | 282.12196459390725 | 245.30355493979354 | 322.8905045720611 |
| LCE3C | 1551.5341199397653 | 144.3793119789212 | 380.9974951652516 | 188.35057321332667 | 531.5855760571379 |
| LCE3D | 7045.47161975766 | 1889.755604113052 | 3322.723373816404 | 2419.2889892918556 | 4072.0959898113965 |
| ODAM | 952.1367444822796 | 545.2188762270381 | 691.0639888255014 | 690.858545717118 | 859.6236029509167 |
| POF1B | 2174.1400271208436 | 1083.3107887476347 | 1217.6081166514791 | 1155.6380622113352 | 1451.8077184659332 |
| RPTN | 6101.709695835388 | 2880.938122576027 | 3123.558585664826 | 3633.6978505487227 | 5176.496889098715 |
| VCAN | 967.7440740520386 | 1268.81906982716 | 846.5584402526679 | 850.7101194561814 | 1244.7903115905226 |
| VIM | 1638.5928077981412 | 2156.0169541656046 | 1846.1820120627162 | 1885.1541832484775 | 1603.590937952122 |
### Chart: AG
| Category | BL | 2 | 1 | 3 | 5 |
|---|---|---|---|---|---|
| COL5A1 | 59.72257428457081 | 89.11707489269554 | 74.71352251838103 | 71.21664180820602 | 68.00514731279745 |
| COL5A2 | 134.3162062399779 | 244.91194337417392 | 189.38284969485147 | 178.92298828774884 | 161.22577939035034 |
| COL7A1 | 114.28671547644434 | 179.07311339694934 | 142.71663526727238 | 121.0486941503546 | 117.92747387949397 |
| CRISPLD2 | 270.5407182877975 | 644.2274580438065 | 377.109905697444 | 394.78911526892614 | 313.5725531955136 |
| FBLN5 | 168.52444089728002 | 251.0757602672904 | 183.79020092248854 | 187.3990187898475 | 171.53481338780426 |
| FBN1 | 426.92460329938945 | 870.0457986690419 | 599.0284671828501 | 603.8315015880567 | 478.4514830072359 |
| FLG2 | 36.4232152290696 | 21.441429718085857 | 21.99569375247823 | 18.223763379771626 | 21.366314200890354 |
| HSPG2 | 325.08599086644307 | 601.861499604544 | 466.9310445149076 | 441.76831748416066 | 380.1620147918773 |
| IFFO2 | 31.01809694399133 | 28.225281717531217 | 35.00351018578656 | 28.938003395302857 | 32.8111750496449 |
| KRT18 | 215.55505495758786 | 234.09173962165616 | 208.40629302096585 | 219.38745566359339 | 230.98608903212275 |
| KRT2 | 207.9873434771444 | 35.21812618509665 | 36.47656039257603 | 42.44468877344897 | 63.99757096165786 |
| KRT20 | 45.81413107459708 | 32.64495491691201 | 31.3055428765418 | 29.310010029061637 | 38.534414945311795 |
| KRT25 | 22.887840996737363 | 22.61205038572347 | 28.054540304872887 | 22.006103103663115 | 25.788016360503832 |
| KRT27 | 63.50727255823228 | 45.11027671199513 | 62.98086415661019 | 43.14203291375455 | 47.77128534096147 |
| KRT7 | 108.04225885050315 | 129.7640232962418 | 128.95684130137008 | 113.60470596345884 | 102.79251195595191 |
| KRT74 | 47.992268876540635 | 48.063710353327764 | 51.41380351243082 | 45.00158657614966 | 48.89175306862151 |
| KRT75 | 220.0762267829461 | 83.70180977336229 | 106.02283973900067 | 65.17548659235158 | 94.14114043935736 |
| KRT8 | 88.96612649236125 | 82.24749123214492 | 83.93799439022641 | 73.8404926889386 | 79.29501584996136 |
| KRT9 | 32.43033224242946 | 30.306361708369472 | 33.854526936636326 | 32.826142935549015 | 28.99605831757808 |
| LAMA4 | 313.4833723423831 | 812.7913813998944 | 556.0374468656898 | 513.5962733865421 | 390.33003183065637 |
| LAMA5 | 321.8695078572733 | 376.83533141814246 | 324.04485833033607 | 329.01072695509015 | 338.24447469610124 |
| LAMB4 | 168.9274275775689 | 77.92380597808257 | 72.29306752046445 | 81.03470512195898 | 106.45770353071353 |
| LAMC2 | 297.56661070690905 | 547.1972204147286 | 333.2180998637478 | 306.02801481212487 | 238.1282525875807 |
| LCE2A | 77.77781548072518 | 56.16867165974978 | 59.211473196665814 | 57.930276044857266 | 60.67949595331177 |
| LCE5A | 115.57484615031542 | 50.45969201451989 | 70.74946728782953 | 52.601638683060656 | 68.58953801317418 |
| NID1 | 387.28651578349456 | 1115.046157675329 | 712.68429403204 | 625.0399924616603 | 443.26658020225574 |
| NID2 | 232.72409350284565 | 599.4542652252895 | 444.213933209296 | 377.2899056972922 | 263.9766999793181 |
| NTN1 | 112.15404625515342 | 230.81595993106964 | 157.73677937205784 | 140.98264633725216 | 94.68606076695953 |
| SLURP1 | 141.58508430398777 | 130.38739609321104 | 130.37040011144913 | 135.38531665304737 | 132.1087799839642 |
| SPARC | 18.263333195945332 | 17.88117818607016 | 20.41818949445993 | 18.900039437414883 | 18.36334403278329 |
| SPOCK4 | 17.706298783811448 | 16.75593319085935 | 20.781907466369763 | 16.966909294287106 | 18.859001229385026 |Normalized Expression

## Slide 10
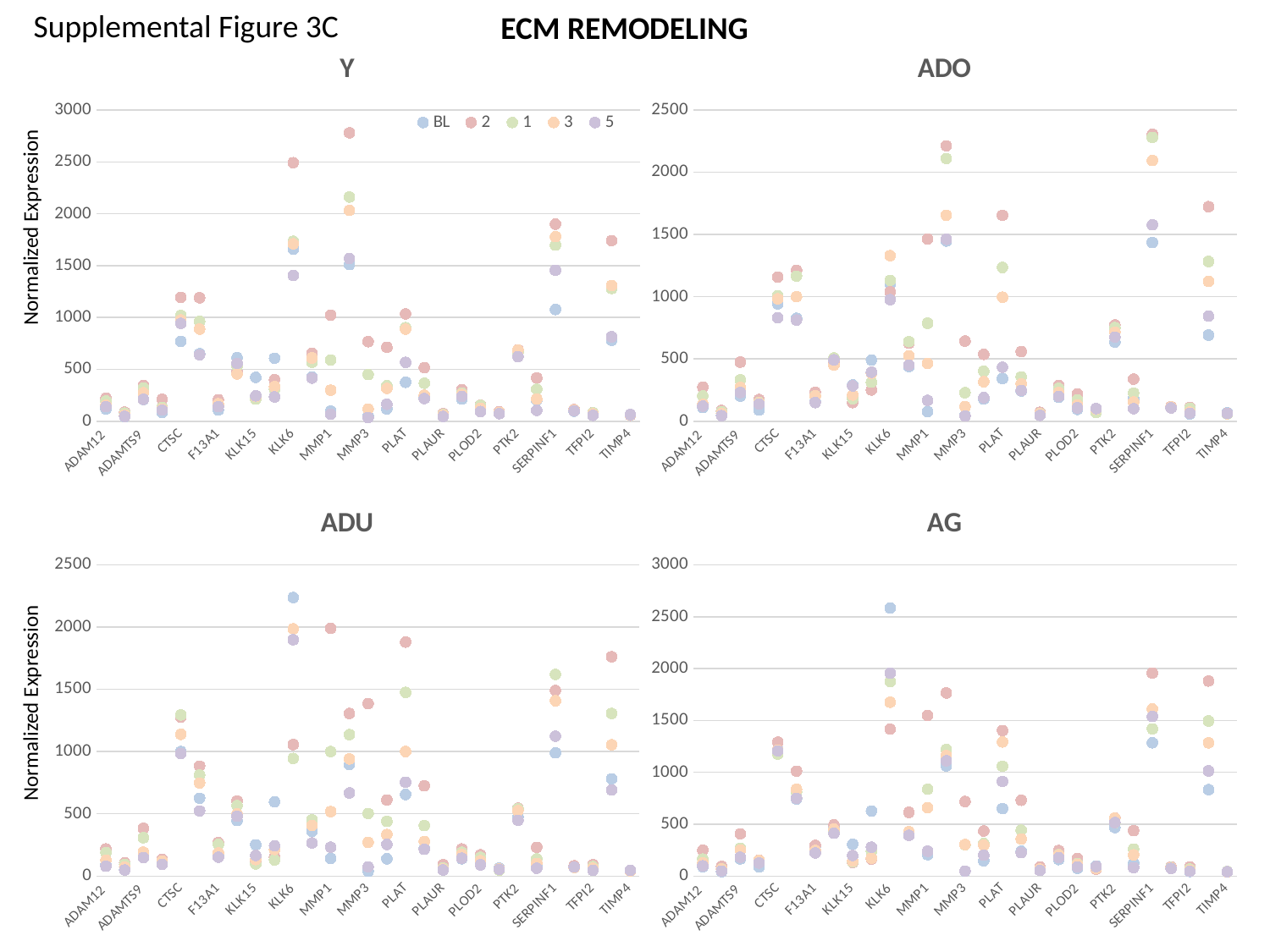

Supplemental Figure 3C
ECM REMODELING
### Chart: Y
| Category | BL | 2 | 1 | 3 | 5 |
|---|---|---|---|---|---|
| ADAM12 | 117.26986132480975 | 221.75166373572344 | 195.28452447718908 | 154.02337486709945 | 139.8028211474698 |
| ADAMTS6 | 43.25309579556711 | 87.9044747926551 | 77.81284591208117 | 60.44798423642151 | 46.71849153379204 |
| ADAMTS9 | 209.50834123293254 | 344.9830888409217 | 319.56145116070616 | 273.7166199461364 | 211.09623848338353 |
| CHI3L1 | 84.4798981490021 | 210.40045799614742 | 133.01028244850028 | 116.72378520508629 | 105.96667412293216 |
| CTSC | 769.7813277911408 | 1193.747437656083 | 1017.8519615277198 | 975.1846487903562 | 942.6171725235594 |
| CTSK | 650.4621059307549 | 1189.0717763712157 | 961.8786137981297 | 887.5805543454265 | 639.8892431381585 |
| F13A1 | 108.00454084919309 | 206.77483315486268 | 155.49154797215678 | 168.18006056014582 | 139.89796489432416 |
| F3 | 612.5982126926879 | 482.24780501404143 | 533.1580559292528 | 455.66945599444836 | 556.9567458894267 |
| KLK15 | 422.4421036412999 | 214.08959866147842 | 212.5581232242322 | 238.7687713099614 | 244.5628409174227 |
| KLK5 | 606.9356274794877 | 401.0889205788627 | 307.8055337550286 | 333.76708374223085 | 233.999431297284 |
| KLK6 | 1659.2680301420964 | 2492.0147285313137 | 1734.249410042445 | 1709.726866809934 | 1404.8497647361428 |
| LOX | 425.8686690668709 | 654.4265337073032 | 567.675935257188 | 609.398191665909 | 415.44348568163764 |
| MMP1 | 96.62984747478843 | 1022.7397795490514 | 590.0532013132088 | 298.9644604688662 | 67.58163712041845 |
| MMP2 | 1509.0645245558946 | 2779.847764289947 | 2161.220545423969 | 2031.9023027626108 | 1568.9424428689022 |
| MMP3 | 50.37205649977378 | 766.3682893708184 | 449.26540297920087 | 116.22017498733648 | 34.59115179633837 |
| MMP9 | 118.63462667433912 | 712.0160318050167 | 342.0691733596587 | 315.8467761204864 | 163.01802830756412 |
| PLAT | 375.11353369131825 | 1033.9371613856165 | 901.735008310358 | 886.725433408177 | 567.2702196130667 |
| PLAU | 237.0340286055073 | 516.2211306192455 | 366.32930175997353 | 250.74686967738384 | 218.0933364330549 |
| PLAUR | 60.35886629066805 | 72.79095926427769 | 64.93041904119299 | 60.57257855639255 | 45.69056548981831 |
| PLOD1 | 212.01979470184605 | 304.32269597935846 | 272.0545605708583 | 258.04503839319455 | 238.23324437650703 |
| PLOD2 | 97.50820346247251 | 154.39238805184297 | 152.14368510847547 | 123.80237499309897 | 93.46248614839818 |
| PRELP | 84.74176169464171 | 91.69484347481236 | 79.47235927982041 | 85.66432399700602 | 72.91681253930798 |
| PTK2 | 624.8194491815098 | 688.4128662494106 | 659.9888467820338 | 685.1328894768326 | 621.8660466655597 |
| SERPINE1 | 200.95637039741624 | 417.258452373654 | 308.48215370495024 | 213.5524769424784 | 103.99846035552447 |
| SERPINF1 | 1076.358821313422 | 1899.7893143279232 | 1697.9604648643567 | 1779.268271888895 | 1454.3693207618564 |
| SERPINF2 | 95.89357963274094 | 112.05751587714423 | 104.75007191732594 | 108.92613632655973 | 100.22604319983849 |
| TFPI2 | 62.29456382586861 | 82.81228117456996 | 80.21517702299955 | 69.87274116842526 | 56.38171536245272 |
| TIMP1 | 780.0529854288737 | 1741.3541275608545 | 1279.0615455161917 | 1307.9901555048566 | 813.5403400236113 |
| TIMP4 | 63.762017601322526 | 57.23036859292219 | 58.41064350432733 | 60.00905236899533 | 63.55133103196235 |
### Chart: ADO
| Category | BL | 2 | 1 | 3 | 5 |
|---|---|---|---|---|---|
| ADAM12 | 109.60211238271872 | 273.47077421048175 | 202.99044600550928 | 135.6577631338963 | 121.62206717274161 |
| ADAMTS6 | 50.07421549399983 | 86.09427409389679 | 77.40545301888068 | 62.24071275392728 | 44.40555722880853 |
| ADAMTS9 | 201.35972678305455 | 474.49251229088907 | 332.6841617417805 | 271.3047126679533 | 229.9383449293134 |
| CHI3L1 | 88.60724357771251 | 173.6020252863821 | 152.41616239410007 | 146.71229104272933 | 137.63796724474707 |
| CTSC | 942.1555046429572 | 1158.2579433291608 | 1008.1044541476052 | 982.9596966400859 | 830.6991736244267 |
| CTSK | 826.3202316903933 | 1211.7715119992477 | 1165.2268442370898 | 1000.26184722048 | 811.9882452781517 |
| F13A1 | 154.48330860834568 | 233.225360926252 | 205.62407995415288 | 203.0806728389283 | 149.61522352048155 |
| F3 | 480.7035266420344 | 483.3762462458048 | 507.3534431058663 | 452.04934896062673 | 492.8926329219141 |
| KLK15 | 291.7755242495026 | 149.17664867781227 | 180.02418228135411 | 210.2109886427205 | 283.24802835061786 |
| KLK5 | 491.5145218097442 | 251.00037030234913 | 312.9729212980247 | 384.1590796128214 | 393.4821443213161 |
| KLK6 | 1102.691870467951 | 1038.4669308715631 | 1131.046951754068 | 1330.2862170334706 | 976.525617028711 |
| LOX | 438.6372298143899 | 625.7434726385005 | 638.8844225691755 | 526.5258587961623 | 449.5690582364866 |
| MMP1 | 77.34742447341783 | 1463.9988751832707 | 786.6843857439194 | 463.8701604589685 | 167.3080781122325 |
| MMP2 | 1447.4977992066024 | 2212.4599046090575 | 2110.239233295333 | 1654.319151453642 | 1461.6014774494456 |
| MMP3 | 45.0366760983482 | 643.1942140695747 | 228.82827725464136 | 117.04538845697581 | 41.24604356515857 |
| MMP9 | 178.59929460459705 | 536.4707552762213 | 401.48490050383407 | 316.77388123345406 | 189.39515051272818 |
| PLAT | 342.38257221059376 | 1653.9678424746128 | 1234.5413036747757 | 995.9316793297332 | 433.8824897944397 |
| PLAU | 242.53836099323533 | 558.8544590157264 | 356.12394877230207 | 299.6371431739895 | 246.69696515981175 |
| PLAUR | 57.06166495574713 | 71.48818386070226 | 62.53447801519329 | 59.2938493933285 | 47.249456510368375 |
| PLOD1 | 190.6697433391846 | 287.8872989841807 | 263.1397971357686 | 229.92039050301284 | 199.91037823802148 |
| PLOD2 | 94.9001437485344 | 220.20511508520292 | 171.25457509057026 | 130.05944666314434 | 107.26945562884627 |
| PRELP | 82.30031776968241 | 70.9445963085696 | 68.45961305182097 | 96.0001806104023 | 101.53188841811635 |
| PTK2 | 634.7865343212618 | 772.4720349830845 | 751.5650666910394 | 714.4953726605822 | 676.0394998729266 |
| SERPINE1 | 183.41556465859932 | 337.57216359354743 | 225.98195934994166 | 154.9547031733283 | 100.77655929112413 |
| SERPINF1 | 1435.924575592449 | 2305.1182301763465 | 2280.02708276487 | 2094.175680945277 | 1577.9216861532652 |
| SERPINF2 | 108.25652163753153 | 116.52142047997157 | 109.99188888988459 | 114.41345456264827 | 106.76453097939914 |
| TFPI2 | 57.253268825670744 | 111.63742290266634 | 104.16260701094859 | 73.14566689118166 | 64.09131994211113 |
| TIMP1 | 691.4762954630919 | 1723.3485917945964 | 1283.225026277234 | 1122.0856481734954 | 843.2706041975686 |
| TIMP4 | 68.32918451257322 | 62.15814842532927 | 58.296877759951904 | 60.81606474854372 | 61.5096970572521 |Normalized Expression
### Chart: ADU
| Category | BL | 2 | 1 | 3 | 5 |
|---|---|---|---|---|---|
| ADAM12 | 90.66401392491814 | 217.592598889111 | 189.20611262774443 | 127.2701974300043 | 79.2488774489309 |
| ADAMTS6 | 49.50324828016671 | 107.0093254582155 | 94.79712208002508 | 72.09353627851704 | 48.49719464207945 |
| ADAMTS9 | 165.8565891831708 | 383.34925415908106 | 308.047335233094 | 191.98407803744482 | 149.0027313830516 |
| CHI3L1 | 103.10261317440215 | 134.13483369696766 | 101.5630904914709 | 120.00087402137109 | 94.99203820265593 |
| CTSC | 999.8989732608292 | 1276.3531421900943 | 1294.3255697171512 | 1138.4174875271337 | 985.2332353227373 |
| CTSK | 624.7412711935737 | 883.1626087205267 | 811.6317263354634 | 747.5393701549345 | 523.0649876039685 |
| F13A1 | 161.34969856943235 | 269.13667028067266 | 254.60786326450523 | 185.80048467770092 | 151.5815371223086 |
| F3 | 445.9455650404562 | 603.1727318209146 | 565.4462918636078 | 499.15260033214395 | 480.7773914368071 |
| KLK15 | 250.92453710600546 | 107.72272244512129 | 97.27238140610608 | 132.20301735233278 | 165.42898010368273 |
| KLK5 | 596.3307789009033 | 154.12500429363433 | 129.84870395316602 | 213.38350686147214 | 243.61692958886428 |
| KLK6 | 2236.999568992257 | 1056.0790408541989 | 944.9318960403739 | 1985.778215641737 | 1898.575958817194 |
| LOX | 358.65909312131885 | 451.29390426651446 | 451.88123927106204 | 405.41546592201234 | 266.00321367864956 |
| MMP1 | 142.0351034382368 | 1989.3327980445074 | 999.8167831252259 | 517.6427119054147 | 231.66960186078848 |
| MMP2 | 895.6298137429154 | 1305.9368465578552 | 1135.966876619932 | 941.109270704748 | 667.6983065062354 |
| MMP3 | 38.40810421438795 | 1385.71187724753 | 502.6522444728129 | 270.4441912315823 | 74.13863965063024 |
| MMP9 | 138.86902821481996 | 611.1940423925731 | 439.73247350095534 | 333.2692875664932 | 254.46062001077877 |
| PLAT | 655.2735747045276 | 1879.709753710266 | 1475.7382458553564 | 1000.0334772673959 | 753.7061841971998 |
| PLAU | 221.51041406919404 | 724.8433066738385 | 405.3036137388835 | 277.8554507323199 | 214.85931648977956 |
| PLAUR | 56.59911942385499 | 90.31653174497434 | 64.81153078087746 | 55.75965741425994 | 48.9007498898212 |
| PLOD1 | 139.12281758779716 | 217.7918245410755 | 190.99834516225923 | 172.92136529671183 | 143.76438875347054 |
| PLOD2 | 96.19369856010726 | 170.7840360268394 | 150.12965815568566 | 122.96477877469354 | 90.40438916808571 |
| PRELP | 63.385596705600925 | 49.54500137560013 | 47.228116414123875 | 57.186320687133694 | 53.849955784341155 |
| PTK2 | 472.2276179332156 | 544.9197067501525 | 540.4291837943391 | 524.7306160186379 | 447.7235472447494 |
| SERPINE1 | 90.00298149281788 | 230.61151220247564 | 137.207979860445 | 101.49214011702685 | 62.60348387559355 |
| SERPINF1 | 988.9326281104586 | 1491.2959549840525 | 1619.6091122952628 | 1407.8602267481683 | 1123.4248710077231 |
| SERPINF2 | 69.23855481710925 | 82.24252355659222 | 72.08936703059187 | 69.28946331052435 | 71.59710988886211 |
| TFPI2 | 52.05275611026975 | 90.77819994805171 | 75.06645862579335 | 63.31850221567337 | 46.30697803578574 |
| TIMP1 | 781.1386072155013 | 1761.882656311244 | 1306.4443705573049 | 1054.2013894164925 | 691.0942994448591 |
| TIMP4 | 46.446423540749436 | 44.702607634499635 | 43.24050911194345 | 41.70159908478404 | 47.29872158458142 |
### Chart: AG
| Category | BL | 2 | 1 | 3 | 5 |
|---|---|---|---|---|---|
| ADAM12 | 89.65325818804712 | 248.9174299018035 | 163.86923019279234 | 126.62214406031997 | 98.4176559471731 |
| ADAMTS6 | 42.66817782394188 | 92.24518948461922 | 69.66543473043343 | 67.49360279605156 | 48.938214497614645 |
| ADAMTS9 | 165.79532631698956 | 407.70340310757035 | 268.29271871171755 | 249.90903363990427 | 181.52021041185367 |
| CHI3L1 | 86.61629617699673 | 151.92457082084204 | 145.46382613500415 | 151.16272303341648 | 126.09622549223026 |
| CTSC | 1217.219318299005 | 1290.7990964678402 | 1175.3042870952693 | 1213.2250674988836 | 1206.8824118647374 |
| CTSK | 741.937249603693 | 1011.2578402883812 | 808.456175689936 | 836.9985849837933 | 749.7723748144597 |
| F13A1 | 283.787297111373 | 296.70371657198234 | 239.6038064726589 | 246.13608971136057 | 222.3633179939733 |
| F3 | 467.7902335725347 | 494.34540135379393 | 428.65197312668806 | 453.5972452024255 | 412.62697618689833 |
| KLK15 | 306.6638442413401 | 131.2960822454587 | 136.5194020409561 | 144.59850212278602 | 197.9639236079913 |
| KLK5 | 627.0749209584052 | 162.5470508297291 | 239.2009259619212 | 172.3100588256152 | 278.3121618657447 |
| KLK6 | 2582.6099930460987 | 1415.5896981113435 | 1874.6106807649296 | 1675.8790469947926 | 1955.2995483129291 |
| LOX | 407.54116381804187 | 613.8929554680473 | 392.57163408021523 | 426.81463747206664 | 392.29658976044044 |
| MMP1 | 206.34113289243714 | 1547.8014847925372 | 836.2528905819393 | 658.8721753612239 | 239.27549933756563 |
| MMP2 | 1061.5827061552518 | 1765.0572107013586 | 1220.1921683182964 | 1161.093667011834 | 1110.5322010300717 |
| MMP3 | 47.513141981464 | 718.6711583075452 | 302.61150651600275 | 304.08953732927455 | 48.29967712891405 |
| MMP9 | 147.7657509113516 | 433.8763284572014 | 313.32899154377424 | 297.7003360399209 | 201.28404459445133 |
| PLAT | 650.4809042418698 | 1402.6782658283819 | 1058.6236031245685 | 1292.5313544354608 | 912.1096429297015 |
| PLAU | 239.21613261258977 | 730.1185581761666 | 443.03314808037453 | 356.45247407363524 | 225.3543395727118 |
| PLAUR | 58.45880118377128 | 88.38232090595675 | 62.40135432577225 | 66.7350428863029 | 53.81207189285291 |
| PLOD1 | 159.17190831873853 | 245.8973589814915 | 206.39662782635185 | 203.88281764378362 | 178.00865367978653 |
| PLOD2 | 75.49543253922393 | 171.39609737141143 | 119.9605015278608 | 112.88153556608897 | 89.07887134877477 |
| PRELP | 97.68957974135515 | 65.98192189583364 | 77.78255233333411 | 74.88946195648771 | 91.90718574578148 |
| PTK2 | 467.0600292279606 | 558.8813288041911 | 514.5609435975981 | 558.249051034413 | 515.384965706853 |
| SERPINE1 | 122.71524075443057 | 438.09074525538495 | 259.9311727122821 | 204.3459804631299 | 81.1842335887957 |
| SERPINF1 | 1284.2936537356072 | 1956.8818266834367 | 1418.8628317105745 | 1610.2477976355124 | 1537.9501517622596 |
| SERPINF2 | 81.14986321969758 | 89.21469687263051 | 81.63721942535969 | 78.3566508960275 | 74.14452723012903 |
| TFPI2 | 44.16012466030815 | 88.97836351217292 | 67.5681051779579 | 56.81759818742882 | 46.19332168288765 |
| TIMP1 | 832.5201887864905 | 1880.3869865986821 | 1494.4101383963452 | 1284.208344655424 | 1013.1929885626355 |
| TIMP4 | 39.53509170861136 | 41.1225472711577 | 43.71353863950238 | 38.02698337282811 | 41.14515525164175 |Normalized Expression

## Slide 11
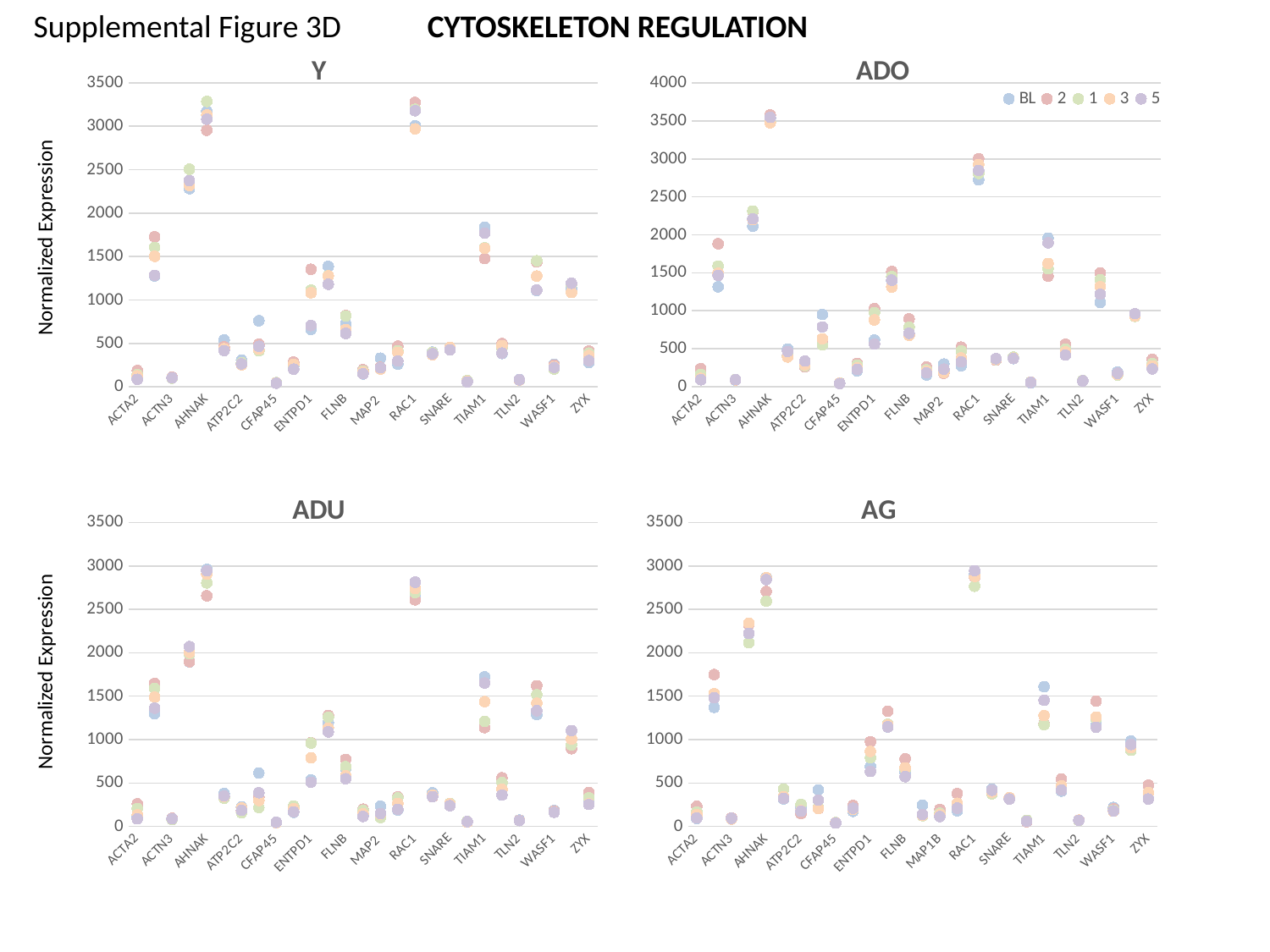

Supplemental Figure 3D
CYTOSKELETON REGULATION
### Chart: Y
| Category | BL | 2 | 1 | 3 | 5 |
|---|---|---|---|---|---|
| ACTA2 | 96.02444081696682 | 186.0510117910485 | 139.35688724132615 | 125.72097740500787 | 85.79910721816815 |
| ACTN1 | 1276.8410537181835 | 1727.2366685733339 | 1606.021730032108 | 1502.535925343984 | 1283.8688641820222 |
| ACTN3 | 100.91383441100979 | 110.06727549642982 | 98.15545110291751 | 104.19555465494588 | 103.06966425865703 |
| ACTN4 | 2282.216015058467 | 2333.6329191706127 | 2505.7109452312557 | 2317.3259030056324 | 2375.477586060978 |
| AHNAK | 3172.7429264095176 | 2954.8729427473645 | 3286.37693947302 | 3131.1332453515365 | 3082.6485890218337 |
| ATP2C1 | 540.1733762684552 | 459.79310331544934 | 419.4706281754851 | 438.1071797847661 | 416.2223473240936 |
| ATP2C2 | 307.83431920491705 | 255.07861478441225 | 278.90572405997983 | 253.37365401091964 | 264.0436708569181 |
| CALML5 | 759.6453379535424 | 490.61853441750617 | 413.53511717954746 | 433.0004462090755 | 465.8534795192435 |
| CFAP45 | 44.72349181054662 | 44.48785498001152 | 47.50796647953407 | 45.202228923073676 | 41.12155626554125 |
| DNM1 | 235.3694333050849 | 284.4226356689515 | 248.42742927945812 | 259.26563188721116 | 203.43257922277087 |
| ENTPD1 | 660.9039467098914 | 1352.7790211956974 | 1113.2149050856567 | 1082.5819471436826 | 704.6863681320797 |
| FLNA | 1386.2509265903936 | 1279.1401484520395 | 1277.8874164031868 | 1276.2331758826679 | 1180.090124986176 |
| FLNB | 726.5221473655108 | 821.1870066859059 | 815.4487461835594 | 659.759456426847 | 614.6227188824498 |
| MAP1B | 147.86171858191426 | 196.7433366784357 | 180.8960999956422 | 168.67606568078696 | 149.71483825688262 |
| MAP2 | 329.2262778112086 | 224.27423806597494 | 209.33035964761675 | 203.55418479593942 | 218.43785488451093 |
| PDGFRB | 259.93260685283593 | 467.5890228775109 | 415.85127316689596 | 397.05687415104063 | 295.4594755368678 |
| RAC1 | 3006.574071178853 | 3274.4193599215864 | 3196.1848908911966 | 2970.641521186102 | 3179.247408443548 |
| SMURF1 | 398.78392461652317 | 385.45343470143115 | 395.7556956287535 | 368.8434419218976 | 379.8826380019052 |
| SNARE | 438.36583342392936 | 445.0770523813037 | 446.85957861439283 | 452.1079275637633 | 425.6914314965659 |
| TAGLN | 61.62585340238825 | 70.79976700201264 | 72.18253773927836 | 61.544561639942835 | 56.39069637699943 |
| TIAM1 | 1837.281285063799 | 1476.6193888137732 | 1601.4742280981302 | 1594.0013536680312 | 1769.4167200192348 |
| TLN1 | 382.61031756779784 | 496.44315024639764 | 446.69487074341447 | 473.9946549797644 | 387.9917894721483 |
| TLN2 | 81.70823040761579 | 78.12898261873585 | 76.45196202924637 | 79.05454812076306 | 80.67117431896166 |
| VCL | 1109.802678479261 | 1437.694566445097 | 1451.5520641200424 | 1275.1763928033133 | 1115.0498201865043 |
| WASF1 | 258.5918282138282 | 247.91486412208027 | 205.50634670744688 | 228.98575481464286 | 222.20217891027858 |
| WASL | 1132.7304230903187 | 1186.8586411532765 | 1178.9473409800212 | 1085.8765005047112 | 1191.4689991450837 |
| ZYX | 277.4419000242209 | 411.431251721667 | 388.0191499687335 | 359.7640021543497 | 300.79137666114235 |
### Chart: ADO
| Category | BL | 2 | 1 | 3 | 5 |
|---|---|---|---|---|---|
| ACTA2 | 106.23626216870649 | 238.37203720027594 | 163.41262335579384 | 109.95462290624039 | 92.3576643509325 |
| ACTN1 | 1315.7695600499499 | 1882.945360456545 | 1590.4029526879629 | 1495.352707264145 | 1465.922846578963 |
| ACTN3 | 88.9897482691132 | 90.84783283232571 | 96.00806922265026 | 92.00224842542606 | 92.97360878129751 |
| ACTN4 | 2115.436516460708 | 2309.784868095259 | 2314.8886011590957 | 2199.050322914367 | 2209.3000340490457 |
| AHNAK | 3529.3839568990397 | 3577.1438920237656 | 3492.0748523223256 | 3474.5481187213536 | 3545.6842159330718 |
| ATP2C1 | 497.8290259631548 | 408.1027781266508 | 399.9112244462308 | 392.4042456342647 | 464.8841950260799 |
| ATP2C2 | 295.8970544629326 | 261.6672947634648 | 276.08698680905906 | 289.90193024989065 | 337.8615968676355 |
| CALML5 | 951.595655443725 | 566.6978250330635 | 551.8901120222642 | 632.4063417130059 | 787.5731840228684 |
| CFAP45 | 44.57185442581477 | 49.30890622272774 | 48.65716160009089 | 47.28787447961536 | 42.09976501633767 |
| DNM1 | 210.3818723461808 | 308.4547357632479 | 286.2822122822106 | 245.97031220539722 | 228.3926782228556 |
| ENTPD1 | 615.0535885687485 | 1028.3864166187439 | 975.6491095033938 | 879.8422284887889 | 562.0805389782341 |
| FLNA | 1389.249004684331 | 1516.111152592387 | 1450.4640604699946 | 1311.6627445038619 | 1402.9766780838459 |
| FLNB | 687.9268346795111 | 893.3044555135459 | 783.9086933002309 | 676.7436803386762 | 704.312577335792 |
| MAP1B | 156.34330236017956 | 259.37796402849085 | 222.29792139809126 | 204.59248572263655 | 184.10621427822844 |
| MAP2 | 298.8140895804871 | 177.85472525204125 | 205.63212932122815 | 188.25750604035946 | 231.59407771129105 |
| PDGFRB | 276.573535431602 | 521.4168662374194 | 471.98893767852746 | 378.0917996588744 | 326.3880687998343 |
| RAC1 | 2724.0512053864913 | 3001.893699178703 | 2808.030396739265 | 2925.704044151947 | 2848.121071053292 |
| SMURF1 | 364.7466663527375 | 348.80204121316564 | 359.4626482478336 | 359.7413359334477 | 369.7569485252344 |
| SNARE | 370.65192325471895 | 385.1199065669107 | 389.64663189166964 | 376.3770346758888 | 371.80382426753727 |
| TAGLN | 52.04044009832555 | 59.80039252904766 | 63.70071502374188 | 60.575480679005764 | 58.1651528350665 |
| TIAM1 | 1957.164568623667 | 1454.8251270866103 | 1553.1036309243611 | 1624.2898394449924 | 1895.6476270402798 |
| TLN1 | 416.3658041252568 | 558.6208822074306 | 496.43667142558115 | 458.18955280325855 | 416.1790727839738 |
| TLN2 | 76.39087995680295 | 77.08156135399574 | 81.09711636161086 | 76.4046563152073 | 76.89136568366729 |
| VCL | 1108.9787107821198 | 1499.1360574951686 | 1409.557671312773 | 1317.029539368581 | 1217.5751145728934 |
| WASF1 | 193.6835302934968 | 175.09484136749097 | 155.37068065798724 | 165.02335135996768 | 177.77670752016007 |
| WASL | 944.4797971159034 | 937.7206621956557 | 926.1265363268192 | 930.0150170984479 | 961.9108648408813 |
| ZYX | 248.23141932746213 | 361.82552281694814 | 305.2134084100465 | 275.1531212866706 | 234.54618822830238 |Normalized Expression
### Chart: ADU
| Category | BL | 2 | 1 | 3 | 5 |
|---|---|---|---|---|---|
| ACTA2 | 105.03459374761185 | 259.3769982851173 | 207.38041515809496 | 132.78133308610646 | 87.08465519031259 |
| ACTN1 | 1297.9892272515972 | 1647.24612157392 | 1588.9254031824923 | 1489.07590332928 | 1361.162646792579 |
| ACTN3 | 82.29847294619009 | 95.03636903959764 | 78.982714697829 | 88.49253196594458 | 88.68405815093882 |
| ACTN4 | 1894.5789518128654 | 1897.5354270438402 | 1986.63917388387 | 2005.0120401550548 | 2070.5649191438806 |
| AHNAK | 2959.914183384894 | 2655.817524533892 | 2804.5422783302715 | 2904.853988274537 | 2944.209872833211 |
| ATP2C1 | 379.58748352779963 | 343.3865586481684 | 324.5137798586122 | 346.45545246288 | 350.85989498517915 |
| ATP2C2 | 225.58266176335596 | 180.92474256080567 | 156.71514138881471 | 211.48988665708762 | 184.4780592712783 |
| CALML5 | 614.4087292068557 | 337.3461068555347 | 216.34773865305553 | 298.2947722034532 | 387.6768930533257 |
| CFAP45 | 42.48927301627567 | 42.52593653227118 | 43.83026841892807 | 42.28831984017738 | 46.88068426850295 |
| DNM1 | 162.27240375838866 | 231.056463140198 | 234.4282426622077 | 204.35537194408204 | 167.36002330768827 |
| ENTPD1 | 534.1369093213298 | 964.4570916196504 | 959.2038122178233 | 789.9867151400389 | 509.13451795903046 |
| FLNA | 1198.5205303122987 | 1278.1760031750991 | 1257.3309461900237 | 1132.3476389500477 | 1087.8815935243063 |
| FLNB | 664.6274807340776 | 772.1924171169604 | 686.8511816755446 | 583.8418050682441 | 548.4989575451617 |
| MAP1B | 123.05898106692001 | 198.2972005108512 | 183.29179441102022 | 144.71063348183014 | 114.42001406442003 |
| MAP2 | 233.22200489526642 | 133.62221254806417 | 103.0231532430465 | 142.76087886653244 | 148.32932710643624 |
| PDGFRB | 187.44381291065181 | 339.0023230096683 | 328.77092126412407 | 263.78322430031585 | 196.81021667230095 |
| RAC1 | 2672.170486060262 | 2609.3686909096205 | 2693.0598268775716 | 2738.6458978162464 | 2812.942731662962 |
| SMURF1 | 386.1318098545478 | 342.2081717014263 | 344.84349966226995 | 360.3282811530504 | 343.9715095915677 |
| SNARE | 248.1060750610667 | 246.68748846604922 | 264.75841112188056 | 258.59397297978273 | 238.88797822941666 |
| TAGLN | 56.08631767266135 | 56.99638114755044 | 56.85845699940257 | 49.4610921845639 | 55.09847436481865 |
| TIAM1 | 1720.92440766701 | 1137.0680603973951 | 1208.9687460879036 | 1437.0150434875056 | 1652.1239433225232 |
| TLN1 | 360.86850233104576 | 559.2114013345962 | 503.8976582556076 | 426.8728524317387 | 361.833448331088 |
| TLN2 | 72.90350002423686 | 70.73769238504667 | 69.59743344296885 | 68.0592941327373 | 66.68104224678841 |
| VCL | 1287.257470478133 | 1620.6252612914463 | 1516.244667441451 | 1418.953918420676 | 1332.0988081348821 |
| WASF1 | 182.91242551945422 | 175.01252833152904 | 167.35870068176203 | 166.2720411029674 | 162.72338378291295 |
| WASL | 1009.5857488164719 | 894.4091583689658 | 938.9912135905761 | 1006.1971586591612 | 1103.0109122064741 |
| ZYX | 261.0087606150499 | 392.00754781672856 | 329.10323177837955 | 258.27544600482105 | 252.25510968244302 |
### Chart: AG
| Category | BL | 2 | 1 | 3 | 5 |
|---|---|---|---|---|---|
| ACTA2 | 91.80121013479717 | 231.24356905054935 | 166.25156253927827 | 136.94545552777814 | 95.5311217695542 |
| ACTN1 | 1370.4888220689045 | 1747.9653779495234 | 1483.1343332130964 | 1529.3380519054465 | 1480.6526986191086 |
| ACTN3 | 89.1085341236908 | 98.67566115584573 | 87.1185711583715 | 83.09069042566624 | 92.54010307511686 |
| ACTN4 | 2220.317348097713 | 2325.246144375704 | 2114.684095551689 | 2340.9709787507986 | 2221.8905640126886 |
| AHNAK | 2865.784746251864 | 2706.1833477778773 | 2594.182566418269 | 2864.739121022069 | 2842.0404525001213 |
| ATP2C1 | 316.0956011304117 | 335.9643500290804 | 428.0271274539572 | 340.99612925869354 | 320.6162237182999 |
| ATP2C2 | 215.21151151706204 | 147.2909324489438 | 251.50981520757333 | 180.63714844030898 | 174.0269922924251 |
| CALML5 | 419.85600846426973 | 203.58583101819582 | 216.95756443223195 | 204.83049279640645 | 304.0239304621759 |
| CFAP45 | 37.73117009253811 | 44.91250243279879 | 44.2110799122184 | 41.624058262414785 | 38.798339058077325 |
| DNM1 | 173.54336185779513 | 240.326083195707 | 204.87684600056264 | 195.63307797649512 | 209.67195810414864 |
| ENTPD1 | 688.9469142489381 | 975.356126420788 | 790.7156287179973 | 863.367902179618 | 633.1025155930615 |
| FLNA | 1145.4005194756778 | 1326.1677776815964 | 1176.353031646059 | 1166.567109091469 | 1146.2776293231218 |
| FLNB | 617.8184730866346 | 778.0385567069948 | 655.9084138463531 | 675.4016148831016 | 572.482000205577 |
| MAP2 | 244.11597121911825 | 129.57025507092044 | 123.29328819809676 | 129.37508782711802 | 138.2338562374431 |
| MAP1B | 115.55353686446693 | 195.1432992221227 | 152.0517813935558 | 134.3895208199053 | 113.42378397015518 |
| PDGFRB | 180.9659969043433 | 377.5424395066914 | 265.1069545728812 | 266.0600467864143 | 210.4367550419058 |
| RAC1 | 2904.3362807001067 | 2869.391499244997 | 2766.077657012025 | 2879.5101367387997 | 2944.9427307397345 |
| SMURF1 | 429.2814550665926 | 413.0875452782104 | 374.8547366755155 | 393.9299990763126 | 416.87750728276234 |
| SNARE | 323.2783651538319 | 316.22694481064474 | 324.6756943039742 | 330.36279525367416 | 315.0527143600832 |
| TAGLN | 59.06758978221506 | 53.30078654271041 | 71.14374606620017 | 59.591781879500935 | 57.690709913101486 |
| TIAM1 | 1609.0745066147701 | 1181.759243429241 | 1171.1397169050545 | 1275.0974827337175 | 1452.9115089704912 |
| TLN1 | 406.0639168929534 | 546.5299733726737 | 460.3067605228121 | 465.9513435148804 | 417.8758011445052 |
| TLN2 | 71.15728070762933 | 72.46834080266903 | 68.43653384960552 | 72.10512616807434 | 70.99124006935727 |
| VCL | 1165.2158375410331 | 1442.7267140546412 | 1236.897861127028 | 1260.204190913767 | 1141.7766537689977 |
| WASF1 | 217.7775844345992 | 202.32436810453393 | 188.07181282648105 | 173.94772524372706 | 178.8272827252986 |
| WASL | 983.697893447621 | 909.1316905226668 | 875.7229663880353 | 906.0700481442508 | 943.678161645087 |
| ZYX | 321.4273634477479 | 473.98531835546004 | 391.07843554404286 | 388.50728613429123 | 314.93719202546913 |Normalized Expression

## Slide 12
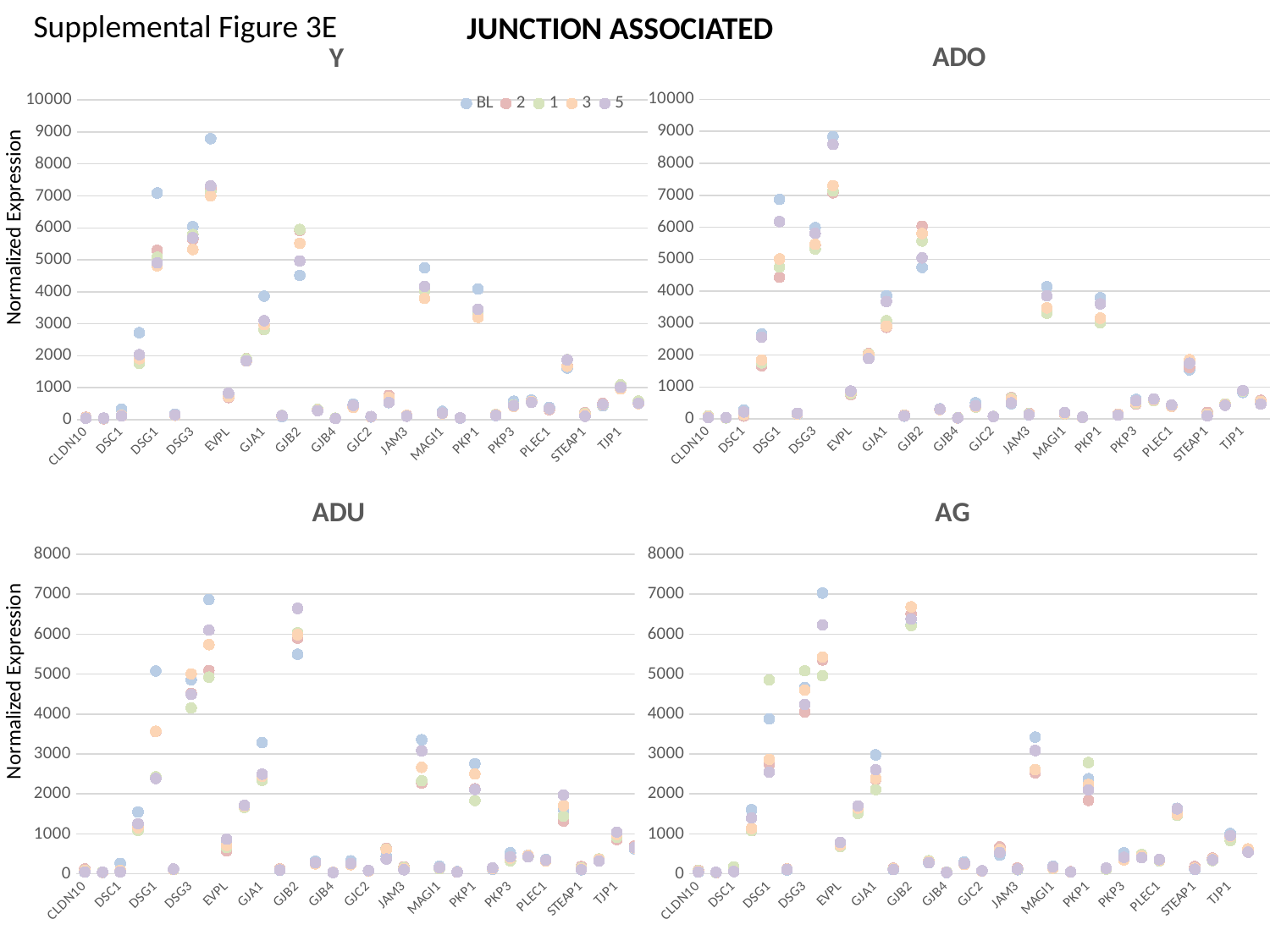

Supplemental Figure 3E
JUNCTION ASSOCIATED
### Chart: ADO
| Category | BL | 2 | 1 | 3 | 5 |
|---|---|---|---|---|---|
| CLDN10 | 43.75786896876971 | 97.87245715220917 | 88.22612701633929 | 65.66620078857545 | 44.620423201204524 |
| CLDN8 | 40.274582196925905 | 34.626007854549385 | 36.41496296736639 | 41.01327892094216 | 42.92067576910664 |
| DSC1 | 288.1289526347438 | 92.06135923879457 | 140.51412901886778 | 144.08175778905877 | 222.8438445788035 |
| DSC2 | 2657.2993850916273 | 1665.07467289421 | 1756.1126277536423 | 1848.69563934109 | 2561.3753525459197 |
| DSG1 | 6871.190167794233 | 4437.803780012287 | 4755.710281849305 | 5005.4889966765995 | 6176.354662583705 |
| DSG2 | 147.28602048351567 | 173.52855254347176 | 180.2087802916509 | 160.66441176008928 | 183.89366363295932 |
| DSG3 | 5986.31695634467 | 5448.663210589651 | 5318.04974322959 | 5465.660041414087 | 5810.849966259267 |
| DSP | 8834.99135015292 | 7078.10022032359 | 7136.174901894371 | 7303.5603783592605 | 8589.771424457758 |
| EVPL | 819.4022483706027 | 762.1460275321444 | 802.6305160728914 | 852.941309909598 | 875.5700886709951 |
| F11R | 1955.4156626335882 | 2048.3730619600333 | 2035.1643441046654 | 2019.4155359309434 | 1891.7834474923523 |
| GJA1 | 3857.607249698686 | 2870.9070155003997 | 3075.3756678431846 | 2907.734550364008 | 3677.723821610396 |
| GJA5 | 91.98913679545933 | 119.19198004068643 | 109.16746587987136 | 106.2911964737456 | 96.2820181976886 |
| GJB2 | 4741.748686211122 | 6032.840898266163 | 5570.40360026725 | 5802.9033615294775 | 5039.247117336659 |
| GJB3 | 318.96225692354045 | 304.37913718329537 | 296.11209452171187 | 297.8891306398394 | 310.1898441969258 |
| GJB4 | 34.0587731588413 | 45.92105257165448 | 40.39096632737442 | 33.664035887622845 | 36.16282054944699 |
| GJB5 | 508.1388432815031 | 395.7330603408546 | 373.8509777801464 | 391.5137665801592 | 417.85105726389247 |
| GJC2 | 75.53731962107663 | 75.61775544008117 | 81.33265507151768 | 79.08754395312043 | 79.54698284336553 |
| JAM2 | 476.98989543154363 | 668.2939263682296 | 647.0697411726859 | 613.729791611601 | 503.1808113403616 |
| JAM3 | 130.59400214212383 | 173.9740373357102 | 161.98957576563419 | 150.71566056340043 | 135.85311600360114 |
| JUP | 4139.132379312523 | 3347.0036637123158 | 3312.1801400038285 | 3478.5678979721542 | 3852.149376378451 |
| MAGI1 | 208.53405134004123 | 163.4791839859881 | 166.9588762012498 | 174.1756608846439 | 207.75621514784518 |
| MAGI2 | 56.23898368604844 | 58.93361420967936 | 50.096080876414106 | 54.88865747614459 | 59.40766966843407 |
| PKP1 | 3790.508730024345 | 3009.5915873960057 | 3010.27267352741 | 3154.6098855954133 | 3601.5716732314445 |
| PKP2 | 120.20034631112958 | 155.7681800560243 | 137.04780231469957 | 146.46541454188593 | 124.91183338275239 |
| PKP3 | 614.1717237899487 | 467.7978093829567 | 498.67442954676216 | 517.5421849068927 | 569.3759825826035 |
| PKP4 | 610.0547867891094 | 596.7695877685162 | 574.5621934256806 | 603.1561055326134 | 626.812854543567 |
| PLEC1 | 415.60322333951024 | 392.7329840741727 | 416.75033492593144 | 399.6614741607693 | 438.75972822396557 |
| PPL | 1538.8562094416131 | 1610.2388252837611 | 1760.414641387809 | 1856.2365193392025 | 1748.2255403394277 |
| STEAP1 | 115.4566998828415 | 208.42697061957404 | 157.47031273129335 | 142.1226379221817 | 100.90467517935971 |
| TJAP1 | 428.511052350497 | 471.60295048541593 | 474.84678762188446 | 450.9971962899732 | 427.07089146711024 |
| TJP1 | 832.5596136691651 | 881.5591513044668 | 874.1819353193179 | 887.9147111273537 | 893.9731260229614 |
| TJP2 | 484.2234270365373 | 584.9778931844287 | 553.9227387032663 | 546.4262736291494 | 469.73620594403445 |
### Chart: Y
| Category | BL | 2 | 1 | 3 | 5 |
|---|---|---|---|---|---|
| CLDN10 | 45.1085743413163 | 75.38707261112091 | 69.74577221890073 | 62.72067352505842 | 44.946591656742925 |
| CLDN8 | 29.119654251039517 | 30.435583050450727 | 35.3416173928654 | 35.19530967525546 | 48.93733606119823 |
| DSC1 | 325.719203570384 | 111.01996304692388 | 117.53112599229163 | 134.55379540968465 | 119.77659569909636 |
| DSC2 | 2718.95002923261 | 2029.40040504877 | 1759.2436314640906 | 1906.7017117229775 | 2031.3377828727632 |
| DSG1 | 7089.114927500589 | 5297.55936023098 | 5078.009403232858 | 4812.137359273852 | 4901.898183615237 |
| DSG2 | 173.1040722584083 | 139.797855106244 | 144.08285237633987 | 137.1665294797315 | 147.21571364633107 |
| DSG3 | 6037.743784314017 | 5640.01372768795 | 5790.084296567423 | 5323.504086065026 | 5690.747351510315 |
| DSP | 8790.997412659142 | 7223.101448353717 | 7136.799439503491 | 7000.418773072151 | 7311.813627282592 |
| EVPL | 742.1457785571082 | 692.3538565468583 | 780.0224768007436 | 721.0985629703559 | 828.1030844364642 |
| F11R | 1902.5307305487222 | 1906.9767718033052 | 1904.0170557036 | 1831.4845317049683 | 1840.442381749227 |
| GJA1 | 3861.742886366329 | 2816.3109104516475 | 2818.627505502599 | 2971.6471429776 | 3093.336960416839 |
| GJA5 | 98.55843319604597 | 117.8557810447339 | 128.21050633586472 | 118.27510576861397 | 124.68935931604811 |
| GJB2 | 4511.394265686813 | 5918.00903000277 | 5948.386329367309 | 5517.900584339628 | 4962.963649806602 |
| GJB3 | 297.6065235620547 | 298.9383034887352 | 318.7084210582359 | 287.7639629568691 | 280.8640462711406 |
| GJB4 | 37.73987025165555 | 41.61049033167444 | 44.1017573021907 | 35.04716515039582 | 35.88078015706098 |
| GJB5 | 485.31170055996466 | 386.664061061343 | 427.13398789590235 | 382.6329696048621 | 447.41211833552757 |
| GJC2 | 84.04376002370252 | 94.45208721371505 | 89.49030915386486 | 85.8407933046732 | 97.13958846169778 |
| JAM2 | 533.7109311260489 | 757.8986499099052 | 686.0244067483404 | 696.6815422451235 | 536.7089073932838 |
| JAM3 | 120.67956272882793 | 134.9540940478413 | 128.28145115066874 | 129.29727709927843 | 109.34419571802748 |
| JUP | 4751.8293478619635 | 4076.0483650341434 | 4041.0173381607756 | 3790.7119536362643 | 4164.099744782884 |
| MAGI1 | 256.0705008969992 | 194.06639284711542 | 190.62205672835967 | 189.12410374457946 | 208.5547812093932 |
| MAGI2 | 49.69314368442204 | 54.28411370421026 | 55.43398209056457 | 56.60118386552443 | 54.4689174434066 |
| PKP1 | 4085.3331934442613 | 3302.583210709328 | 3289.9985493094127 | 3202.1355188400976 | 3451.566291834995 |
| PKP2 | 127.68021527813337 | 141.4383050870798 | 157.98358190569053 | 153.33785786101234 | 134.79642327780988 |
| PKP3 | 574.6263077238309 | 427.53608523920275 | 462.68419218780787 | 413.69397359313257 | 442.10880086634916 |
| PKP4 | 608.0991081055529 | 565.4417679707147 | 541.3910399059354 | 570.2092375921967 | 541.7396126476456 |
| PLEC1 | 381.1581111200813 | 314.0303646292175 | 345.7061304482461 | 340.9218219502521 | 342.9421364823363 |
| PPL | 1612.5317622368657 | 1684.8871183299946 | 1873.8199133310113 | 1671.0143694262724 | 1871.7218572688278 |
| STEAP1 | 107.97439126736394 | 219.70355720059868 | 189.7847849945476 | 166.73214851121713 | 107.09248161913818 |
| TJAP1 | 431.0559048535182 | 506.4002925653845 | 461.0997317679829 | 469.13841655287354 | 472.78724779384254 |
| TJP1 | 972.9412065995267 | 1046.813152438147 | 1087.7534214644909 | 961.3253804292876 | 1015.1811175206213 |
| TJP2 | 548.0885094739763 | 571.5191692379168 | 579.5486858725629 | 501.9476667304585 | 514.2503558512807 |Normalized Expression
### Chart: ADU
| Category | BL | 2 | 1 | 3 | 5 |
|---|---|---|---|---|---|
| CLDN10 | 46.46904202297539 | 122.11964680864892 | 89.71312853041508 | 79.45994639250725 | 49.9837342190355 |
| CLDN8 | 33.33472342505557 | 43.15247544860142 | 47.368070921278566 | 34.52539149122669 | 40.073006363043724 |
| DSC1 | 262.4387767511945 | 87.4985367239514 | 50.958145100573674 | 87.18985379258567 | 50.27059784094959 |
| DSC2 | 1547.9383712294632 | 1150.2268964572168 | 1087.1447959015961 | 1154.1744133648626 | 1254.3270998281105 |
| DSG1 | 5078.3092979265275 | 3561.2440704622345 | 2428.6295949933237 | 3567.2591661904275 | 2387.193708306907 |
| DSG2 | 120.53641573665313 | 114.49387881237845 | 106.82315469033387 | 113.50441846521939 | 121.89553653891517 |
| DSG3 | 4861.554937717579 | 4520.156402082812 | 4151.826549086209 | 5006.61615744471 | 4492.026178845726 |
| DSP | 6866.032740564006 | 5090.635847833585 | 4922.578164227143 | 5737.949478243911 | 6100.947876950183 |
| EVPL | 739.179977592831 | 574.9509159273088 | 660.3026113017397 | 715.3189390810053 | 866.8432562284647 |
| F11R | 1680.073677697938 | 1671.2684473503862 | 1663.6150834459675 | 1695.5873626404973 | 1714.7321445031764 |
| GJA1 | 3283.718627882781 | 2413.1482099335176 | 2342.449429448067 | 2443.708151506478 | 2496.094809869566 |
| GJA5 | 90.89548639832232 | 124.43542565398515 | 115.00416645204284 | 115.57291574182032 | 97.7338115869581 |
| GJB2 | 5497.0519930713435 | 5900.1750933127505 | 6034.653464375346 | 5988.143019943179 | 6644.609161703544 |
| GJB3 | 315.73738434008163 | 263.0247556283959 | 273.4223886324072 | 251.41465099055753 | 275.0504437155953 |
| GJB4 | 32.352078636904366 | 41.89204247965271 | 40.609840494566086 | 37.595851560480064 | 34.48768191858328 |
| GJB5 | 326.6985658392668 | 247.33763007729925 | 233.27501716260693 | 230.02148352253803 | 258.363737127612 |
| GJC2 | 77.95160041923353 | 80.647093006636 | 76.17462088408504 | 72.68725686485817 | 82.71872117877218 |
| JAM2 | 441.344162267786 | 640.6983835018553 | 621.0353520646443 | 608.2980306288578 | 371.5920204820069 |
| JAM3 | 124.94722477766497 | 176.34421367785774 | 158.22582890070584 | 130.04045859238633 | 102.23065659177868 |
| JUP | 3354.637429501466 | 2271.5804425263063 | 2329.028808063509 | 2665.494612697001 | 3078.892020832664 |
| MAGI1 | 188.41083055316136 | 140.89462606894867 | 129.65576749861577 | 150.0723741781622 | 156.560408509309 |
| MAGI2 | 54.904295729242314 | 46.168015839141006 | 48.718529820402956 | 49.1099202461562 | 47.30530866009561 |
| PKP1 | 2754.3521016358677 | 2127.9992769753007 | 1834.7768226037454 | 2499.1741871603745 | 2110.1572521471876 |
| PKP2 | 115.44038570205628 | 133.7685005595595 | 146.9802092597647 | 131.5406354403945 | 148.20561636620556 |
| PKP3 | 529.0913620072149 | 326.03090872579173 | 324.2275603547586 | 395.14380896220297 | 426.38632377985203 |
| PKP4 | 466.01331645143625 | 443.7156637765463 | 426.74891536806257 | 459.1464388524862 | 428.87280233609323 |
| PLEC1 | 362.0800875123241 | 327.5384192092472 | 332.446049983675 | 322.06037917488624 | 344.1662506493716 |
| PPL | 1602.1371278746183 | 1321.7341380042517 | 1445.539841191906 | 1708.194821784108 | 1972.325743611375 |
| STEAP1 | 102.47353813058152 | 181.58703498072873 | 153.06387393710364 | 132.2496590221029 | 105.6289623112988 |
| TJAP1 | 360.2139829125074 | 354.3945499406815 | 372.2333938318094 | 361.23457184250975 | 322.4262923479452 |
| TJP1 | 959.815432277691 | 857.4688871221813 | 915.375136863432 | 988.6245482078092 | 1046.6809632558163 |
| TJP2 | 624.8333205641391 | 695.3362398958064 | 658.211405453601 | 652.8487573883516 | 657.6413082470582 |
### Chart: AG
| Category | BL | 2 | 1 | 3 | 5 |
|---|---|---|---|---|---|
| CLDN10 | 44.562033212256786 | 91.94391622375649 | 80.60594052438606 | 66.87096928500647 | 49.350677961504665 |
| CLDN8 | 31.188072371254616 | 31.606058119633822 | 34.2864068720718 | 37.35239952738327 | 39.162202507441826 |
| DSC1 | 106.80819882781819 | 61.55359749701039 | 175.8644567609529 | 61.78242093772896 | 59.268447985418156 |
| DSC2 | 1604.3756848552543 | 1078.4332856745038 | 1079.3068957631197 | 1137.8892977037133 | 1397.348714542378 |
| DSG1 | 3876.6748556779753 | 2744.089338589395 | 4855.99046477566 | 2867.2819181419004 | 2545.0023061331935 |
| DSG2 | 96.30249342462658 | 121.78147350855822 | 110.25906853800726 | 116.25200125710174 | 110.30635885110625 |
| DSG3 | 4662.190711225163 | 4048.5243967527545 | 5083.391352763956 | 4603.330926192068 | 4238.998380727022 |
| DSP | 7028.351812856217 | 5354.651126680048 | 4957.250576198513 | 5427.540117757886 | 6235.100156507228 |
| EVPL | 761.519795883061 | 721.8391214394719 | 684.2583049470604 | 718.2761380124115 | 785.2016723607594 |
| F11R | 1615.3954151781463 | 1598.3847176622485 | 1512.977320440365 | 1645.533427941863 | 1702.305604287588 |
| GJA1 | 2978.6152833221868 | 2356.612720317993 | 2108.2663686694195 | 2387.331970481957 | 2606.53361020882 |
| GJA5 | 103.84919748588129 | 140.37219328725521 | 128.63096497712448 | 122.01571622445346 | 111.5450918025901 |
| GJB2 | 6242.05111652109 | 6502.391780869915 | 6216.422998265651 | 6683.314249948613 | 6376.977931991722 |
| GJB3 | 276.4923561917687 | 315.5922961043076 | 327.2295311613417 | 305.1100946752099 | 281.9484374198412 |
| GJB4 | 32.712155735196944 | 45.152049896452496 | 41.13961481896262 | 37.516847743976655 | 34.60776319345713 |
| GJB5 | 295.70966622759767 | 259.9587010520993 | 253.51009091530892 | 233.44217287764909 | 257.00998319159913 |
| GJC2 | 77.1020783588213 | 79.053273229087 | 80.05338033309928 | 71.55936868341203 | 80.29258812724987 |
| JAM2 | 468.1044040627139 | 674.4051485687097 | 553.5289355541846 | 608.2263568580681 | 534.756399864165 |
| JAM3 | 103.76396977608374 | 145.02422818325806 | 110.91559736117912 | 120.5438700693908 | 119.28679378946198 |
| JUP | 3420.4964704050412 | 2527.0395279743657 | 2612.980884608677 | 2608.142483204344 | 3086.2282581795575 |
| MAGI1 | 191.20958609778097 | 151.6602523595757 | 140.4348973872044 | 143.52368715679086 | 174.8612180469882 |
| MAGI2 | 50.4100024954514 | 53.70588793691898 | 48.42344854585773 | 45.406193686634 | 50.50948633552262 |
| PKP1 | 2380.3739960221446 | 1839.5287764142622 | 2784.2301599779817 | 2236.4867993596954 | 2098.3610256417155 |
| PKP2 | 134.03785971168006 | 133.43134475093373 | 117.57397963108114 | 143.3735297093939 | 142.20485587499564 |
| PKP3 | 527.9664181036719 | 362.27546850625953 | 367.00313937962227 | 350.5806564663266 | 412.35836711003117 |
| PKP4 | 419.7666017251908 | 412.4051041013107 | 480.0335836648673 | 444.7402098722898 | 403.4375771407706 |
| PLEC1 | 326.5044579321441 | 341.5533948299896 | 331.8512714942048 | 345.04742968205323 | 358.2950934055209 |
| PPL | 1639.5441952650172 | 1520.6249753836757 | 1465.3912853715528 | 1495.9498733909345 | 1627.102490148134 |
| STEAP1 | 108.18156608672179 | 182.7064572620309 | 138.65661969967394 | 139.5774708564671 | 116.31369079343368 |
| TJAP1 | 361.77721857596157 | 395.9079637711934 | 332.671592287811 | 373.44671273451087 | 352.5351478248035 |
| TJP1 | 1007.6792533908888 | 917.6808522001379 | 830.5710391666589 | 940.8632721991152 | 958.048165952568 |
| TJP2 | 605.2276643914694 | 603.1906452185021 | 573.1237563363634 | 623.7303814119103 | 542.4516921859008 |Normalized Expression

## Slide 13
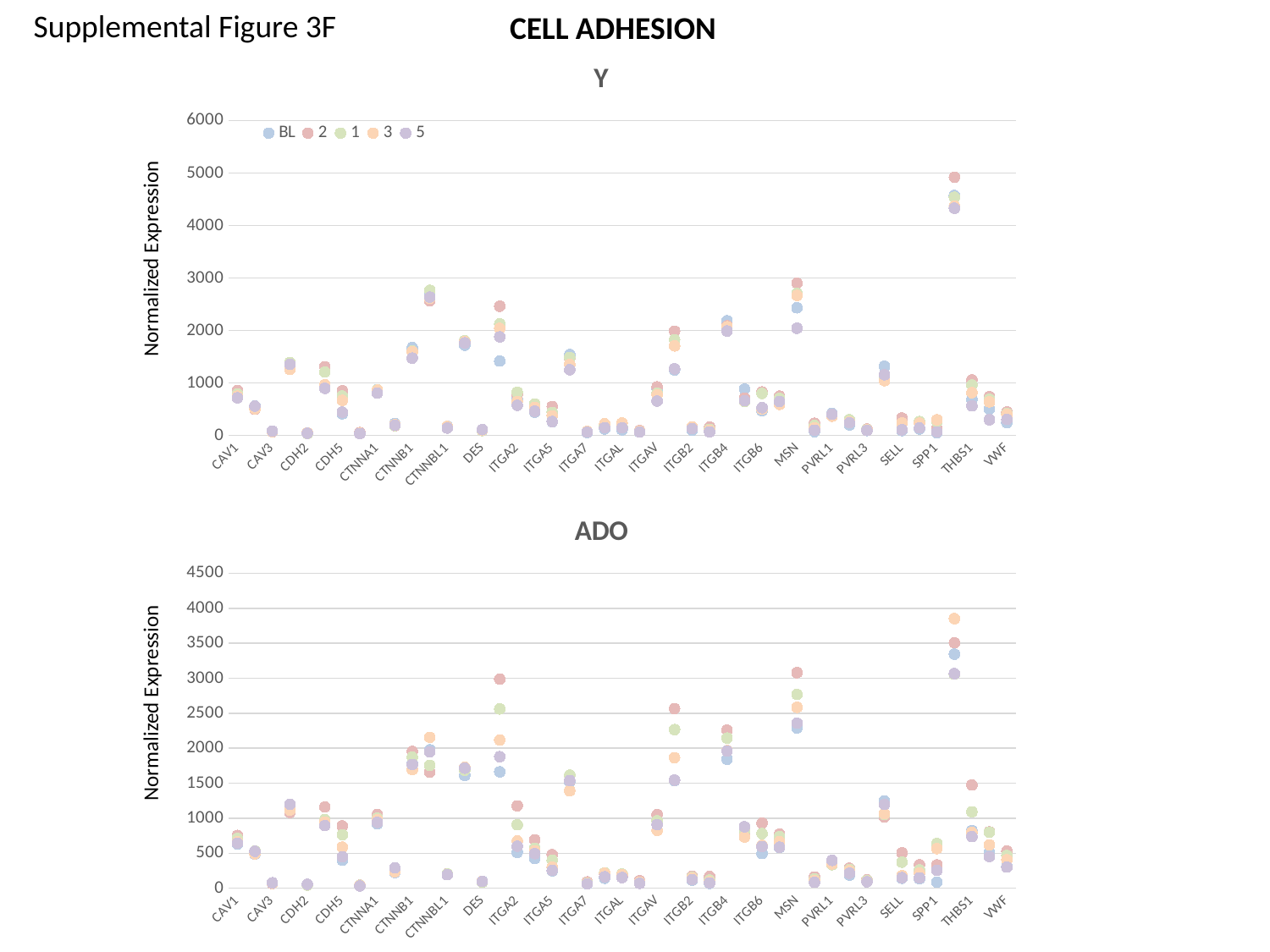

Supplemental Figure 3F
CELL ADHESION
### Chart: Y
| Category | BL | 2 | 1 | 3 | 5 |
|---|---|---|---|---|---|
| CAV1 | 779.0053825127395 | 858.5423360564904 | 790.2660967558061 | 751.4116188637537 | 718.9964208617885 |
| CAV2 | 561.6697750563628 | 501.9101368261429 | 518.7339511508936 | 510.8138049844839 | 560.0435808481235 |
| CAV3 | 82.45506859482502 | 72.38451013311854 | 76.2582331149257 | 72.98892628762577 | 85.14152462996013 |
| CDH1 | 1361.706842480478 | 1303.6841724864564 | 1391.6582822708162 | 1258.2393912526836 | 1356.1064502185777 |
| CDH2 | 51.90693530747786 | 45.32254960603599 | 39.68522448011058 | 50.80566371929057 | 44.61728329240509 |
| CDH3 | 936.7575946342129 | 1311.0430457916555 | 1210.4986363277758 | 967.8083379201578 | 899.5157781939172 |
| CDH5 | 414.5330837961312 | 852.8137310432436 | 750.6531095934535 | 669.9060521420477 | 446.03785319237267 |
| CEACAM8 | 35.36621701238474 | 56.3366795448126 | 41.361722906690765 | 42.82591245036722 | 37.151514377960645 |
| CTNNA1 | 873.0496797041027 | 852.3423516453502 | 871.7579253523548 | 864.4839824761752 | 808.5729969467393 |
| CTNNAL1 | 230.7384802731853 | 186.405001588799 | 191.0209155445123 | 206.6572166589831 | 197.4871094824349 |
| CTNNB1 | 1675.8307552925253 | 1600.6433597891046 | 1609.0466993346138 | 1598.8606955427877 | 1474.5617823431287 |
| CTNNBIP1 | 2702.1026240232713 | 2566.138534022945 | 2765.4570524858223 | 2615.870404837888 | 2635.5983019734394 |
| CTNNBL1 | 175.89095305999123 | 166.17150578123605 | 170.9157469537531 | 171.5662796921907 | 143.5788269544938 |
| CTNND1 | 1719.1400290787049 | 1768.3532787138602 | 1804.5140990770624 | 1786.5945390356278 | 1764.5480500053632 |
| DES | 96.7775351391349 | 105.19766583395419 | 95.69446331159997 | 106.87334160297229 | 112.04969165455715 |
| FN1 | 1417.457290754557 | 2462.4233908147294 | 2128.8376396808035 | 2040.178010611073 | 1879.034200844418 |
| ITGA2 | 693.4292253833322 | 780.3946740107363 | 825.1697633233198 | 646.6963614308793 | 576.4282011176064 |
| ITGA3 | 445.5084757226506 | 555.643837787668 | 600.7179910660054 | 535.9482850431182 | 462.5781186670631 |
| ITGA5 | 261.05486165475287 | 553.1390353966342 | 442.04171912392417 | 377.29699118648284 | 266.0365140585908 |
| ITGA6 | 1540.933176959835 | 1474.490070552843 | 1486.0980491335313 | 1352.557675653006 | 1252.667402615256 |
| ITGA7 | 57.00465297657195 | 72.09613157123032 | 70.17974446350314 | 76.77091131087874 | 64.12205231350839 |
| ITGA8 | 125.40827809498835 | 152.46671168236475 | 189.08440768455375 | 223.91961973315858 | 151.04250161938117 |
| ITGAL | 108.53255952946441 | 157.8032147280233 | 167.32395000848894 | 237.60106879778388 | 147.35321049075162 |
| ITGAM | 64.98713721798423 | 92.75343941116074 | 68.69000256736993 | 85.8554866522582 | 68.35051693398071 |
| ITGAV | 898.8783488029103 | 927.5754943994939 | 806.4894114840549 | 773.7832432558791 | 656.6975507342863 |
| ITGB1 | 1249.4550882389383 | 1988.5792880613355 | 1825.6158796397815 | 1706.8190421149484 | 1269.7531550387646 |
| ITGB2 | 99.56785736701438 | 156.3491140373713 | 145.80263323556784 | 166.31455171913538 | 133.04182456216571 |
| ITGB3 | 68.2608852394088 | 159.82942952176313 | 119.1968845264149 | 100.5624695817744 | 69.53619224362426 |
| ITGB4 | 2184.1048550907167 | 2088.939364095196 | 2028.3331923610292 | 2070.7029493061727 | 1990.1393102967875 |
| ITGB5 | 884.8107712070129 | 714.9451188895073 | 649.6916673771302 | 662.7550139668768 | 663.9378001666119 |
| ITGB6 | 471.57622813739425 | 829.7231353482053 | 801.8503758098359 | 505.17014023603235 | 529.660817933255 |
| LGALS3 | 678.2068733273795 | 749.6296014316041 | 705.3633487339171 | 594.777962628626 | 649.3107178164563 |
| MSN | 2432.679227249848 | 2900.9927977294533 | 2707.2617022628833 | 2667.9789036282427 | 2043.5438145431815 |
| PLIN2 | 72.78326845957241 | 232.98182468358377 | 191.38124629802462 | 147.70365310672491 | 103.04767624715964 |
| PVRL1 | 422.6837661543075 | 370.0709166821224 | 377.0540599526886 | 368.2414921183802 | 403.16607213841087 |
| PVRL2 | 203.99290119500137 | 296.0610136857125 | 300.0147171559966 | 256.17612177283223 | 241.9825015343182 |
| PVRL3 | 94.91170867541895 | 122.53340404357444 | 111.44251924984997 | 105.35671153243793 | 96.39070318227112 |
| PVRL4 | 1319.3061426552958 | 1049.741478094241 | 1143.6503703396684 | 1044.950267927304 | 1157.433456612436 |
| SELL | 95.2423357858482 | 333.722125312503 | 236.0159709857264 | 239.8249808085294 | 109.6316121798866 |
| SELP | 124.52359458159903 | 265.90731624973824 | 264.8328660223862 | 236.05081705124636 | 144.73373653778916 |
| SPP1 | 51.3917651421482 | 135.15717484295408 | 266.8069934396766 | 299.83394265468013 | 70.99947871636485 |
| SPRR2D | 4571.317612775484 | 4921.188280410429 | 4538.296368657172 | 4371.6332695207575 | 4330.056455275545 |
| THBS1 | 682.8105115924828 | 1057.553810667154 | 962.2539786524051 | 816.1543297824907 | 565.4748486066193 |
| THBS2 | 505.98509668996616 | 740.1490746463037 | 689.0960326116541 | 641.2969474431507 | 300.9768025575961 |
| VWF | 246.13769604983008 | 449.53401394639724 | 419.27587161868905 | 402.4000993887222 | 307.910479758399 |Normalized Expression
### Chart: ADO
| Category | BL | 2 | 1 | 3 | 5 |
|---|---|---|---|---|---|
| CAV1 | 632.1187847933924 | 754.6334668905583 | 712.3966865528182 | 660.0923619616423 | 645.4903158943293 |
| CAV2 | 486.3763801965992 | 506.240326994541 | 532.1005607111479 | 492.84704530019854 | 527.414338459515 |
| CAV3 | 70.23586043391232 | 66.82795459889218 | 70.86148037486133 | 71.05896620312666 | 78.21313105260907 |
| CDH1 | 1128.2458721905557 | 1082.6773663676877 | 1118.2975255469505 | 1117.5090184523056 | 1198.9547307122314 |
| CDH2 | 56.70644914654752 | 54.90582391813409 | 45.18869455105238 | 57.116577433493426 | 57.406824168875374 |
| CDH3 | 948.6789803760873 | 1160.7722174124747 | 981.0678956152702 | 947.6589870642212 | 895.2488279294391 |
| CDH5 | 399.78732660114594 | 889.6825144638079 | 764.8105683469092 | 587.0560020319286 | 448.955333618334 |
| CEACAM8 | 33.82332353270321 | 45.13337621381782 | 42.526726875254624 | 37.727529467398966 | 32.299903967687825 |
| CTNNA1 | 919.7187365805471 | 1053.254788357206 | 1000.2888554880052 | 979.9345189581117 | 940.8374787928229 |
| CTNNAL1 | 224.97830136634707 | 239.16508465528972 | 251.76063917385466 | 234.54560353767627 | 292.0599547115403 |
| CTNNB1 | 1779.425628158937 | 1955.180186256099 | 1873.1997427677152 | 1695.4527443713846 | 1769.2541196138916 |
| CTNNBIP1 | 1977.37753069103 | 1658.6012176756904 | 1756.4860570993003 | 2156.0788454910416 | 1949.1725428731556 |
| CTNNBL1 | 199.72385221338462 | 204.64941084900317 | 202.74333028670435 | 193.9684362371919 | 193.01010233857343 |
| CTNND1 | 1611.92024139344 | 1725.5871543979736 | 1686.5050135919253 | 1726.3504936560896 | 1715.2667147271143 |
| DES | 100.39916208646748 | 85.357579411621 | 85.49683430666165 | 98.57860307373662 | 95.99914992740109 |
| FN1 | 1662.8419693333387 | 2988.362420750776 | 2562.0295694572496 | 2117.4283678254747 | 1879.1469329824458 |
| ITGA2 | 512.4493212330116 | 1177.551873841332 | 906.3924528012423 | 674.5432587971909 | 599.9642497395363 |
| ITGA3 | 423.16124552309407 | 691.741314366671 | 572.3289200216959 | 535.2330881093576 | 494.4661337429556 |
| ITGA5 | 248.58159455050267 | 478.2614821771015 | 401.18781506649543 | 302.9722585059519 | 255.54014835408123 |
| ITGA6 | 1518.5902661332207 | 1537.8601123776941 | 1615.5760610731852 | 1391.9798178156384 | 1538.9285981320002 |
| ITGA7 | 60.412565429012666 | 90.43480088615607 | 75.98206533634547 | 68.06033749822426 | 65.39218110006568 |
| ITGA8 | 143.0907812781121 | 202.16354518387791 | 221.3211922958468 | 212.4585160824728 | 163.55929983296988 |
| ITGAL | 153.90333688577826 | 180.38095272483164 | 204.13540227774524 | 193.80717694533848 | 152.89883150754238 |
| ITGAM | 72.64970139978487 | 106.65900962456573 | 89.35857267895985 | 88.18895490021826 | 70.41788368302099 |
| ITGAV | 970.0903559263102 | 1050.605971681781 | 962.5307056334482 | 826.850176469395 | 908.8874701796816 |
| ITGB1 | 1538.052730985453 | 2566.4084742634036 | 2265.943490863737 | 1865.0311924373661 | 1545.0583071162437 |
| ITGB2 | 115.12521123498485 | 169.55308811002385 | 154.42689553641367 | 144.99217666135195 | 122.35354810216904 |
| ITGB3 | 70.53558059997387 | 167.308043978955 | 114.73893875538505 | 93.39896625387561 | 77.31596158890417 |
| ITGB4 | 1842.1395512784375 | 2259.241217420211 | 2144.88061898128 | 1965.4354346733753 | 1961.0920877138867 |
| ITGB5 | 839.8985002311705 | 787.6099148375292 | 824.4236736056009 | 732.9365205364733 | 878.2226542607726 |
| ITGB6 | 496.46444514559346 | 931.1296791038177 | 780.3250715668516 | 613.8546359233196 | 595.6699871871743 |
| LGALS3 | 581.6253486771038 | 772.4363784566008 | 737.4914584085 | 666.1614522243095 | 586.1319944911205 |
| MSN | 2289.0757229991336 | 3080.294016957483 | 2768.6154537958428 | 2585.587332826726 | 2358.78202031698 |
| PLIN2 | 84.49994917091641 | 160.64647734465558 | 132.46860145326872 | 111.18009349635716 | 84.85705334533424 |
| PVRL1 | 374.4265276276817 | 357.2461018157603 | 336.664128746687 | 348.9182583459012 | 397.7779300993982 |
| PVRL2 | 187.3658538013782 | 285.2070213668922 | 268.72104551135106 | 250.38897318919533 | 214.95911993051766 |
| PVRL3 | 92.10494703710566 | 119.23793171600225 | 108.37884258439843 | 95.35326213663392 | 91.81061390682345 |
| PVRL4 | 1245.8874069736955 | 1018.9654303717439 | 1053.8655637375045 | 1065.391104039565 | 1196.2562759714378 |
| SELL | 143.40120697088835 | 504.7715313467445 | 373.15871427982233 | 179.42499125880875 | 150.71132330694542 |
| SELP | 135.62925321645207 | 334.53053559512944 | 263.33423988158125 | 216.80200517730873 | 149.67657044712223 |
| SPP1 | 86.94449922962708 | 333.44334318206774 | 639.5993667087895 | 567.2008466072984 | 254.30553892506558 |
| SPRR2D | 3345.1174775573577 | 3507.4472989125165 | 3058.308615253916 | 3852.010648553038 | 3066.147640255537 |
| THBS1 | 823.6679238573314 | 1475.1405184418009 | 1090.9610216706112 | 797.5677431207508 | 737.5622558503087 |
| THBS2 | 509.444290737029 | 804.8470352509802 | 801.997696307441 | 619.6825568593916 | 452.5518663906931 |
| VWF | 306.29144730495284 | 530.4518527348902 | 468.09463160775965 | 406.97187613545896 | 302.2043533731117 |Normalized Expression

## Slide 14
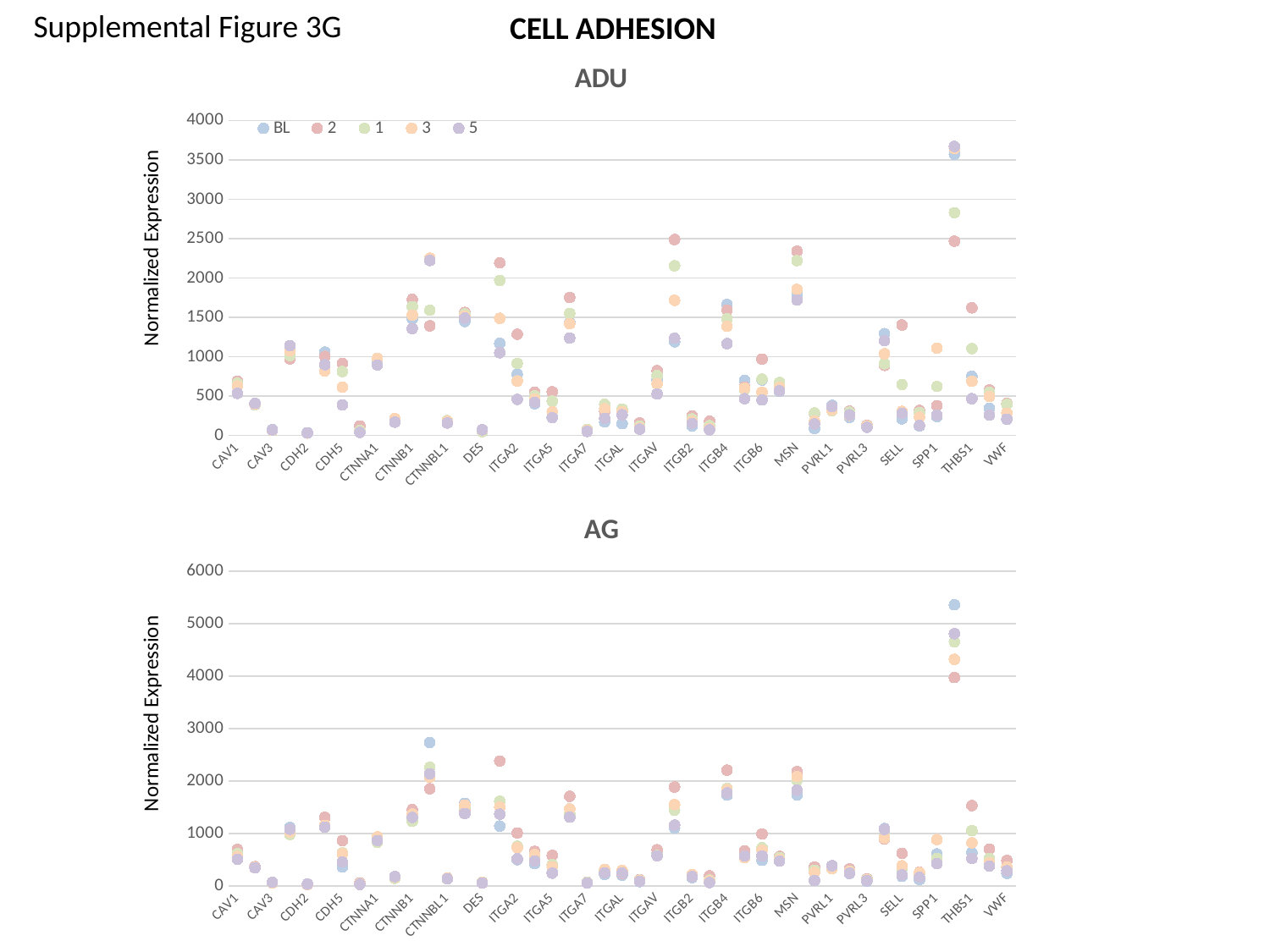

Supplemental Figure 3G
CELL ADHESION
### Chart: ADU
| Category | BL | 2 | 1 | 3 | 5 |
|---|---|---|---|---|---|
| CAV1 | 660.434975822685 | 690.8194661983262 | 662.497277119738 | 629.3353518838215 | 535.5934304249668 |
| CAV2 | 390.8152794815089 | 401.83224152814114 | 393.4866351054186 | 398.75311383788153 | 408.144185797625 |
| CAV3 | 75.3756122292888 | 70.79116008966582 | 64.89672450040382 | 66.18617744896704 | 72.19401366884783 |
| CDH1 | 1081.4021064582653 | 973.4495472702632 | 1015.4716124403452 | 1075.3949355166942 | 1143.0632588439394 |
| CDH2 | 35.715070744144086 | 32.221975857698936 | 31.20411592481592 | 31.105131123447897 | 31.948764834198432 |
| CDH3 | 1059.26678207071 | 1004.3734995679255 | 894.6133254614775 | 819.2948907499768 | 900.0513920724379 |
| CDH5 | 390.99382368407464 | 915.2806255070097 | 812.4175216464624 | 614.4202213736155 | 387.1014683891422 |
| CEACAM8 | 40.615375517501214 | 122.83068794995668 | 64.96402103632056 | 42.72280657269653 | 39.79071963195661 |
| CTNNA1 | 961.3004598543589 | 973.4058812002617 | 976.1081026276692 | 980.904619522223 | 896.0329899544247 |
| CTNNAL1 | 174.19192018922072 | 183.76004952917413 | 201.47221703169552 | 216.16897754324222 | 170.29911168009278 |
| CTNNB1 | 1478.9015145903322 | 1730.3513588998767 | 1636.7222118342165 | 1530.0377024196819 | 1358.6684893409456 |
| CTNNBIP1 | 2223.258287356381 | 1393.3468084896008 | 1590.3863830389482 | 2252.852584918677 | 2223.084916748948 |
| CTNNBL1 | 167.1720254203823 | 182.48421300081628 | 189.43800567208945 | 181.26030327130727 | 159.25103782964288 |
| CTNND1 | 1447.9400488292208 | 1563.2804317310627 | 1545.122995593636 | 1514.637181352788 | 1491.9223818805172 |
| DES | 57.01477233338234 | 55.755779349392014 | 49.91428371479014 | 71.31550186169926 | 74.27807240456843 |
| FN1 | 1171.7784451390958 | 2193.698457067076 | 1969.134190022588 | 1488.179593546487 | 1052.0789376822745 |
| ITGA2 | 776.8859694541911 | 1286.608341958207 | 915.8963421264505 | 692.3461687909252 | 460.18141043678213 |
| ITGA3 | 404.2815712724306 | 549.2865541789171 | 504.1020550587513 | 462.5698342306524 | 419.67588184570616 |
| ITGA5 | 234.56665714778032 | 555.516922528185 | 436.60165423889987 | 303.47234325684957 | 227.08116113882247 |
| ITGA6 | 1437.3396020371385 | 1752.9864548375363 | 1549.8946342033137 | 1420.9162782772944 | 1237.803972111845 |
| ITGA7 | 56.70163266007879 | 76.49507175043357 | 79.63361364250777 | 64.95561514628668 | 51.53937294286766 |
| ITGA8 | 173.2663426774096 | 306.9797938385951 | 395.9740445047629 | 339.6887629696994 | 219.26481367961563 |
| ITGAL | 150.29581938646237 | 301.20469278367455 | 337.3049027590427 | 303.8090648256289 | 263.45343836745724 |
| ITGAM | 80.56916203065938 | 160.26936817543455 | 121.33858706314749 | 95.09915137951779 | 80.62214942625035 |
| ITGAV | 708.1785733704636 | 823.2178419476776 | 761.1347993370239 | 658.6307969061314 | 528.1845320229401 |
| ITGB1 | 1190.5960201047185 | 2489.4604692907915 | 2155.3115204894666 | 1716.8725085555789 | 1236.411096825032 |
| ITGB2 | 120.50581473029443 | 247.83738875252084 | 210.6529143895325 | 179.29622582432498 | 154.37162763226843 |
| ITGB3 | 73.65523957651696 | 181.28542630479086 | 125.69259901798263 | 90.01489089167848 | 71.87726891593269 |
| ITGB4 | 1663.02813105487 | 1593.3277522911417 | 1484.003463671453 | 1387.7206990536342 | 1166.990893352576 |
| ITGB5 | 700.6346656727474 | 616.427289611091 | 586.0392405569156 | 600.7716073494789 | 468.08917203951455 |
| ITGB6 | 705.266461910257 | 970.1829608871054 | 716.3499611144069 | 550.5528778495009 | 451.4663077382313 |
| LGALS3 | 559.3676147290698 | 640.5042705620306 | 673.3431710441697 | 612.861288370566 | 567.8758167238527 |
| MSN | 1792.73423219064 | 2341.146947111662 | 2221.7652768222356 | 1855.7854893944193 | 1721.3704538359166 |
| PLIN2 | 88.64183006128688 | 273.88500233003475 | 288.24453319126076 | 182.49529093410086 | 147.43641695142458 |
| PVRL1 | 383.1363095526361 | 315.71132563476635 | 315.3284702149839 | 323.98579336584794 | 366.8181954351988 |
| PVRL2 | 229.70141884345503 | 308.655823010001 | 295.77500982623224 | 257.14565651194164 | 263.1364861697897 |
| PVRL3 | 111.40559372934952 | 130.31340052414728 | 122.48244332028723 | 117.53364653463248 | 105.43159854743598 |
| PVRL4 | 1293.900661054266 | 891.1202621899542 | 911.8542830054367 | 1038.6965797990351 | 1203.5392761258304 |
| SELL | 212.5684572429974 | 1403.33942326355 | 647.768864014209 | 306.4649745096194 | 281.4183907179674 |
| SELP | 121.2915559130871 | 318.84586141624277 | 292.1027495838959 | 232.47867125669765 | 129.00012091962137 |
| SPP1 | 240.94984897456823 | 379.80846998727446 | 623.2510975431126 | 1110.1485679001003 | 259.1238881302383 |
| SPRR2D | 3571.6781685070123 | 2468.679522105832 | 2830.575893917644 | 3644.436282762365 | 3671.3422242076504 |
| THBS1 | 751.568362865864 | 1621.5904881547247 | 1104.86259182479 | 689.2557350522238 | 467.6652451042363 |
| THBS2 | 345.30582046888014 | 578.2806022711629 | 548.3987451948423 | 495.18711306753016 | 258.8506243230719 |
| VWF | 206.86332060141189 | 408.0072331863425 | 395.0995132901618 | 284.1555444682248 | 208.75330410783474 |Normalized Expression
### Chart: AG
| Category | BL | 2 | 1 | 3 | 5 |
|---|---|---|---|---|---|
| CAV1 | 546.0825456957972 | 699.8407034771573 | 620.3893922083085 | 566.2650260053093 | 511.1326861869394 |
| CAV2 | 361.24134252153533 | 370.9775569281812 | 356.8376838894805 | 355.356988535937 | 350.2127309185717 |
| CAV3 | 73.5063207841451 | 68.22517902121753 | 72.3536281918864 | 61.04030776751439 | 74.24926493495484 |
| CDH1 | 1116.1157593504618 | 1078.0856193556303 | 984.8840455203665 | 1033.705215245857 | 1084.6299258235877 |
| CDH2 | 31.25479082773474 | 28.536794950322797 | 31.016504669517637 | 29.78242608479274 | 39.426391231900915 |
| CDH3 | 1135.429557647104 | 1311.8067955711626 | 1123.2026782798819 | 1159.1526535565947 | 1124.7405144981415 |
| CDH5 | 365.05614823335395 | 867.6693842379462 | 632.1054621135005 | 615.8038776952098 | 459.5078058055847 |
| CEACAM8 | 42.61189168550426 | 63.71914412452216 | 45.91224794025704 | 42.76843709103203 | 33.0092193688398 |
| CTNNA1 | 900.6523364814503 | 885.5538176329881 | 834.8250906804866 | 936.916770274149 | 868.5934488193293 |
| CTNNAL1 | 168.8779100676802 | 164.93120553574778 | 149.70619123713928 | 171.29230990921366 | 183.18542024145054 |
| CTNNB1 | 1303.1306308259473 | 1457.1272095076633 | 1236.3543356388602 | 1372.862126405045 | 1309.4399413626688 |
| CTNNBIP1 | 2735.1172445311013 | 1853.4247423275035 | 2263.430841553633 | 2073.4294503547735 | 2140.142254603517 |
| CTNNBL1 | 150.52462195198237 | 155.31836275575415 | 137.88124922297982 | 154.3325194069149 | 141.42025208750292 |
| CTNND1 | 1577.9656892091311 | 1450.2738105126298 | 1498.2969169147566 | 1545.0826869068744 | 1383.221141512177 |
| DES | 64.82857354526993 | 57.66396678334169 | 79.9217401086535 | 68.654081573622 | 63.15751545764488 |
| FN1 | 1143.9416850987093 | 2383.5182327583493 | 1616.1651764226817 | 1509.028260572299 | 1370.9227549865175 |
| ITGA2 | 501.7050448307985 | 1012.6315603171365 | 761.1292354067277 | 731.9376243288085 | 521.0588468668743 |
| ITGA3 | 426.42911280449823 | 662.6046537603439 | 535.6588465503662 | 600.7005422611635 | 480.30218874044175 |
| ITGA5 | 267.18421018899994 | 585.221890567113 | 410.89259219867347 | 369.14924776422583 | 247.88844636142943 |
| ITGA6 | 1318.1663746308711 | 1710.4233069605446 | 1378.6797044957877 | 1471.7612646349523 | 1314.7434214789907 |
| ITGA7 | 58.24556013429539 | 75.81667041730265 | 70.01785304636273 | 63.23163369779268 | 60.20409066388858 |
| ITGA8 | 221.87606013291335 | 280.7577308733255 | 272.3615153254398 | 319.71596628683506 | 252.51745239551337 |
| ITGAL | 209.7185319203502 | 238.06079057146047 | 262.8846806325879 | 298.3800130651191 | 250.32044001877966 |
| ITGAM | 86.88223380282933 | 123.68910738550237 | 112.92747237615616 | 110.59853598388925 | 91.41101611388444 |
| ITGAV | 621.903774466892 | 692.5700763519989 | 583.0310257774483 | 579.2857245127208 | 577.9434243015434 |
| ITGB1 | 1108.3442389667325 | 1888.5155336041628 | 1447.1781317949915 | 1553.9465280727118 | 1162.0178546112775 |
| ITGB2 | 164.78392776989392 | 204.33695810884166 | 183.12407328155604 | 219.07554751284817 | 181.13205462160286 |
| ITGB3 | 68.98612661038841 | 196.57243129883773 | 114.86534161898497 | 100.54241063966866 | 68.77470295288848 |
| ITGB4 | 1733.439428045181 | 2209.5104648821016 | 1862.2448148074413 | 1837.7075116765477 | 1774.888036927349 |
| ITGB5 | 620.0521376043657 | 672.7904645184462 | 549.5277035400146 | 541.096281230391 | 575.011049635504 |
| ITGB6 | 492.0726153454121 | 993.6228490321319 | 730.4788191593807 | 686.0744638263354 | 575.3571657015608 |
| LGALS3 | 490.1425498862067 | 566.5183670034733 | 544.5825223702915 | 508.6792175688493 | 476.0640590365938 |
| MSN | 1736.5378810268219 | 2181.7454236174744 | 2016.64713135899 | 2086.911949454127 | 1827.8979378570814 |
| PLIN2 | 106.95572810964421 | 364.8458966007651 | 303.5348433069968 | 261.08237574923373 | 109.32574229705952 |
| PVRL1 | 368.2677981491808 | 346.4570321076004 | 332.2301785524654 | 336.7674512380832 | 390.0519851391879 |
| PVRL2 | 241.63106109263356 | 328.71123278523334 | 285.0827243642957 | 277.108807709656 | 245.89955439046176 |
| PVRL3 | 95.38017975287005 | 139.6406529852764 | 127.26599159383817 | 119.28969628406941 | 103.61789942257695 |
| PVRL4 | 1096.8461680467294 | 900.287625599896 | 957.0311073182318 | 916.9606441080294 | 1082.675907122594 |
| SELL | 185.91974962411697 | 624.6193137663181 | 323.0438672653273 | 386.8718698590408 | 214.28905830122216 |
| SELP | 125.07433082718222 | 264.5730442474445 | 242.10168935038277 | 257.79837383550534 | 169.12310331084848 |
| SPP1 | 612.9693423778677 | 470.8457357140012 | 529.9644371728936 | 886.0390787376538 | 427.9291672669154 |
| SPRR2D | 5359.757704451624 | 3973.2780003024536 | 4654.6374805994765 | 4320.564755154736 | 4809.211016111245 |
| THBS1 | 636.6325619727625 | 1533.383680245101 | 1057.8106830044437 | 826.2485318609129 | 528.5561968717466 |
| THBS2 | 403.40537847197186 | 706.4277384907576 | 524.9912561645208 | 448.3880480490541 | 381.42962107351116 |
| VWF | 242.38714072541865 | 488.6951044175438 | 366.9875461434828 | 364.1587539464077 | 291.14606593750295 |Normalized Expression

## Slide 15
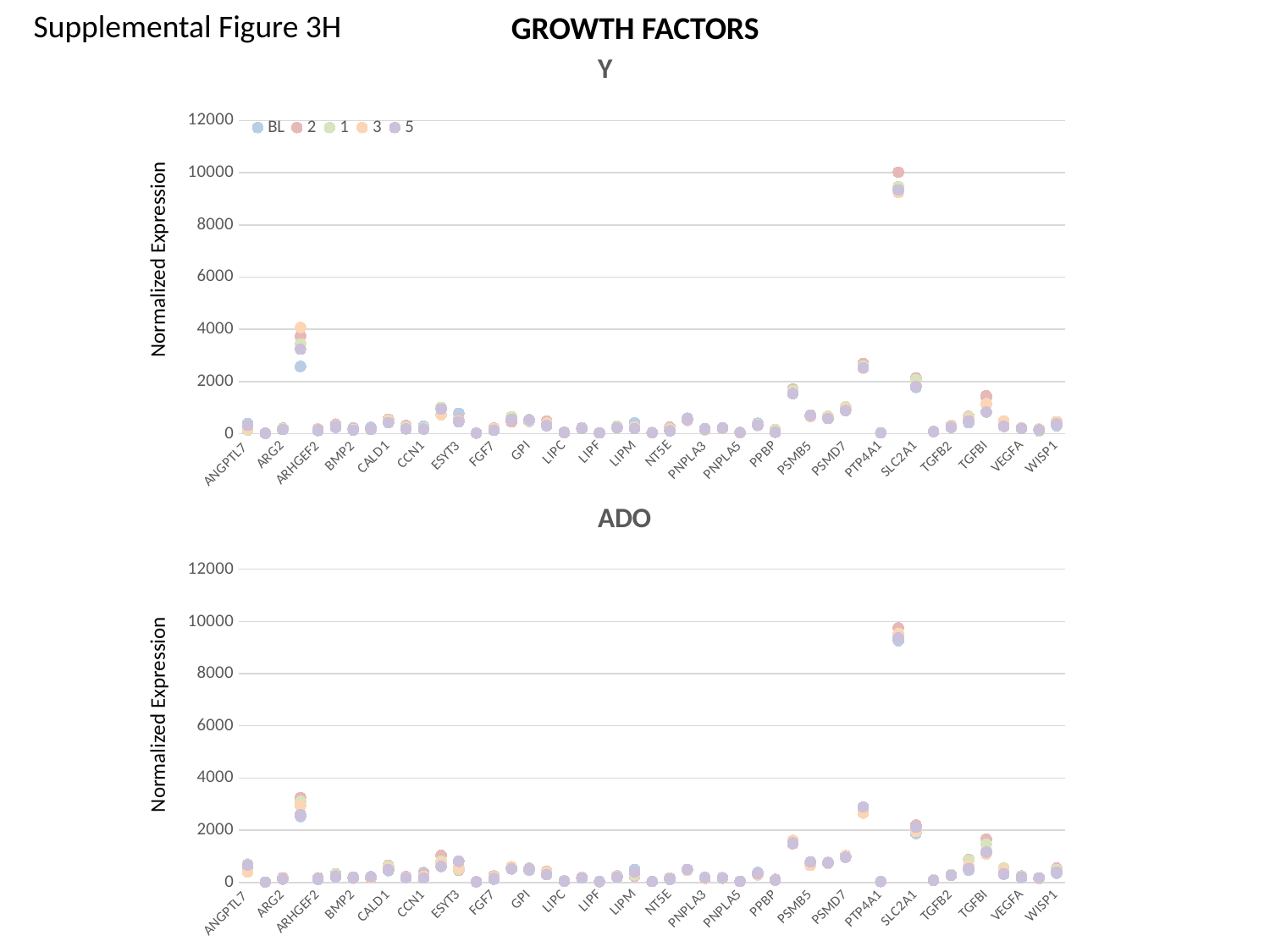

Supplemental Figure 3H
GROWTH FACTORS
### Chart: Y
| Category | BL | 2 | 1 | 3 | 5 |
|---|---|---|---|---|---|
| ANGPTL7 | 388.02491766818616 | 135.50084346694814 | 142.50008407183697 | 187.13278105005662 | 319.09847845585557 |
| APOH | 13.206770170531062 | 14.00221271019117 | 13.409697652046994 | 15.35904630216146 | 15.037299246293887 |
| ARG2 | 165.6151468941625 | 214.72136635216918 | 190.4655892687072 | 156.31366885895125 | 155.72149186889595 |
| ARHGDIB | 2573.5052663556685 | 3740.264539216105 | 3442.2479130509996 | 4069.4446736733466 | 3237.832470116991 |
| ARHGEF2 | 115.97389363510082 | 181.6067186940978 | 162.73747058540664 | 173.5045342444496 | 144.6151258949531 |
| BMP1 | 236.169167404528 | 364.321461994247 | 304.81243554110017 | 283.5298236095074 | 278.18797058773083 |
| BMP2 | 220.4358675517448 | 175.30762104564047 | 160.70075695578947 | 147.72193726429535 | 135.37675614957323 |
| BMP7 | 239.57522562511335 | 157.77154433537856 | 170.56878290883165 | 179.9170966025963 | 180.04350635149396 |
| CALD1 | 420.9528577442644 | 553.4760150681417 | 508.9352155169553 | 476.7058138062169 | 423.8378995054804 |
| CAMK2N1 | 208.35068051870496 | 309.2856442956819 | 256.4505367905397 | 202.94294535494885 | 181.28758543305892 |
| CCN1 | 286.3103188100153 | 220.78097705798754 | 226.46788057708793 | 182.27287235929458 | 175.71843137378477 |
| CTGF | 969.8473618735856 | 993.6344609156217 | 1010.3349887073548 | 724.2141150362411 | 941.3099754517625 |
| ESYT3 | 783.7986014575404 | 534.1380046128124 | 480.1426690363785 | 486.3100620937816 | 453.58259428509416 |
| FGF10 | 22.92947808015721 | 23.68266252488469 | 21.68291490221056 | 22.23155799046755 | 21.858799195574232 |
| FGF7 | 122.89286868801904 | 213.80516426078788 | 176.4106978999507 | 190.1966037941722 | 136.308743174416 |
| FGFBP1 | 527.5574083712044 | 453.4575013031107 | 649.2111020613683 | 577.5608616827551 | 551.89659032552 |
| GPI | 468.96042990526956 | 502.9264336942407 | 504.4876303951557 | 498.07414564224655 | 535.1829674147245 |
| LIPA | 305.23839337336386 | 475.72276321935595 | 390.82275898883177 | 401.61041324984046 | 313.1703110062583 |
| LIPC | 36.10640128695118 | 53.407415712530934 | 48.38586071194485 | 48.48553299182005 | 50.65877980454909 |
| LIPE | 186.8576062626998 | 228.14646830804836 | 217.69838357110376 | 194.47716431388181 | 207.64942821167136 |
| LIPF | 28.778390115171028 | 23.745276169074057 | 26.023510535080803 | 23.824238428542223 | 29.342526034160397 |
| LIPG | 254.81661367814772 | 223.58892361151024 | 290.63179981330063 | 231.38299047332737 | 224.69935922514853 |
| LIPM | 412.8315781106235 | 255.9882855914188 | 257.14293972271815 | 241.6896074515476 | 191.70731047133643 |
| NODAL | 34.1627167998198 | 35.07628400825874 | 37.88742760358589 | 34.50733994106425 | 39.42502064146164 |
| NT5E | 132.3944443334662 | 247.89327680959104 | 183.39502712352044 | 139.80890173168413 | 106.03528744356178 |
| PNPLA2 | 592.9078313438359 | 553.3440893236083 | 521.5316869529607 | 503.2842629910357 | 534.8698210416144 |
| PNPLA3 | 191.83934662576704 | 149.9594488626383 | 144.7991163117253 | 151.19406040316483 | 171.17683572877843 |
| PNPLA4 | 220.82678252363021 | 191.02502627720412 | 186.79890221660193 | 186.62121922413158 | 214.4549376253301 |
| PNPLA5 | 39.764105318729875 | 40.33594340282643 | 40.040684375391876 | 37.63253275257158 | 45.69500454625527 |
| PNPLA8 | 396.4905903600488 | 355.2393740590692 | 349.8870555074807 | 311.1240029078885 | 324.98128088294754 |
| PPBP | 72.64650762582639 | 142.1668568581839 | 161.87273229753526 | 72.98198992206443 | 50.89937071888025 |
| PPP2CA | 1557.6956428358656 | 1709.013542693484 | 1659.33259832094 | 1541.9681999717623 | 1533.9039072416074 |
| PSMB5 | 704.4555787934885 | 713.2525794568301 | 685.344253489181 | 650.0350493545982 | 693.901787134469 |
| PSMB7 | 646.6630512521712 | 654.4647483216954 | 669.5281270644971 | 620.6965817495743 | 575.8463651717875 |
| PSMD7 | 925.2272286802929 | 965.877703206886 | 1030.3154340094964 | 953.9920179502458 | 882.0649814421417 |
| PTEN | 2558.3339158457225 | 2687.20893522569 | 2589.6698437778423 | 2507.8183540007403 | 2523.5595781948405 |
| PTP4A1 | 34.12516911482791 | 35.24247921174063 | 37.76174979182719 | 32.17078501449165 | 31.652485883706024 |
| RHOA | 9249.301128160785 | 10023.1074448457 | 9469.031489366023 | 9254.728342985545 | 9345.003425535477 |
| SLC2A1 | 1773.142033260506 | 2135.825159823855 | 2066.792715318092 | 1846.7239115762402 | 1815.240773319093 |
| TGFB1 | 82.51392764308918 | 89.35800786561822 | 87.71252544296827 | 87.91765967697835 | 78.64138273809164 |
| TGFB2 | 294.6343746770125 | 309.09912804626515 | 300.81367538436126 | 290.3738640495028 | 241.60366705863555 |
| TGFB3 | 415.64071882587564 | 661.4133754263476 | 637.7383128569605 | 555.0483782444464 | 488.14755565875265 |
| TGFBI | 1386.0415245290467 | 1452.068956637865 | 1128.1850791119891 | 1146.2440835270247 | 829.6951688008138 |
| TSPAN13 | 302.79253030942357 | 317.72910487171987 | 440.21973435752557 | 483.3586625962672 | 276.4897348985241 |
| VEGFA | 203.34623775687365 | 210.27250015826382 | 225.86409890657765 | 217.26583231538822 | 202.50642678054487 |
| VEGFD | 167.51886023792434 | 104.2269980978389 | 109.5671368897309 | 176.82706062040018 | 138.3606906176317 |
| WISP1 | 303.7822840461729 | 454.73037907579084 | 437.12867400961426 | 445.47093162620575 | 369.50354067535005 |Normalized Expression
### Chart: ADO
| Category | BL | 2 | 1 | 3 | 5 |
|---|---|---|---|---|---|
| ANGPTL7 | 531.11397297478 | 431.29167705061076 | 697.111020064787 | 401.7688817202509 | 667.880272566754 |
| APOH | 13.751072279677354 | 14.282772975744498 | 13.871582952381974 | 14.35234242097587 | 13.704760666289534 |
| ARG2 | 154.42344579895263 | 176.09240026538058 | 137.25821618332614 | 137.1729593364718 | 124.0641121220037 |
| ARHGDIB | 2523.058673537378 | 3247.6733203491563 | 3103.428164235871 | 2942.6471090829714 | 2598.366074139333 |
| ARHGEF2 | 113.49029934524225 | 176.34025002518973 | 169.12537546018584 | 150.30961260562796 | 146.05727102349283 |
| BMP1 | 208.95063698847858 | 335.0684756633989 | 312.2554405441211 | 253.74750850951145 | 244.94001589632612 |
| BMP2 | 199.9071332956902 | 175.95843802509697 | 164.13793057818148 | 156.22604680244328 | 167.66101221154037 |
| BMP7 | 210.29515537905854 | 162.20648918872473 | 182.3057146014456 | 189.72336114930837 | 220.33350776137456 |
| CALD1 | 450.2202769386763 | 653.2002526376987 | 623.2773561611084 | 530.0350903149877 | 492.91480680131383 |
| CAMK2N1 | 180.10901106627148 | 215.9583861585363 | 206.8016872967638 | 182.0822700154563 | 178.43854053057248 |
| CCN1 | 373.87444426205525 | 326.68351862721283 | 232.49976693457185 | 209.5830888299641 | 161.56177401359656 |
| CTGF | 768.4391111858037 | 1033.362124484592 | 815.4912858567617 | 735.0844956756418 | 610.0319233399389 |
| ESYT3 | 812.195180573681 | 463.3057349069278 | 503.6298591853344 | 575.9092001932618 | 803.443429577064 |
| FGF10 | 26.743130649435233 | 24.197290838633823 | 22.406628910502256 | 23.187321674203694 | 22.220858850707998 |
| FGF7 | 122.70968326613125 | 248.16501809807653 | 211.15093077188575 | 180.5594189247463 | 148.94832790723856 |
| FGFBP1 | 517.8749916526904 | 525.9476649426347 | 549.5334107789148 | 598.8558536210522 | 514.5842927298097 |
| GPI | 464.49822862043015 | 543.3356919897492 | 516.096405006403 | 503.76411328067235 | 511.6900776095599 |
| LIPA | 333.06853514972113 | 420.0982814573859 | 392.0952270617285 | 390.5993331230603 | 293.1341803039883 |
| LIPC | 45.315325348803505 | 65.22468816673758 | 66.42054510535904 | 60.71120090174164 | 52.15412828957124 |
| LIPE | 163.88488056257142 | 180.2622013403024 | 193.75893960955537 | 193.42478579435422 | 173.81842180865976 |
| LIPF | 31.5517778423051 | 28.87764928542311 | 23.472170811711162 | 27.561199687893563 | 28.63207950524831 |
| LIPG | 250.4056097610481 | 235.61552437033885 | 195.1285464610969 | 220.21311099704076 | 198.00969704272075 |
| LIPM | 498.80605332641215 | 238.83950963728563 | 286.7865926840342 | 355.8549325711243 | 393.4120855398494 |
| NODAL | 33.192099867089006 | 34.50693231658859 | 37.181417724756145 | 35.76716952944421 | 35.88300082430931 |
| NT5E | 115.16615718633291 | 170.076287571392 | 153.5654186989908 | 129.17767403248888 | 133.07013094818248 |
| PNPLA2 | 496.043203645299 | 460.86689649313524 | 459.83769116599825 | 466.46206482099063 | 499.8465455899279 |
| PNPLA3 | 154.38038119602834 | 154.5479668131013 | 162.88081101096708 | 158.21556230908544 | 185.7925757961037 |
| PNPLA4 | 162.49237978569172 | 157.51373297704401 | 162.23366296863978 | 169.88150494529566 | 177.04956977863904 |
| PNPLA5 | 35.132254583887715 | 37.937292675129726 | 42.187062636602114 | 39.04199880634833 | 41.18004792843725 |
| PNPLA8 | 376.4793114060487 | 302.11127562305256 | 298.2667956498713 | 286.9023072345871 | 339.4592016730034 |
| PPBP | 85.3030764639153 | 112.84047883157166 | 84.19132197857908 | 105.7638649778549 | 84.8690547188254 |
| PPP2CA | 1508.558815282367 | 1484.863507916499 | 1520.7633070780907 | 1606.1398252117115 | 1511.83399586536 |
| PSMB5 | 663.9489314888756 | 705.578954107767 | 663.2295201944248 | 649.6655640789255 | 784.2543322874623 |
| PSMB7 | 732.8145076580879 | 762.705279203274 | 741.4445302707727 | 735.8912590205438 | 748.847658592496 |
| PSMD7 | 972.756554700291 | 1021.1227742145323 | 965.549701959376 | 1011.0322801474288 | 958.8136922693876 |
| PTEN | 2841.234813405716 | 2857.257593771769 | 2810.5435125043596 | 2656.758481904693 | 2887.0913887683187 |
| PTP4A1 | 30.44121828654939 | 32.58225246617276 | 30.854621152043112 | 35.690531277144046 | 30.327580378409532 |
| RHOA | 9264.176070899517 | 9755.242896207708 | 9450.05338076902 | 9537.766928365858 | 9368.60338235054 |
| SLC2A1 | 1874.3404170630504 | 2191.8897728994666 | 2011.9146693446492 | 1950.5080490104447 | 2115.4959267089826 |
| TGFB1 | 74.70284467992899 | 85.84186354233674 | 83.85840453854695 | 77.0175935935214 | 77.23987806490766 |
| TGFB2 | 269.94200920086746 | 280.6603030020227 | 299.4860736510517 | 263.1492340752445 | 271.014216058706 |
| TGFB3 | 465.3885150954862 | 877.9062599863835 | 841.8543709666698 | 630.9857136367359 | 530.9327520160218 |
| TGFBI | 1188.9565330897738 | 1654.8506186827137 | 1462.2580568932708 | 1085.9701965803533 | 1158.9411075266942 |
| TSPAN13 | 308.7272701595824 | 391.1560610248511 | 549.6154365087075 | 461.2980169959893 | 329.719640372746 |
| VEGFA | 176.4702778516187 | 243.91949415685895 | 229.79126901277428 | 195.0670716515025 | 198.569032511769 |
| VEGFD | 174.69291974968147 | 133.44733675005352 | 138.1500485247427 | 152.3953734049261 | 172.7894834515265 |
| WISP1 | 352.3880843088117 | 549.0879269061605 | 495.7020558382986 | 422.0224581533964 | 394.59635278151785 |Normalized Expression

## Slide 16
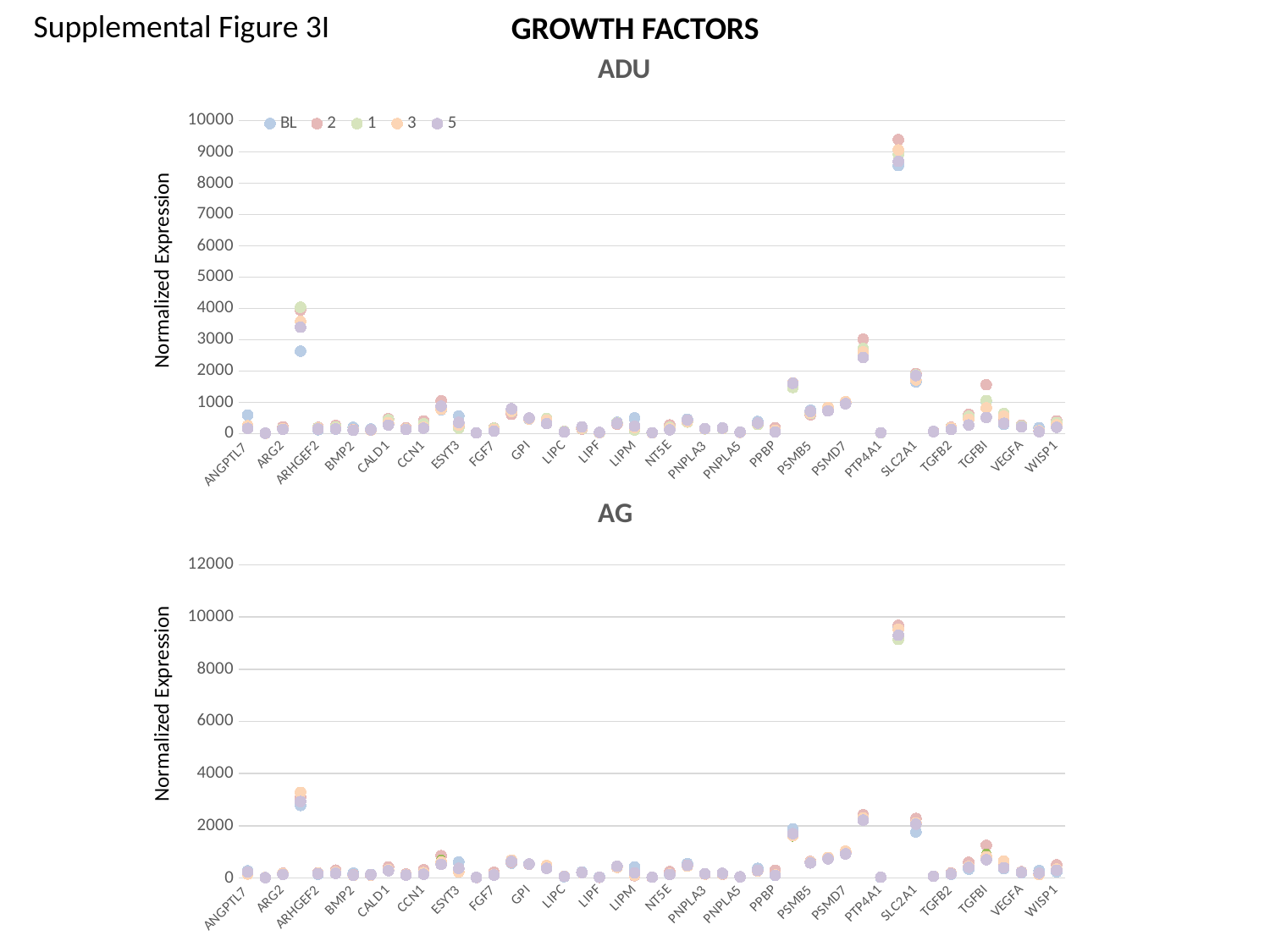

Supplemental Figure 3I
GROWTH FACTORS
### Chart: ADU
| Category | BL | 2 | 1 | 3 | 5 |
|---|---|---|---|---|---|
| ANGPTL7 | 596.7981615274044 | 183.50672309635232 | 243.71155004430906 | 252.25623927226636 | 167.15320003912137 |
| APOH | 12.004987733180691 | 11.57230231399175 | 10.53254477090099 | 11.27257899947273 | 12.243602180588919 |
| ARG2 | 173.26233077292898 | 214.35533310658894 | 157.68724296508125 | 153.18001174459005 | 136.25702325061457 |
| ARHGDIB | 2633.90183357803 | 3948.2776565292183 | 4034.6383290033864 | 3581.562103348797 | 3399.239777169293 |
| ARHGEF2 | 123.85955284436851 | 199.79240698563595 | 185.51307162396054 | 170.031650578329 | 146.8769687546817 |
| BMP1 | 146.87351063911322 | 254.45483465711783 | 209.74928713799883 | 166.3076048240641 | 149.12879099237662 |
| BMP2 | 198.4617818394896 | 125.37435930140967 | 108.72685803619055 | 114.6378492513039 | 106.8997694535742 |
| BMP7 | 144.19362282182536 | 111.3596170171141 | 106.26202138722535 | 105.77764400148273 | 118.23901924869875 |
| CALD1 | 308.4605606911053 | 473.2238679006722 | 437.733604135831 | 346.3840188310647 | 273.16949519029845 |
| CAMK2N1 | 140.98011399184685 | 184.97468912723534 | 147.67514927968315 | 143.40693672617448 | 134.5541378747521 |
| CCN1 | 197.32913108795697 | 407.40812408299394 | 311.50492817338846 | 205.1432424199302 | 173.72955956564522 |
| CTGF | 762.22261395939 | 1046.5882437477887 | 874.2044905250124 | 777.1525209158928 | 874.4019536870705 |
| ESYT3 | 560.0728882358746 | 231.6154717484768 | 172.42551170638123 | 271.5474939885496 | 357.08299977956096 |
| FGF10 | 20.24905972716766 | 19.931607815271732 | 19.448518121332473 | 21.389474914641067 | 21.38361444302284 |
| FGF7 | 97.96016436953444 | 177.99488859451594 | 167.7108701240997 | 137.97400499425336 | 77.63986434556719 |
| FGFBP1 | 724.4715768732199 | 613.4696974789304 | 708.6874659625173 | 717.0645271941913 | 791.878695716424 |
| GPI | 463.2101239893607 | 484.0381170234424 | 489.88313089497206 | 468.14411912319406 | 497.88330442093684 |
| LIPA | 358.11421445043436 | 460.7998932126771 | 482.8965759815226 | 433.5118862020191 | 319.9472508597794 |
| LIPC | 46.59264561702306 | 64.20286769942402 | 62.11162891806292 | 54.417146616843986 | 49.15289851314842 |
| LIPE | 182.65563011656897 | 145.8561424010328 | 162.76305258483504 | 176.4458145814202 | 214.17232468561613 |
| LIPF | 35.19361991303338 | 26.17407496999957 | 29.94439725861682 | 31.52071143080453 | 40.082523419569334 |
| LIPG | 361.9581405619378 | 297.6478035776139 | 342.5347120635116 | 326.91681751287047 | 330.2227674363729 |
| LIPM | 496.33431730111704 | 165.13025635669317 | 117.27259121376915 | 195.456389315752 | 235.84315568369934 |
| NODAL | 25.90263644094712 | 28.29564084270369 | 24.521591098548424 | 26.669021487022498 | 29.32199454473668 |
| NT5E | 169.01720031994532 | 276.8347925040372 | 201.8163255316667 | 161.64860528033677 | 118.79919691209622 |
| PNPLA2 | 455.94251232500613 | 399.0864834121944 | 367.7878466808288 | 391.1912140597942 | 433.530898903341 |
| PNPLA3 | 159.24264380600007 | 154.41535930082927 | 152.3551707829458 | 152.72907049444257 | 156.83140089428974 |
| PNPLA4 | 181.05433954312974 | 156.07445906063276 | 154.59670714059814 | 172.39614189390602 | 176.07981650428536 |
| PNPLA5 | 38.10262628422757 | 41.174897870730966 | 38.66470018654537 | 39.22441464946198 | 43.28462974283109 |
| PNPLA8 | 385.1828812773914 | 289.0284476270798 | 290.51890469032156 | 329.34111343098783 | 335.1271995490204 |
| PPBP | 91.55983472508802 | 183.53589992954315 | 69.01565378676766 | 101.45302598805779 | 54.1224682650155 |
| PPP2CA | 1609.5630730932908 | 1477.0894545527353 | 1473.0728384915399 | 1618.7909095665814 | 1607.9783702528632 |
| PSMB5 | 743.2179812745368 | 604.529926649046 | 645.998422206255 | 665.4158103714997 | 708.2729995747479 |
| PSMB7 | 803.0106344755827 | 799.7657308378821 | 819.1280148945439 | 836.1909367566515 | 727.218235615423 |
| PSMD7 | 953.3819822366992 | 975.0448443068547 | 986.1365632645151 | 1018.3510841019431 | 955.2326805568357 |
| PTEN | 2515.368487611497 | 3011.6483401564624 | 2714.9245369258624 | 2605.720415996123 | 2426.6017562830198 |
| PTP4A1 | 27.69571042011435 | 23.963185206524717 | 25.325290274917066 | 28.35275754330595 | 28.64049118648293 |
| RHOA | 8565.90779246581 | 9386.472957605634 | 8919.92842664454 | 9061.34975525328 | 8696.110282539308 |
| SLC2A1 | 1652.1930757613063 | 1918.117119808542 | 1873.1135661006988 | 1715.8824390643429 | 1853.462562541734 |
| TGFB1 | 61.545839368480785 | 68.20081871424667 | 66.2578942892511 | 64.81545432128739 | 65.60236331042937 |
| TGFB2 | 163.62405829254715 | 199.503676832246 | 188.44326442941338 | 185.10889139509618 | 130.0649025497102 |
| TGFB3 | 450.34623130721656 | 615.5871234917713 | 548.7955290132188 | 459.0480982689773 | 275.79304144033404 |
| TGFBI | 1028.9263634619826 | 1561.3227612144963 | 1055.1398710818576 | 820.0387584767021 | 515.5193669110595 |
| TSPAN13 | 291.28401448961927 | 484.16480597455495 | 638.777820651643 | 556.8615643365029 | 328.38681865785867 |
| VEGFA | 228.63476452179242 | 264.53148569787874 | 237.99309933159708 | 217.42693169422918 | 213.37154455648917 |
| VEGFD | 187.03301252318934 | 81.51553234117372 | 82.18035860223355 | 86.80686855201222 | 63.48969555513674 |
| WISP1 | 209.8001627345194 | 406.82605095245395 | 352.55086507284136 | 280.484384645162 | 203.33518377029935 |Normalized Expression
### Chart: AG
| Category | BL | 2 | 1 | 3 | 5 |
|---|---|---|---|---|---|
| ANGPTL7 | 268.2478937041717 | 200.22010836386164 | 168.46068796482498 | 149.03702181268667 | 228.6434324388836 |
| APOH | 10.272224193108293 | 9.379165765697108 | 11.677348831102409 | 10.998323095704913 | 9.798218498709243 |
| ARG2 | 174.01545606885097 | 178.9661657809853 | 154.6380468049059 | 167.3231126031275 | 135.1488346954151 |
| ARHGDIB | 2777.474576784459 | 3096.95876714507 | 2893.0396705901985 | 3279.339067596567 | 2927.280571620525 |
| ARHGEF2 | 145.657372928339 | 192.92598480712948 | 169.01324567289623 | 173.57866615407963 | 165.57452638972578 |
| BMP1 | 183.100673881756 | 299.22606082774917 | 227.2936927851337 | 215.89000614149302 | 175.96775025956694 |
| BMP2 | 178.65912953890006 | 117.84079058968945 | 113.63942460308404 | 99.62059905323456 | 103.91124768647983 |
| BMP7 | 131.44113960195207 | 108.46019434544736 | 106.19914179932157 | 110.51873171553152 | 124.90023213778613 |
| CALD1 | 276.92490039351986 | 420.56209521477854 | 321.16996984648574 | 315.5356662913882 | 278.20618328408796 |
| CAMK2N1 | 114.51116144117493 | 146.54875812032523 | 123.6817317522669 | 117.32542746391606 | 108.65150830348045 |
| CCN1 | 234.4923271580216 | 312.33634727840865 | 215.95213113532708 | 202.17378353030753 | 140.4805209021847 |
| CTGF | 554.3184189810103 | 852.6501973169002 | 708.6077283517026 | 609.783494609463 | 519.0138297908769 |
| ESYT3 | 606.1604898515857 | 234.6204179552833 | 252.90081343400445 | 225.9106188480291 | 367.0626472355476 |
| FGF10 | 20.20013911919597 | 17.806911831028497 | 20.547285057128864 | 21.60592654907739 | 18.792103068761683 |
| FGF7 | 113.40761629615868 | 215.62286712243505 | 128.3160859265463 | 132.00667990080478 | 113.45536875488756 |
| FGFBP1 | 564.8860757036286 | 598.6735461555442 | 658.3957297239674 | 677.9441623292786 | 628.8459960080414 |
| GPI | 536.7205046768711 | 527.8876223003331 | 536.0880592490964 | 530.5749802454507 | 532.3416697824964 |
| LIPA | 404.6223278479466 | 456.7640954638212 | 436.5223628513103 | 470.72751140107255 | 367.2394213643796 |
| LIPC | 49.16409021653655 | 66.13371408059139 | 54.58209049698318 | 60.56381666098159 | 55.55010121734183 |
| LIPE | 224.0800392186294 | 204.3811785166564 | 201.4125769957739 | 202.94616553862122 | 208.02012325816258 |
| LIPF | 28.21957377225462 | 21.470941333594887 | 25.369222338884825 | 24.37901242104983 | 26.50242399209698 |
| LIPG | 436.3936829494444 | 406.1295030013974 | 377.5325105028908 | 409.3743319808006 | 444.8834984136911 |
| LIPM | 420.92799478415037 | 85.63473104936449 | 136.57169712323358 | 110.55022478647487 | 192.2279993574145 |
| NODAL | 26.713428122020808 | 26.136229978045336 | 26.45609400270689 | 27.335262614305623 | 27.14960439548587 |
| NT5E | 130.14857868067116 | 239.80301328572705 | 177.1573273524641 | 143.29460129631053 | 135.70320496281573 |
| PNPLA2 | 546.2717614460398 | 487.1237888987212 | 468.5427147851496 | 450.14243483623954 | 480.7640496609521 |
| PNPLA3 | 159.22206603245075 | 146.32239763709575 | 143.63509334885907 | 151.68837713440752 | 155.71072303095082 |
| PNPLA4 | 154.65082982254572 | 145.52999440283932 | 138.74273886616007 | 151.68436341246587 | 175.0294375131253 |
| PNPLA5 | 40.276623053871326 | 40.27726052661356 | 37.53446519676127 | 40.09807152442245 | 42.464078293546926 |
| PNPLA8 | 366.57133660200367 | 264.8476842196218 | 281.74069563412195 | 274.1775191305753 | 290.84025019518026 |
| PPBP | 97.40194928830952 | 295.95440502190064 | 97.61537948265898 | 109.293415535206 | 102.52123434774198 |
| PPP2CA | 1873.5053613873981 | 1633.3762760313027 | 1577.158409830291 | 1621.576292396568 | 1694.3774180975681 |
| PSMB5 | 615.2438850971126 | 628.2208263371186 | 576.0873537217024 | 599.6725135319786 | 573.6477275550446 |
| PSMB7 | 766.673600033272 | 769.3190925846825 | 768.0658522775486 | 782.9969615390495 | 729.2991944183681 |
| PSMD7 | 1000.8952052285414 | 969.1056105393922 | 973.6939488512364 | 1022.0808506946954 | 921.4773008952702 |
| PTEN | 2301.159881490515 | 2414.273054663352 | 2174.718479306084 | 2298.2905583905927 | 2212.8329835822265 |
| PTP4A1 | 24.751024695982537 | 27.122365654709814 | 31.881143157462585 | 27.689073032807347 | 27.892373076997668 |
| RHOA | 9552.733500605065 | 9681.023303039998 | 9143.402420647699 | 9535.216758827773 | 9302.370609653495 |
| SLC2A1 | 1761.3401529718287 | 2279.757407549926 | 2097.134369524182 | 2108.9534501160865 | 2061.278084095442 |
| TGFB1 | 65.81706624190991 | 68.49734443704615 | 63.590174896974546 | 67.38808619111771 | 65.19068479319677 |
| TGFB2 | 144.12053291428973 | 186.81282448106 | 150.84089304550918 | 167.99510322239192 | 153.12091469357063 |
| TGFB3 | 336.99240368377093 | 597.6494941740485 | 469.775217069721 | 434.909795007672 | 408.96911005158904 |
| TGFBI | 701.5911969324017 | 1250.8553398648712 | 936.6120992617616 | 817.4542120664761 | 697.8475515105873 |
| TSPAN13 | 350.0991919393089 | 476.53115364607646 | 634.0944915948286 | 645.3858164536048 | 391.9115759389111 |
| VEGFA | 194.3183725089492 | 235.52619450610675 | 226.16622413028992 | 224.85678444110715 | 217.04427996547957 |
| VEGFD | 277.6350126518292 | 141.98163658150807 | 134.95811695667697 | 125.66771874234912 | 181.05527769775952 |
| WISP1 | 233.72784524045403 | 500.3748831782242 | 346.95549704422183 | 335.740947433754 | 297.8170636544362 |Normalized Expression

## Slide 17
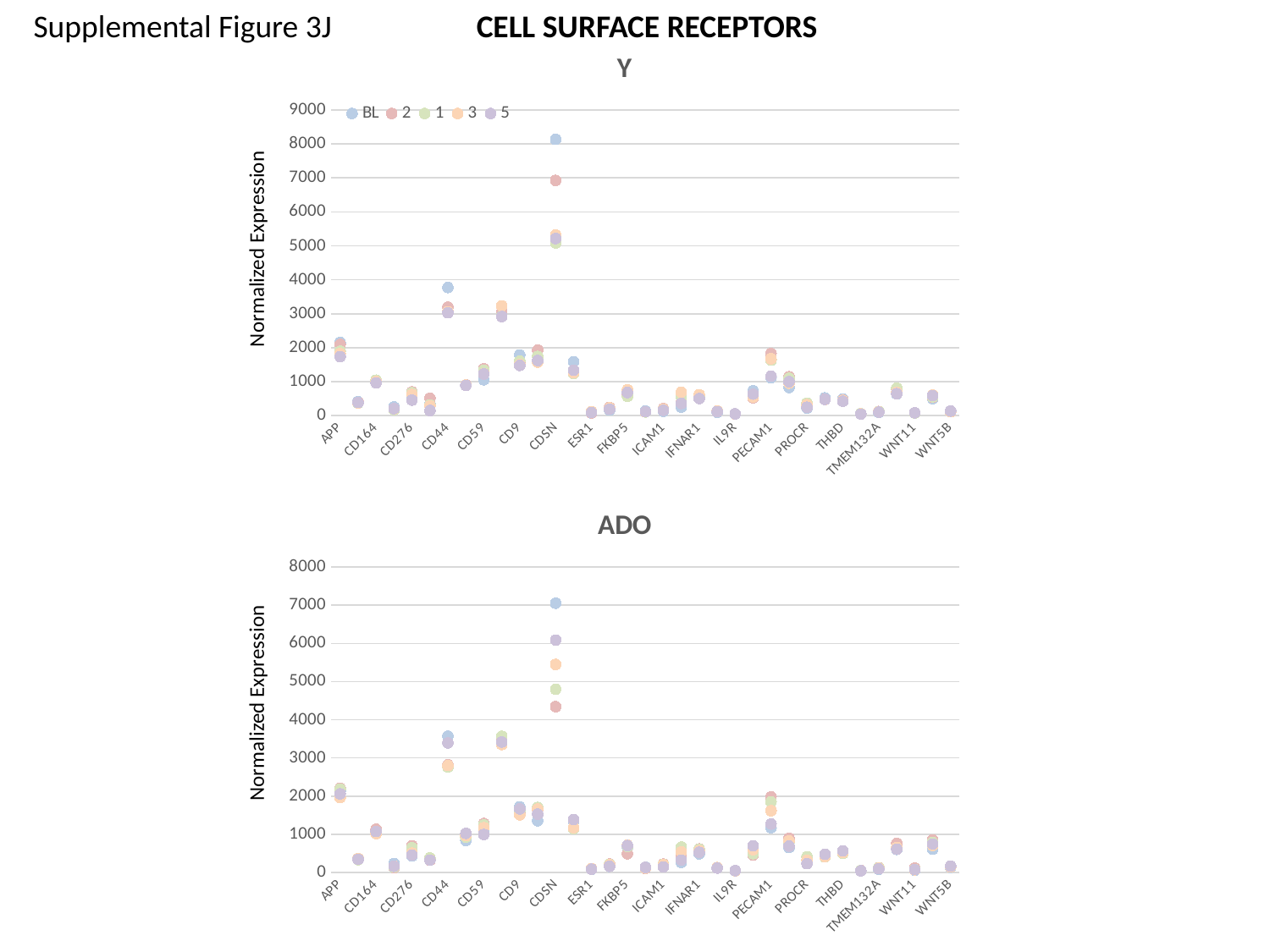

Supplemental Figure 3J
CELL SURFACE RECEPTORS
### Chart: Y
| Category | BL | 2 | 1 | 3 | 5 |
|---|---|---|---|---|---|
| APP | 2152.641102720199 | 2094.7817529702934 | 1897.1833163215965 | 1825.97520794531 | 1735.6975561391805 |
| BCAP31 | 409.3415642734182 | 388.00379521068385 | 370.65982699048067 | 373.62733448176067 | 385.59688721345395 |
| CD164 | 997.3680213846742 | 1017.4557564130153 | 1043.2046381815474 | 1001.5565706864537 | 962.5313194210962 |
| CD207 | 251.85048264262136 | 192.15146591665484 | 172.3576586083954 | 203.02996414314714 | 204.76174365529369 |
| CD276 | 513.0627652738701 | 698.3191214997927 | 670.9918278132508 | 623.1402935359308 | 454.9176841799583 |
| CD36 | 339.00179872605327 | 510.76403018626127 | 316.2609743491676 | 287.4129256498561 | 149.35782156387035 |
| CD44 | 3772.057828963385 | 3187.97151237926 | 3058.643454193567 | 3059.547368163154 | 3028.130537594535 |
| CD46 | 904.2624121235663 | 903.9000655568584 | 895.5748634116842 | 898.9887196511786 | 887.4901764884803 |
| CD59 | 1053.6907103453873 | 1372.749355964661 | 1335.7940183002947 | 1235.791369082254 | 1225.2526212727478 |
| CD81 | 3219.7800867413553 | 3051.0932984690808 | 2930.1335962599805 | 3234.575772011424 | 2917.427055681399 |
| CD9 | 1784.4048848438986 | 1570.9953702399866 | 1602.9571979124894 | 1502.8948926802595 | 1477.3345249982146 |
| CD99 | 1605.4757793136284 | 1927.1972319387864 | 1741.00727302839 | 1576.1166976761813 | 1620.9335128264158 |
| CDSN | 8135.290608516818 | 6924.281824475436 | 5087.666029954697 | 5317.561285773776 | 5213.78941708228 |
| EGFR | 1587.0380351512185 | 1236.0108320931151 | 1237.4600516224566 | 1264.535774662333 | 1335.4273053836985 |
| ESR1 | 75.60248090897639 | 78.71975570219196 | 119.34170457474595 | 119.22688018981648 | 91.66274173606732 |
| F2R | 161.83067759711204 | 241.0811270363714 | 223.81975727966523 | 224.16632637707056 | 188.26706298253904 |
| FKBP5 | 617.6381575252899 | 646.2008771757894 | 567.2262568244835 | 764.8346743746869 | 678.9946124354028 |
| FZD7 | 133.52409491598397 | 107.8420166863884 | 106.63186705840413 | 109.81360477047058 | 113.82664286191562 |
| ICAM1 | 129.91065768103556 | 201.57617822282543 | 186.90867801292814 | 187.38418129696208 | 157.37818979943873 |
| ICAM2 | 250.99131704721003 | 381.51335354965494 | 546.2313702981191 | 689.7113141987919 | 357.63555141883666 |
| IFNAR1 | 513.4768678840185 | 601.7007846375996 | 595.6676737026369 | 612.3116660381919 | 501.2993132370575 |
| IGFLR1 | 99.45224115575834 | 120.91869566505483 | 120.29447184508848 | 134.16716935078108 | 118.75218601650691 |
| IL9R | 46.04875138241519 | 46.75619926398907 | 49.536529680451864 | 47.81631758469923 | 50.78916600670404 |
| OCLN | 725.7785976629016 | 524.6093451322503 | 568.2971975821224 | 549.8889409014206 | 632.6599014731087 |
| PECAM1 | 1113.890022864428 | 1825.8572582907946 | 1630.4157091789848 | 1672.8628335010626 | 1163.2995471087565 |
| PLEKHA1 | 826.1678803865584 | 1145.5406873269956 | 1099.3354001198304 | 954.3910796325488 | 996.8936983480697 |
| PROCR | 219.6260564810479 | 341.8505082470281 | 369.31843094874733 | 321.60097904799704 | 245.9589050129843 |
| SOS1 | 520.5005762778633 | 485.1139271985117 | 480.7978905242827 | 476.57509145503053 | 478.6816442036613 |
| THBD | 494.2425983312121 | 458.8643213158842 | 426.2222790979111 | 447.5315983993462 | 423.3758632830541 |
| TMEFF1 | 44.586855555761225 | 56.70654529341718 | 56.16790429916538 | 50.07603222734205 | 45.78591385310247 |
| TMEM132A | 97.35116816271852 | 120.65539442558803 | 118.59407578550014 | 109.64204427148378 | 105.63587533393925 |
| TNFRSF1A | 636.5760925019086 | 777.6579888144923 | 807.6433395819855 | 686.1101696883748 | 645.2572021593413 |
| WNT11 | 73.26841335021582 | 73.4523405312568 | 72.98595338762959 | 83.01992605789388 | 82.90131364800861 |
| WNT5A | 498.58353952452893 | 537.2483654331577 | 554.3386890880779 | 605.9991931064504 | 587.9522663942204 |
| WNT5B | 136.05512494809565 | 119.43490998314034 | 117.02330661193976 | 110.66018238290553 | 133.36185248846525 |Normalized Expression
### Chart: ADO
| Category | BL | 2 | 1 | 3 | 5 |
|---|---|---|---|---|---|
| APP | 2123.073766091438 | 2208.059645670851 | 2185.4725446964967 | 1962.2764921640398 | 2060.0951472666206 |
| BCAP31 | 363.4773722629051 | 333.94220608443015 | 336.45108561120776 | 366.760723570452 | 347.22949337972796 |
| CD164 | 1077.3518904428438 | 1135.7699191346724 | 1064.9323488009784 | 1019.2197088138265 | 1072.752580285819 |
| CD207 | 237.05430271341825 | 126.98624957284574 | 141.44395655669666 | 164.92424533669407 | 163.22893496017292 |
| CD276 | 430.5904930746738 | 705.7052854605316 | 650.525560860817 | 509.3714833340269 | 456.88430961041377 |
| CD36 | 359.21240346395103 | 323.3041803654225 | 381.5575154250436 | 329.35825976447006 | 327.3346308890417 |
| CD44 | 3568.0587743179362 | 2822.8567032544224 | 2764.4013337513506 | 2801.124564737492 | 3392.4036700605316 |
| CD46 | 840.024057593096 | 950.5418564932952 | 926.2219739129994 | 966.8914703168125 | 1025.2612552618075 |
| CD59 | 1036.6634143856306 | 1283.0268661865994 | 1243.443374115404 | 1174.3569397788024 | 998.6521877359555 |
| CD81 | 3524.1790934112028 | 3534.788187748541 | 3565.543221642611 | 3350.2269586577277 | 3417.778224556419 |
| CD9 | 1719.7440234531482 | 1547.8658479166163 | 1601.7842268572922 | 1509.2139847742726 | 1665.3061454702308 |
| CD99 | 1353.8650293758517 | 1653.1532863582527 | 1704.830015586713 | 1665.9934257839432 | 1528.1125734736343 |
| CDSN | 7050.721419291182 | 4342.848160864665 | 4800.7163690208545 | 5447.198354351965 | 6086.488071599 |
| EGFR | 1347.8319879760672 | 1157.78861346219 | 1140.7222467987542 | 1173.3207328096291 | 1388.0203079544526 |
| ESR1 | 84.17088173671526 | 91.90975313425287 | 105.71676684172795 | 105.45396876842443 | 87.86771606151528 |
| F2R | 162.18402141660138 | 223.16141920991853 | 214.63668490999652 | 199.42455314993424 | 162.78799081629325 |
| FKBP5 | 663.2886153555293 | 493.14223343523435 | 683.1695107090095 | 718.4729447648535 | 707.0694335973229 |
| FZD7 | 139.71477957747234 | 106.38767512676496 | 118.04611186257529 | 117.05226678092157 | 135.84567538353127 |
| ICAM1 | 165.8856696418987 | 221.7241350068721 | 209.5722932554381 | 201.39304249517605 | 149.70587944770432 |
| ICAM2 | 266.70123362776513 | 420.690599474174 | 665.8410987107637 | 542.3727347238992 | 327.4897939165965 |
| IFNAR1 | 488.4913099788781 | 619.2144709986037 | 610.1420283575975 | 574.8728509566369 | 531.1218696639811 |
| IGFLR1 | 117.86581486535952 | 126.78650582145045 | 130.72213344761076 | 130.32885371977602 | 117.56713331109258 |
| IL9R | 49.842006739199064 | 46.45416754341516 | 46.762035839548815 | 45.53397752987468 | 50.589592032399956 |
| OCLN | 701.6771097715721 | 457.8989631171652 | 493.539522389028 | 600.437404759366 | 700.9344197734398 |
| PECAM1 | 1174.6139472871391 | 1982.0495123976898 | 1851.7830631645015 | 1619.2878644962689 | 1270.8207769323344 |
| PLEKHA1 | 655.6486847536792 | 898.2812373429105 | 805.0781592512262 | 829.4593459460461 | 693.6039121255334 |
| PROCR | 238.81209706258198 | 412.75071267242186 | 412.63148987414183 | 322.60193689689595 | 231.6599763503007 |
| SOS1 | 434.9989696323598 | 413.57972240064805 | 407.36824958647315 | 417.05453282375726 | 476.71234029451756 |
| THBD | 568.4877714621193 | 529.2877977826381 | 496.7398157743838 | 518.9044267866252 | 572.2000086893972 |
| TMEFF1 | 51.70097234890334 | 54.21182070109206 | 49.37232773914315 | 45.25395811532749 | 45.42605662978659 |
| TMEM132A | 87.4221093010268 | 129.2801580464378 | 125.92255409110356 | 111.8103617376745 | 99.43235682780261 |
| TNFRSF1A | 605.9831328190517 | 761.7854158548832 | 646.2692501889866 | 659.3558029001701 | 613.2829691004644 |
| WNT11 | 61.69132912328295 | 118.17768838740092 | 85.36576627010385 | 71.8942143737907 | 75.58251511160388 |
| WNT5A | 608.3776665259707 | 856.666774841217 | 789.049576228564 | 712.1756630917321 | 749.9367322378652 |
| WNT5B | 135.1656801068031 | 147.22411084397217 | 156.40113112107952 | 146.97129717554847 | 170.33361594685798 |Normalized Expression

## Slide 18
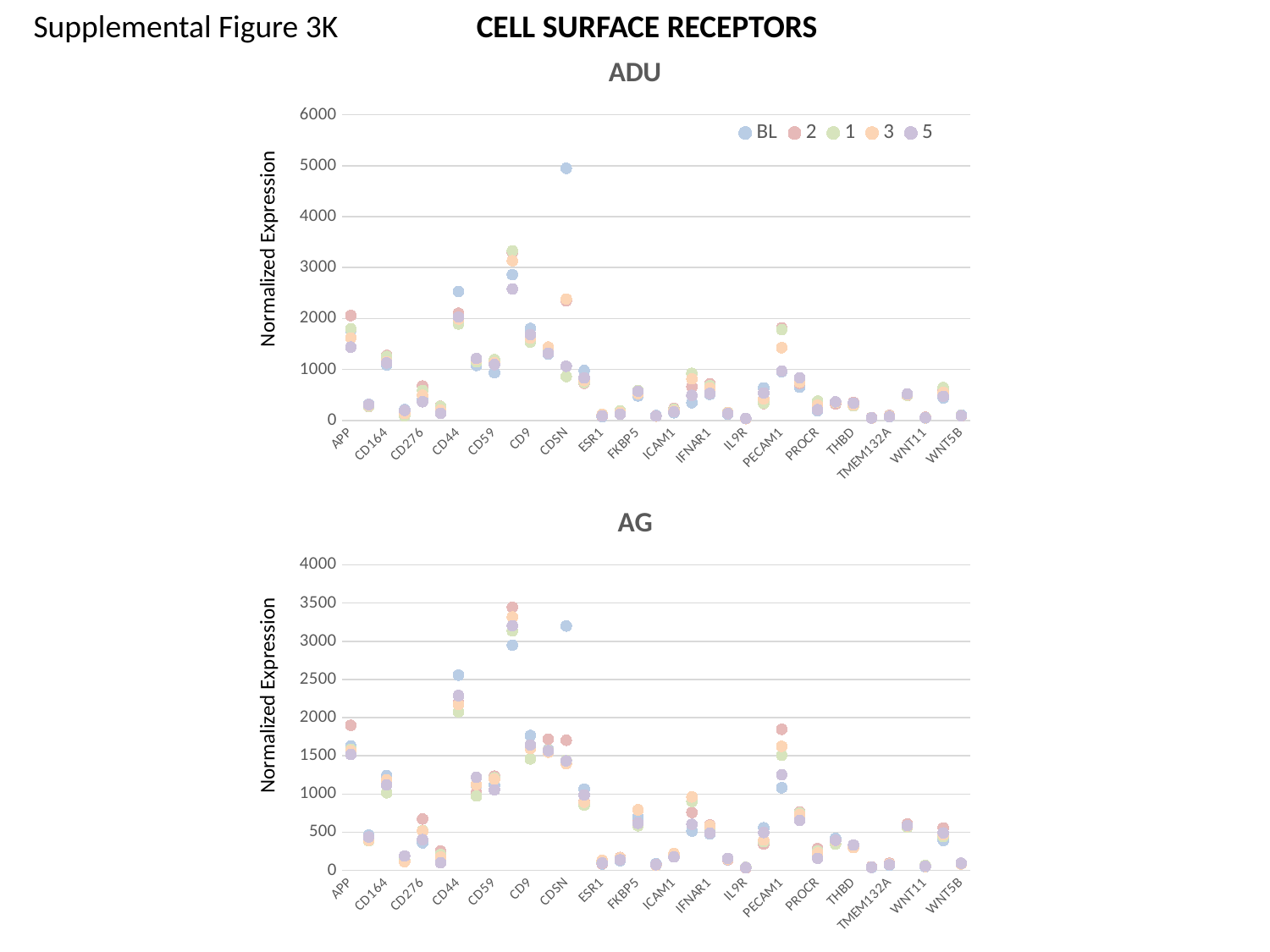

Supplemental Figure 3K
CELL SURFACE RECEPTORS
### Chart: ADU
| Category | BL | 2 | 1 | 3 | 5 |
|---|---|---|---|---|---|
| APP | 1770.3137948540964 | 2057.0485887637155 | 1798.2076624871388 | 1617.4798533202372 | 1437.6575287161493 |
| BCAP31 | 313.39618109374186 | 270.1222504083922 | 274.64123187272 | 295.91141458833437 | 309.09398114871385 |
| CD164 | 1086.9925460377522 | 1273.360427911351 | 1254.5703586030013 | 1152.187757847584 | 1128.7321203808172 |
| CD207 | 211.3356239412983 | 96.80689507687842 | 86.27190054406128 | 135.59884753063096 | 195.86756446692237 |
| CD276 | 416.78310281299713 | 674.5339345842144 | 586.5185118712764 | 493.12638934813157 | 365.17321674652635 |
| CD36 | 219.44774006355217 | 277.05663169135215 | 274.2413388632859 | 200.52108525631078 | 138.94630562300708 |
| CD44 | 2529.493413121362 | 2102.9330480720037 | 1891.2179825290004 | 1990.1791955176923 | 2027.506550604796 |
| CD46 | 1078.6875429778245 | 1164.4792035877333 | 1147.198954305287 | 1197.7560913876123 | 1214.1426212701037 |
| CD59 | 936.2855799914079 | 1140.14072323392 | 1191.1630881429478 | 1129.3209655359933 | 1094.8601717342704 |
| CD81 | 2860.5684110754823 | 3298.4753531409797 | 3327.828870700655 | 3133.465819034617 | 2579.700582589245 |
| CD9 | 1803.7115984492748 | 1599.515556370319 | 1537.14068010958 | 1614.263832050781 | 1684.4033157173737 |
| CD99 | 1299.0111066233565 | 1328.3159729402528 | 1356.22720618305 | 1437.2427226460209 | 1319.0593400939247 |
| CDSN | 4947.48557290323 | 2346.7244596310015 | 861.7317415261748 | 2382.6890533240694 | 1062.355128361882 |
| EGFR | 978.2405268798232 | 727.4468679305379 | 743.5741971486256 | 780.0606470416509 | 833.8077556501017 |
| ESR1 | 71.169737206667 | 83.7758693683318 | 117.27174368358777 | 114.79379125242725 | 87.98711332671539 |
| F2R | 121.11094157164808 | 170.83515338848687 | 187.59403439222024 | 153.1940798172607 | 123.48285659500158 |
| FKBP5 | 479.27021061111674 | 523.9845934876719 | 587.7868980510016 | 527.4110338254892 | 572.2698560701101 |
| FZD7 | 94.43829756528588 | 86.63848235940628 | 82.65295811169727 | 82.94410798362253 | 84.01675368565282 |
| ICAM1 | 148.65866442902518 | 233.7259562285584 | 213.46175211847856 | 185.33076764228358 | 166.6227694809204 |
| ICAM2 | 346.19780839817565 | 659.6832378381694 | 922.8978774355112 | 814.8323358844792 | 489.85522518460857 |
| IFNAR1 | 512.1628997755789 | 717.2572218831106 | 679.3333215895813 | 638.4159869778076 | 528.6546381616719 |
| IGFLR1 | 120.96159340025793 | 143.29995771534763 | 157.67954998516956 | 150.5325135402051 | 132.5829482252957 |
| IL9R | 38.78002808683614 | 36.89863699882926 | 35.31765522837663 | 32.77251151997122 | 37.51693781234241 |
| OCLN | 636.9042299504794 | 330.16354205880737 | 335.01295062283236 | 421.45738567259303 | 537.9928736163141 |
| PECAM1 | 952.7933712579668 | 1816.759437881066 | 1781.0820860664903 | 1426.3706546008975 | 966.7550495492537 |
| PLEKHA1 | 650.049946030916 | 727.8547395540586 | 750.4543968745813 | 747.4018346638507 | 833.0323474361085 |
| PROCR | 186.73278325911667 | 357.25200465490997 | 376.56033644603343 | 295.71376131434204 | 208.4660933775752 |
| SOS1 | 358.2054144197033 | 321.4175319921817 | 351.47098705274027 | 354.95708221736953 | 364.2955922720474 |
| THBD | 332.76628697321263 | 352.1220739314424 | 283.0102051499027 | 300.61496259669354 | 339.7066137794281 |
| TMEFF1 | 51.12698144900755 | 55.66463497038727 | 49.29070366207018 | 43.305671098651516 | 46.97730855245063 |
| TMEM132A | 71.55589164289293 | 96.94869726452492 | 91.16905978415322 | 80.86074997588196 | 76.3613020771275 |
| TNFRSF1A | 514.3605698148373 | 487.80631986455046 | 491.9958766415373 | 506.10470096394397 | 518.4085551390148 |
| WNT11 | 46.988381444205245 | 62.720728023320945 | 54.72212315631204 | 48.74498500828557 | 50.62284764005043 |
| WNT5A | 440.98255688139284 | 589.0528002782016 | 644.2389008602912 | 549.2645498508648 | 471.9900902284448 |
| WNT5B | 103.59819550428838 | 89.34777675819831 | 93.7200643229521 | 90.4494298000706 | 91.7229309411743 |Normalized Expression
### Chart: AG
| Category | BL | 2 | 1 | 3 | 5 |
|---|---|---|---|---|---|
| APP | 1629.284777904847 | 1899.2117884726265 | 1586.444933496295 | 1565.8369198283149 | 1518.429060556674 |
| BCAP31 | 461.74589514125324 | 387.7935793823094 | 386.82026911182686 | 396.03651670528586 | 438.3420698578185 |
| CD164 | 1242.3826973037762 | 1176.1170215637137 | 1016.888027477837 | 1186.6660230061789 | 1119.6240842123332 |
| CD207 | 189.08184194811906 | 124.87342233396498 | 133.75060527816376 | 115.27343284512874 | 189.91585936427222 |
| CD276 | 360.9725346877281 | 674.013832776915 | 523.6290413135224 | 515.5005707914601 | 403.2433408440469 |
| CD36 | 196.63567284492706 | 253.98102815673514 | 208.1621864487899 | 175.89694072968803 | 101.07081374720207 |
| CD44 | 2556.4282970077143 | 2198.602439893991 | 2076.6744432725404 | 2173.503591602221 | 2288.5475649401724 |
| CD46 | 1109.239948412062 | 1020.9279218067592 | 973.9232387608126 | 1124.0721856015216 | 1220.1894065336553 |
| CD59 | 1118.5126299123838 | 1233.453429943149 | 1211.1673875849485 | 1195.2595400972557 | 1055.7480615788943 |
| CD81 | 2949.0189744637814 | 3444.5969094011043 | 3135.1266254888715 | 3317.592289968034 | 3203.6778539098837 |
| CD9 | 1765.866580163162 | 1605.6444417767377 | 1459.204465095537 | 1594.5587131194288 | 1643.1535395032874 |
| CD99 | 1581.3555176110629 | 1718.2522703779405 | 1572.7283166916263 | 1547.7545916509685 | 1566.347976216987 |
| CDSN | 3199.4533592358985 | 1703.5333835226197 | 1441.76884577711 | 1397.5486169544074 | 1434.2296835073448 |
| EGFR | 1065.0415567225498 | 903.8310065249511 | 856.0423445978244 | 893.702339659158 | 988.4639081624799 |
| ESR1 | 84.47109261694638 | 88.00626328305876 | 116.32146723535102 | 132.30440035481882 | 96.7850667246422 |
| F2R | 128.1057961757014 | 170.8930041324908 | 162.50222216722517 | 162.278064817864 | 138.79108532463755 |
| FKBP5 | 701.846562843321 | 622.3855252860244 | 584.9266493995934 | 793.0681008662343 | 617.4124273551975 |
| FZD7 | 87.3401531806479 | 77.50987539347452 | 74.02741144809049 | 68.82276399579735 | 75.24377250053169 |
| ICAM1 | 177.13843300130827 | 217.37614644665183 | 209.15603852734915 | 221.36426145693738 | 176.56029157090683 |
| ICAM2 | 515.9185184222325 | 759.5171315357542 | 902.6834508673833 | 962.2903606288073 | 603.7842102897432 |
| IFNAR1 | 477.3121327545641 | 595.0495075221546 | 516.9209885361878 | 584.1795544118478 | 489.17173020665336 |
| IGFLR1 | 139.63288188260785 | 141.62681093808393 | 158.19608999184294 | 149.6091188290011 | 158.5495491311516 |
| IL9R | 35.64385235650161 | 32.8460754958682 | 41.10507340425063 | 37.09552011873532 | 38.37344028900761 |
| OCLN | 557.9822555812477 | 346.36650590649964 | 377.2468109128488 | 398.5903222003614 | 497.42182921993106 |
| PECAM1 | 1082.2744837633763 | 1847.4132878161026 | 1507.8997424147763 | 1623.99135615512 | 1253.1394673091022 |
| PLEKHA1 | 663.9486024510372 | 765.5836196504779 | 750.7185614458009 | 733.9622330914978 | 656.0922856767314 |
| PROCR | 160.57833632207388 | 286.35647613714747 | 251.2175176379117 | 227.6894659383575 | 158.31761366270408 |
| SOS1 | 420.4355878146925 | 347.4240890576925 | 345.85679990691017 | 388.29504707406454 | 394.50884645458524 |
| THBD | 317.4602984846208 | 331.283308667974 | 306.87901429511084 | 299.24120809335056 | 333.0686230503659 |
| TMEFF1 | 39.05883164807886 | 48.289587429227176 | 45.058297707281035 | 42.79868477445283 | 41.75824966581754 |
| TMEM132A | 69.2361643057032 | 94.30572317470738 | 82.26427499388713 | 83.26152938771912 | 76.40619654054413 |
| TNFRSF1A | 584.2100645582664 | 609.5319699272244 | 566.6928901473217 | 585.0283576388933 | 586.7365528928066 |
| WNT11 | 48.65570810908949 | 64.35229807156057 | 65.70864998158396 | 52.60295719008035 | 55.4178000780514 |
| WNT5A | 393.04155873429437 | 557.4814471059585 | 442.7901988543848 | 468.4218103278902 | 491.7545485662465 |
| WNT5B | 94.5053464421268 | 85.63844362128482 | 85.81741827968214 | 85.96256226374854 | 94.55322244636261 |Normalized Expression

## Slide 19
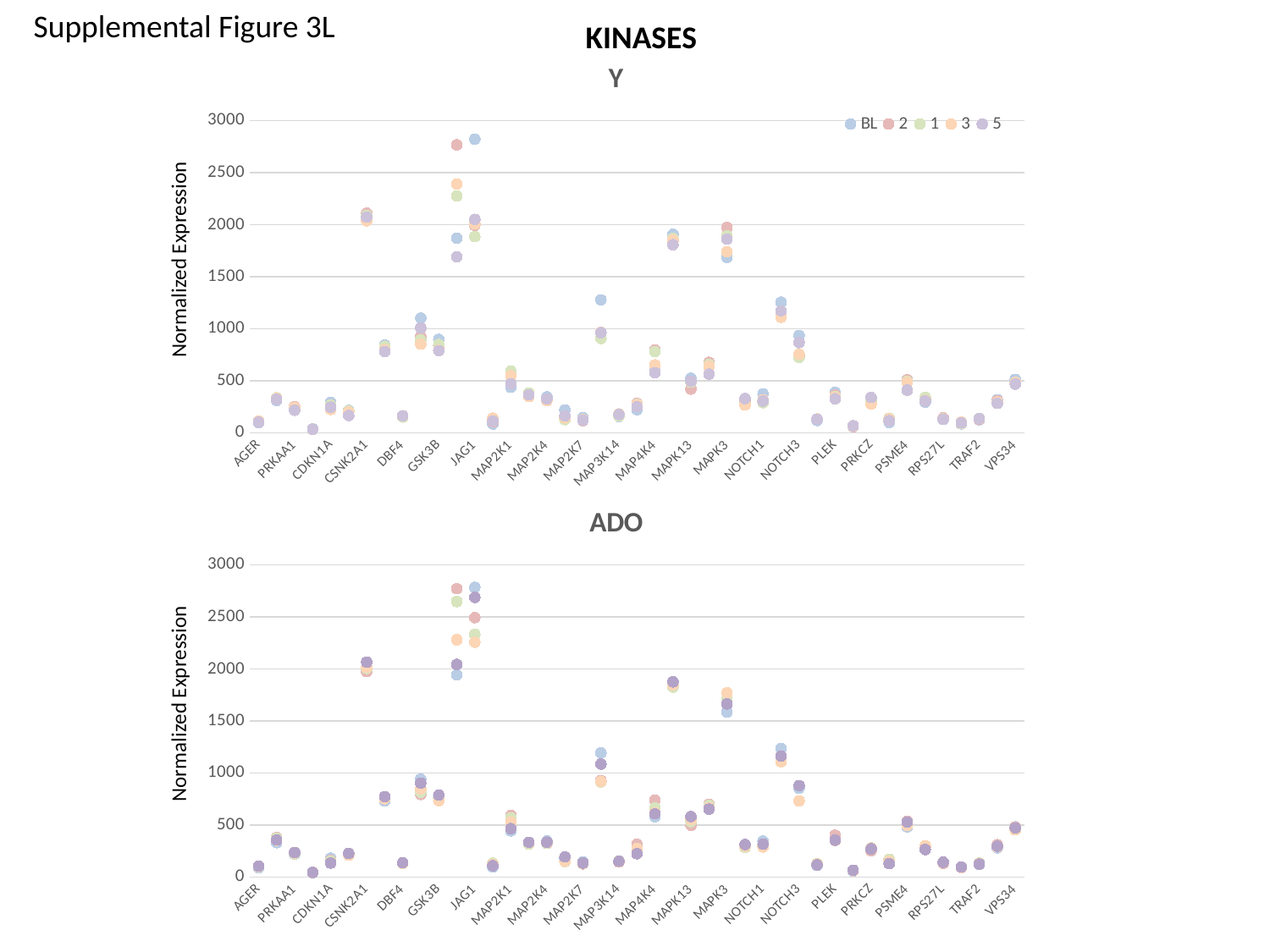

Supplemental Figure 3L
KINASES
### Chart: Y
| Category | BL | 2 | 1 | 3 | 5 |
|---|---|---|---|---|---|
| AGER | 96.69792039048758 | 106.90618303043948 | 102.13952577891523 | 114.12234622642414 | 101.8996975788392 |
| AKT1 | 310.13472912816985 | 329.67460575635135 | 334.7594486442448 | 328.7529261358194 | 321.0038860378875 |
| PRKAA1 | 246.046500400088 | 248.9206962665557 | 232.53482978656743 | 241.41339965800202 | 216.29554361139088 |
| PRKAA2 | 36.0878923632959 | 33.54187853931775 | 34.158450852486354 | 32.004713938096785 | 33.89561311148059 |
| CDKN1A | 293.3877765588877 | 266.46016134672385 | 265.14466064286665 | 224.21771214600662 | 246.33185700850532 |
| CHUK | 213.725350215326 | 204.016823127535 | 209.57453576864523 | 198.89930018316784 | 165.798272688384 |
| CSNK2A1 | 2070.1077442281367 | 2110.7010494705805 | 2088.721587558349 | 2036.4167254915556 | 2073.4981489905595 |
| CSNK2A2 | 842.5850169077885 | 826.6567097267862 | 830.4081368732346 | 795.8782139117604 | 780.4430530615388 |
| DBF4 | 156.41494838358352 | 157.20234969444078 | 151.41497986410135 | 163.94088515458566 | 163.02377116494358 |
| ERBB3 | 1101.0373709587561 | 924.0704876979685 | 892.3460220898755 | 851.522905098955 | 1006.9989370195362 |
| GSK3B | 896.6381287192498 | 837.6234636195549 | 847.1636974133942 | 792.2827816399636 | 788.9274087961351 |
| IGFBP4 | 1869.8971805854283 | 2767.281233425886 | 2275.4088187840916 | 2390.3313475025593 | 1691.5255271774367 |
| JAG1 | 2822.1394612820714 | 1993.8829725756175 | 1885.6991314993882 | 2008.2693448355021 | 2050.6139602622643 |
| JAK3 | 84.03221886230192 | 105.14025321017019 | 113.6055887948117 | 139.8245019237358 | 111.8978616785654 |
| MAP2K1 | 436.2257337380852 | 580.606230794582 | 591.6479909604483 | 547.4532413165444 | 472.0435401072091 |
| MAP2K3 | 349.0670120623161 | 355.2868147683874 | 382.12267779708213 | 346.8102928688338 | 363.7121457449538 |
| MAP2K4 | 344.77056731604966 | 309.702438062054 | 324.3863296630771 | 310.76799421004966 | 326.03540979189825 |
| MAP2K6 | 219.55938463876308 | 136.08530818859174 | 126.11701098855013 | 143.37620774824055 | 162.08360017138995 |
| MAP2K7 | 145.2112491367651 | 117.20965018337468 | 120.21288176598578 | 125.4299416008336 | 122.21451506911245 |
| MAP3K1 | 1277.1454669090556 | 907.1726828457698 | 906.5520217938465 | 965.2672422919919 | 961.5151046111182 |
| MAP3K14 | 156.30445157554792 | 165.58108496588582 | 161.56650256841021 | 180.2338697219433 | 175.48165589534898 |
| MAP3K5 | 222.4829613557179 | 281.83424808025035 | 272.38803975568675 | 268.69988033852337 | 249.9608198261867 |
| MAP4K4 | 614.5362331871474 | 796.7842790494528 | 778.9243243623407 | 651.9921713473553 | 576.4718780525174 |
| MAPK1 | 1907.502036192192 | 1846.7174266840957 | 1867.9369430511788 | 1851.8662293918105 | 1807.0497305765045 |
| MAPK13 | 523.6372997398665 | 419.5684638705163 | 485.1587875287622 | 495.69619501032525 | 497.03441013256037 |
| MAPK14 | 637.5121085343003 | 676.4523790275275 | 655.880521689622 | 638.3193611173388 | 562.9711488335148 |
| MAPK3 | 1686.3074591727066 | 1972.815613105138 | 1893.3634036565516 | 1739.3946531225931 | 1860.7886600327859 |
| MAPK8 | 326.47952139251754 | 293.6793369292676 | 281.5550420818667 | 267.44256011935136 | 327.99272843198764 |
| NOTCH1 | 373.45309344390415 | 301.9493123622426 | 289.4563339023882 | 316.52862122459425 | 305.05581469194857 |
| NOTCH2 | 1254.4048590545988 | 1117.593662022711 | 1134.0823362179638 | 1109.8837058147137 | 1172.225692605244 |
| NOTCH3 | 934.4149781285628 | 744.8851249295872 | 725.6823244392583 | 754.6728526349799 | 866.4351082458675 |
| NOTCH4 | 118.60662949916612 | 129.78232862533403 | 132.0776938544086 | 132.01376388333298 | 130.3890910201316 |
| PLEK | 387.8955133638618 | 361.1967677376306 | 348.5170428274136 | 339.83287704121204 | 326.3879976794513 |
| PRKCG | 58.34162840908227 | 57.821877639297426 | 63.889841623304356 | 65.71808637401533 | 67.34078418003202 |
| PRKCZ | 293.38491365140004 | 325.02764172381217 | 314.04095717266546 | 276.69596219014755 | 338.7462259412198 |
| PRKD1 | 98.77879410033842 | 138.85136418663325 | 139.28579685046802 | 132.84826260866305 | 114.947592367824 |
| PSME4 | 509.78548384146075 | 508.01248885444215 | 493.732839535031 | 483.72228024358316 | 409.60779017840395 |
| RIPK1 | 294.63436447929934 | 331.1569544096354 | 339.92050089449634 | 310.3333371782517 | 305.5937505104097 |
| RPS27L | 142.42199492752536 | 142.60749019280968 | 132.04739574984006 | 130.3515360354218 | 128.3037727764596 |
| TRAF1 | 85.72672316682875 | 87.24078123425414 | 90.64591725765332 | 101.50782413607772 | 95.17509327916497 |
| TRAF2 | 124.45848689949197 | 128.4877301872418 | 138.81963977333834 | 135.59378512094221 | 135.70607509138156 |
| VPS13A | 317.11893932100025 | 307.50593968059707 | 288.1320441998321 | 294.5144740839119 | 282.09027383828084 |
| VPS34 | 513.2868700121022 | 472.0501080133815 | 483.30622323163215 | 483.2216049433998 | 467.42998990212186 |Normalized Expression
### Chart: ADO
| Category | BL | 2 | 1 | 3 | 5 |
|---|---|---|---|---|---|
| AGER | 87.88728597057163 | 105.35969664797818 | 100.22375781518626 | 102.12493250183138 | 103.87200503091658 |
| AKT1 | 330.3431188585961 | 379.22872055885983 | 373.59232204607645 | 354.52046773201977 | 355.11428346571046 |
| PRKAA1 | 220.31443533363887 | 236.597035815582 | 223.29673644166635 | 230.5206939487008 | 235.35965098494955 |
| PRKAA2 | 44.23956693969897 | 45.26307297412316 | 41.10596166036075 | 42.525679595372246 | 43.16912236312528 |
| CDKN1A | 180.9678902003779 | 160.29059916149058 | 154.03499405535408 | 138.41387373243504 | 134.42666643333658 |
| CHUK | 211.84121684360895 | 225.10563963300626 | 216.46921371111907 | 209.36120702258904 | 226.60125815746372 |
| CSNK2A1 | 1990.4212302685685 | 1973.8522310183532 | 2005.5455735088824 | 2017.2074499241737 | 2064.5493348968134 |
| CSNK2A2 | 729.3968463628312 | 762.7636524649593 | 762.9365506935105 | 752.852464047804 | 773.1026390129263 |
| DBF4 | 133.55888460814973 | 133.44531429499204 | 135.26380400098412 | 133.0706796414919 | 137.4326273420216 |
| ERBB3 | 941.4459379675233 | 794.1531933214064 | 815.2167236182944 | 847.5928684167109 | 902.7827750199787 |
| GSK3B | 768.3554718402443 | 755.0562454724588 | 760.3174709156832 | 734.0565980378186 | 787.8280637294833 |
| IGFBP4 | 1943.3083513069084 | 2770.774213215594 | 2647.657540307964 | 2280.5787277394247 | 2043.3977159699534 |
| JAG1 | 2783.320451938585 | 2493.160922304998 | 2332.5656950519974 | 2256.339412549083 | 2688.3488598190665 |
| JAK3 | 96.42366356006507 | 132.9054918243644 | 132.78355356706464 | 121.12219816816408 | 108.2832174091921 |
| MAP2K1 | 443.5781240273627 | 591.9859809105544 | 569.8350907257881 | 528.7677922003629 | 464.69803138683085 |
| MAP2K3 | 327.21647805731084 | 325.858798177272 | 317.3700724437732 | 330.3532084480507 | 333.85438290872247 |
| MAP2K4 | 345.6143387801409 | 323.9619425187227 | 327.04555924975705 | 332.93523821666673 | 335.01064825434173 |
| MAP2K6 | 176.76043839505948 | 152.0136867577597 | 148.24614969822647 | 151.35634119008625 | 193.45585582952555 |
| MAP2K7 | 144.6793834152228 | 126.86555353277247 | 130.85758006957232 | 127.41424524865764 | 134.5914884279578 |
| MAP3K1 | 1193.3778556137845 | 927.7866672149119 | 913.6650248353027 | 917.0285011991821 | 1085.488428832162 |
| MAP3K14 | 148.57150593749282 | 144.83225115057377 | 153.31974833449698 | 147.61830764414776 | 151.74944795798402 |
| MAP3K5 | 224.6271468035194 | 314.1653166769061 | 273.6741613596021 | 271.510352933073 | 224.0646956712005 |
| MAP4K4 | 580.4902120948977 | 738.3958122839495 | 663.1437975025278 | 617.1044120723805 | 607.0661887469968 |
| MAPK1 | 1850.8498632655965 | 1840.7261093736213 | 1826.0008006484968 | 1847.9715655548093 | 1876.0224260054358 |
| MAPK13 | 564.43398268738 | 497.8069459574025 | 533.1114011982453 | 553.3509541616406 | 580.906375770008 |
| MAPK14 | 652.0888025593526 | 700.0353211309274 | 687.6085635445289 | 665.4056070706538 | 653.1351195535736 |
| MAPK3 | 1584.1661226903768 | 1686.8945425662196 | 1708.9038008879338 | 1771.0571786515263 | 1663.7012612897709 |
| MAPK8 | 291.3079980466171 | 284.98742920240176 | 285.8195722745603 | 296.8001777792915 | 312.5010015208769 |
| NOTCH1 | 344.05516608156114 | 300.2290633828465 | 303.2820007238904 | 289.6153683158507 | 316.95859120361473 |
| NOTCH2 | 1235.4490543132872 | 1122.497339521224 | 1121.8467270423985 | 1107.3223715407637 | 1162.7727781524204 |
| NOTCH3 | 853.1585523140607 | 732.4820531402498 | 732.5571697543086 | 731.1797354507922 | 878.8822718108264 |
| NOTCH4 | 110.97997400389823 | 121.64408049255839 | 129.21525781809686 | 123.25422606329954 | 116.62937878807467 |
| PLEK | 356.21960804779957 | 401.50089503668966 | 351.26889602136924 | 350.7784708369244 | 354.6914193337312 |
| PRKCG | 56.1682983002804 | 63.79372971879541 | 62.60130476147814 | 61.57249578582402 | 66.08454263343681 |
| PRKCZ | 252.8976906213027 | 253.0375951552682 | 280.30163195362707 | 277.84731595058827 | 270.4318785930918 |
| PRKD1 | 129.27537438454138 | 169.6737953528357 | 169.12265281764155 | 148.99171264602072 | 128.41988348285506 |
| PSME4 | 481.57453133961764 | 536.1581834282499 | 514.2428258490643 | 494.85496071937916 | 528.0317347492635 |
| RIPK1 | 291.0403618603846 | 292.69454501480874 | 295.50717549088 | 301.6734797776276 | 264.1357873191548 |
| RPS27L | 141.09799441781865 | 131.84118151352894 | 140.88501685734414 | 140.4154670277585 | 142.3760221118588 |
| TRAF1 | 95.79875744716577 | 90.92074657373384 | 94.42135039256404 | 93.79393795155949 | 95.54402476809587 |
| TRAF2 | 120.89968552474437 | 130.46267494286195 | 134.17225993727243 | 128.17911940487676 | 124.11531070168947 |
| VPS13A | 280.2846205737851 | 310.6834304678973 | 290.16869051775654 | 291.0056049320176 | 296.38708616605464 |
| VPS34 | 481.501016926435 | 480.6289974126603 | 456.2499873997956 | 452.84171033670054 | 472.1280029181941 |Normalized Expression

## Slide 20
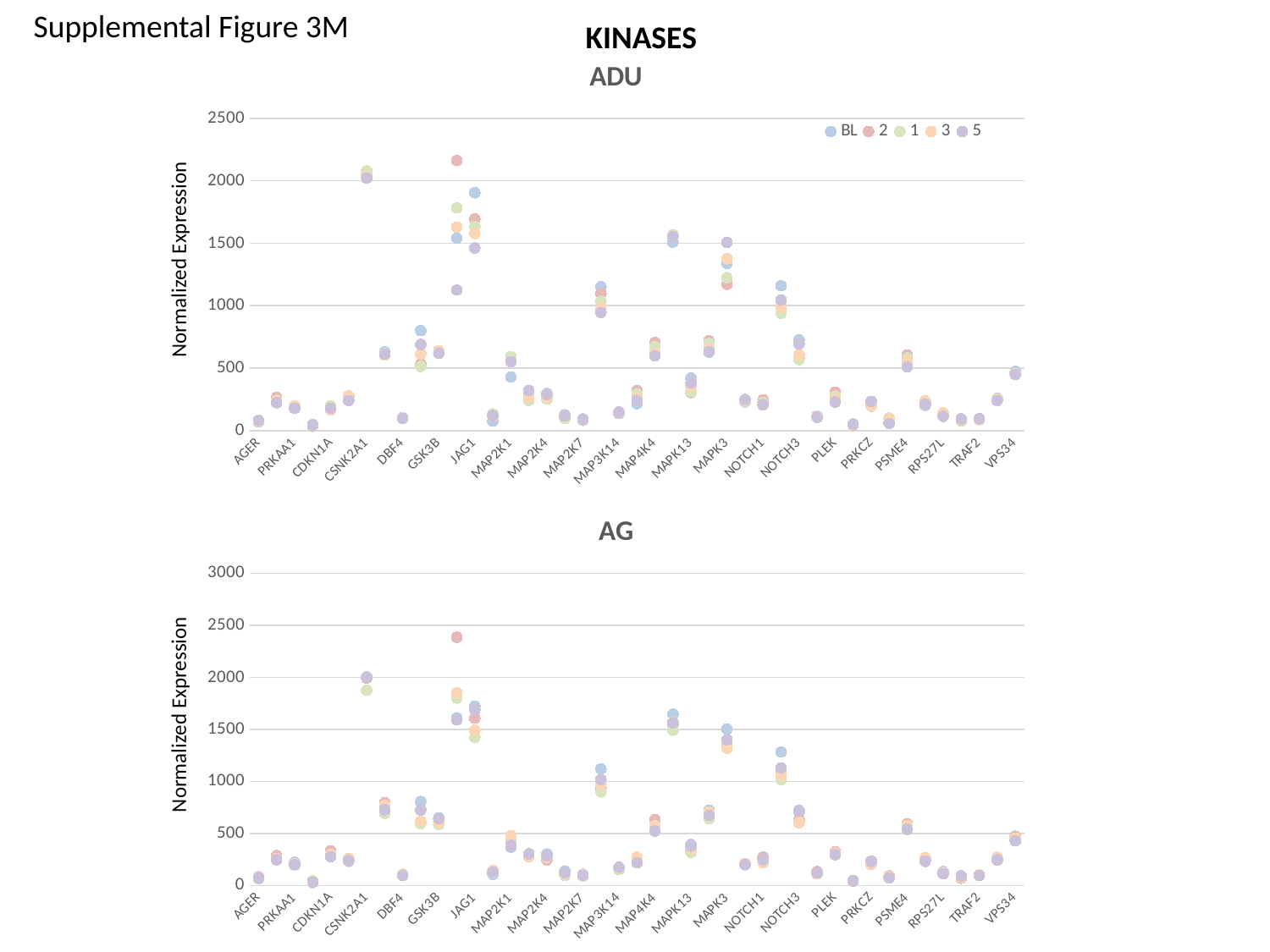

Supplemental Figure 3M
KINASES
### Chart: ADU
| Category | BL | 2 | 1 | 3 | 5 |
|---|---|---|---|---|---|
| AGER | 69.23196527919242 | 81.19806635767297 | 81.59249184686891 | 75.64542128021442 | 81.92166356446009 |
| AKT1 | 223.35046955822338 | 264.6555631247333 | 243.04429764031198 | 240.54286711823994 | 224.26660308989716 |
| PRKAA1 | 195.16504837801247 | 193.52112187814367 | 195.76047599628953 | 198.3830414874998 | 179.44204328113958 |
| PRKAA2 | 46.99577702042711 | 40.680627932401954 | 34.051631300022095 | 37.21498757995505 | 40.27535321938487 |
| CDKN1A | 196.04576298010917 | 195.84611609053562 | 199.74305127230758 | 164.9386059411646 | 180.0342488219785 |
| CHUK | 266.0487692638261 | 278.93829103786396 | 275.890867626516 | 279.09477452593165 | 240.57912266289037 |
| CSNK2A1 | 2030.7110363186212 | 2046.3031312063754 | 2078.716195530463 | 2040.0110342987116 | 2022.44237291001 |
| CSNK2A2 | 630.1404658535984 | 609.9364994158543 | 617.0783245148384 | 603.9186866131463 | 611.2772201596886 |
| DBF4 | 101.58886332360669 | 103.89699080815848 | 102.06973374992123 | 96.07577892055446 | 98.35100222233456 |
| ERBB3 | 800.7680396108467 | 533.1764427222819 | 513.2835589741509 | 613.8110101379597 | 689.3544189456463 |
| GSK3B | 640.8379424094538 | 640.1016256397278 | 622.070981470824 | 639.6992609356253 | 618.832810890837 |
| IGFBP4 | 1540.4962639302837 | 2162.5801378774977 | 1783.232229184866 | 1629.1501076039592 | 1125.7295247272345 |
| JAG1 | 1904.8903711927753 | 1695.3035770180013 | 1636.2855089485088 | 1579.0059244699924 | 1462.8894663136177 |
| JAK3 | 75.5315541698384 | 128.0689285673831 | 133.4572270393404 | 116.17013128202419 | 120.76611697275332 |
| MAP2K1 | 428.16131719148467 | 581.0125987774061 | 592.2760315774433 | 555.2856697027339 | 552.2488632775576 |
| MAP2K3 | 311.8840773549451 | 259.6169395894117 | 242.9054628034342 | 268.99358438619413 | 321.5912373260678 |
| MAP2K4 | 296.55428071316624 | 255.16334234696154 | 251.92549589310087 | 269.69982719768166 | 289.1347702210318 |
| MAP2K6 | 126.10049280730449 | 102.88540828130064 | 96.67879301377722 | 114.84022787428606 | 116.60792071566749 |
| MAP2K7 | 92.77324413435004 | 81.90884683858603 | 82.68573251030769 | 87.98861741679013 | 88.40525428805373 |
| MAP3K1 | 1151.5339357071941 | 1097.5467988061116 | 1034.6172110917807 | 981.9482998575004 | 945.6854092017895 |
| MAP3K14 | 137.00199933518172 | 142.9464328522493 | 149.88547999059546 | 144.8519512687878 | 148.9493338200218 |
| MAP3K5 | 216.26895860309904 | 321.00365464877297 | 293.68213764748424 | 257.6253103072661 | 241.60894183354543 |
| MAP4K4 | 608.5246290725984 | 704.6183199021403 | 674.5114322034283 | 620.571067559509 | 599.4489611132034 |
| MAPK1 | 1509.6040921693739 | 1561.1471979185098 | 1569.343161099449 | 1553.3575492932328 | 1553.7916519156115 |
| MAPK13 | 420.2048906916191 | 305.4053799218372 | 313.97908209311635 | 358.7883555946988 | 383.13528118905026 |
| MAPK14 | 629.5368048340724 | 718.6987261853984 | 696.1519835147315 | 656.1724145846287 | 632.5540915037583 |
| MAPK3 | 1339.448992569847 | 1171.8585503602164 | 1224.3772507857927 | 1376.2031008428607 | 1507.8245185588166 |
| MAPK8 | 232.31099242494008 | 229.5043435343128 | 236.78760328183242 | 245.728971462937 | 250.31674947048998 |
| NOTCH1 | 233.2439398498896 | 246.1059600241387 | 225.56248081487246 | 204.53509202354422 | 207.43546259115305 |
| NOTCH2 | 1159.8004673032747 | 1041.090168448215 | 941.2091659980046 | 988.0515900807311 | 1045.5562361717803 |
| NOTCH3 | 725.6135710280826 | 578.2312401372474 | 568.5136727939625 | 605.6411382442801 | 694.0943247596393 |
| NOTCH4 | 106.08510573665977 | 117.67383816208748 | 116.21192935723404 | 110.79354566130797 | 106.41009652579983 |
| PLEK | 299.6215799073758 | 308.39484919049437 | 275.5620001989627 | 251.81443588957154 | 229.6003670963683 |
| PRKCG | 47.4623944841662 | 46.717553858187244 | 48.96442633888343 | 48.53959006266521 | 52.02735714700245 |
| PRKCZ | 214.33738082070542 | 195.79297010708532 | 197.5649934996509 | 204.68745967602985 | 233.94393567853317 |
| PRKD1 | 70.67038741217846 | 92.33677214633695 | 100.6006170445541 | 92.45917549628601 | 57.38642971878091 |
| PSME4 | 530.5805655634344 | 605.274026143397 | 580.1211567980014 | 560.3374259484161 | 511.7708019183153 |
| RIPK1 | 202.289155361902 | 211.65398665547104 | 233.88461993968247 | 238.65596277837716 | 210.35117112861903 |
| RPS27L | 116.35512914630742 | 119.87102300675198 | 128.46406962096202 | 140.67126020204262 | 114.57145803330052 |
| TRAF1 | 77.83445216088833 | 80.29237638888056 | 78.93965939653383 | 85.33203771763112 | 93.20123721303409 |
| TRAF2 | 91.51659590893843 | 94.17872219667557 | 95.43370436265363 | 87.49604551696389 | 95.27653760155032 |
| VPS13A | 257.2496759024996 | 242.78201107874293 | 252.85616602579253 | 250.9579268495025 | 241.0535065276051 |
| VPS34 | 473.8347875550757 | 455.6279037841457 | 456.8157521592436 | 457.38571360482706 | 449.775786925846 |Normalized Expression
### Chart: AG
| Category | BL | 2 | 1 | 3 | 5 |
|---|---|---|---|---|---|
| AGER | 68.15842248277846 | 73.22415268009848 | 77.0656133565485 | 85.02585095197497 | 75.64087345142035 |
| AKT1 | 247.35999989829713 | 286.56958764181746 | 248.9256011402153 | 259.43622262791564 | 248.0363455483099 |
| PRKAA1 | 222.1311506293374 | 208.63473631105546 | 194.7753517959404 | 210.67702659792914 | 201.88986170342724 |
| PRKAA2 | 28.145587068071137 | 36.53650891797534 | 43.45358345266579 | 29.987053952502507 | 32.698288663686824 |
| CDKN1A | 296.63671846262184 | 332.8611615978639 | 294.94375663579103 | 298.55313347728435 | 277.4682997753355 |
| CHUK | 255.86238967891828 | 234.08536250972668 | 231.2077730185418 | 259.07784392884986 | 237.8325988500206 |
| CSNK2A1 | 2003.8576122468066 | 1992.1289069261193 | 1875.9439554629325 | 1999.2073886602027 | 2002.68319771558 |
| CSNK2A2 | 760.0786995373414 | 794.9265351179171 | 694.0324803824581 | 771.681674271391 | 727.510259908807 |
| DBF4 | 106.84737236288925 | 104.89835370668578 | 106.25587180400179 | 107.19362757825974 | 95.72051927058011 |
| ERBB3 | 806.2716581766443 | 609.5592243039131 | 595.1958477460529 | 616.724542315998 | 724.6636762902874 |
| GSK3B | 650.5768818638122 | 637.7303599040577 | 586.5710017920113 | 606.8470395635987 | 641.4600766850806 |
| IGFBP4 | 1611.8401994551398 | 2385.3321864619593 | 1801.8090731672403 | 1851.778241915144 | 1591.0294225082444 |
| JAG1 | 1723.0909761341597 | 1608.1199272362658 | 1424.2959576696521 | 1492.26995677428 | 1690.5733013639656 |
| JAK3 | 106.77058879558886 | 121.08265452964355 | 121.86213528553913 | 143.39524794773035 | 126.82000499993852 |
| MAP2K1 | 368.8761597766618 | 461.08787374181765 | 427.5456138263472 | 479.2317055442255 | 386.0792828359454 |
| MAP2K3 | 291.14763314842565 | 277.9732486383517 | 301.7333044922946 | 276.1702093161466 | 303.4903502477226 |
| MAP2K4 | 301.8657062937306 | 245.7698946915324 | 270.2654867620793 | 272.6877995325893 | 284.7823615702316 |
| MAP2K6 | 137.8169575989524 | 97.58276152301015 | 101.50110967118505 | 116.85194641539248 | 123.16944371962339 |
| MAP2K7 | 106.9624082432564 | 97.08625827180578 | 91.15722398174229 | 100.24531379519415 | 96.38100655548246 |
| MAP3K1 | 1120.475140650277 | 938.7330666571052 | 899.2699100226235 | 969.8069183216264 | 1016.7180320781385 |
| MAP3K14 | 164.22929487917847 | 153.94667084281622 | 157.44928464636376 | 171.0899254903993 | 175.64642306872855 |
| MAP3K5 | 223.174757497482 | 269.49187925227795 | 237.1018050528964 | 271.0449691097807 | 218.23237298527954 |
| MAP4K4 | 557.536891597512 | 632.5147900450258 | 568.1955723612427 | 572.310854687023 | 523.2182252290354 |
| MAPK1 | 1645.9534686884224 | 1547.9942275962271 | 1492.483409371474 | 1572.0152040099374 | 1564.0565241159238 |
| MAPK13 | 393.9891383088915 | 320.47344022085787 | 318.5319048002805 | 351.4755773409154 | 379.11765734887865 |
| MAPK14 | 723.0765810555426 | 664.4011033621638 | 642.2841668274339 | 704.5745346851423 | 673.7999546101946 |
| MAPK3 | 1503.5538902857681 | 1365.2306190530558 | 1388.2464456319633 | 1320.7927414244596 | 1400.9391837924368 |
| MAPK8 | 199.2824417195174 | 206.4611669650248 | 199.04877175037473 | 211.7254525322472 | 200.96066682666543 |
| NOTCH1 | 272.12574344254665 | 262.8651779418963 | 241.9210613346824 | 218.34588397207733 | 248.77429627643843 |
| NOTCH2 | 1281.921739850127 | 1101.5644860771345 | 1015.2162705324162 | 1058.3772510008082 | 1129.758676852831 |
| NOTCH3 | 706.9628798581967 | 635.3469117113823 | 602.7791921633977 | 607.9367031958008 | 720.3801319093421 |
| NOTCH4 | 116.8871577973841 | 133.89703171654534 | 118.25966331567413 | 112.22453996911723 | 119.0639708902084 |
| PLEK | 314.70939459427115 | 324.0150806447451 | 295.8673298607131 | 311.0585796491574 | 297.30003374225544 |
| PRKCG | 42.20386695597174 | 43.3326087818746 | 49.451579822287286 | 49.317787093237946 | 46.35755447658054 |
| PRKCZ | 224.50814816850814 | 234.30262951257316 | 223.8365519948176 | 202.05134923330226 | 231.54792852317325 |
| PRKD1 | 77.82851533709767 | 91.34516895394492 | 82.46357927729284 | 84.25164151144362 | 72.85593002398146 |
| PSME4 | 542.5808611399179 | 594.2346374956672 | 542.5201152730357 | 575.3572624785393 | 539.8112419761261 |
| RIPK1 | 231.5007064325239 | 251.6562038713933 | 247.30145323254254 | 266.12636876910847 | 234.3557702214144 |
| RPS27L | 132.57660538284725 | 115.0713866878107 | 118.57028237720577 | 122.10837774292662 | 112.36829294092561 |
| TRAF1 | 78.7753936426057 | 70.05338290984733 | 81.49530638954764 | 79.91119761012811 | 91.67010364832457 |
| TRAF2 | 96.60155871224869 | 100.46233650519119 | 101.83434260437376 | 101.24354834866114 | 95.52207985732447 |
| VPS13A | 268.9897547190498 | 254.90416899357731 | 241.26924355696565 | 270.50142984913003 | 246.6089280345164 |
| VPS34 | 472.82665065066794 | 466.646629868712 | 436.6960703627518 | 454.35054838457665 | 428.11406092878207 |Normalized Expression

## Slide 21
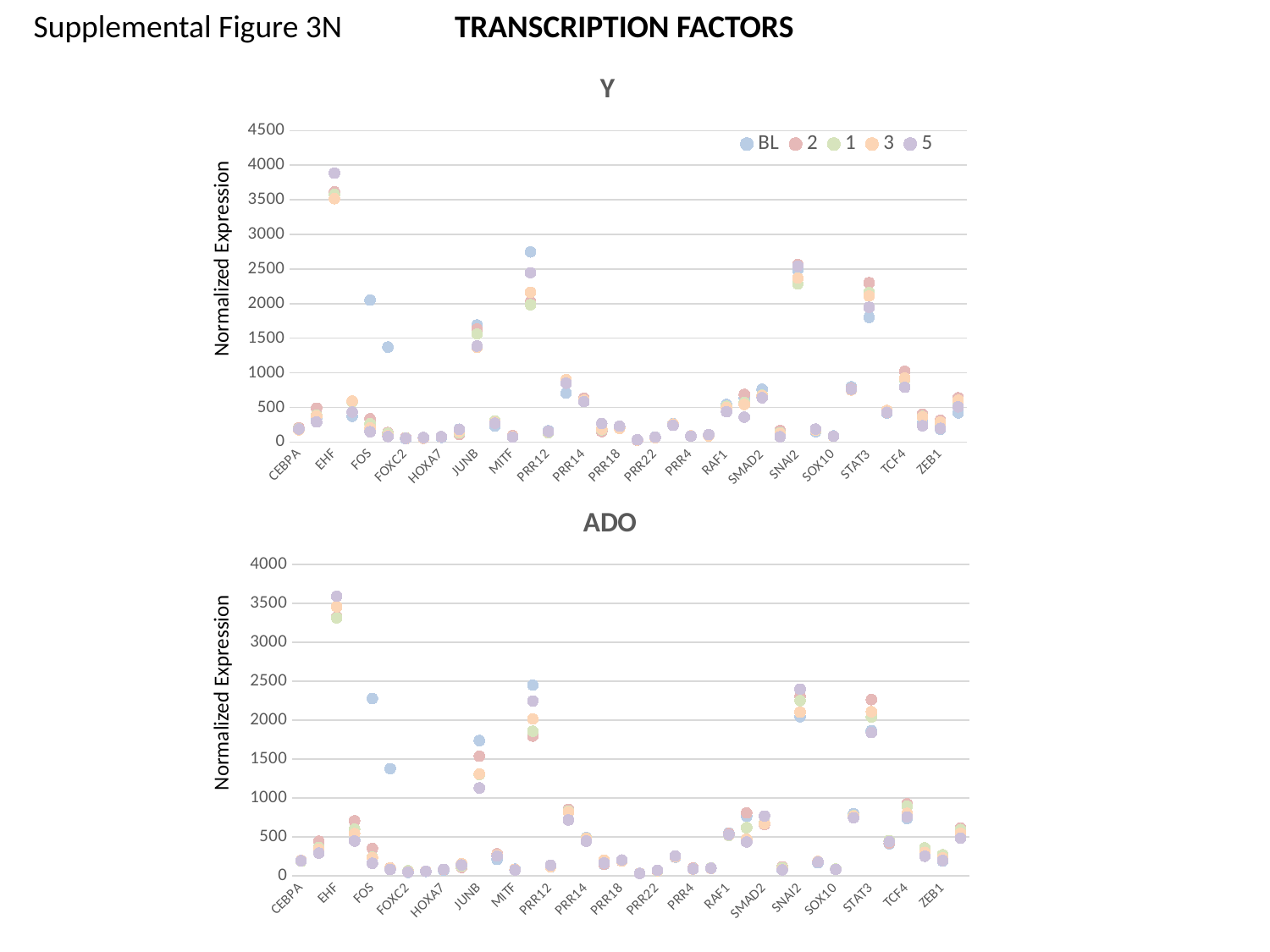

Supplemental Figure 3N
TRANSCRIPTION FACTORS
### Chart: Y
| Category | BL | 2 | 1 | 3 | 5 |
|---|---|---|---|---|---|
| CEBPA | 196.345343286124 | 206.80156298143754 | 192.15964295518137 | 175.63806594280769 | 189.14274699384202 |
| CEBPB | 346.3636212328085 | 489.5923475002628 | 387.61742285879876 | 374.6667304129466 | 289.412841045165 |
| EHF | 3602.841412399216 | 3615.0758319826923 | 3577.6273120169103 | 3516.3894029118023 | 3886.30066205578 |
| ETS1 | 371.0326244308042 | 590.2436240353924 | 579.4889753354922 | 591.5162090210016 | 433.63980155925486 |
| FOS | 2049.7149092917734 | 334.93351058895723 | 263.88448813169134 | 202.5369096568679 | 148.63751993729704 |
| FOSB | 1371.8188810350466 | 136.61752662578775 | 125.05640594506434 | 88.96502344627895 | 80.12416412840435 |
| FOXC2 | 46.16239717952663 | 62.718497034375254 | 56.97128391050842 | 52.66199474300177 | 53.595393466164694 |
| GSC | 54.35565767131291 | 54.0763029053316 | 55.63384442498242 | 55.34438837817772 | 65.22834090230091 |
| HOXA7 | 67.05760587629761 | 77.65018976721746 | 77.03074224736464 | 74.71635274575796 | 76.9709822769767 |
| ID4 | 184.9372534155047 | 109.47702212458634 | 131.8762479221939 | 154.00771236700058 | 182.61223116950066 |
| JUNB | 1690.9681469905222 | 1632.9749478361523 | 1560.427123557462 | 1368.9426960814335 | 1387.003441335653 |
| KRAS | 232.87761431896718 | 289.06236827839007 | 304.75198466060675 | 282.94189544157655 | 271.7664859139783 |
| MITF | 81.58218384060291 | 89.64102068562384 | 76.25306529367248 | 77.0754263354041 | 72.34345325242253 |
| NFE2L2 | 2746.9885474139155 | 2025.097573320648 | 1983.5272284093007 | 2164.8156372455555 | 2446.874017297881 |
| PRR12 | 162.4182303756219 | 133.55913603802492 | 134.69727329871426 | 149.26642782220944 | 152.39242442457217 |
| PRR13 | 708.2769425307416 | 852.4720992558908 | 853.1254611980431 | 900.9838119428617 | 849.9205671127128 |
| PRR14 | 627.0110341537907 | 632.1735396338861 | 585.7730779408854 | 603.7222419645517 | 583.9249199210741 |
| PRR15 | 164.54192340689517 | 151.45816045245758 | 177.53515156099203 | 188.74121486338217 | 268.3994748296563 |
| PRR18 | 201.2028876515721 | 197.18363753775637 | 208.00220861234374 | 196.8574403104147 | 228.38938258030151 |
| PRR19 | 31.17590751896662 | 28.430133793843954 | 32.1387907064813 | 31.040449961274646 | 32.55678292480235 |
| PRR22 | 72.32077007640586 | 64.36442368781738 | 65.30802588158502 | 61.80144257717773 | 69.94564144580104 |
| PRR3 | 263.8040931329676 | 248.6946554520267 | 242.9704398722249 | 259.41828572055863 | 240.7467952475485 |
| PRR4 | 90.9717798205406 | 87.21779893186883 | 86.03126240369168 | 90.71947453604513 | 85.6295282846262 |
| PRR7 | 90.24486213831814 | 92.11958816162002 | 93.44963361769724 | 89.17940718629916 | 107.22840318917824 |
| RAF1 | 543.5057286212159 | 511.61948067750615 | 516.2060190530575 | 496.88647129947236 | 438.9409140926007 |
| RGS2 | 632.0288969518272 | 687.4984152483097 | 569.9417725178503 | 542.4358828948957 | 360.30576642121696 |
| SMAD2 | 761.4393386579296 | 663.7260600694858 | 675.8956386996686 | 669.1573005461128 | 639.913577697701 |
| SNAI1 | 108.81843288979866 | 165.05047280266567 | 134.38790131146618 | 121.82680506050161 | 76.88185415297431 |
| SNAI2 | 2489.822305053853 | 2565.043655452426 | 2285.1251668987384 | 2369.037444248179 | 2543.465789631517 |
| SNAI3 | 151.62531644694502 | 167.8605288119105 | 170.58006472575826 | 174.16638595116157 | 187.10335962510788 |
| SOX10 | 88.60933270173467 | 84.3865146815157 | 86.38773921014038 | 81.87492267776007 | 85.81243693738935 |
| SP1 | 797.0090182992075 | 772.6024498944581 | 759.827225050008 | 752.5421940788111 | 763.6671928454333 |
| STAT3 | 1802.1541214536999 | 2301.5669703250433 | 2157.3528604679764 | 2109.6908894706526 | 1946.9106623307084 |
| TCF3 | 418.23563769825876 | 425.56772890628514 | 445.6749963668695 | 454.6155260779076 | 429.79484922644684 |
| TCF4 | 795.7480037396659 | 1021.7821309349721 | 901.2249754708399 | 922.3926745135245 | 790.0497358006668 |
| TWIST1 | 277.8435409572246 | 402.3733507463374 | 369.5903982305706 | 362.676821669929 | 235.6820623027038 |
| ZEB1 | 185.0651093598233 | 314.41348445169035 | 279.2237688985515 | 286.63077546826867 | 197.46392994816097 |
| ZEB2 | 421.18042662852076 | 638.4446325046624 | 542.3638279447074 | 603.1507687520482 | 508.2688732388698 |Normalized Expression
### Chart: ADO
| Category | BL | 2 | 1 | 3 | 5 |
|---|---|---|---|---|---|
| CEBPA | 193.09123715427185 | 188.9310942247572 | 186.8338223345797 | 197.30857671458858 | 193.01634069449855 |
| CEBPB | 353.73287850964635 | 442.6560612582034 | 361.47424472819574 | 339.62312989982047 | 290.6004606025319 |
| EHF | 3324.5656468536636 | 3323.74274807595 | 3313.6562037262283 | 3455.74353039404 | 3589.8140955368517 |
| ETS1 | 461.15892080053266 | 705.5427085860997 | 597.0838624366328 | 540.5851114416997 | 446.1468796838596 |
| FOS | 2276.293538880401 | 350.83633946045194 | 239.70856140382116 | 230.24013342595856 | 160.54285581423503 |
| FOSB | 1375.7183529161348 | 100.09978869978687 | 96.0984507126171 | 99.29010253928055 | 80.62706665494747 |
| FOXC2 | 44.37445086471374 | 61.80433296343535 | 62.906593867506814 | 50.44485848787152 | 46.739175535766556 |
| GSC | 56.28480624554649 | 57.938353968788725 | 57.73229172356749 | 58.513050892167854 | 57.66234052778347 |
| HOXA7 | 71.85945942890268 | 79.72697282336199 | 83.00407774265395 | 83.04646925751949 | 82.45149707635713 |
| ID4 | 151.25153556402284 | 107.21878171597459 | 121.77122730021804 | 154.9543371015293 | 137.86542310991013 |
| JUNB | 1736.160075108597 | 1535.4072934625956 | 1301.0220716189067 | 1307.0595282208599 | 1127.7068654156396 |
| KRAS | 209.45907771517992 | 281.3185091367277 | 266.17156824362326 | 267.1889010184965 | 252.29888992929 |
| MITF | 84.73714689992438 | 79.32873677219794 | 79.89153782473598 | 83.22179203785761 | 73.9280308319534 |
| NFE2L2 | 2449.301718126193 | 1794.3652677702537 | 1856.1612282972173 | 2014.1367112282314 | 2245.2712246148158 |
| PRR12 | 114.36844710381787 | 119.41126771678172 | 123.27226925427824 | 115.73876219841026 | 135.58509705300418 |
| PRR13 | 720.164422443157 | 853.12131294653 | 831.0950546321296 | 818.45664455444 | 716.5756439192207 |
| PRR14 | 488.3938002221904 | 459.78373427264665 | 481.97315497823547 | 477.87010258700076 | 444.9708691143013 |
| PRR15 | 144.0012828630465 | 150.2526486773461 | 169.79982531733916 | 199.22284418069574 | 163.86324524295176 |
| PRR18 | 200.22526733103393 | 187.73062012929663 | 191.69372080214407 | 190.73471962148272 | 202.9941336482717 |
| PRR19 | 29.7046499631076 | 30.122669181269618 | 29.86828029923474 | 30.948906450985103 | 29.48652338792874 |
| PRR22 | 68.68369471886965 | 62.33500740470197 | 62.81388697177119 | 66.56801554946361 | 68.65335722223683 |
| PRR3 | 238.79341507807555 | 242.72085093752887 | 246.04673654167405 | 246.2829632595109 | 255.25032010350898 |
| PRR4 | 100.00818439979645 | 98.92227234047903 | 90.13999316363038 | 85.35710829084724 | 87.65549509515712 |
| PRR7 | 100.66578917126066 | 94.13737888905403 | 100.1090089613819 | 94.46451982369364 | 97.24890764812011 |
| RAF1 | 548.0270525898788 | 540.6226531795228 | 518.3372829628228 | 525.7332111414066 | 528.4520199919272 |
| RGS2 | 764.1456141638105 | 807.2086665918374 | 617.9904716929582 | 469.6200854004789 | 433.3930478453997 |
| SMAD2 | 671.1100483957514 | 657.5963256207259 | 678.963016086313 | 670.9736303186095 | 767.4195978912885 |
| SNAI1 | 74.65561480435427 | 115.35167377386028 | 104.26231527899422 | 80.25488847947075 | 75.04221633105823 |
| SNAI2 | 2043.1097699263757 | 2300.987024516254 | 2250.2046378064647 | 2101.3020599270717 | 2397.995022704732 |
| SNAI3 | 165.6671221301293 | 180.63504472636268 | 180.2141303562995 | 182.83162693745751 | 176.39649746209386 |
| SOX10 | 83.68266557524603 | 81.91064560724823 | 85.66868896557733 | 81.16762227980058 | 80.99048390161994 |
| SP1 | 797.0201427271796 | 763.7754720120272 | 771.8030464157832 | 765.2041869691999 | 744.9093528243133 |
| STAT3 | 1862.9782673501359 | 2262.3737877186013 | 2038.8885832743686 | 2106.952421941922 | 1838.940114231709 |
| TCF3 | 411.8414857622463 | 419.70935480073183 | 452.1785967017079 | 440.17399195725574 | 439.92164033785684 |
| TCF4 | 735.1220782984574 | 922.7819013781352 | 895.0852929573867 | 801.1078910436423 | 756.3845846430545 |
| TWIST1 | 325.11549103434754 | 349.4279749776358 | 356.6434499769611 | 300.4410891904053 | 251.04748121204022 |
| ZEB1 | 188.61836920229032 | 265.3001609311262 | 269.60974640043776 | 227.11660336089287 | 197.05345371502256 |
| ZEB2 | 481.41346199040424 | 613.816655078013 | 593.8168252822954 | 545.6589286934247 | 483.3465404733873 |Normalized Expression

## Slide 22
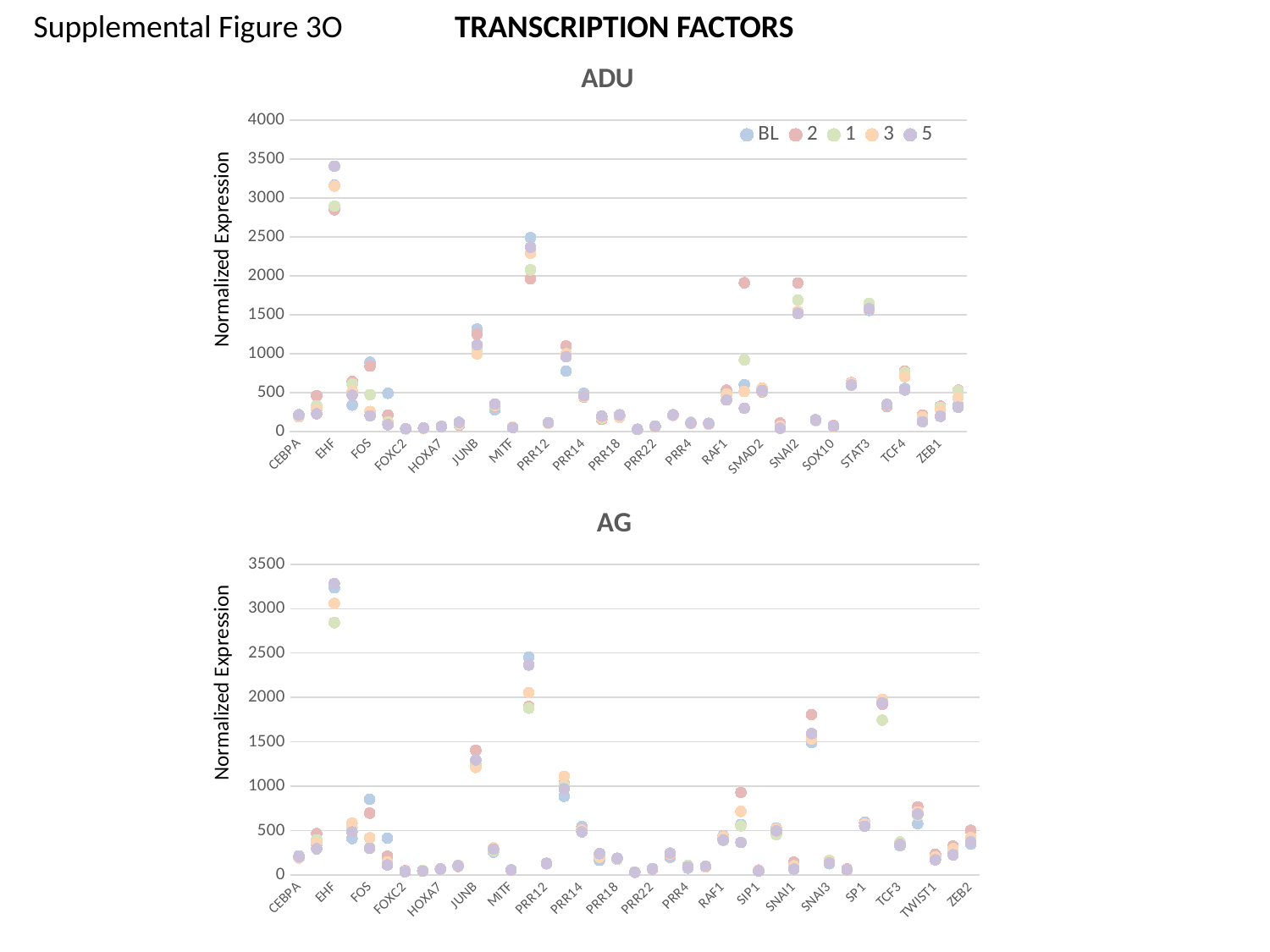

Supplemental Figure 3O
TRANSCRIPTION FACTORS
### Chart: ADU
| Category | BL | 2 | 1 | 3 | 5 |
|---|---|---|---|---|---|
| CEBPA | 216.45454409791265 | 203.47618546587512 | 188.3787384762666 | 196.21807902832873 | 211.7902364904252 |
| CEBPB | 313.1169730219282 | 460.3777173631295 | 329.61695260365036 | 284.7615535289654 | 226.79448116909177 |
| EHF | 3164.054731396269 | 2847.4390465714137 | 2895.619261269608 | 3152.0197510088024 | 3408.306587692061 |
| ETS1 | 338.49367558111817 | 641.8692426070083 | 611.3254187075411 | 513.0905437600809 | 466.1577211782377 |
| FOS | 890.0983134751558 | 840.5510295357412 | 472.8788983082291 | 256.73831466992283 | 202.16296484211028 |
| FOSB | 490.4034235853117 | 210.52044368090438 | 125.86303311661365 | 97.44351872437935 | 87.34342808652056 |
| FOXC2 | 34.34530702974228 | 36.488910644311034 | 36.44927695888191 | 33.61967950451008 | 35.21534195456955 |
| GSC | 43.567050027455224 | 45.29488946335032 | 42.14774262179205 | 40.11511493491839 | 45.3694913524696 |
| HOXA7 | 61.49230848442494 | 65.58659678408769 | 65.43009197758154 | 65.68397938339643 | 65.26815732202427 |
| ID4 | 116.25190896877254 | 81.86878256969163 | 94.26385167302914 | 107.01997983511245 | 117.96165682544132 |
| JUNB | 1315.1497790470257 | 1250.8881434134832 | 1049.9480299342736 | 997.8191228837945 | 1112.2722555763353 |
| KRAS | 278.6654746934538 | 317.0379073503903 | 335.9883213344086 | 345.633033016963 | 350.99568977530237 |
| MITF | 56.90239800499004 | 56.68873082911937 | 54.08189158915669 | 56.934793343873544 | 50.172226213511244 |
| NFE2L2 | 2490.3817624554626 | 1960.1731812262208 | 2076.9172610419655 | 2291.6517923115357 | 2366.8797808102145 |
| PRR12 | 115.2360074629597 | 107.01776017801194 | 110.65048504184824 | 107.1708226150585 | 110.77848355041444 |
| PRR13 | 775.3589963213003 | 1098.6112482314861 | 997.673464392034 | 991.8922680595527 | 959.5824228793618 |
| PRR14 | 488.5620603967883 | 440.7765896865717 | 461.66180845150745 | 447.9482343137725 | 459.7800430206592 |
| PRR15 | 155.23166215331594 | 155.84723043883673 | 173.69265049802368 | 176.1655210243208 | 197.5213921006759 |
| PRR18 | 182.82869611108868 | 191.6307707779864 | 181.03407667423025 | 180.18117550796543 | 214.2245820889502 |
| PRR19 | 29.517524366435524 | 26.37407980553456 | 26.62215603745353 | 27.27063256377237 | 28.707458082529072 |
| PRR22 | 70.13715376881748 | 61.82779768884765 | 63.46956475763019 | 60.481953802276685 | 67.2292935457196 |
| PRR3 | 216.09258974432134 | 212.6004682706637 | 216.86567354406554 | 205.1101835754359 | 214.53194630488446 |
| PRR4 | 118.59615814123481 | 113.21081390234846 | 115.90743708765247 | 103.9055479766201 | 109.08024035080425 |
| PRR7 | 93.85502126662365 | 103.09260393614488 | 96.75780548331252 | 93.74446500546661 | 104.89742282030254 |
| RAF1 | 490.6494376073227 | 531.8280439583913 | 479.53174178938923 | 467.4646843507205 | 405.06463542474404 |
| RGS2 | 600.9379805913467 | 1909.930433221651 | 920.7288078776237 | 514.0639941339691 | 299.08090071781476 |
| SMAD2 | 557.3336108108583 | 506.80513270689204 | 522.444517894198 | 555.5454483147093 | 525.8393300931224 |
| SNAI1 | 59.1683613066568 | 108.29791896988478 | 70.00922190699208 | 64.72046638360081 | 41.45074480558111 |
| SNAI2 | 1515.1723406437688 | 1907.5176647089881 | 1689.4709120517546 | 1542.0963834029153 | 1518.0407599809346 |
| SNAI3 | 140.92155687949258 | 150.9367313070736 | 145.74595971562186 | 139.08772885469384 | 146.6676311866567 |
| SOX10 | 60.854638393356026 | 75.68836115830953 | 66.80857618039853 | 64.10801021748311 | 68.39500105795273 |
| SP1 | 627.4708978472624 | 627.0035125949954 | 590.8759630696665 | 618.915160220703 | 598.0583809444092 |
| STAT3 | 1555.0667595609007 | 1618.7930595182943 | 1644.4906175903404 | 1572.679008435299 | 1575.8902672922031 |
| TCF3 | 318.98546716141243 | 324.40963232866 | 342.85712924855414 | 340.50521126893085 | 346.7368475603196 |
| TCF4 | 552.0522734839818 | 776.3646491672985 | 758.7020224116875 | 704.8009317977743 | 533.7425325725068 |
| TWIST1 | 165.6835822729609 | 212.4079549950027 | 179.31961869374612 | 189.41524634636968 | 125.85948961793659 |
| ZEB1 | 194.71430128133898 | 324.82137107760786 | 311.06589241449916 | 277.07808892817076 | 195.1043730012481 |
| ZEB2 | 338.6941131450439 | 530.5073605215399 | 521.3090284577404 | 438.5399213940575 | 312.29679071710615 |Normalized Expression
### Chart: AG
| Category | BL | 2 | 1 | 3 | 5 |
|---|---|---|---|---|---|
| CEBPA | 215.15798186792287 | 191.61292565016402 | 192.22351275339412 | 188.72154600895408 | 205.69253225455964 |
| CEBPB | 346.8822724567844 | 466.32281428242953 | 396.0282371494119 | 363.06292331144766 | 292.78140884852945 |
| EHF | 3234.209973298745 | 2844.4624656452293 | 2842.254629979871 | 3060.9350864035823 | 3282.714226439 |
| ETS1 | 409.2125843654224 | 583.3773199577104 | 531.3251103685689 | 584.353185131507 | 482.93097202732065 |
| FOS | 850.5193737029103 | 695.5656958235488 | 305.82416218818173 | 418.0252312279432 | 300.20211659866834 |
| FOSB | 414.00757277056937 | 211.07402400314422 | 121.71626368424418 | 144.69384752693045 | 110.76360415136261 |
| FOXC2 | 34.24193579885925 | 47.553214985446246 | 39.50732660104162 | 38.51422667785433 | 35.663039774581556 |
| GSC | 40.156346178953676 | 39.75172318488015 | 50.31147159136564 | 39.66365946926484 | 43.92056098515795 |
| HOXA7 | 69.1569477961042 | 64.7359085905248 | 64.4992692748043 | 67.3844472614798 | 65.85389758619381 |
| ID4 | 101.52415558033857 | 93.78036668042488 | 96.51778640645287 | 105.688550250471 | 101.89053895074309 |
| JUNB | 1293.0653546837975 | 1405.4545580832294 | 1246.7287309490976 | 1211.2791628424793 | 1295.9301387302633 |
| KRAS | 255.7805193274512 | 298.38717084021107 | 271.6938529890964 | 303.4551207715383 | 290.3554759970531 |
| MITF | 57.90667346514137 | 56.6853264509801 | 57.971960552445864 | 53.86823295906953 | 55.047660257697295 |
| NFE2L2 | 2453.120578006295 | 1898.5333691821154 | 1879.0908432148326 | 2053.0298397081438 | 2365.4839808249963 |
| PRR12 | 131.47093563071778 | 128.36192214958893 | 123.90655891550375 | 123.76738718538705 | 127.80297633856986 |
| PRR13 | 885.9026907579242 | 1029.874998133641 | 1010.2788530905185 | 1111.070349786348 | 968.9346492032221 |
| PRR14 | 544.8077116241486 | 511.0905454271472 | 502.4813565255063 | 503.2576363825513 | 484.37260565028396 |
| PRR15 | 163.65528465567294 | 195.64415106919552 | 198.618278733876 | 204.93946303390678 | 239.25448094494382 |
| PRR18 | 176.83651808450685 | 180.7827380235875 | 180.21171204012097 | 187.29239151603028 | 187.7011978121584 |
| PRR19 | 30.14547441087906 | 29.9802010802205 | 32.82899142412663 | 27.995992961652647 | 29.179578044124355 |
| PRR22 | 65.41144599334558 | 65.91635846730972 | 68.95287046959618 | 70.97646468709554 | 71.60810785743303 |
| PRR3 | 197.04794041001801 | 227.88623954875322 | 235.58648932642234 | 235.6828602437044 | 244.65514100727103 |
| PRR4 | 76.56477690793328 | 93.1868265036953 | 106.78107271698235 | 87.48728330881099 | 90.37674157041512 |
| PRR7 | 87.92765027746954 | 90.28738115728126 | 91.89323197525907 | 89.8576290865801 | 97.56771619328953 |
| RAF1 | 441.71267812660176 | 421.87649047976925 | 388.667385114376 | 429.54628554420776 | 391.69114446444945 |
| RGS2 | 566.9812742549534 | 928.9673538144668 | 552.6337960093738 | 715.2702841799152 | 365.6989820084307 |
| SIP1 | 54.2306640896223 | 41.82456026280118 | 47.586908817373775 | 53.255587927438796 | 45.23182182031424 |
| SMAD2 | 528.8855693074034 | 483.1132503790363 | 453.37878017834333 | 517.4204447872428 | 496.4829413314867 |
| SNAI1 | 67.53512730230862 | 143.50714632426607 | 101.61163244912221 | 100.62435553562597 | 65.5869203419367 |
| SNAI2 | 1489.9358454532749 | 1805.1960661672706 | 1581.3589200112735 | 1533.3023366304021 | 1592.501811723241 |
| SNAI3 | 124.9231764390465 | 143.00465564350037 | 163.35469638748089 | 142.96791700435142 | 134.375367059214 |
| SOX10 | 58.725890631938 | 66.35715967277349 | 62.58854225814319 | 54.18621428281262 | 56.79165881960679 |
| SP1 | 594.3628976315312 | 577.7276502095211 | 552.0046968840907 | 574.7574554119713 | 548.4697930918462 |
| STAT3 | 1970.087717925083 | 1923.3449884627726 | 1743.6125602616353 | 1978.4173462834437 | 1938.9364322796673 |
| TCF3 | 328.74448807123736 | 341.7120127742001 | 368.9742358932185 | 345.21369500466886 | 343.2063044402051 |
| TCF4 | 577.6156310216109 | 765.738522733261 | 672.3694547277227 | 709.6897315704862 | 685.3154786186481 |
| TWIST1 | 200.22326454495027 | 232.64156608670731 | 181.73282119874892 | 202.12783819085587 | 168.92652006697136 |
| ZEB1 | 229.79825819489184 | 323.915108127225 | 257.6549856218626 | 293.2232083666722 | 225.56183536500833 |
| ZEB2 | 345.5662487130335 | 502.1884328600124 | 420.3124776008458 | 417.3026009013835 | 371.42180318646587 |Normalized Expression

## Slide 23
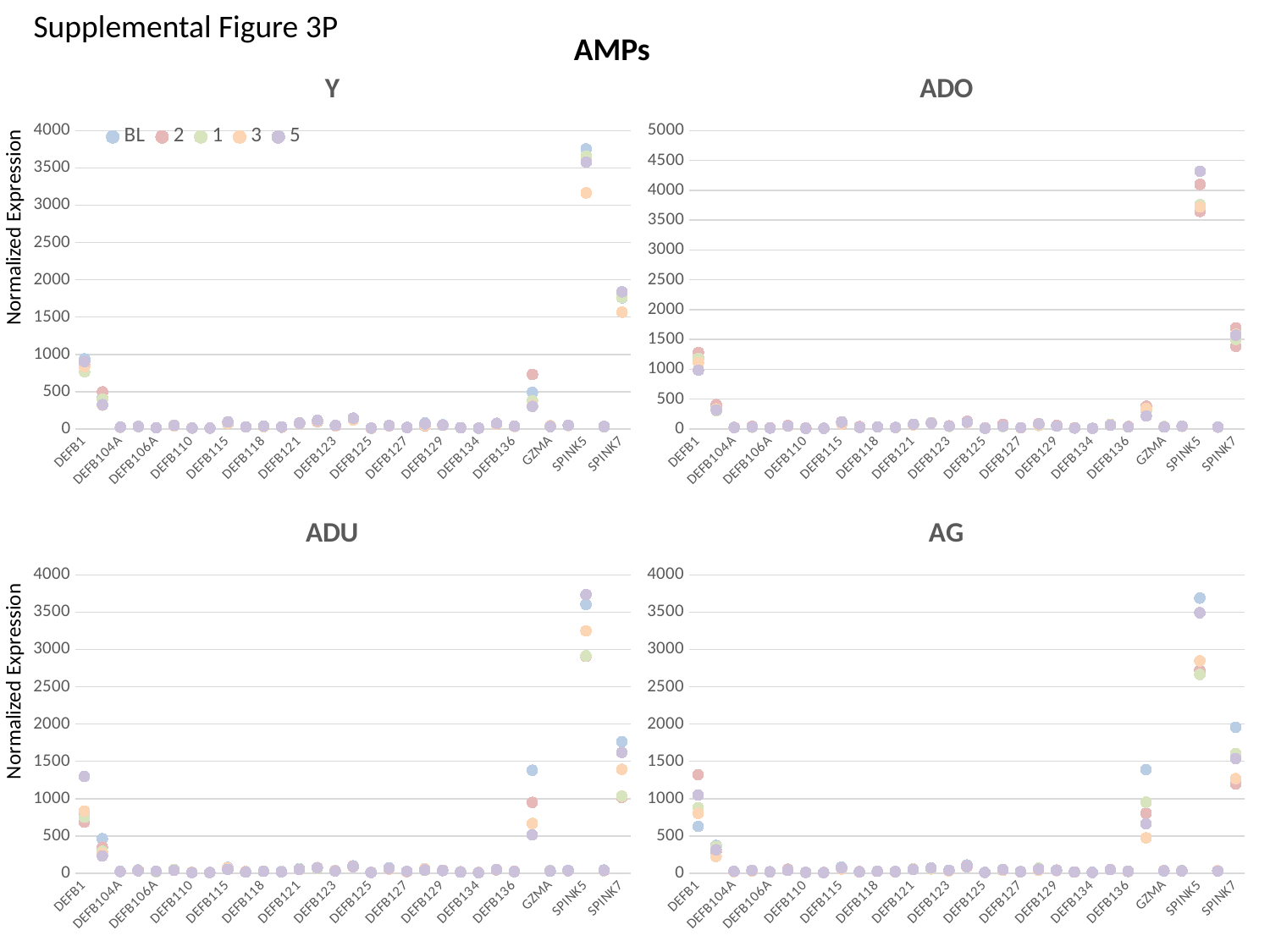

Supplemental Figure 3P
AMPs
### Chart: Y
| Category | BL | 2 | 1 | 3 | 5 |
|---|---|---|---|---|---|
| DEFB1 | 938.4957097326015 | 850.7922216511612 | 765.2545235932006 | 837.0068545279852 | 901.844258908955 |
| DEFB103A | 416.0412333408507 | 496.59566736411824 | 399.82441290707874 | 319.5105264897225 | 325.55723633876227 |
| DEFB104A | 23.87283561749702 | 23.144840132037285 | 23.46334001923044 | 22.75594632307485 | 24.666777512466606 |
| DEFB105A | 31.710069202994436 | 32.44714787290512 | 32.36619079436996 | 32.26910462549117 | 33.21701326313429 |
| DEFB106A | 16.343705553864435 | 15.53124906402512 | 17.45689000610698 | 16.388990656553446 | 15.714954974924801 |
| DEFB108B | 49.43640892953443 | 50.36132089241038 | 51.60131964371872 | 41.97947954531303 | 47.380018721812405 |
| DEFB110 | 11.903721577599025 | 11.603934981890522 | 10.848796639428206 | 13.11080005876309 | 12.54675228488868 |
| DEFB114 | 11.345101544483704 | 11.204363843469078 | 11.30777578656129 | 11.036276928387515 | 11.930001364433073 |
| DEFB115 | 91.81278853733086 | 70.4386070823181 | 73.64255661203283 | 74.97727126707122 | 95.1095476622389 |
| DEFB116 | 27.41355373578152 | 25.791544275016587 | 28.579105617888995 | 26.7696081918266 | 28.761910833734632 |
| DEFB118 | 34.171964226656286 | 31.776542241177495 | 34.2178202167884 | 34.46854579715761 | 36.90966426650855 |
| DEFB119 | 26.002196202440185 | 24.096576406574858 | 28.0938169005278 | 24.990704736446144 | 29.162246192122844 |
| DEFB121 | 82.92793278404699 | 79.92431557959613 | 73.58162767588965 | 69.59404616932136 | 75.06152819556657 |
| DEFB122 | 97.855637165611 | 96.0969015617923 | 103.97886209350531 | 107.27674020859337 | 115.76825179227573 |
| DEFB123 | 44.18960193238227 | 41.85219733924378 | 47.78711416811089 | 43.24075108970882 | 48.11750573478084 |
| DEFB124 | 122.70253573099288 | 119.44144762676723 | 121.56402496392542 | 120.8598803749596 | 144.95555125181355 |
| DEFB125 | 10.490934633738856 | 12.015391430725401 | 12.09321638161865 | 10.993425783450599 | 12.62247640072174 |
| DEFB126 | 44.17842524958458 | 39.704963928029734 | 42.970255988082684 | 38.95162584033684 | 46.17728087602385 |
| DEFB127 | 21.256749378477675 | 19.79177876644925 | 20.119177185150928 | 21.44414618618778 | 21.03806222083348 |
| DEFB128 | 79.64758535046208 | 41.574489934914446 | 49.63792692010716 | 51.810899645230705 | 67.2309244240336 |
| DEFB129 | 54.85464193212096 | 49.94739775039198 | 49.090118413781184 | 47.748497320431326 | 52.083624833042876 |
| DEFB131 | 18.226075650684177 | 16.53452618447846 | 17.7747220723288 | 18.022476242209727 | 18.538654026825768 |
| DEFB134 | 9.047847395644995 | 10.941371169518376 | 9.859111647675412 | 9.166293492992056 | 11.513797493482441 |
| DEFB135 | 63.68986134486416 | 66.54750755295282 | 68.41858006800481 | 65.79973111971202 | 75.43871989583621 |
| DEFB136 | 37.60383679307258 | 38.63872399696794 | 33.478677041554405 | 33.63677625636294 | 36.18714591146015 |
| DEFB4 | 490.07935654100453 | 730.2842250668998 | 376.4157085079071 | 314.1467687985629 | 302.93416615232474 |
| GZMA | 31.486388354223422 | 40.16488867278168 | 37.149259729616176 | 43.369475734409185 | 32.98823593721611 |
| BPIFA1 | 49.26672438830134 | 44.734478161625326 | 47.88045272377352 | 45.38674270665198 | 49.18387921473716 |
| SPINK5 | 3754.634237298245 | 3650.3206605236837 | 3659.5355503674186 | 3165.1638558506047 | 3576.862896399213 |
| SPINK6 | 34.25869399706564 | 29.4821964553281 | 28.075544328826407 | 30.453910606761813 | 30.651188961594862 |
| SPINK7 | 1758.1544808017616 | 1807.0682981131256 | 1762.2714536945177 | 1565.7625461816258 | 1838.779310820496 |
### Chart: ADO
| Category | BL | 2 | 1 | 3 | 5 |
|---|---|---|---|---|---|
| DEFB1 | 1197.0129459735483 | 1281.2665244250325 | 1181.635563021152 | 1113.1216182648864 | 984.912382270955 |
| DEFB103A | 406.9542481909806 | 362.8288898438793 | 305.74265644149324 | 336.1131146908913 | 319.48963072984446 |
| DEFB104A | 27.0934820645921 | 24.38874105931242 | 22.5284894736615 | 22.909207468772475 | 23.59442748012583 |
| DEFB105A | 45.36176100684353 | 35.75077167931295 | 30.04207397480905 | 32.128899315250166 | 35.252484867896925 |
| DEFB106A | 18.741538226228275 | 17.30910217750965 | 16.223528531462946 | 16.31319720038978 | 15.955596975912515 |
| DEFB108B | 58.2756912166777 | 53.70181333220413 | 46.26339131119218 | 46.415512327372575 | 51.3706183583529 |
| DEFB110 | 17.166881845675462 | 13.359076275154488 | 10.00166685154655 | 13.552511316257325 | 11.085651190011369 |
| DEFB114 | 12.348303296607813 | 10.937337004109118 | 10.389711784749501 | 11.232723317626059 | 10.617916819423016 |
| DEFB115 | 121.8621428766675 | 83.37743180744667 | 86.95523296857587 | 84.29295179388009 | 113.22227656909611 |
| DEFB116 | 39.674963877425334 | 28.071510374393824 | 25.11088668103074 | 27.870557448238443 | 27.18915406074458 |
| DEFB118 | 34.91151592212135 | 34.58342428635889 | 35.977239447342896 | 35.091283179808514 | 37.354379103571624 |
| DEFB119 | 26.933406517627127 | 27.462666770323878 | 25.65000342688425 | 23.574586276190033 | 25.087259331368973 |
| DEFB121 | 78.81886438605414 | 70.97452076413045 | 72.76346601814639 | 68.51190924791186 | 79.36684825943087 |
| DEFB122 | 99.76640287085193 | 107.05944040401072 | 105.83224718861182 | 99.8390968291746 | 96.32123900913852 |
| DEFB123 | 52.178461483091596 | 44.51781585356983 | 43.52349604557303 | 48.83383925550377 | 49.38785761065913 |
| DEFB124 | 126.49571216954756 | 107.50636905840061 | 113.76621796781612 | 112.24496160433716 | 115.32368139052839 |
| DEFB125 | 18.01788993830471 | 13.136997620442399 | 11.551065437491687 | 12.628603361228235 | 11.797081348706476 |
| DEFB126 | 78.61697437757961 | 53.34397389696233 | 39.86577344733461 | 46.12266973218449 | 44.9438508349541 |
| DEFB127 | 21.108673099690318 | 21.0565978335309 | 20.350769755482016 | 18.576014282336587 | 21.534403498813358 |
| DEFB128 | 89.56931375660201 | 58.78256435957542 | 57.764350101963686 | 69.92944739618946 | 83.17187426571789 |
| DEFB129 | 60.423932266776156 | 54.185776866482904 | 47.84973790721805 | 48.53718930436892 | 48.89299477019612 |
| DEFB131 | 21.712137677232644 | 16.467336749130695 | 18.355553224830537 | 17.687820965301704 | 15.518201951509601 |
| DEFB134 | 11.814563885635222 | 11.123414938181 | 9.789371493467234 | 10.691002387985954 | 11.080335111872408 |
| DEFB135 | 75.15107534816418 | 67.02298078068455 | 71.00155711445073 | 65.86058762105527 | 64.60949596162918 |
| DEFB136 | 40.110816139023356 | 35.94370858380338 | 35.52045979397613 | 33.90258075490561 | 35.17921935779231 |
| DEFB4 | 318.40895329355686 | 380.029010211555 | 303.326270175442 | 342.564256992521 | 219.0570453108032 |
| GZMA | 37.64852020739203 | 37.22012936606419 | 39.46697115406798 | 37.173619860630595 | 33.35312548517428 |
| BPIFA1 | 47.091744955466545 | 45.49128208650083 | 46.80216047803532 | 43.811774158793334 | 45.81549505990027 |
| SPINK5 | 4099.063423793185 | 3646.366381190448 | 3758.1242364517584 | 3720.162843944769 | 4320.367188761443 |
| SPINK6 | 36.21975376396143 | 32.57461157053743 | 29.921448628200867 | 32.43523047840103 | 30.9247644808355 |
| SPINK7 | 1694.9203439735927 | 1385.5879069633788 | 1499.415332045188 | 1586.7042021086656 | 1571.6524568382579 |Normalized Expression
### Chart: ADU
| Category | BL | 2 | 1 | 3 | 5 |
|---|---|---|---|---|---|
| DEFB1 | 808.5832814059535 | 685.0844631020802 | 750.2537141222336 | 834.4068946401054 | 1297.3612119611614 |
| DEFB103A | 462.2151820948793 | 348.4289623394064 | 292.4674592795258 | 262.7751068502332 | 231.80085968957187 |
| DEFB104A | 24.38504063760854 | 22.627682991363187 | 22.728694545792173 | 22.437369750559796 | 22.749578767226538 |
| DEFB105A | 44.55944311832749 | 32.63362524045325 | 36.5571275991812 | 38.29643329394127 | 34.53696593302001 |
| DEFB106A | 25.861747530671494 | 20.01187944141888 | 21.402153353275008 | 25.238502717952798 | 25.570928283279056 |
| DEFB108B | 49.879837427684244 | 46.221169039679914 | 48.425452613280164 | 39.275707543271835 | 39.831729137661036 |
| DEFB110 | 11.818610110295369 | 11.402864670045835 | 9.466650536629166 | 10.698400868902404 | 8.485515938980507 |
| DEFB114 | 9.618127881837207 | 10.145732664281311 | 8.531462728161737 | 8.661890181600182 | 8.701714683220962 |
| DEFB115 | 78.6621862646343 | 64.68366188331407 | 60.6060807676478 | 70.05039417110885 | 51.32965058674393 |
| DEFB116 | 22.301199313468842 | 20.604129165703878 | 19.337221690071065 | 19.223190331099474 | 16.253928022256105 |
| DEFB118 | 28.209163603578652 | 28.08150045842906 | 24.856397335465928 | 27.957536660809655 | 25.124869086014677 |
| DEFB119 | 21.492929189858227 | 18.613252326062696 | 19.407405335902755 | 18.866784650906684 | 18.402928188873503 |
| DEFB121 | 60.83932273178672 | 56.61707824299471 | 54.20808021690409 | 48.7463113339027 | 51.44543557017572 |
| DEFB122 | 61.96667872360044 | 63.97600740298268 | 65.90490289260589 | 75.38738662027168 | 74.22705460076062 |
| DEFB123 | 36.34241503695467 | 33.54772248074223 | 34.427191782547574 | 36.067106811548676 | 30.38848712515869 |
| DEFB124 | 98.46347017342785 | 93.71150991169145 | 89.5387059110221 | 84.57943955450986 | 88.21102189980299 |
| DEFB125 | 12.604284490175466 | 11.497111869527563 | 11.86746664699968 | 12.029012486750123 | 9.635460223651494 |
| DEFB126 | 73.21526737997877 | 58.83318317705222 | 53.93318132590105 | 57.24382732780014 | 63.26346182754438 |
| DEFB127 | 24.998343005935823 | 21.76144704462914 | 19.437515617381727 | 20.946938119137304 | 24.279248075514445 |
| DEFB128 | 61.19338923567065 | 43.841956892563644 | 36.69756712371535 | 60.63871699822312 | 43.75976934456682 |
| DEFB129 | 41.34595152672638 | 41.399663792745045 | 36.634138056588114 | 37.00540668609563 | 35.43643455029451 |
| DEFB131 | 18.72507541401129 | 19.623349322985504 | 18.14897757030126 | 14.430776531705428 | 14.15030258846189 |
| DEFB134 | 11.786038752125716 | 12.00606432674181 | 11.329989609274612 | 10.810175756839149 | 7.909759747926706 |
| DEFB135 | 50.617304712297646 | 45.2350452382402 | 48.70631622769156 | 51.37608995939834 | 47.97359177804718 |
| DEFB136 | 27.179178591410647 | 26.400123093412596 | 25.06073105091199 | 25.61970277075177 | 21.790394833509495 |
| DEFB4 | 1380.3952374981136 | 948.9430040449693 | 667.5431976422241 | 666.3629511347596 | 513.9568395630727 |
| GZMA | 26.791351939247775 | 36.93053321134482 | 36.14439954687842 | 34.73937291223963 | 31.199138224579755 |
| BPIFA1 | 37.964115470020126 | 32.706526224523614 | 32.0236560041052 | 33.62873548613813 | 33.365044164242065 |
| SPINK5 | 3602.9123529892863 | 2907.7615971025825 | 2915.886181250675 | 3248.9722959736764 | 3735.6866410058865 |
| SPINK6 | 42.30210017248176 | 33.598784138230506 | 34.952674824634954 | 36.76767074005443 | 40.15735310575414 |
| SPINK7 | 1762.8859976176996 | 1016.660789684321 | 1032.598403418134 | 1392.140247091997 | 1619.1466653682664 |
### Chart: AG
| Category | BL | 2 | 1 | 3 | 5 |
|---|---|---|---|---|---|
| DEFB1 | 626.174730279446 | 1320.4642144787454 | 880.4594491319169 | 801.9633679232896 | 1047.2564584588756 |
| DEFB103A | 371.76763093255875 | 287.4621525343946 | 358.9523330980049 | 225.12608952832716 | 314.0081486563184 |
| DEFB104A | 21.860472174358307 | 23.191735457382073 | 22.178270631946518 | 20.665801465652006 | 24.96271838077395 |
| DEFB105A | 37.04597191510322 | 32.575630600230085 | 39.98733650213745 | 30.669152220396327 | 37.815366513182056 |
| DEFB106A | 18.60584369545875 | 17.138257384839036 | 19.4812507283079 | 17.853572281811253 | 19.338192092305203 |
| DEFB108B | 53.95050317781862 | 45.55651092337349 | 39.77999858928309 | 40.63319234130599 | 36.80208429610408 |
| DEFB110 | 12.196847959903744 | 10.676149456424723 | 8.417689160391962 | 8.913385756979446 | 12.691852818112814 |
| DEFB114 | 9.872330796730527 | 9.800509199021663 | 8.006862700128202 | 7.959925614909947 | 8.999484252922315 |
| DEFB115 | 82.29184889463448 | 58.508400224682255 | 73.28968087073261 | 56.936771811158415 | 72.8557691089291 |
| DEFB116 | 20.928057785864095 | 20.114898654647604 | 18.677512805055514 | 17.40910658789584 | 19.10791427791043 |
| DEFB118 | 28.334736103092034 | 25.669822647292104 | 26.87122931133093 | 26.797684688874934 | 26.21617296610336 |
| DEFB119 | 23.69705530395221 | 19.962756038325427 | 17.320744797559325 | 18.559124272199853 | 21.177815424727346 |
| DEFB121 | 55.09113345720582 | 55.743961324163294 | 54.53431844452739 | 49.72584787740535 | 52.29514815335055 |
| DEFB122 | 70.39990365338741 | 61.139786448521555 | 57.25010492245964 | 69.29198079491823 | 71.81058451871948 |
| DEFB123 | 37.33240444441769 | 40.42001837451924 | 32.09946147952938 | 31.539947841544134 | 40.72534854384826 |
| DEFB124 | 110.14161862911583 | 97.04886382294745 | 84.38921768913495 | 85.54093104170417 | 87.68021143700241 |
| DEFB125 | 11.918896290114127 | 11.540894242386242 | 9.573465260042328 | 9.81230620784216 | 11.429624338699707 |
| DEFB126 | 37.29377813227312 | 43.966927845749666 | 52.230461801822926 | 40.57548692026282 | 50.539577209486964 |
| DEFB127 | 24.024533130537467 | 18.643795602898297 | 22.507888547511556 | 19.996409487624927 | 20.293518865289045 |
| DEFB128 | 59.000289689407005 | 43.51958076184977 | 66.76360235247054 | 38.8624213450241 | 54.75833405526241 |
| DEFB129 | 42.00067232005786 | 41.614031358276755 | 35.18555835318875 | 34.406625047551664 | 38.222928706983495 |
| DEFB131 | 18.733518101651168 | 17.130034423734365 | 16.072958046421643 | 15.235727544323359 | 15.96999105106008 |
| DEFB134 | 11.72500555051191 | 10.430841871396602 | 9.260348245523687 | 9.531521756751786 | 9.423251501609368 |
| DEFB135 | 49.359562174834444 | 50.857196311050274 | 47.52564746982834 | 49.76000373706052 | 49.17205330189375 |
| DEFB136 | 28.346424559938715 | 26.460558495340344 | 25.12928977058958 | 22.473546628120783 | 23.78935246354119 |
| DEFB4 | 1388.2956949399083 | 804.850929148613 | 951.0963000788818 | 474.20090021961937 | 662.6056546141249 |
| GZMA | 30.85393229331111 | 35.78187006906603 | 36.1150643218712 | 35.42223823940334 | 33.22866273337674 |
| BPIFA1 | 36.11827230976634 | 32.57160959872995 | 35.67967404985657 | 30.70952452376778 | 31.378753161479615 |
| SPINK5 | 3688.5664277684077 | 2720.789681747148 | 2664.757769645791 | 2847.9125042489895 | 3492.1077275871603 |
| SPINK6 | 33.394315526148596 | 33.09311370515526 | 31.599801466497475 | 33.29273911277626 | 29.571194327211074 |
| SPINK7 | 1957.4632178846607 | 1197.7869673948128 | 1602.5935600894309 | 1267.4728400976685 | 1537.1072154855594 |Normalized Expression

## Slide 24
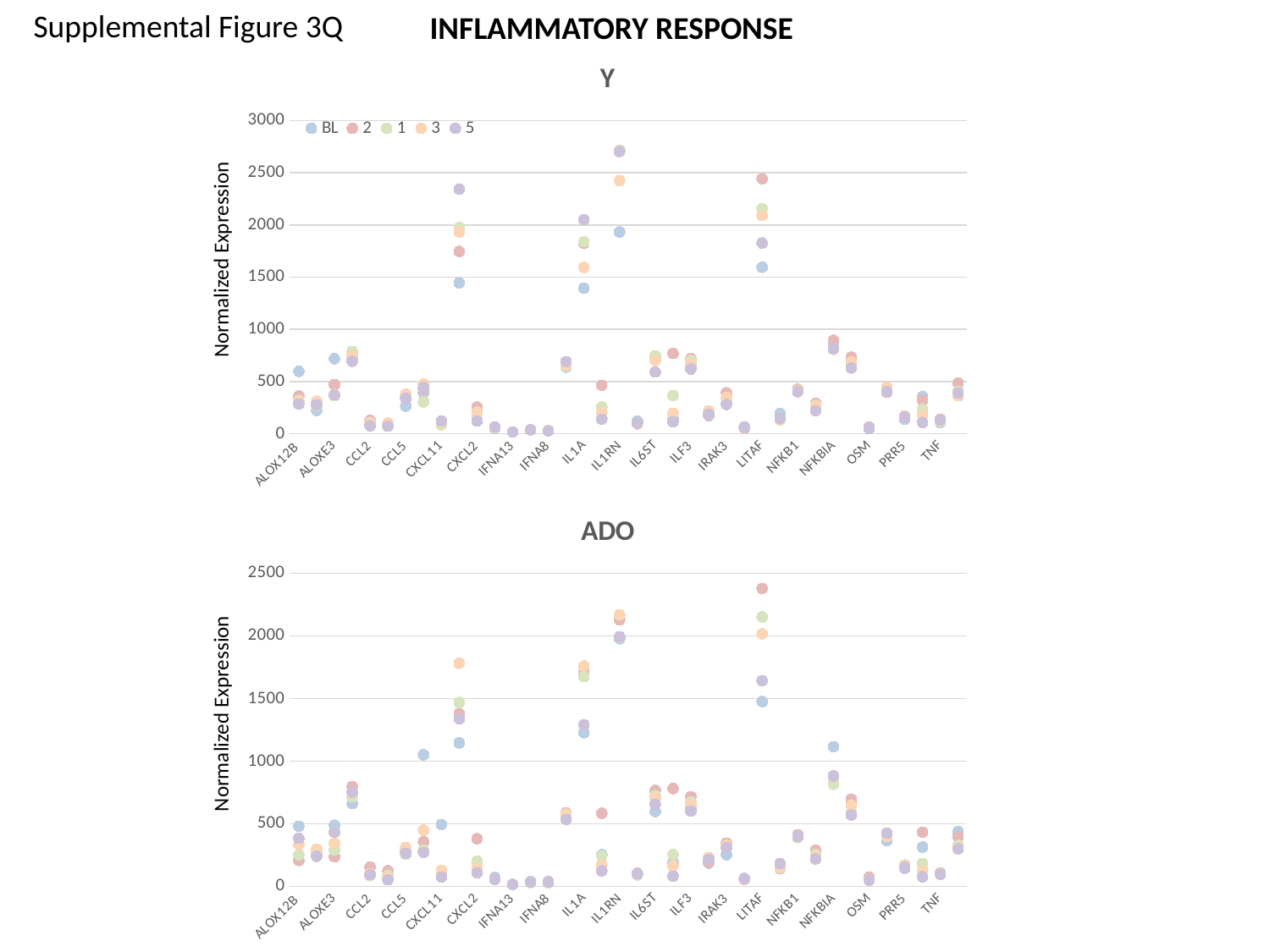

Supplemental Figure 3Q
INFLAMMATORY RESPONSE
### Chart: Y
| Category | BL | 2 | 1 | 3 | 5 |
|---|---|---|---|---|---|
| ALOX12B | 596.2825239113521 | 360.6756595850507 | 318.52005728390884 | 312.6010820772038 | 284.6328471839581 |
| ALOX5 | 222.2976135162597 | 310.1568129356548 | 267.8773469408736 | 310.3054970870333 | 276.8993348287893 |
| ALOXE3 | 718.5163973340794 | 471.48803746530007 | 364.02785889154194 | 375.47653635553934 | 370.65328270562685 |
| C1QBP | 726.4462974581802 | 747.3789365717698 | 786.8883206971068 | 745.2223785150723 | 693.5328876291101 |
| CCL2 | 83.55081932702971 | 126.58587538257744 | 106.94639388034847 | 101.35098954647124 | 74.88112789361622 |
| CCL20 | 70.91525318217383 | 94.6743164805988 | 93.08776353456486 | 102.48823679255504 | 71.11036299272297 |
| CCL5 | 263.73583172580965 | 372.55078867750063 | 366.1886135635166 | 377.5081791614714 | 338.00469584660345 |
| CXCL10 | 388.681954085144 | 426.83138625725763 | 304.63779151457203 | 474.8310305209555 | 442.7098680908244 |
| CXCL11 | 117.8196779109045 | 107.8579272467864 | 85.20236152470572 | 113.45992080508123 | 121.2249355100555 |
| CXCL17 | 1444.4520501179356 | 1745.9347467326775 | 1974.9768851995714 | 1932.3398339437144 | 2342.9735547536493 |
| CXCL2 | 133.69280593267277 | 254.3504078528605 | 205.18593851299664 | 198.92796244108303 | 120.01721088016562 |
| IFNA10 | 50.456534220490965 | 55.39134351408873 | 56.05167997026416 | 57.85721713394106 | 64.67196241277742 |
| IFNA13 | 15.533570639440963 | 16.21072923712954 | 15.418447807662425 | 15.534216980911545 | 16.034546777100275 |
| IFNA14 | 35.07570733334551 | 34.98227095478228 | 36.86789728903302 | 34.46693719757632 | 37.75081394755197 |
| IFNA8 | 29.17353812110712 | 28.287718479113774 | 29.182760038677277 | 29.223291126115416 | 26.7178906486821 |
| IKBKB | 637.0417530334998 | 667.3296102707368 | 648.2885197172277 | 658.6672643911613 | 690.4351878771199 |
| IL1A | 1394.4254284363008 | 1823.3512754201636 | 1838.7074197553177 | 1593.301665080716 | 2050.174737447165 |
| IL1B | 142.72079499585612 | 463.29204841312065 | 254.15573411048646 | 201.7644471285918 | 138.71791895112085 |
| IL1RN | 1932.2293582070788 | 2714.0519475400165 | 2716.3764204613562 | 2424.867326507418 | 2703.1985502506886 |
| IL23A | 119.90693713381961 | 94.13166539681008 | 100.15206607368961 | 97.45466124585528 | 101.51802809173677 |
| IL6ST | 589.7619667704456 | 718.54174602193 | 747.0757460429523 | 700.7173083294881 | 589.5765762308051 |
| IL8 | 138.68056736129938 | 768.9602042293817 | 365.4506792282103 | 195.744243506423 | 114.18251874430098 |
| ILF3 | 642.389697372186 | 720.1726949823613 | 700.9051727199017 | 667.9816864855732 | 618.7018117030498 |
| IRAK1 | 172.76775376045245 | 177.20291234913321 | 206.01516387457295 | 217.47637597947465 | 184.76127010911463 |
| IRAK3 | 289.0075411251971 | 390.98290860784294 | 323.7996978462154 | 346.70115467159144 | 279.8663579534212 |
| LIF | 64.44897614876373 | 50.01656638919266 | 55.74774956070991 | 52.97953656314112 | 58.90035580946793 |
| LITAF | 1594.4028248026664 | 2441.982771517616 | 2155.4368582604175 | 2089.8800625571894 | 1827.044430470647 |
| MST1R | 192.15207021630204 | 133.14586696255202 | 130.42107649692375 | 136.3723696792242 | 147.17257938898808 |
| NFKB1 | 416.63060214662863 | 424.4991702297791 | 411.64780044935077 | 409.42159429864904 | 402.0508076517056 |
| NFKB2 | 234.08137123001532 | 291.8855538686564 | 272.7934261769402 | 264.1095488857617 | 220.2023352849053 |
| NFKBIA | 873.2238778263046 | 894.215099409278 | 807.5695071047563 | 812.2908577797807 | 815.2991762192553 |
| NFKBIZ | 720.5333496518667 | 732.550316745714 | 647.4641814018676 | 684.7282329773093 | 629.6909481947748 |
| OSM | 50.115354761277615 | 64.97688959529049 | 56.0142313627459 | 55.460148040767095 | 53.43156829524262 |
| PNN | 420.05859558743157 | 395.19703663509637 | 423.25009498737955 | 443.8520934397765 | 401.63128767845706 |
| PRR5 | 138.1579632283948 | 161.45471469795785 | 162.52086767656644 | 168.6910872073198 | 161.50956142266344 |
| PTGS2 | 354.28667821680676 | 320.9894400722112 | 236.5564200783561 | 178.70806091594642 | 107.29708811124937 |
| TNF | 106.1671429731731 | 138.69861333580886 | 117.77664253994372 | 124.06521824396116 | 130.549341061071 |
| TNFAIP3 | 407.9960850666948 | 483.81771756193626 | 405.96518976481354 | 362.4835557711205 | 389.3664287871292 |Normalized Expression
### Chart: ADO
| Category | BL | 2 | 1 | 3 | 5 |
|---|---|---|---|---|---|
| ALOX12B | 479.50288455010207 | 206.49889590942752 | 252.65183457914756 | 333.04796809147564 | 382.9385599027357 |
| ALOX5 | 253.64028405273328 | 269.84784133525943 | 278.4164957524896 | 296.45156850818864 | 240.01742128235614 |
| ALOXE3 | 487.6133758761873 | 235.59015367544842 | 291.1526142363316 | 343.4298652538872 | 431.8393683891975 |
| C1QBP | 660.4834880676686 | 796.3189623874688 | 713.9472105265415 | 745.0910767951132 | 751.7741608400369 |
| CCL2 | 104.16386396878023 | 153.56728318131235 | 85.21869837488003 | 102.00549426262027 | 92.78204919755363 |
| CCL20 | 74.47931622331649 | 123.50907231151749 | 85.07042546878137 | 70.07007262344555 | 52.2604162839099 |
| CCL5 | 300.1329334723854 | 274.93664263532605 | 255.22977235596696 | 309.9930134470841 | 263.9077734944992 |
| CXCL10 | 1050.0411890693663 | 356.50755699397655 | 289.62801328131417 | 448.2015472801525 | 270.78591608533856 |
| CXCL11 | 493.91135107096125 | 118.69713737009413 | 100.51513046843066 | 125.84465454708567 | 73.53584019636509 |
| CXCL17 | 1144.8890316098596 | 1379.392794093214 | 1468.525556099074 | 1781.678526173956 | 1337.6503413354228 |
| CXCL2 | 121.41439193034469 | 379.9321973413769 | 202.27039197792467 | 147.27953510256498 | 108.25511208225065 |
| IFNA10 | 70.64636229925783 | 61.41049033991405 | 59.47902677584151 | 59.193529769093146 | 56.34106021257963 |
| IFNA13 | 16.1329274192278 | 15.320150715938098 | 16.702921376642173 | 15.473139248244173 | 14.944030844421185 |
| IFNA14 | 37.09920894389949 | 32.673000469918655 | 29.77157778242598 | 33.62229618469166 | 35.30591606860142 |
| IFNA8 | 38.3589233491187 | 35.96527165304526 | 27.22228449579974 | 31.185510117731027 | 31.789259715158227 |
| IKBKB | 540.114882172421 | 586.8260617192468 | 553.5688261380151 | 575.8508949467719 | 534.4656999915933 |
| IL1A | 1226.8133798041492 | 1713.7375309254496 | 1673.9660954182245 | 1759.4029682462287 | 1290.6001660882187 |
| IL1B | 251.97522786829234 | 582.9193419970131 | 242.31756058311643 | 172.73616838802965 | 123.58953643703255 |
| IL1RN | 1977.3251152046041 | 2128.8604364052208 | 1987.811096155449 | 2168.8146977515125 | 1993.2784416086045 |
| IL23A | 100.27827316203404 | 104.52428618442097 | 91.13664222031522 | 101.19276082854324 | 99.29833479151563 |
| IL6ST | 597.7199307169575 | 766.0305860794938 | 724.2699832689866 | 705.368169336912 | 655.508278836432 |
| IL8 | 192.0994863551605 | 780.5494306299047 | 253.34281125539096 | 163.54398096325522 | 81.13929310261099 |
| ILF3 | 624.1480850454059 | 714.8176928077523 | 673.6519857875153 | 647.5926076279143 | 601.0522587677361 |
| IRAK1 | 185.65511877491628 | 192.86795259412054 | 222.95743071019933 | 230.41576639382208 | 214.18775445912746 |
| IRAK3 | 251.96931088808697 | 344.93724730668487 | 323.76809459082557 | 327.09118075171267 | 309.148175047697 |
| LIF | 62.70534877587019 | 54.1776220318483 | 54.21552303684807 | 55.14250161023963 | 61.0283034132511 |
| LITAF | 1475.568842615755 | 2378.9256273649385 | 2151.10219935735 | 2016.6489621870185 | 1642.519747922278 |
| MST1R | 176.79348931227182 | 140.701568542692 | 147.0565403010788 | 153.4342260931245 | 182.28297639749042 |
| NFKB1 | 412.1602868319562 | 411.46871915199915 | 389.16624605460584 | 398.3747071437481 | 397.94382570904406 |
| NFKB2 | 243.1650620469468 | 287.38183875686383 | 252.2142024842303 | 238.47250296449556 | 218.8885280780214 |
| NFKBIA | 1115.3978372607983 | 873.9265411358112 | 814.9801263218136 | 872.6244322773741 | 881.0842083979442 |
| NFKBIZ | 655.2128176087631 | 696.4929296525065 | 589.3396020384856 | 647.2548280522889 | 568.8983869042642 |
| OSM | 50.783871439931005 | 74.59503757786155 | 53.841774368785416 | 52.982751813852914 | 49.86253238988277 |
| PNN | 363.3402444633432 | 403.14834328120446 | 397.1063835114386 | 399.30909056198135 | 424.1554734015139 |
| PRR5 | 143.29944273931164 | 164.00220572980572 | 170.91114065035407 | 164.34377766757578 | 151.20547052789638 |
| PTGS2 | 313.2821932263081 | 433.1728976348107 | 182.87910803881834 | 126.4671529192403 | 75.79623161338992 |
| TNF | 105.98936735510883 | 106.29060303887806 | 95.00019568225262 | 96.43235301054021 | 95.2437463317477 |
| TNFAIP3 | 437.47063648959795 | 393.74611047500764 | 326.69727494535334 | 304.3120548985465 | 299.43481898679903 |Normalized Expression

## Slide 25
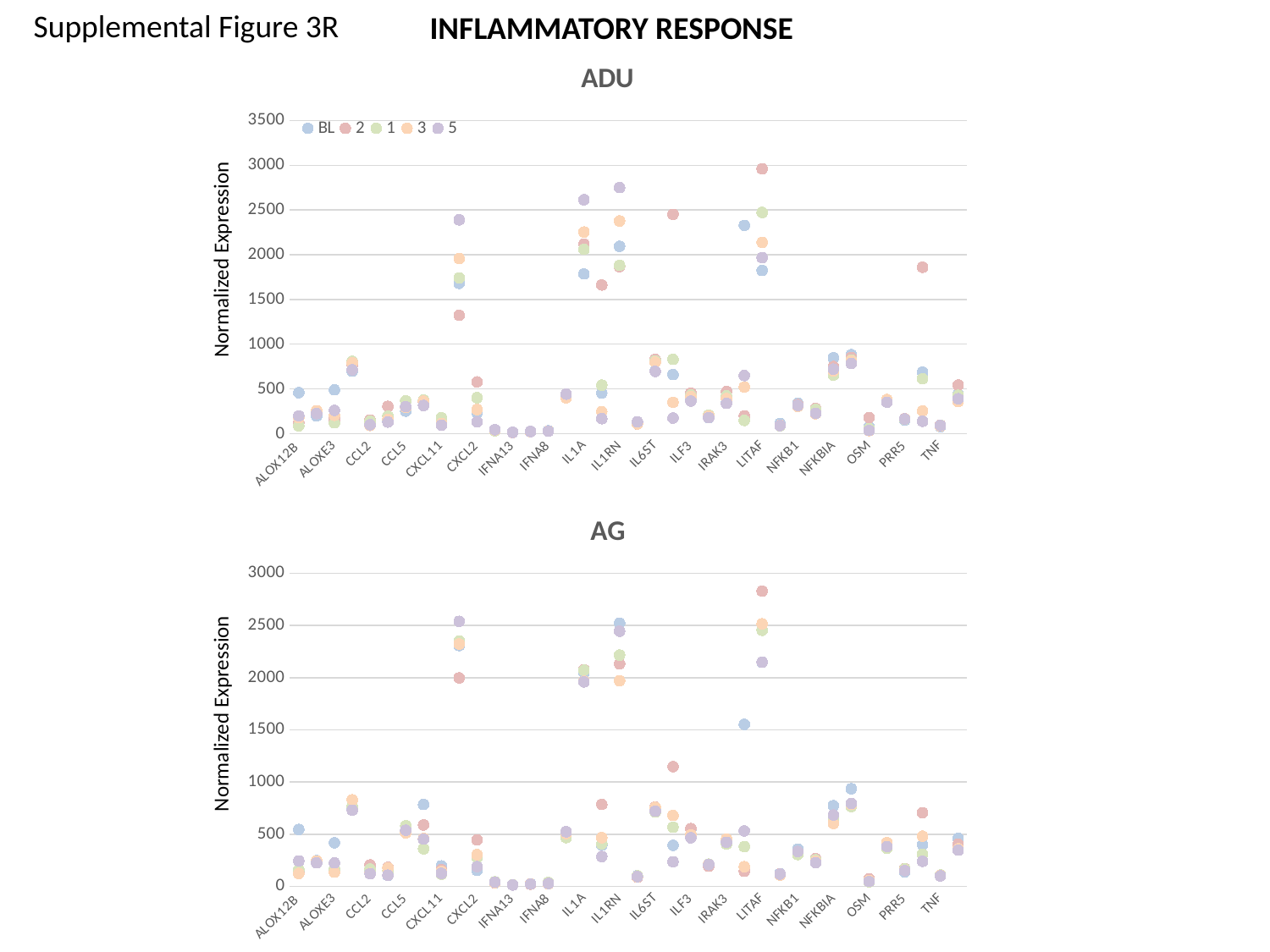

Supplemental Figure 3R
INFLAMMATORY RESPONSE
### Chart: ADU
| Category | BL | 2 | 1 | 3 | 5 |
|---|---|---|---|---|---|
| ALOX12B | 457.8302846396846 | 122.65229753747478 | 87.15957252323989 | 178.5533743893144 | 198.66038564596442 |
| ALOX5 | 197.85053223294648 | 231.8331429202402 | 232.57129979948547 | 256.2560558390182 | 224.40594861488782 |
| ALOXE3 | 489.51468130731223 | 160.45439562048648 | 120.92050436721154 | 204.90322153427167 | 259.2975068311988 |
| C1QBP | 698.9827206068578 | 769.0294260291269 | 808.3932451713332 | 792.8858241179086 | 713.0042570587925 |
| CCL2 | 113.86148413790339 | 152.88450309549256 | 139.43221544725117 | 91.48121304658041 | 99.52975458669408 |
| CCL20 | 140.4815517212683 | 304.6324993565313 | 195.34467848969712 | 158.86414470843602 | 129.11945753948794 |
| CCL5 | 251.61543349774652 | 366.3995749126638 | 367.05073803691903 | 291.9083138930807 | 301.21952259664 |
| CXCL10 | 359.14583570144055 | 355.94585368377193 | 376.6117419175261 | 363.5220110126853 | 314.60935842133836 |
| CXCL11 | 95.71887933746203 | 110.71730662097917 | 177.6460801519661 | 113.60396106216461 | 95.19965989143915 |
| CXCL17 | 1680.4997295538872 | 1321.99571380077 | 1740.9220133667602 | 1956.4601370528533 | 2390.1714814953953 |
| CXCL2 | 233.13842511196702 | 578.864492670939 | 401.1750987095968 | 269.29574566388055 | 132.7831266079185 |
| IFNA10 | 40.54788110135903 | 43.66649345469611 | 31.856753868633145 | 40.081176688959346 | 44.08228071724183 |
| IFNA13 | 15.47273718182635 | 13.909722394830046 | 14.437605229166328 | 14.434710815323188 | 14.955758201152378 |
| IFNA14 | 25.253918688006234 | 23.68012295281165 | 22.02383815222752 | 20.739845428393732 | 24.034454692207408 |
| IFNA8 | 31.52331198455397 | 29.88666751485268 | 28.78952863290875 | 28.564373180596128 | 27.261700837577582 |
| IKBKB | 430.3845868444448 | 410.42179400623996 | 423.8666973246691 | 400.9500288206399 | 441.60795752472075 |
| IL1A | 1785.2175994853244 | 2119.865576453459 | 2060.7709072156185 | 2253.553526800142 | 2614.408514930076 |
| IL1B | 454.11603174855543 | 1661.5308259398491 | 542.0538761989911 | 245.81060708723135 | 167.2486468768861 |
| IL1RN | 2093.9745444746327 | 1866.4490999646841 | 1881.5657367075178 | 2377.4540483036953 | 2750.5494443572006 |
| IL23A | 124.74168868248746 | 108.35757475925729 | 109.19789359348727 | 108.96056451400096 | 132.0075888523052 |
| IL6ST | 698.0604076115097 | 829.8268478444475 | 814.7056059609599 | 799.6461212879957 | 696.0149485274511 |
| IL8 | 659.5283552951119 | 2451.5197010925185 | 830.0285195559592 | 350.27319762304256 | 173.92129584535797 |
| ILF3 | 406.33078692842014 | 452.8603244511776 | 431.21168786745983 | 414.2866002730804 | 363.4716156831385 |
| IRAK1 | 178.37038627086534 | 180.94821142406536 | 207.45508759479355 | 196.95494949645465 | 179.6640505901594 |
| IRAK3 | 344.21594501323193 | 470.7841527399401 | 421.7087842531485 | 391.5711897545213 | 338.14349135750194 |
| LIF | 2328.9985856057165 | 199.06155078050725 | 147.8133920409376 | 521.556353338055 | 648.9186292605633 |
| LITAF | 1823.8761559484337 | 2959.9776192980553 | 2472.616876997783 | 2138.0590497272506 | 1967.0464364053287 |
| MST1R | 113.98378134319637 | 93.5436445007195 | 87.82677990028287 | 94.40425880263315 | 90.24907659534215 |
| NFKB1 | 339.8797530078156 | 323.96470369287914 | 306.7491950677391 | 304.06374468191603 | 315.4042420399211 |
| NFKB2 | 250.91196689275634 | 281.56001247978105 | 269.93944276244827 | 222.41248936970382 | 227.91714369033585 |
| NFKBIA | 846.6900195283138 | 749.8334106847542 | 654.1939094016384 | 700.9148826840952 | 721.0263964054637 |
| NFKBIZ | 881.6819178758818 | 852.6347572441409 | 822.6556430396374 | 815.155827194336 | 783.2335102794518 |
| OSM | 72.68695650018729 | 181.0287375978395 | 56.59804909830548 | 33.617617143995 | 35.28411230840365 |
| PNN | 366.35702408924203 | 357.9036696875012 | 379.01530467786523 | 381.0735385969363 | 351.7594972120012 |
| PRR5 | 149.41147318568085 | 166.23487781372208 | 160.33756775026825 | 164.06881661281196 | 163.2184867167516 |
| PTGS2 | 686.133734300221 | 1859.746773281819 | 613.0127479071889 | 253.2862332870746 | 137.7735061437649 |
| TNF | 76.93363314898562 | 95.74363683817329 | 93.13516615063834 | 84.27088897305421 | 91.08231407900087 |
| TNFAIP3 | 439.0342789347533 | 542.9526041222441 | 428.010554896034 | 358.3800966611633 | 389.5158523701341 |Normalized Expression
### Chart: AG
| Category | BL | 2 | 1 | 3 | 5 |
|---|---|---|---|---|---|
| ALOX12B | 544.5230190733337 | 132.89861471164815 | 159.08080991837758 | 123.90466768795775 | 243.380289691822 |
| ALOX5 | 247.25750680906438 | 232.5452084988262 | 227.04263867831568 | 243.9179388611781 | 224.98685710078533 |
| ALOXE3 | 417.25917435539225 | 139.80995197384453 | 161.88112480790863 | 136.12528223829534 | 224.6164425858348 |
| C1QBP | 741.9416557617635 | 765.301880223989 | 765.8497642328443 | 828.9459901923423 | 729.76111826839 |
| CCL2 | 141.37085197305998 | 204.98158355374608 | 165.9808249338135 | 123.85590918809281 | 120.7830458222805 |
| CCL20 | 104.98846437334937 | 182.87456075912903 | 150.83095856540928 | 175.85468125899638 | 107.05204035875516 |
| CCL5 | 516.6626795204324 | 516.0270835957277 | 580.1288738475604 | 512.7832775109337 | 537.616933573475 |
| CXCL10 | 783.8210068006861 | 587.8595179948996 | 360.0932792263642 | 457.77259673367644 | 452.3363259807072 |
| CXCL11 | 195.85862692105016 | 160.19856219251554 | 116.23603300826771 | 142.13039842085516 | 124.99408111416443 |
| CXCL17 | 2307.6908855707807 | 1997.7364951438005 | 2350.081762183683 | 2322.653427053814 | 2539.6054790284034 |
| CXCL2 | 152.59158995238144 | 444.7071005076696 | 277.07669268539155 | 303.33188993466297 | 188.38922329162068 |
| IFNA10 | 31.884613659641275 | 33.568894836013364 | 43.769291390852835 | 34.58657708850748 | 39.56771988995531 |
| IFNA13 | 13.579854443915549 | 15.274383419013592 | 15.253660666379094 | 13.134047090204339 | 12.989217829924337 |
| IFNA14 | 24.493077354194277 | 20.76524575942065 | 24.22551841135369 | 23.705036057035777 | 22.311272491061477 |
| IFNA8 | 24.19713408050881 | 26.51443331850268 | 37.13189314644519 | 22.807850188947096 | 28.183247506337416 |
| IKBKB | 482.3139393063858 | 511.12703323433584 | 466.6468495664969 | 505.80199257050066 | 522.8489861376324 |
| IL1A | 2048.4250185352394 | 2076.7467758211 | 2070.977856282169 | 1965.9838866967302 | 1959.3987826708399 |
| IL1B | 395.2470799694915 | 785.0324769118705 | 407.3874132767221 | 466.09128574969924 | 285.94413016982475 |
| IL1RN | 2521.622742945752 | 2132.1709718148113 | 2215.8048760669376 | 1969.9777938231264 | 2444.6877392204183 |
| IL23A | 101.94343313955095 | 86.92707547442794 | 100.73518264343177 | 90.26443490392882 | 93.2543522972575 |
| IL6ST | 724.5438939016201 | 759.8245616320596 | 711.8259080490615 | 756.5104516587116 | 720.0358897509034 |
| IL8 | 393.2845637090265 | 1146.78788439567 | 566.659651097326 | 679.2722517399366 | 235.3015078354882 |
| ILF3 | 551.0058166493666 | 552.9297793482579 | 483.97127045969376 | 493.29059067836795 | 465.04382009505855 |
| IRAK1 | 200.52190587005447 | 193.34979832329216 | 212.2400760930191 | 209.52645301676034 | 208.0263192594846 |
| IRAK3 | 421.81325269144264 | 439.47410744871155 | 405.8273862130201 | 452.98164005701824 | 422.3275744665901 |
| LIF | 1551.5341199397653 | 144.3793119789212 | 380.9974951652516 | 188.35057321332667 | 531.5855760571379 |
| LITAF | 2146.9571471006434 | 2828.9832484816325 | 2453.8984328858246 | 2515.3146746166017 | 2148.723982221743 |
| MST1R | 122.80508405022238 | 119.85686600918068 | 110.19852264432348 | 107.93143071858275 | 117.8628558810709 |
| NFKB1 | 356.9366722114543 | 330.1661318477564 | 305.10647467489673 | 334.1351749668384 | 334.10822130718503 |
| NFKB2 | 235.7072635458718 | 265.13161690294015 | 254.18976033521375 | 241.2252106005764 | 227.27096350125134 |
| NFKBIA | 773.4227542744712 | 605.955175732229 | 641.0764945852717 | 602.5770354087826 | 683.3316360044771 |
| NFKBIZ | 934.8926334176584 | 767.1093466932879 | 765.1335206848212 | 783.9650492496162 | 793.8822733643602 |
| OSM | 44.83779178931027 | 72.58400180975922 | 43.18479300089621 | 57.14801718244897 | 46.69377763440985 |
| PNN | 392.08260246999794 | 377.26150987574607 | 363.90489539541795 | 419.05098663930255 | 382.13664526304996 |
| PRR5 | 138.5330101992417 | 171.01201861001323 | 166.69705131688673 | 154.04187207106753 | 150.89690772799364 |
| PTGS2 | 399.37449923626986 | 705.3020785434178 | 308.22328593018335 | 480.23071082550604 | 239.90083272361483 |
| TNF | 105.23659547698574 | 105.57579137464856 | 108.22093334222052 | 105.39035633746694 | 100.0320603802682 |
| TNFAIP3 | 460.4742010323499 | 404.79257944623 | 351.14367661242443 | 359.36532248840734 | 346.79470653022713 |Normalized Expression
