# Supplementary material for: Comparative Analysis of Gene Expression Patterns for Oral Epithelial Cell Functions in Periodontitis
Source: Front Oral Health. 2022 May 23;3:863231. doi: 10.3389/froh.2022.863231 (PMC9169451; doi:10.3389/froh.2022.863231)
Supplement: Supplementary Table 1 — Panther pathway analysis of altered gene expression patterns that were over-represented within the epithelial cell biology using M. mulatta as the reference genome. [file Table_1.docx]

**Supplemental Table 1:** Panther pathway analysis of altered gene expression patterns that were over-represented within the epithelial cell biology using *M. mulatta* as the reference genome.

| [**PANTHER Pathways**](http://www.pantherdb.org/tools/compareToRefList.jsp?sortOrder=1&sortList=categories) | [**#**](http://www.pantherdb.org/tools/compareToRefList.jsp?sortOrder=2&sortList=Macaca%20mulatta) | | [**#**](http://www.pantherdb.org/tools/compareToRefList.jsp?sortOrder=2&sortList=Client%20Text%20Box%20Input&sortField=num) | [**expected**](http://www.pantherdb.org/tools/compareToRefList.jsp?sortOrder=2&sortList=Client%20Text%20Box%20Input&sortField=exp) | [**Fold Enrichment**](http://www.pantherdb.org/tools/compareToRefList.jsp?sortOrder=2&sortList=Client%20Text%20Box%20Input&sortField=foldEnrich) | [**+/-**](http://www.pantherdb.org/tools/compareToRefList.jsp?sortOrder=1&sortList=Client%20Text%20Box%20Input&sortField=rep) | [**raw P value**](http://www.pantherdb.org/tools/compareToRefList.jsp?sortOrder=1&sortList=Client%20Text%20Box%20Input&sortField=pval) | [**FDR**](http://www.pantherdb.org/tools/compareToRefList.jsp?sortOrder=2&sortList=Client%20Text%20Box%20Input&sortField=fdr) |
| --- | --- | --- | --- | --- | --- | --- | --- | --- |
| [Plasminogen activating cascade](javascript:openDiagramWindow('/pathway/pathwayDiagram.jsp?color=1&catsInfo=true&catAccession=P00050%27);) | [18](http://www.pantherdb.org/tools/gxIdsList.do?acc=P00050&reflist=1) | | [3](http://www.pantherdb.org/tools/gxIdsList.do?acc=P00050&list=Client%20Text%20Box%20Input&organism=Macaca%20mulatta) | .10 | 29.91 | + | 2.05E-04 | 2.72E-03 |
| [Blood coagulation](javascript:openDiagramWindow('/pathway/pathwayDiagram.jsp?color=1&catsInfo=true&catAccession=P00011%27);) | [48](http://www.pantherdb.org/tools/gxIdsList.do?acc=P00011&reflist=1) | | [6](http://www.pantherdb.org/tools/gxIdsList.do?acc=P00011&list=Client%20Text%20Box%20Input&organism=Macaca%20mulatta) | .27 | 22.43 | + | 5.32E-07 | 1.41E-05 |
| [Notch signaling pathway](javascript:openDiagramWindow('/pathway/pathwayDiagram.jsp?color=1&catsInfo=true&catAccession=P00045%27);) | [42](http://www.pantherdb.org/tools/gxIdsList.do?acc=P00045&reflist=1) | | [4](http://www.pantherdb.org/tools/gxIdsList.do?acc=P00045&list=Client%20Text%20Box%20Input&organism=Macaca%20mulatta) | .23 | 17.09 | + | 1.22E-04 | 1.95E-03 |
| [Integrin signalling pathway](javascript:openDiagramWindow('/pathway/pathwayDiagram.jsp?color=1&catsInfo=true&catAccession=P00034%27);) | [183](http://www.pantherdb.org/tools/gxIdsList.do?acc=P00034&reflist=1) | | [17](http://www.pantherdb.org/tools/gxIdsList.do?acc=P00034&list=Client%20Text%20Box%20Input&organism=Macaca%20mulatta) | 1.02 | 16.67 | + | 1.10E-15 | 8.72E-14 |
| [Alzheimer disease-presenilin pathway](javascript:openDiagramWindow('/pathway/pathwayDiagram.jsp?color=1&catsInfo=true&catAccession=P00004%27);) | [125](http://www.pantherdb.org/tools/gxIdsList.do?acc=P00004&reflist=1) | | [10](http://www.pantherdb.org/tools/gxIdsList.do?acc=P00004&list=Client%20Text%20Box%20Input&organism=Macaca%20mulatta) | .70 | 14.35 | + | 4.14E-09 | 1.64E-07 |
| [Angiogenesis](javascript:openDiagramWindow('/pathway/pathwayDiagram.jsp?color=1&catsInfo=true&catAccession=P00005%27);) | [167](http://www.pantherdb.org/tools/gxIdsList.do?acc=P00005&reflist=1) | | [11](http://www.pantherdb.org/tools/gxIdsList.do?acc=P00005&list=Client%20Text%20Box%20Input&organism=Macaca%20mulatta) | .93 | 11.82 | + | 4.57E-09 | 1.45E-07 |
| [Ras Pathway](javascript:openDiagramWindow('/pathway/pathwayDiagram.jsp?color=1&catsInfo=true&catAccession=P04393%27);) | [67](http://www.pantherdb.org/tools/gxIdsList.do?acc=P04393&reflist=1) | | [4](http://www.pantherdb.org/tools/gxIdsList.do?acc=P04393&list=Client%20Text%20Box%20Input&organism=Macaca%20mulatta) | .37 | 10.71 | + | 6.55E-04 | 8.01E-03 |
| [Cadherin signaling pathway](javascript:openDiagramWindow('/pathway/pathwayDiagram.jsp?color=1&catsInfo=true&catAccession=P00012%27);) | [123](http://www.pantherdb.org/tools/gxIdsList.do?acc=P00012&reflist=1) | | [7](http://www.pantherdb.org/tools/gxIdsList.do?acc=P00012&list=Client%20Text%20Box%20Input&organism=Macaca%20mulatta) | .69 | 10.21 | + | 8.12E-06 | 1.61E-04 |
| [Gonadotropin-releasing hormone receptor pathway](javascript:openDiagramWindow('/pathway/pathwayDiagram.jsp?color=1&catsInfo=true&catAccession=P06664%27);) | [235](http://www.pantherdb.org/tools/gxIdsList.do?acc=P06664&reflist=1) | | [13](http://www.pantherdb.org/tools/gxIdsList.do?acc=P06664&list=Client%20Text%20Box%20Input&organism=Macaca%20mulatta) | 1.31 | 9.93 | + | 1.27E-09 | 6.75E-08 |
| [CCKR signaling map](javascript:openDiagramWindow('/pathway/pathwayDiagram.jsp?color=1&catsInfo=true&catAccession=P06959%27);) | [169](http://www.pantherdb.org/tools/gxIdsList.do?acc=P06959&reflist=1) | | [9](http://www.pantherdb.org/tools/gxIdsList.do?acc=P06959&list=Client%20Text%20Box%20Input&organism=Macaca%20mulatta) | .94 | 9.56 | + | 6.72E-07 | 1.53E-05 |
| [Toll receptor signaling pathway](javascript:openDiagramWindow('/pathway/pathwayDiagram.jsp?color=1&catsInfo=true&catAccession=P00054%27);) | [57](http://www.pantherdb.org/tools/gxIdsList.do?acc=P00054&reflist=1) | | [3](http://www.pantherdb.org/tools/gxIdsList.do?acc=P00054&list=Client%20Text%20Box%20Input&organism=Macaca%20mulatta) | .32 | 9.44 | + | 4.52E-03 | 5.13E-02 |
| [PDGF signaling pathway](javascript:openDiagramWindow('/pathway/pathwayDiagram.jsp?color=1&catsInfo=true&catAccession=P00047%27);) | [140](http://www.pantherdb.org/tools/gxIdsList.do?acc=P00047&reflist=1) | | [7](http://www.pantherdb.org/tools/gxIdsList.do?acc=P00047&list=Client%20Text%20Box%20Input&organism=Macaca%20mulatta) | .78 | 8.97 | + | 1.81E-05 | 3.20E-04 |
| [EGF receptor signaling pathway](javascript:openDiagramWindow('/pathway/pathwayDiagram.jsp?color=1&catsInfo=true&catAccession=P00018%27);) | [139](http://www.pantherdb.org/tools/gxIdsList.do?acc=P00018&reflist=1) | | [6](http://www.pantherdb.org/tools/gxIdsList.do?acc=P00018&list=Client%20Text%20Box%20Input&organism=Macaca%20mulatta) | .77 | 7.75 | + | 1.59E-04 | 2.30E-03 |
| [Inflammation mediated by chemokine and cytokine signaling pathway](javascript:openDiagramWindow('/pathway/pathwayDiagram.jsp?color=1&catsInfo=true&catAccession=P00031%27);) | [273](http://www.pantherdb.org/tools/gxIdsList.do?acc=P00031&reflist=1) | | [6](http://www.pantherdb.org/tools/gxIdsList.do?acc=P00031&list=Client%20Text%20Box%20Input&organism=Macaca%20mulatta) | 1.52 | 3.94 | + | 4.66E-03 | 4.94E-02 |
| Unclassified | | [19322](http://www.pantherdb.org/tools/gxIdsList.do?acc=UNCLASSIFIED&reflist=1) | [67](http://www.pantherdb.org/tools/gxIdsList.do?acc=UNCLASSIFIED&list=Client%20Text%20Box%20Input&organism=Macaca%20mulatta) | 107.68 | .62 | - | 5.69E-20 | 9.04E-18 |
